# β‑Substituted Styrenes in Heteroaryl-Directed Hydroalkylative Cross-Couplings: Regio‑, Diastereo‑, and Enantioselective Formation of β‑Stereogenic Tertiary Alcohols

**DOI:** 10.1021/jacs.5c17840

**Published:** 2025-11-13

**Authors:** Wenbin Mao, Craig M. Robertson, John F. Bower

**Affiliations:** Department of Chemistry, 4591 University of Liverpool, Crown Street, Liverpool L69 7ZD, United Kingdom

## Abstract

Under iridium-catalyzed conditions, 2-azaaryl-substituted secondary alcohols undergo C­(sp^3^)–H addition reactions to β-substituted styrenes to provide alkylated tertiary alcohols. The processes occur with high regio-, diastereo-, and enantioselectivity and offer unusual examples of 1,2-disubstituted styrenes engaging in α-selective and stereocontrolled C–H addition reactions.

## Graphical abstract

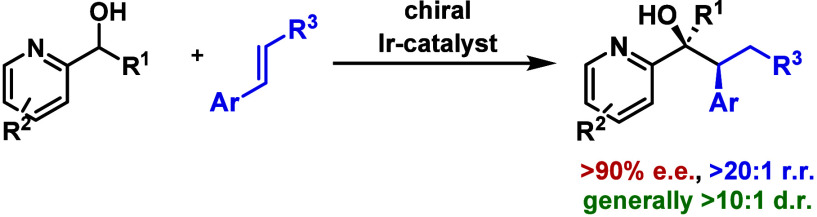

Although rare, a small group of methods exist that allow the intermolecular, α-selective, and enantioselective addition of C–H bonds to styrenes (Scheme 1A). These processes are significant because they offer an atom and step economical framework for executing challenging C–C cross-couplings.^1,2^ Within this context, asymmetric C­(sp^2^)–H additions are most established, and include Rajanbabu’s C–H additions of ethylene,^3^ directing group mediated hydroarylation^4^ and alkenylation reactions,^5^ and Ir-aza-enolate-based processes.^6^ By contrast, protocols that can effect C­(sp^3^)–H additions are much rarer, with our recently developed Ir-enolate-based methods offering unique examples.^7^ A general observation is that these C­(sp^2^/sp^3^)–H addition processes become more challenging as the styrenic alkene becomes more highly substituted.^8^ Indeed, although progress has been made in developing regioselective and enantioselective C–H additions to 1,1-disubstituted styrenes,^3,4c,4d,7c^ analogous processes that leverage 1,2-disubstituted variants have, to the best of our knowledge, not been described.

**Scheme 1:**
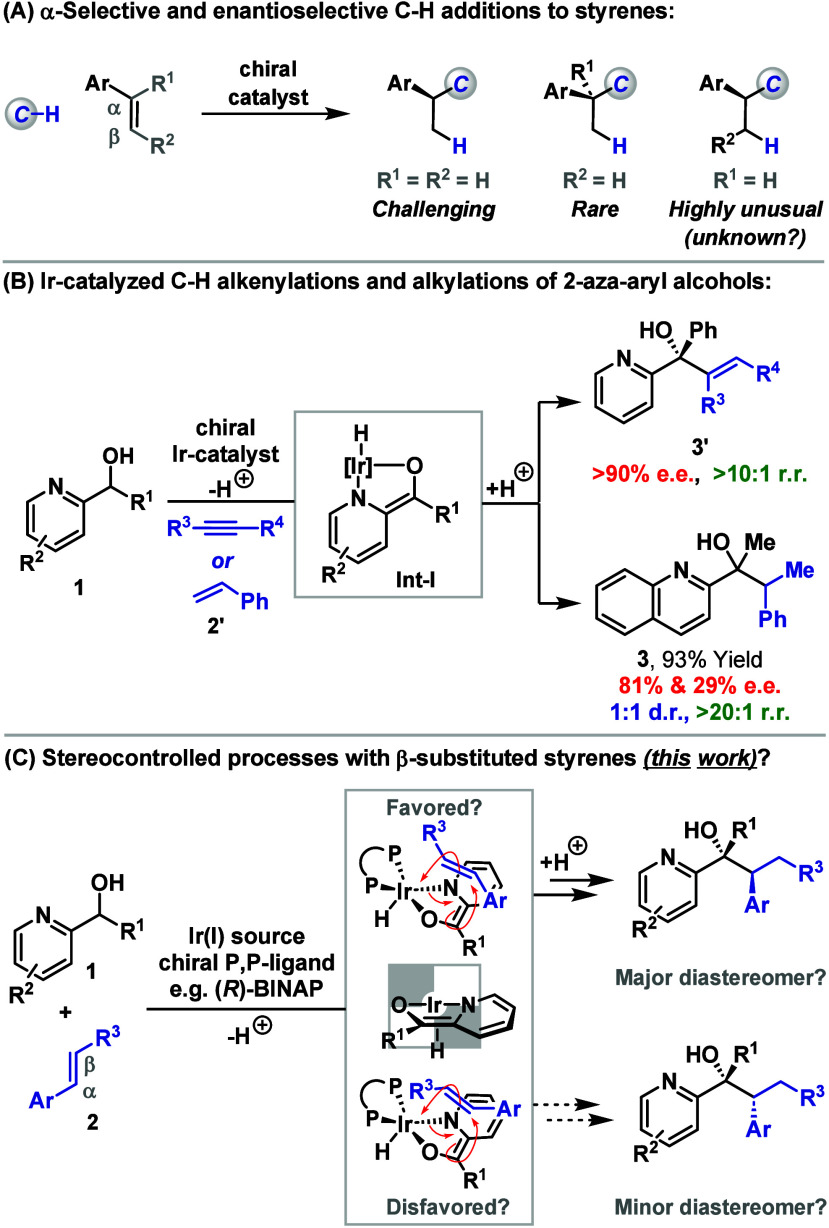
Introduction

We have previously demonstrated that 2-azaaryl alcohols **1** can undergo regioselective and enantioselective C­(sp^3^)–H additions to alkynes to provide **3′** under iridium-catalyzed conditions (Scheme 1B).^9^ This offers an unusual C­(sp^3^)–H activation-based method for upgrading racemic secondary alcohols to enantioenriched tertiary derivatives. Although speculative, one rationalization of these processes is that C–C bond formation occurs via inner sphere addition of the alkyne to an Ir-aza-enolate (**Int-I**). Preliminary efforts to replace the alkyne with styrene **2′** revealed that enantioselective C–C bond formation to provide **3** was feasible, but this promise was compromised by low diastereocontrol (1:1 d.r.). To address this, we considered whether the catalyst system might be able to tolerate more challenging 1,2-disubstituted styrenes **2** (Scheme 1C). In turn, we hoped that higher diastereoselectivity might be achieved because the β-R^3^-substituent would project toward the homochiral P,P-ligand of the Ir-catalyst, resulting in a more highly ordered carbometalation step (a cartoon representation is depicted).^10^ As described below, this hypothesis has led to highly regioselective, enantioselective and diastereoselective C­(sp^3^)–H additions to β-substituted styrenes. Beyond the area of alkene functionalization, these processes are also unusual because they offer a stereocontrolled method where internal alkenes are used as alkylating agents for alcohol C–H functionalization.^8a,8c,11^

In preliminary studies, we evaluated the Ir-catalyzed C­(sp^3^)–H addition of **1a** to β-methyl styrene **2a**. Promisingly, by using [Ir­(cod)_2_]­BARF/(*R*)-BINAP (5 mol %) at 110 °C in PhMe, target **3aa** was generated in 72% isolated yield, 4:1 d.r. and 98% e.e., favoring the indicated stereoisomer (Scheme 2A). The process was completely regioselective, with C–C bond formation occurring at the α-position of **2a**. To build on this result, we sought to upgrade the diastereoselectivity via choice of diphosphine ligand. Note that previous studies using styrene established that monophosphines are not suitable.^9^ In the event, the process proceeded using a wide range of distinct diphosphines **L2**–**L12**, with complete α-selectivity maintained in all cases. The best balance between yield and stereoselectivity was achieved using **L5**, which generated **3aa** in 61% yield, >99% e.e., and 6:1 d.r.; notably, **L9** provided **3aa** in 16:1 d.r., but with diminished enantioselectivity (88% e.e.). Using **L5**, we next evaluated the choice of reaction solvent, a variable that the process proved to be highly robust toward (Scheme 2B, entries 1–10), even proceeding in the absence of solvent (entry 11). From these studies, DCB (1,2-dichlorobenzene) was deemed the optimal choice (entry 4), giving **3aa** in 76% yield, >99% e.e., and 8:1 d.r. The flexibility of the process with respect to solvent is significant and offered an avenue for optimization during subsequent scope studies (vide infra). In all cases in Scheme 2, the ketone of **1a** was observed as a side product (6–36% yield), with this oxidation process likely driven by Ir-catalyzed transfer hydrogenative reduction of **2a** to *n*-propylbenzene.^12^

**Scheme 2:**
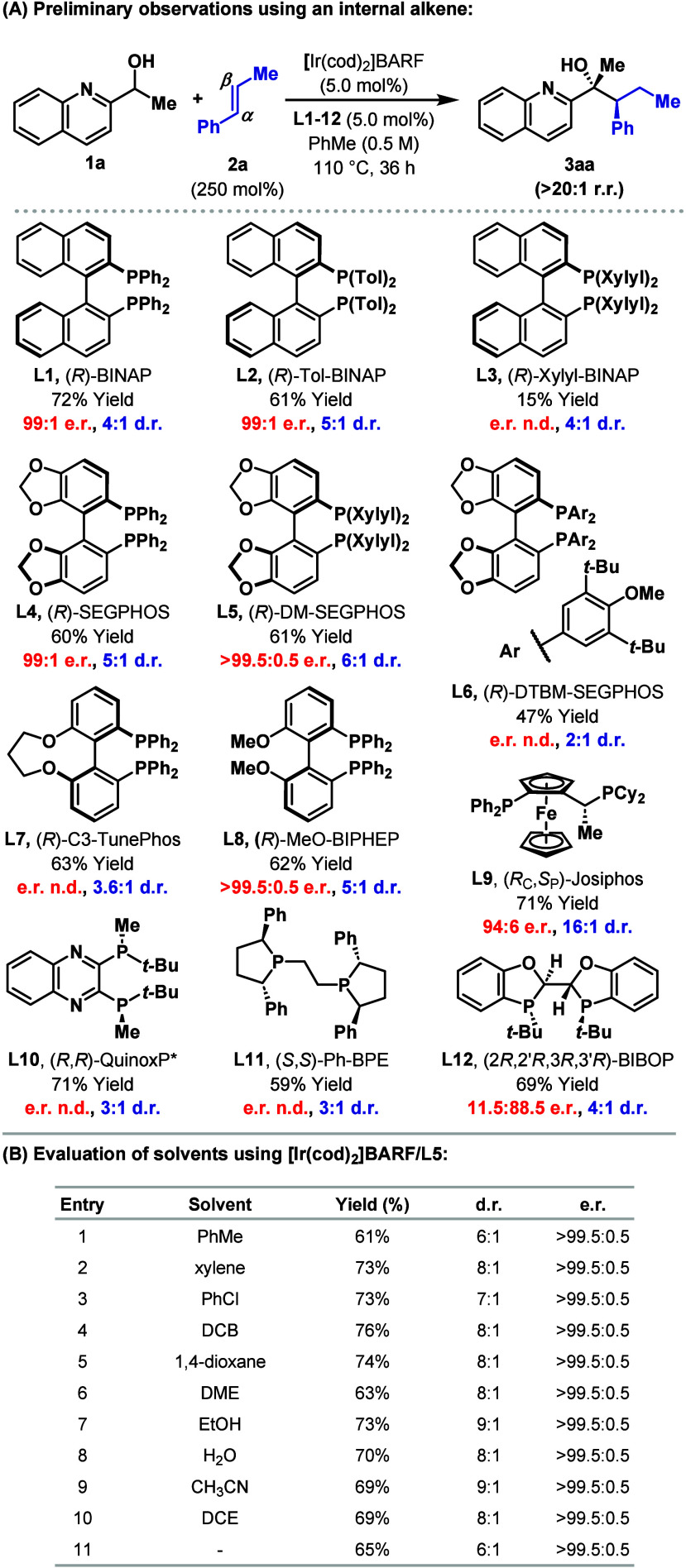
Reaction Development^a^

a All reactions were conducted on a 0.10 mmol scale. Isolated yields are reported; r.r. is the ratio of α:β selectivity and was determined by ^1^H NMR analysis of the crude mixture. 1-(Quinolin-2-yl)­ethan-1-one (i.e., the ketone of **1a**) was observed as a side product in all reactions. Further optimization results are given in the SI. **L12** gave *ent*-**3aa** as the major enantiomer.

Initial studies to explore scope focused on variations of the styrene β-substituent as outlined in Table 1A. Systems **2b**–**d**, where R = Et, (CH_2_)_2_Cl, and Bn, all reacted efficiently with **1a** to give **3ab**–**ad** in good yields and selectivities. The high yielding formation of **3ac** is of particular interest because it shows that relatively sensitive alkyl chlorides can be transferred through the process. For **2e**, where R = *i*-Pr, optimal efficiencies were achieved by switching the reaction solvent from DCB to H_2_O,^13^ and this allowed **3ae** to be isolated in 71% yield, >99% e.e., and >20:1 d.r. β-Phenyl styrene **2f** presented an interesting testbed as this system is electronically distinct to **2b**–**e**; nevertheless, the process was still efficient, delivering **3af** in 13:1 d.r. Substitution on the aromatic unit of the styrene is also well tolerated. For example, differentially positioned alkyl units, including an *o*-methyl group, were easily accommodated to provide **3ag**–**ai** in high yields. Aryl-halides (**3aj**–**al**) can be transferred without incident, and the process is relatively insensitive to the electronics of the aryl unit (**3am**–**ao**). The structure of **3al** was determined unambiguously by single crystal X-ray diffraction, and this provided the basis for the absolute and relative stereochemical assignments of the other reaction products.

**Table 1:**
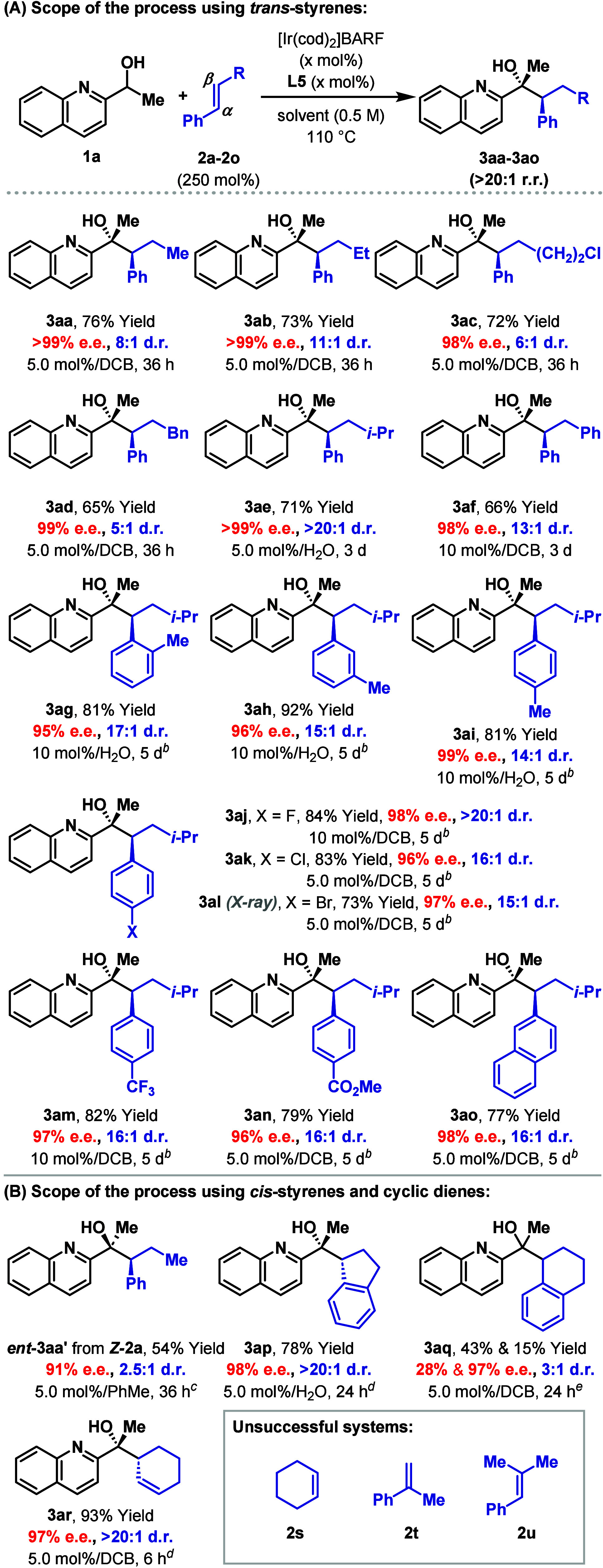
Scope of Alkenes^a^

a See the Scheme 2 footnote. Isolated yields are reported.

b *i*-PrOH (250 mol %) was added after 3 days.

c [Ir­(cod)_2_]­BARF/**L12** was used.

d [Ir­(cod)_2_]­BARF/**L11** was used.

e [Ir­(cod)_2_]­BARF/**L1** was used.

We next explored processes involving *cis*-configured styrenes (Table 1B). The hydroalkylative cross-coupling of *Z*-**2a** with **1a** occurred in 54% yield, 91% e.e., and 2.5:1 d.r. favoring diastereomer *ent*-**3aa′** over *ent*-**3aa**. Here **L12** provided the optimal balance between yield and stereoselectivity (see the SI for further details). The relatively low d.r. of this process is attributed to competitive isomerization of *Z*-**2a** to **2a** under the reaction conditions, which reacts to give *ent*-**3aa** as the minor product; this assertion is supported by a control experiment detailed in the SI. Nevertheless, this result is significant because it shows that the geometry of the alkene can be used to program the relative stereochemistry of the product. To develop more highly diastereoselective processes, we evaluated cyclic systems, because these mitigate issues associated with *cis*- to *trans*-isomerization. Pleasingly, indene **2p** reacted smoothly to provide **3ap** in high yield and e.e., and, importantly, with >20:1 d.r. using **L11**. Interestingly, extension of the method to dialin **2q** was nontrivial, and **3aq** was generated with low diastereoselectivity. In contrast, we found that 1,3-cyclohexadiene **2r** is a viable reaction partner, and this provided **3ar** in 97% e.e. and >20:1 d.r. using **L11** as the ligand (optimization results are provided in the SI).^15^ The relative and absolute stereochemistries of this product were assigned tentatively by analogy to **3ap** and related systems (vide infra). This result shows that the process can extend beyond styrenic systems to other types of conjugated alkene. Certain limitations have been identified; for example, nonconjugated alkenes, such as **2s**, and more highly substituted styrenes **2t** and **2u** are not suitable.

Based on the above, we selected indene **2p** to explore the scope of the process with respect to the heteroaryl alcohol (Table 2). These efforts focused predominantly on pyridyl-type systems, and these necessitated a reoptimization of the ligand, with **L10** and **L1** emerging as optimal choices using water as the solvent. Initially, the steric and electronic effects of the R^1^ unit were explored, and we found that the nature of this had only a minor influence on efficiency, such that **3bp**–**3ep** were accessed in high yield and stereoselectivity. Substitution on the pyridyl unit is well tolerated as evidenced by the efficient formation of C-6, C-5 and C-4 methylated systems **3fp**–**3hp**. The relative and absolute stereochemistries of **3ep** and **3fp** were assigned by single crystal X-ray diffraction. Electron-donating (**3ip**) and electron-withdrawing groups (**3jp**–**3np**) can also be accommodated, and this encompasses synthetically versatile halide units. An isoquinoline-based system participated smoothly to provide **3op**. Five-membered heteroarenes (e.g., **1r**) are not suitable, and the presence of an appropriately positioned N-center and OH unit is critical, with no cross-coupling observed using **1t**, **1u**, **1v**, or **O-Me-1b**. Alcohols **1p** and **1q** were also not amenable to cross-coupling; a rationalization for the failure of these is that the *t*-butyl unit (**1p**) and the C3-methyl substituent (**1q**) disfavor the formation of the putative Ir-aza-enolate due to developing A­(1,3)-type interactions (cf. **Int-I** in Scheme 1B).

**Table 2:**
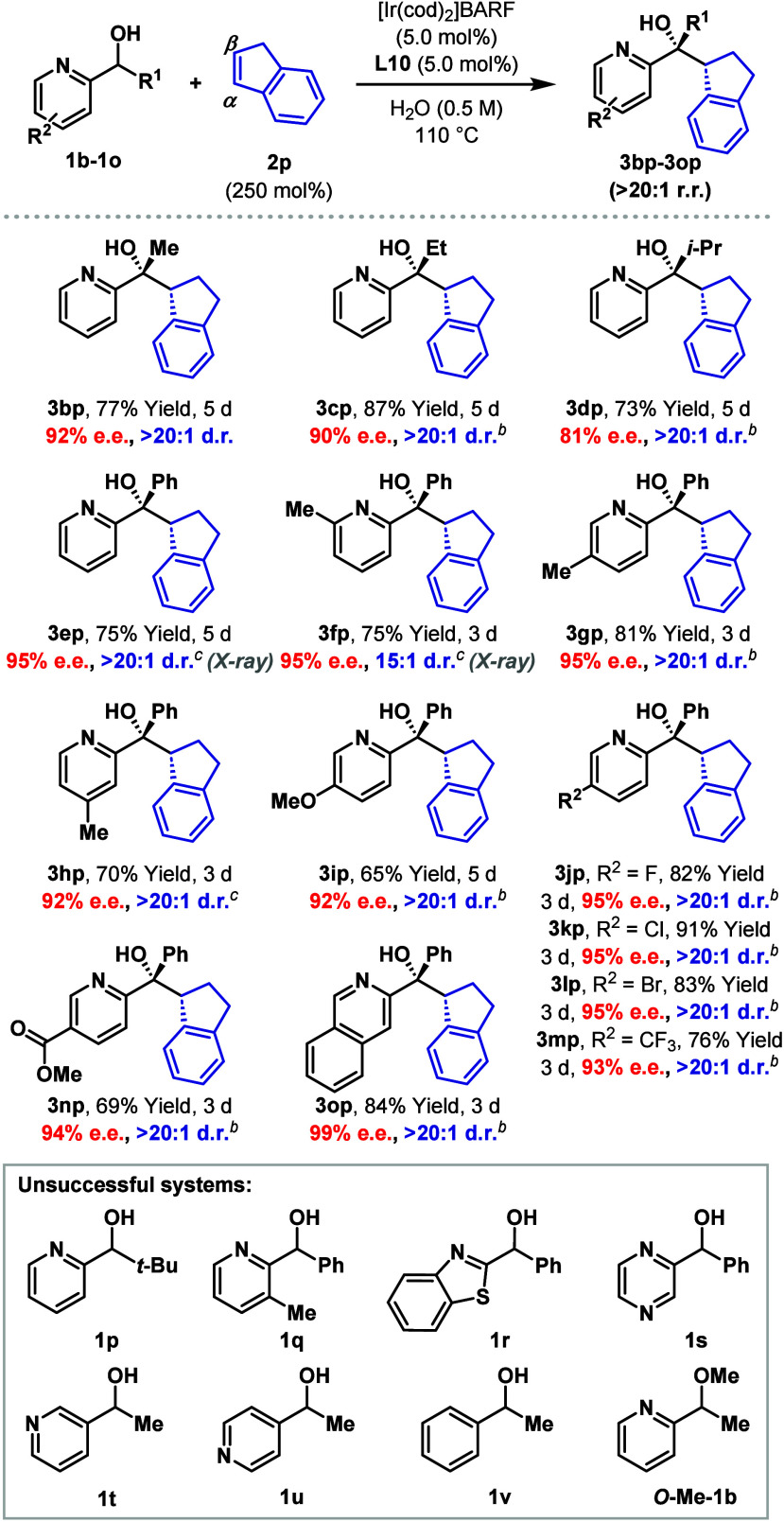
Scope of Heteroaryl Alcohols^a^

a See the Scheme 2 footnote. Isolated yields are reported.

b [Ir­(cod)_2_]­BARF/**L10** (10 mol %) was used.

c [Ir­(cod)_2_]­BARF/**L1** (5.0 mol %) was used.

The products accessed in this study are amenable to potentially useful derivatizations (Scheme 3). For example, Ir-catalyst controlled semihydrogenation of the quinoline ring of **3aa** and **3aa′** provided **4a** and **4b** with very high diastereocontrol.^16,17^ Thus, sequential C–H addition and hydrogenation processes can be used to assemble these complex systems with complete atom economy. **3ac** cyclized efficiently under basic conditions to provide tetrahydropyran **5** in 91% yield; this demonstrates the value of being able to transfer alkyl chlorides through the C–H addition process.

**Scheme 3:**
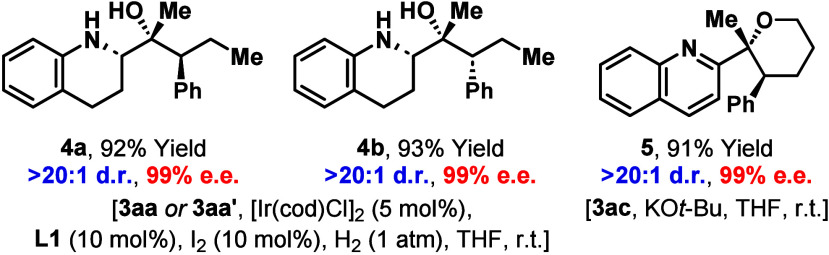
Derivatizations

At the current stage, and although other options cannot be discounted, we are primarily considering two distinct mechanistic frameworks to account for the processes described here (Scheme 4A). *Path a* is in accord with the proposition outlined in Scheme 1B, where 2-azaaryl-directed oxidative addition of the O–H bond of **1** to the Ir-catalyst is followed by aza-enolization to give **Int-I**. Carbometalation then occurs via 1,4-addition of the alkene to the Ir-aza-enolate to give **Int-II** (cf. Scheme 1C). The product **3** is then released by reductive elimination and protodemetalation. The carbometalation process, although unusual, is precedented in stoichiometric studies,^18^ and is invoked in other Ir-(aza)-enolate-promoted C–C bond formations.^6,7^ The α-selectivity likely reflects electronic effects associated with the aryl unit of **2**; the results in Table 1A show that steric effects have minimal influence. The viability of “soft” aza-enolization to give **Int-I** is supported by the deuterium exchange experiment shown in Scheme 4B.^9,19^

**Scheme 4:**
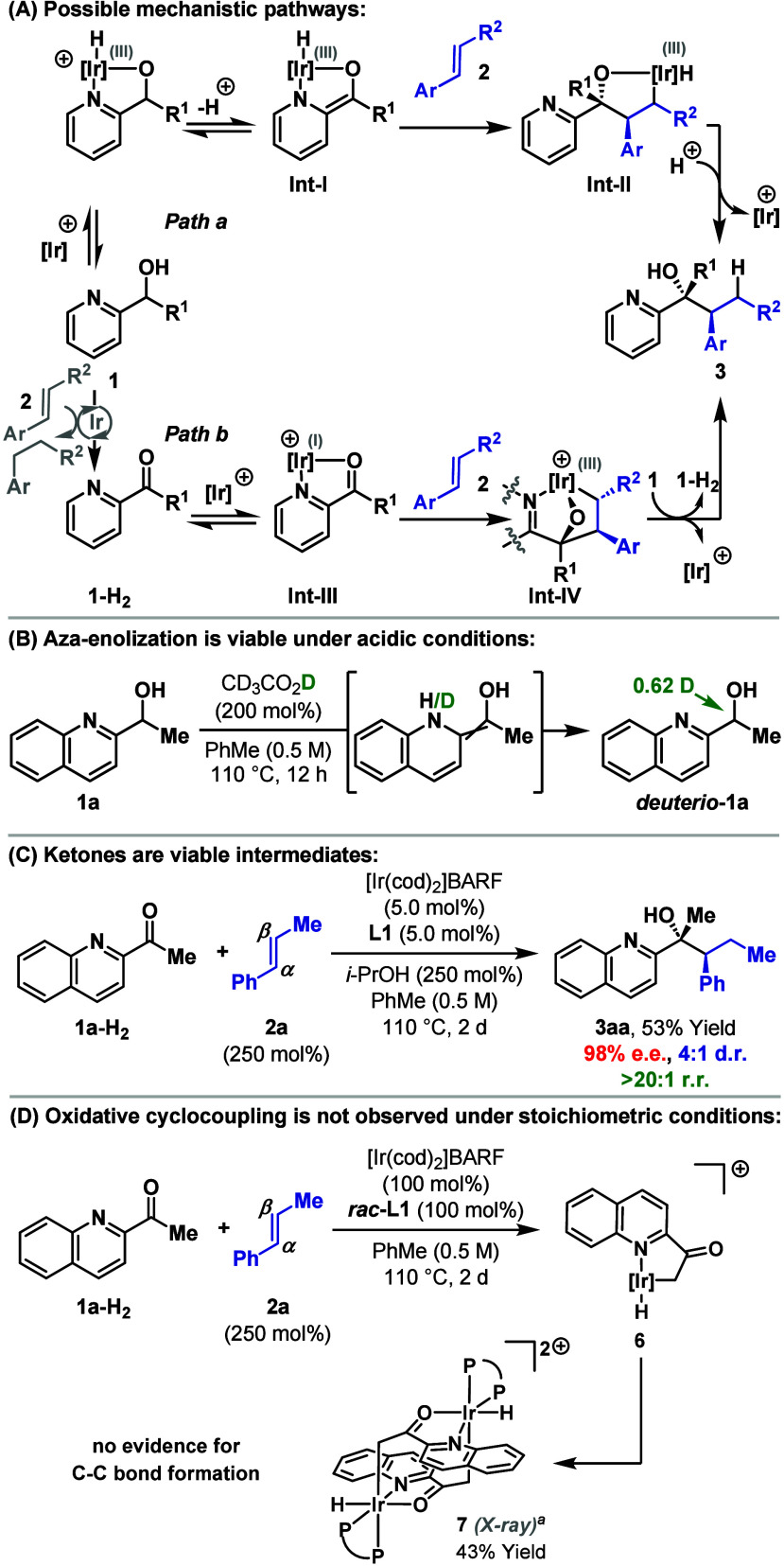
Mechanistic Considerations

a For clarity, the P,P-ligands (*rac*-**L1**) have been simplified and the BARF counterions have been omitted in the structure of **7**.

As noted earlier, the major side product of the reactions described here is **1-H**_**2**_ (the ketone of **1**) and this likely forms via transfer hydrogenative reduction of the alkene **2**.^12^ **1-H**_**2**_ is closely related to systems that have been employed in Ru-catalyzed oxidative cyclocoupling-based carbonylation reactions.^20^ Oxidative cyclocoupling also underpins a family of transfer hydrogenative C–C bond formations.^11,21,22^ Based on this, **1-H**_**2**_ may undergo oxidative cyclocoupling with **2** (via **Int-III**) to give **Int-IV** (*path b*, Scheme 4A). Transfer hydrogenation of this with **1** then gives **3** and generates a further equivalent of **1-H**_**2**_. We have confirmed that ketones are viable substrates by exposing **1a-H**_**2**_ and **2a** to Ir-catalyzed conditions in the presence of a *i*-PrOH as a sacrificial reductant (Scheme 4C). This experiment generated **3aa** in 53% yield, 4:1 d.r., and 98% e.e. (cf. Scheme 2A) and can be rationalized via either *path a or path b*, with the key point of difference being whether the reduction event occurs prior to (i.e., **1-H**_**2**_ to **1**) or after (**Int-IV** to **3**) C–C bond formation.^23^ To probe this, we evaluated the stoichiometric coupling of **1a-H**_**2**_ with **2a** in the absence of a reductant; here, C–C bond formation was not observed, and, interestingly, bimetallic iridacycle **7** was isolated in 43% yield. This likely arises via dimerization of monometallic iridacycle **6**, which is the product of N-directed C–H oxidative addition of the methyl group of **1a-H**_**2**_. Thus, under the experimental conditions, oxidative cyclocoupling (to give **Int-IV**) is not the favored outcome.^24^ We note that oxidative cyclocoupling based transfer hydrogenative processes with monosubstituted styrenes are challenging and have not been achieved using Ir-catalysts.^22^

In summary, we have developed an iridium-catalyzed, regio-, diastereo-, and enantioselective hydroalkylative cross-coupling of β-substituted styrenes with 2-azaaryl-substituted secondary alcohols. This method enables highly selective access to β-stereogenic tertiary alcohols, with alkene geometry providing a strategic handle for diastereocontrol. The study provides rare and conceptually distinct examples of α-selective C­(sp^3^)–H additions to 1,2-disubstituted styrenes, and contributes to a growing body of stereocontrolled, C–H activation-based alkene hydrocarbonation reactions.^3–7,9^ Notably, this chemistry illustrates how complex stereochemical architectures can be efficiently assembled, without prefunctionalization, from simple racemic alcohols and minimally functionalized alkenes.

## Supplementary Material



## Notes

Experimental details, characterization data, and crystallographic data (PDF)
