# Supplementary material for: β‑Substituted Styrenes in Heteroaryl-Directed Hydroalkylative Cross-Couplings: Regio‑, Diastereo‑, and Enantioselective Formation of β‑Stereogenic Tertiary Alcohols
Source: J Am Chem Soc. 2025 Nov 13;147(47):43261–6. doi: 10.1021/jacs.5c17840 (PMC12673576; doi:10.1021/jacs.5c17840)
Supplement: Supplementary file 1 [file ja5c17840_si_001.pdf]

β-Substituted Styrenes in Heteroaryl-Directed Hydroalkylative Cross-Couplings: Regio-, Diastereo- and Enantioselective Formation of β-Stereogenic Tertiary Alcohols

Wenbin Mao, Craig M. Robertson, and John F Bower*

Department of Chemistry, University of Liverpool, Crown Street, Liverpool, L69 7ZD, United Kingdom

John.Bower@liverpool.ac.uk

Supporting Information

Table of Contents

1 **General Information** 3

2 **Optimization of Reaction Conditions** 5

3 **Experimental Details and Characterization Data** 8

3.1 Starting Materials 8

3.2 Products 16

4 **Gram Scale Reaction** 65

5 **Derivatizations** 68

5.1 Stereoselective Quinoline Reduction 68

5.2 Intramolecular Cylization of **3ac** 76

6 **Mechanistic Studies** 78

6.1 Control Experiments 78

6.2 Deuterium Exchange Experiments 82

6.3 Visual Kinetic Analysis 95

6.4 Preparation of **7** 98

7 **NMR Spectra** 128

8 **References** 175

1 General Information

All chemicals were purchased from commercial suppliers and used as received unless otherwise stated. All solvents (CH_2Cl_2, *n*-hexane, toluene, Et_2O and THF) were dried and purified following standard procedures.

All reactions were performed in a flame-dried glassware using conventional Schlenk techniques under a static pressure of nitrogen unless otherwise stated. Liquids and solutions were transferred with syringes.

Analytical thin layer chromatography (TLC) was performed on aluminium backed 60 F_{254} silica plates.

Flash column chromatography (FCC) was performed on silica gel (*Aldrich* 40-63 μm, 230-400 mesh) using the indicated solvents.

^{1}H, ^{13}C and ^{19}F NMR spectra were recorded in $CDCl_3$ on *Bruker* AV400 and AV500 instruments. Chemical shifts are reported in parts per million (ppm) and are referenced to the residual solvent resonance as the internal standard ($CHCl_3$: δ = 7.26 ppm for ^{1}H NMR and $CDCl_3$: δ = 77.16 ppm for ^{13}C NMR). Data are reported as following: chemical shift, multiplicity (s = singlet, d = doublet, t = triplet, q = quartet, m = multiplet, m_c = centrosymmetric multiplet, br = broad signal), coupling constants (Hz), and integration. Assignments of ^{1}H NMR and ^{13}C NMR signals were made, where possible, using COSY, HSQC, HMBC, and NOE experiments. Numbering systems for NMR signal assignments are specified on the structure and are not related to those used for the compound names.

Enantiomeric excesses were determined using an *Agilent* 1290 Infinity chiral SFC, equipped with a diode array detector, under conditions stated for each compound.

Infrared (IR) spectra were recorded on on a *Perkin Elmer* Spectrum Two FTIR spectrometer as thin films or solids compressed on a diamond plate. Only selected absorption maxima ($\tilde{\nu}$) are reported in wavenumbers (cm^{-1})

Melting points were determined using *Reichert* melting point apparatus and are uncorrected.

High resolution mass spectra (HRMS) were obtained from the *University of Liverpool* mass spectrometry service, given to four decimal places. Mass spectra were recorded on *Agilent* 7200 Accurate Mass QTOF GC/MS (under condition of chemical ionization-CI) and Agilent 6540 UHD Accurate Mass Q-TOF LC/MS (under condition of electrospray ionization-ESI).

Optical rotations were measured on ADP440+ polarimeter at the concentration and temperature stated.

Compound names are generated by *ChemDraw* 16.0 software (*PerkinElmer*), following IUPAC nomenclature.

2 Optimization of Reaction Conditions[a]

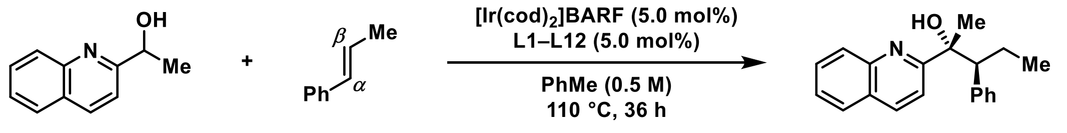

1a (0.10 mmol) 2a (250 mol%) 3aa (>20:1 r.r.)

Evaluation of ligands

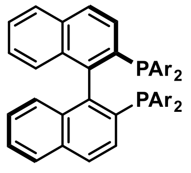

L1–L3

L1 ((*R*)-BINAP), 72% Yield, 99:1/99:1 e.r., 4:1 d.r.
L2 ((*R*)-Tol-BINAP), 61% Yield, 99:1 e.r., 5:1 d.r.
L3 ((*R*)-Xylyl-BINAP), 15% Yield, e.r. n.d., 4:1 d.r.
L4 ((*R*)-SEGPHOS), 60% Yield, 99:1 e.r., 5:1 d.r.
L5 ((*R*)-DM-SEGPHOS), 61% Yield, >99.5:0.5 e.r., 6:1 d.r.
L6 ((*R*)-DTBM-SEGPHOS), 47% Yield, e.r. n.d., 2:1 d.r.

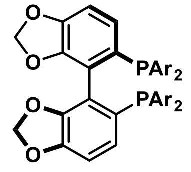

L4–L6

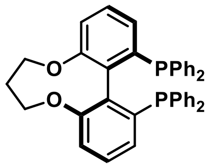

L7 ((*R*)-C3-TunePhos)
63% Yield, e.r. n.d., 3.6:1 d.r.

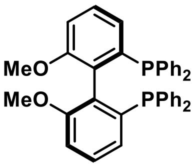

L8 ((*R*)-MeO-BIPHEP)
62% Yield, >99.5:0.5 e.r., 5:1 d.r.

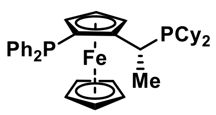

L9 (R_C,S_P-Josiphos)
71% Yield, 94:6 e.r., 16:1 d.r.

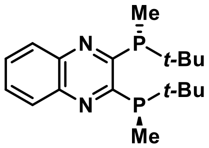

L10 ((*R*,*R*)-QuinoxP*)
71% Yield, e.r. n.d., 3:1 d.r.

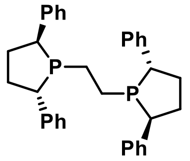

L11 ((*S*,*S*)-Ph-BPE
59% Yield, e.r. n.d., 3:1 d.r.

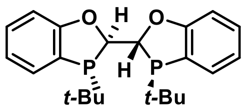

L12 ((2*R*,2'*R*,3*R*,3'*R*)-BIBOP)
69% Yield, 11.5:88.5 e.r., 4:1 d.r.

Evaluation of solvents

Entry	Ligand	Solvent	Yield	d.r.	e.r.
1	L5	PhMe	61%	6:1	>99.5:0.5
2	L5	xylene	73%	8:1	>99.5:0.5
3	L5	mesitylene	74%	7:1	>99.5:0.5
4	L5	PhCl	73%	7:1	>99.5:0.5
5	L5	DCB	76%	8:1	>99.5:0.5
6	L5	THF	trace	-	-
7	L5	1,4-dioxane	74%	8:1	>99.5:0.5
8	L5	DME	63%	8:1	>99.5:0.5
9	L5	*t*-BuOH	69%	9:1	>99.5:0.5
10	L5	*i*-PrOH	63%	8:1	>99.5:0.5
11	L5	EtOH	73%	9:1	>99.5:0.5
12	L5	H_2O	70%	8:1	>99.5:0.5
13	L5	CH_3CN	69%	9:1	>99.5:0.5
14	L5	PhCN	N.D.	-	-
15	L5	DCE	69%	8:1	>99.5:0.5
16	L5	-	65%	6:1	>99.5:0.5
17[b]	L5	DCB	40%	6:1	-

Scheme S1. Reaction Condition Screening. [a] All reactions were conducted on a 0.10 mmol scale. Isolated yields were shown. e.r. values were determined by chiral SFC analysis. r.r. is the ratio of α:β selectivity and was determined by the ^{1}H NMR of the crude mixture. d.r. values were determined by the ^{1}H NMR of the crude mixture. [b] $[Ir(cod)_2]BARF$/**L5** (3.0 mol%) was used.

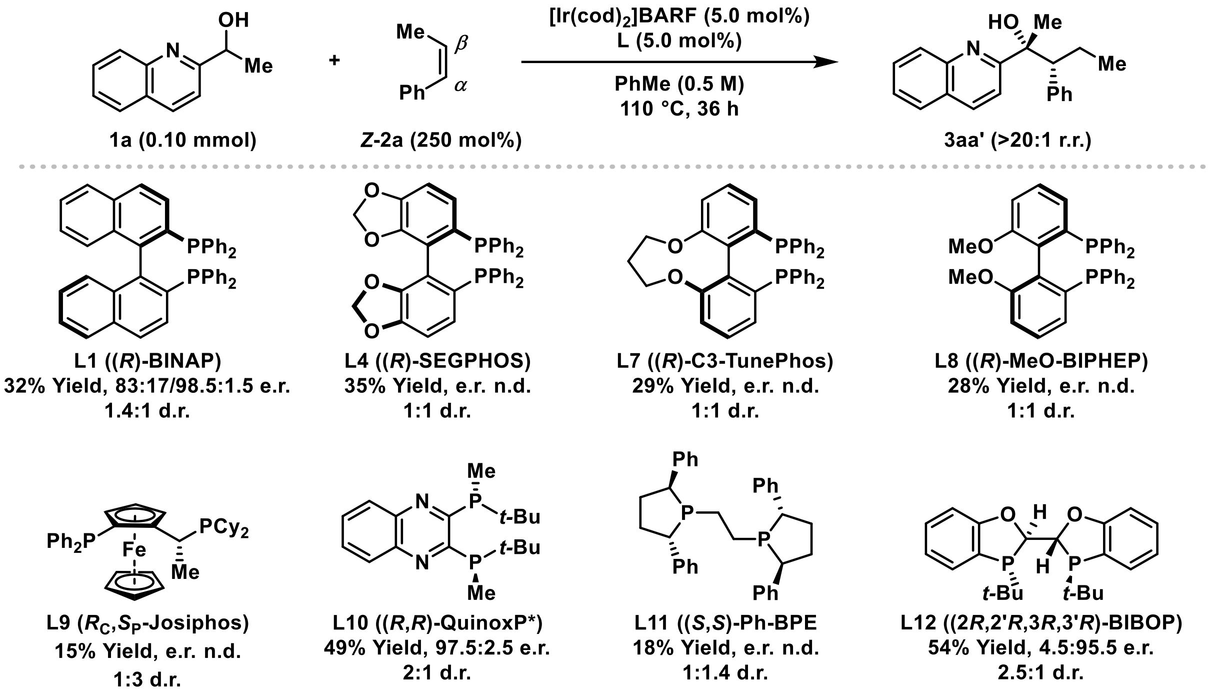

Scheme S2. Reaction Condition Screening. [a] All reactions were conducted on a 0.10 mmol scale. Isolated yields were shown. e.r. values were determined by chiral SFC analysis. r.r. is the ratio of α:β selectivity and was determined by the ^{1}H NMR of the crude mixture. d.r. values were determined by the ^{1}H NMR of the crude mixture.

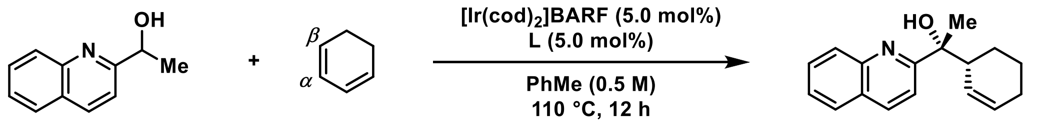

1a (0.10 mmol) 2r (250 mol%) 3ar (>20:1 r.r.)

Evaluation of ligands

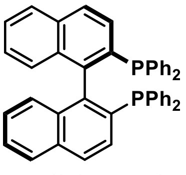

L1 ((*R*)-BINAP)
93% Yield, 72.5:27.5 e.r.
>20:1 d.r.

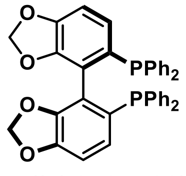

L4 ((*R*)-SEGPHOS)
69% Yield, 89.5:10.5 e.r.
>20:1 d.r.

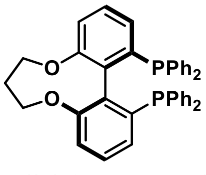

L7 ((*R*)-C3-TunePhos)
85% Yield, 89:11 e.r.
>20:1 d.r.

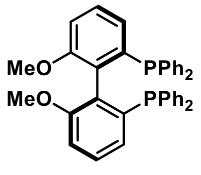

L8 ((*R*)-MeO-BIPHEP)
77% Yield, 85:15 e.r.
>20:1 d.r.

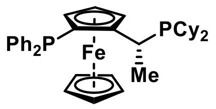

L9 (R_C,S_P-Josiphos)
49% Yield, 90.5:9.5 e.r.
>20:1 d.r.

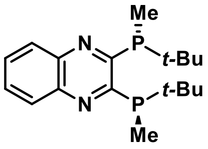

L10 ((*R*,*R*)-QuinoxP*)
77% Yield, 89:11 e.r.
>20:1 d.r.

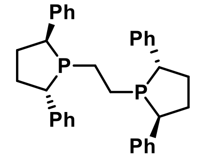

L11 ((*S*,*S*)-Ph-BPE
69% Yield, 98.5:1.5 e.r.
>20:1 d.r.

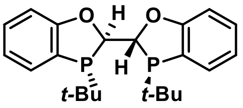

L10 ((2*R*,2'*R*,3*R*,3'*R*)-BIBOP)
76% Yield, 6:94 e.r.
>20:1 d.r.

Evaluation of solvents

entry	solvent	yield	d.r.	e.r.
1	PhMe	69%	>20:1	98.5:1.5
2	PhCl	86%	>20:1	97:3
3	DCB	93%	>20:1	98.5:1.5
4	THF	78%	>20:1	-
5	1,4-dioxane	46%	>20:1	-
6	DME	74%	>20:1	-
7	*t*BuOH	65%	>20:1	-
8	EtOH	trace	-	-
9	H_2O	94%	>20:1	98.5:1.5
10	CH_3CN	N.D.	-	-
11	PhCN	N.D.	-	-
12	DCE	68%	>20:1	-

Scheme S3. Reaction Condition Screening. [a] All reactions were conducted on a 0.10 mmol scale. Isolated yields were shown. e.r. values were determined by chiral SFC analysis. r.r. is the ratio of α:β selectivity and was determined by the ^{1}H NMR of the crude mixture. d.r. values were determined by the ^{1}H NMR of the crude mixture.

3 Experimental Details and Characterization Data

3.1 Starting Materials

GP 1: General Procedure for the Preparation of Alcohols

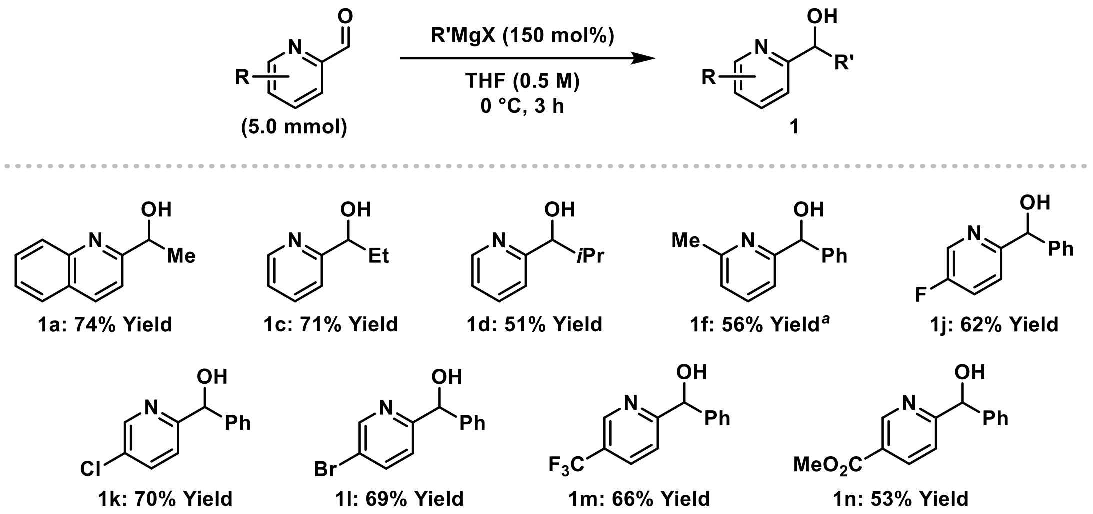

Scheme S4. Preparation of alcohols. [a] PhLi (150 mol% was used) at –78 °C for 3 h.

Alcohols were prepared according to the reported procedure.[1] A flame-dried 100 mL three-neck round-bottom flask equipped with a stirring bar was evacuated and backfilled with N_2 for 3 times. Then aldehyde (5.0 mmol, 100 mol%) and THF (10 mL, 0.5 M) were added. After cooling to 0 °C in an ice bath, Grignard reagent (0.75 mmol, 150 mol%) was added dropwise. Then the reaction mixture was stirred at this temperature for 3 h. Water (20 mL) was added to quench the reaction and the reaction mixture was extracted with ethyl acetate (3 × 20 mL). The combined organic layer was washed with brine, dried with anhydrous $MgSO_4$, filtered, and concentrated under reduced pressure. Purification by flash column chromatography (hexane/ethyl acetate from 100/1 to 3/1) afforded the desired alcohol. Data for known alcohols were in accordance with those reported.[1,2]

GP 2: General Procedure for the Preparation of Alcohols

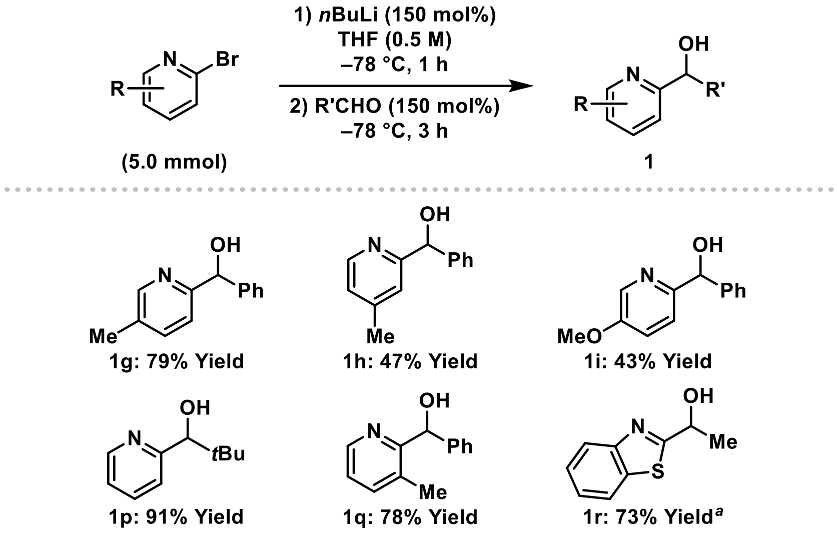

Scheme S5. Preparation of alcohols. [a] Benzothiazole or benzimidazole were used instead of bromide.

Alcohols were prepared according to the reported procedure.[3] A flame-dried 100 mL three-neck round-bottom flask equipped with a stirring bar was evacuated and backfilled with N_2 for 3 times. Then 2-bromopyridines (5.0 mmol, 100 mol%) and THF (10 mL, 0.5 M) were added. After cooling to –78 °C in a dry ice/acetone bath, *n*BuLi (3 mL, 2.5 M in hexane, 7.5 mmol, 150 mol%) was added dropwise. Then the reaction mixture was stirred at –78 °C for 1 h. Aldehyde (0.75 mmol, 150 mol%) was added. After stirring at this temperature for 3 h, water (20 mL) was added to quench the reaction and the reaction mixture was extracted with ethyl acetate (3 × 20 mL). The combined organic layer was washed with brine, dried with anhydrous $MgSO_4$, filtered, and concentrated under reduced pressure. Purification by flash column chromatography (hexane/ethyl acetate from 100/1 to 3/1) afforded the desired alcohol. Data for known alcohols were in accordance with those reported.[2a,4]

GP 3: General Procedure for the Preparation of Alcohols

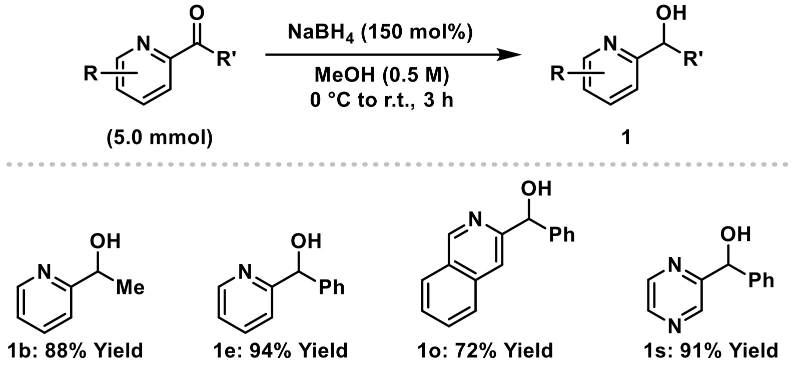

Scheme S6. Preparation of alcohols.

Alcohols were prepared according to a modified procedure.[5] A flame-dried 100 mL three-neck round-bottom flask equipped with a stirring bar was evacuated and backfilled with N_2 for 3 times. Then ketones (5.0 mmol, 100 mol%) and MeOH (10 mL, 0.5 M) were added. After cooling to 0 °C in an ice bath, $NaBH_4$ (567 mg, 0.75 mmol, 150 mol%) was added portionwise. Then the reaction mixture was stirred at room temperature for 3 h. Water (20 mL) was added to quench the reaction at 0 °C in an ice bath and the reaction mixture was extracted with ethyl acetate (3 × 20 mL). The combined organic layer was washed with brine, dried with anhydrous $MgSO_4$, filtered, and concentrated under reduced pressure. Purification by flash column chromatography (hexane/ethyl acetate from 100/1 to 3/1) afforded the desired alcohol. Data for known alcohols were in accordance with those reported.[2d,4e,6]

GP 4: General procedure for the Preparation of Internal Alkenes

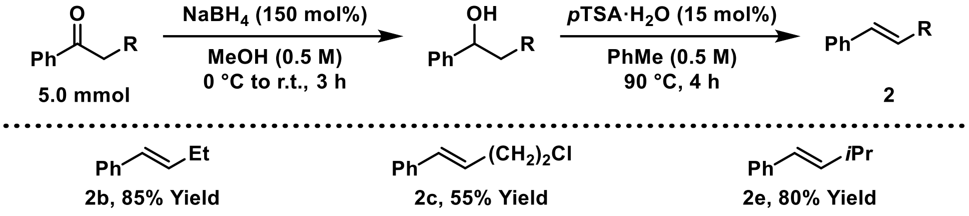

Scheme S7. Preparation of internal alkenes.

Internal alkenes were prepared according to the reported procedure.[7a] To a solution of ketone (5.0 mmol, 100 mol%) in MeOH (10 mL, 0.5 M) was added NaBH4 (284 mg, 7.5 mmol, 150 mol%) at 0 °C, and the reaction mixture was stirred at room temperature for 3 h. Then the reaction was quenched with water, and the resulting mixture was extracted with EtOAc (3 × 30 mL). The combined organic layer was washed with brine (20 mL), dried over $MgSO_4$, filtered, and concentrated under reduced pressure to afford the corresponding alcohol, which was directly used in the next step without further purification. A mixture of above alcohol (5 mmol) and *p*-toluenesulfonic acid monohydrate (143 mg, 0.75 mmol, 15 mol%) in toluene (10 mL) was stirred at 90 °C in a heating plate for 4 h. After cooling to room temperature, the reaction was quenched with aq. NaOH (2 mL, 2 M). The mixture was then diluted with water (10 mL) and EtOAc (10 mL). The aqueous layer was extracted with EtOAc (2 × 30 mL), and the combined organic layer was washed with warer (20 mL) and brine (20 mL), dried over $MgSO_4$, filtered, and concentrated under reduced pressure to give a crude product which was purified by flush column chromatography on silica gel (hexane) to give the desired product. Data for known alkynes were in accordance with those reported.[7a]

Procedure for the Preparation of 2d

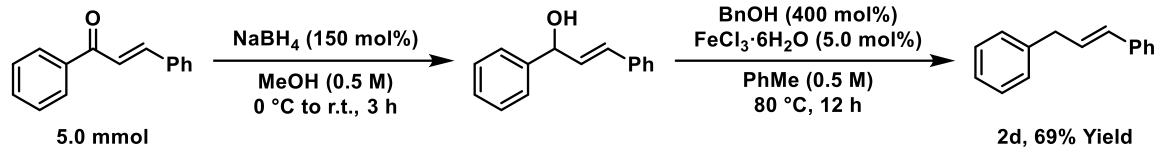

Scheme S8. Preparation of **2d**.

2d was prepared according to a modified procedure.[7b] To a solution of ketone (1.04g, 5.0 mmol, 100 mol%) in MeOH (10 mL, 0.5 M) was added NaBH4 (284 mg, 7.5 mmol, 150 mol%) at 0 °C, and the reaction mixture was stirred at room temperature for 3 h. Then the reaction was quenched with water, and the resulting mixture was extracted with EtOAc (3 × 30 mL). The combined organic layer was washed with brine (20 mL), dried over $MgSO_4$, filtered, and concentrated under reduced pressure to afford the corresponding alcohol, which was directly used in the next step without further purification. To a mixture of allylic alcohol above and benzyl alcohol (2.1 mL, 2.16 g, 20 mmol, 400 mol%) in toluene (10 mL, 0.5 M) was added $FeCl_3{\cdot}6H_2O$ (67.6 mg, 0.25 mmol, 5 mol%) and the reaction mixture was stirred at 80 °C in a heating plate for 12 h. After completion of the reaction, the mixture was quenched with saturated NH_4Cl solution. The aqueous layer was extracted with EtOAc (2 × 30 mL), and the combined organic layer was washed with warer (20 mL) and brine (20 mL), dried over $MgSO_4$, filtered, and concentrated under reduced pressure to give a crude product which was purified by flush column chromatography on silica gel (hexane) to give **2d** as a colorless oil (1.34 g, 69% yield, *E*/*Z* >20:1). Data were in accordance with those reported.[7b]

GP 5: General Procedure for the Preparation of Internal Alkenes

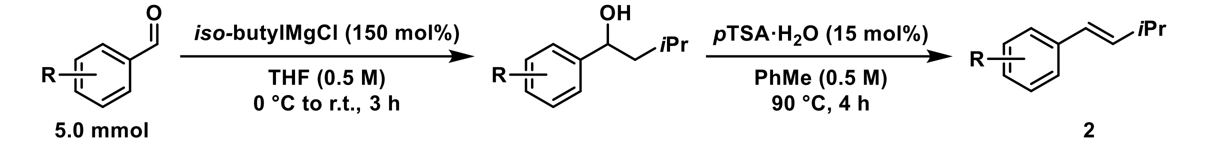

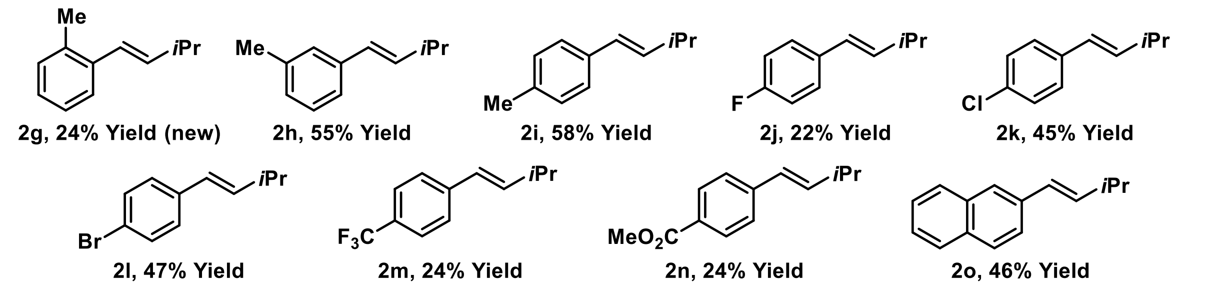

Scheme S9. Preparation of internal alkenes.

Internal alkenes were prepared according to a modified procedure.[7a] A flame-dried 100 mL three-neck round-bottom flask equipped with a stirring bar was evacuated and backfilled with N_2 for 3 times. Then aldehyde (5.0 mmol, 100 mol%) and THF (10 mL, 0.5 M) were added. After cooling to 0 °C in an ice bath, *i*BuMgCl solution (3.8 mL, 7.5 mmol, 150 mol%, 2 M in THF) was added dropwise. Then the reaction mixture was stirred at room temperature for 3 h. Water (20 mL) was added to quench the reaction and the reaction mixture was extracted with ethyl acetate (3 × 20 mL). The combined organic layer was washed with brine, dried with anhydrous $MgSO_4$, filtered, and concentrated under reduced pressure. Purification by flash column chromatography (hexane/ethyl acetate from 100/1 to 3/1) afforded the desired alcohol. A mixture of the above alcohol and *p*-toluenesulfonic acid monohydrate (143 mg, 0.75 mmol, 15 mol%) in toluene (10 mL) was stirred at 90 °C in a heating plate for 4 h. After cooling to room temperature, the reaction was quenched with aq. NaOH (2 mL, 2 M). The mixture was then diluted with water (10 mL) and EtOAc (10 mL). The aqueous layer was extracted with EtOAc (2 × 30 mL), and the combined organic layer was washed with warer (20 mL) and brine (20 mL), dried over $MgSO_4$, filtered, and concentrated under reduced pressure to give a crude product which was purified by flush column chromatography on silica gel (hexane) to give the desired product. Data for known alkynes were in accordance with those reported.[8] Data for unknown alkene **2g** are listed below.

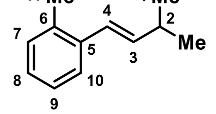

$C_{12}H_{16}$
M = 160.26

(*E*)-1-Methyl-2-(3-methylbut-1-en-1-yl)benzene (**2g**, **GP 5**) was prepared from 2-methylbenzaldehyde (578 μL, 601 mg, 5.0 mmol, 100 mol%) and *i*BuMgCl solution (3.8 mL,

7.5 mmol, 150 mol%, 2 M in THF). Purification by flash column chromatography on silica gel (hexane) as the eluent afforded **2g** (189 mg, 24% yield, *E*/*Z* >20:1) as a colorless oil.

R*f* = 0.8 (Hexane).

^{1}H NMR (500 MHz, $CDCl_3$, 298 K): δ 7.45 (d, *J* = 7.4 Hz, 1H, H-10), 7.21–7.12 (m, 3H, H-7, H-8 and H-9), 6.57 (d, *J* = 15.8 Hz, 1H, H-4), 6.10 (dd, *J* = 15.8, 6.9 Hz, 1H, H-3), 2.58–2.47 (m, 1H, H-2), 2.37 (s, 3H, H-11), 1.15 (s, 3H, H-1), 1.14 (s, 3H, H-1) ppm.

^{13}C NMR (125 MHz, $CDCl_3$, 298 K): δ 139.6 (C-3), 137.2 (C-5), 135.1 (C-6), 130.3 (C-7), 126.8 (C-8 or C-9), 126.1 (C-8 or C-9), 125.5 (C-10), 124.8 (C-4), 32.0 (C-2), 22.7 (C-1), 20.0 (C-11) ppm.

HRMS (CI) m/z: $[M+H]^+$ calcd for $C_{12}H_{17}^+$ 161.1325, found 161.1324.

IR (thin film): ṽ 2958 (s), 1460 (s), 1380 (s), 1223 (s), 965 (s) cm^{-1}.

Preparation of [Ir(cod)$_2$]BARF

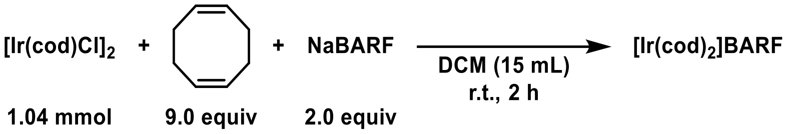

Scheme S10. Preparation of [Ir(cod)$_2$]BARF.

To a solution of chloro(1,5-cyclooctadiene)iridium(I) dimer (672 mg, 1.0 mmol, 100 mol%) and NaBARF (1.77 g, 2.0 mmol) in CH_2Cl_2 (15 mL) was added 1,5-cyclooctadiene (1.1 mL, 978 mg, 9.0 mmol, 900 mol%). The reaction mixture was stirred at room temperature for 2 h and then filtered through Celite. Solvent was removed in vacuo and the residue was dried under vacuum (0.01 mmHg) overnight to afford the desired product (1.23 g, 97% yield) as a burgundy solid. This compound was stored under argon at –20 °C. Data were in accordance with those reported.[9]

^{1}H NMR ($CDCl_3$, 500 MHz): δ 7.70 (8H, s), 7.55 (4H, s), 4.99 (8H, s), 2.47–2.32 (m, 8H), 2.32–2.19 (m, 8H) ppm.

^{13}C NMR ($CDCl_3$, 125 MHz): 161.8 (1:1:1:1 pattern, $J_{C–B}$ = 49.2 Hz), 134.9, 129.1 (qq, $J_{C–F}$ = 31.3 Hz, $J_{C–F}$ = 2.9 Hz), 124.7 (q, $J_{C–F}$ = 270.8 Hz), 117.7, 101.2, 30.5.

^{19}F NMR ($CDCl_3$, 471 MHz): δ –62.3 ppm.

3.2 Products

GP 6: General Procedure for Ir-catalysed asymmetric addition reaction

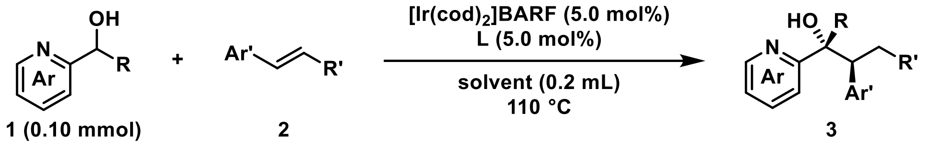

Scheme S11. Ir-catalysed asymmetric addition reaction.

A flame-dried Schlenk tube was charged with alcohol (0.10 mmol, 100 mol%), [Ir(cod)$_2$]BARF (5.0 µmol, 5.0 mol%) and **L** (5.0 µmol, 5.0 mol%). The Schlenk tube was evacuated and backfilled with N_2 for three times. Then solvent (0.2 mL, 0.5 M) was added, followed by the addition of alkene (0.25 mmol, 250 mol%). The tube was sealed and stirred at 110 °C in a heating plate for the indicated time. After cooling to room temperature, the solvent was removed under reduced pressure and the crude reaction mixture was purified by flash column chromatography under the indicated conditions.

Note: All *racemic* compounds are synthesized according to the method outlined above, by using (±)-BINAP or (±)-DM-SEGPHOS.

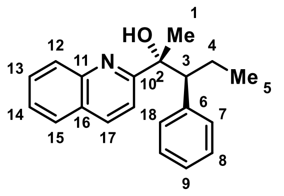

$C_{20}H_{21}NO$
M = 291.39

(2*R*,3*S*)-3-Phenyl-2-(quinolin-2-yl)pentan-2-ol (**3aa**, **GP 6**) was prepared from 1-(quinolin-2-yl)ethan-1-ol (**1a**, 17.3 mg, 0.10 mmol, 100 mol%) and (*E*)-1-propenylbenzene (**2a**, 32.4 µL, 29.5 mg, 0.25 mmol, 250 mol%), using [Ir(cod)$_2$]BARF (6.36 mg, 5.0 µmol, 5.0 mol%) and **L5** ((*R*)-DM-SEGPHOS, 3.61 mg, 5.0 µmol, 5.0 mol%) in DCB (0.2 mL) at 110 °C for 36 h. Purification by flash column chromatography on silica gel (hexane/ethyl acetate from 100/1 to 19/1) afforded **3aa** (22.2 mg, 76% yield, >99% e.e., >20:1 d.r., >20:1 r.r.) as a colorless solid. Diastereomer and regiomer ratios (8:1 d.r., >20:1 r.r.) were determined from the ^{1}H NMR spectrum of crude material.

R*f* = 0.4 (Hexane/EtOAc = 9/1).

M.P. 151–152 °C (Hexane/EtOAc).

^{1}H NMR (500 MHz, $CDCl_3$, 298 K): δ 8.18 (d, *J* = 8.6 Hz, 1H, H-17), 8.13 (d, *J* = 8.5 Hz, 1H, H-12), 7.86 (d, *J* = 8.1 Hz, 1H, H-15), 7.78–7.74 (m, 1H, H-13), 7.60–7.55 (m, 1H, H-14), 7.46–7.40 (m, 3H, H-7 and H-18), 7.36–7.31 (m, 2H, H-8), 7.29–7.24 (m, 1H, H-9), 6.08 (s, 1H, OH), 2.85 (dd, *J* = 11.7, 3.1 Hz, 1H, H-3), 1.87–1.75 (m, 1H, H-4), 1.34 (s, 3H, H-1), 1.22–1.13 (m, 1H, H-4), 0.54 (t, *J* = 7.4 Hz, 3H, H-5) ppm.

^{13}C NMR (125 MHz, $CDCl_3$, 298 K): δ 165.2 (C-10), 145.7 (C-11), 141.4 (C-6), 137.4 (C-17), 130.2 (C-7), 130.0 (C-13), 129.0 (C-12), 128.1 (C-8), 127.6 (C-15), 127.2 (C-16), 126.6 (C-14), 126.6 (C-9), 117.6 (C-18), 76.3 (C-2), 58.7 (C-3), 28.0 (C-1), 23.1 (C-4), 12.6 (C-5) ppm.

HRMS (ESI) m/z: $[M{-}H_2O{+}H]^+$ calcd for $C_{20}H_{20}N^+$ 274.1590, found 274.1601.

IR (thin film): ṽ 3332 (s), 1598 (s), 1504 (s), 1366 (s), 1151 (s), 1058 (s) cm^{-1}.

Specific rotation: $[\alpha]_D^{22}$ = −35.5 (*c* 0.5, CH_2Cl_2).

The **enantiomeric ratio** of **3aa** was determined by SFC analysis (CHIRALPACK OD-H (25 cm), column temperature 25 °C, solvent CO_2/MeOH = 90/10, flow rate = 2.0 mL/min): t_R = 5.6 min (minor), t_R = 6.4 min (major).

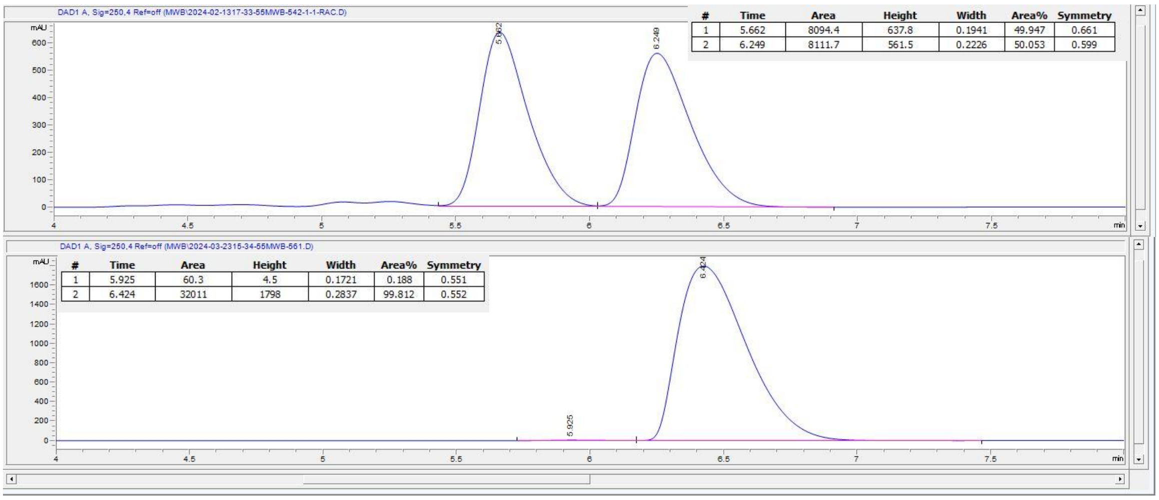

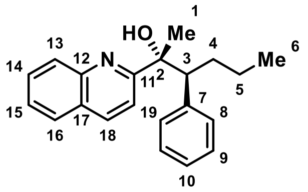

$C_{21}H_{23}NO$
M = 305.42

(2*R*,3*S*)-3-Phenyl-2-(quinolin-2-yl)hexan-2-ol (**3ab**, **GP 6**) was prepared from 1-(quinolin-2-yl)ethan-1-ol (**1a**, 17.3 mg, 0.10 mmol, 100 mol%) and (*E*)-but-1-en-1-ylbenzene (**2b**, 33.1 mg, 0.25 mmol, 250 mol%), using $[Ir(cod)_2]BARF$ (6.36 mg, 5.0 μmol, 5.0 mol%), and **L5** ((*R*)-DM-

SEGPHOS, 3.61 mg, 5.0 μmol, 5.0 mol%) in DCB (0.2 mL) at 110 °C for 36 h. Purification by flash column chromatography on silica gel (hexane/ethyl acetate from 100/1 to 19/1) afforded **3ab** (22.1 mg, 73% yield, >99% e.e., >20:1 d.r., >20:1 r.r.) as a colorless solid. Diastereomer and regiomer ratios (11:1 d.r., >20:1 r.r.) were determined from the ^{1}H NMR spectrum of crude material.

R*f* = 0.4 (Hexane/EtOAc = 9/1).

M.P. 116–117 °C (Hexane/EtOAc).

^{1}H NMR (500 MHz, $CDCl_3$, 298 K): δ 8.18 (d, *J* = 8.6 Hz, 1H, H-18), 8.13 (d, *J* = 8.4 Hz, 1H, H-13), 7.86 (d, *J* = 8.1 Hz, 1H, H-16), 7.78–7.74 (m, 1H, H-14), 7.60–7.55 (m, 1H, H-15), 7.44–7.38 (m, 3H, H-8 and H-19), 7.34–7.30 (m, 2H, H-9), 7.28–7.23 (m, 1H, H-10), 6.06 (s, 1H, OH), 2.96 (dd, *J* = 11.8, 2.9 Hz, 1H, H-3), 1.87–1.78 (m, 1H, H-4), 1.31 (s, 3H, H-1), 1.08–1.00 (m, 1H, H-4), 1.00–0.92 (m, 1H, H-5), 0.90–0.80 (m, 1H, H-5), 0.62 (t, *J* = 7.2 Hz, 3H, H-6) ppm.

^{13}C NMR (125 MHz, $CDCl_3$, 298 K): δ 165.2 (C-11), 145.7 (C-12), 141.7 (C-7), 137.4 (C-18), 130.1 (C-8), 130.0 (C-14), 129.0 (C-13), 128.1 (C-9), 127.6 (C-16), 127.2 (C-17), 126.6 (C-15), 126.6 (C-10), 117.6 (C-19), 76.3 (C-2), 56.2 (C-3), 32.2 (C-4), 28.1 (C-1), 20.8 (C-5), 14.0 (C-6) ppm.

HRMS (ESI) m/z: $[M–H_2O+H]^+$ calcd for $C_{21}H_{22}N^+$ 288.1747, found 288.1758.

IR (thin film): ṽ 3317 (s), 1599 (s), 1504 (s), 1452 (s), 1393 (s), 1160 (s), 1061 (s) cm^{-1}.

Specific rotation: $[\alpha]_D^{22}$ = −27.4 (*c* 0.5, CH_2Cl_2).

The **enantiomeric ratio** of **3ab** was determined by SFC analysis (CHIRALPACK ID (25 cm), column temperature 25 °C, solvent CO_2/MeOH = 80/20, flow rate = 2.0 mL/min): t_R = 3.9 min (major), t_R = 4.2 min (minor).

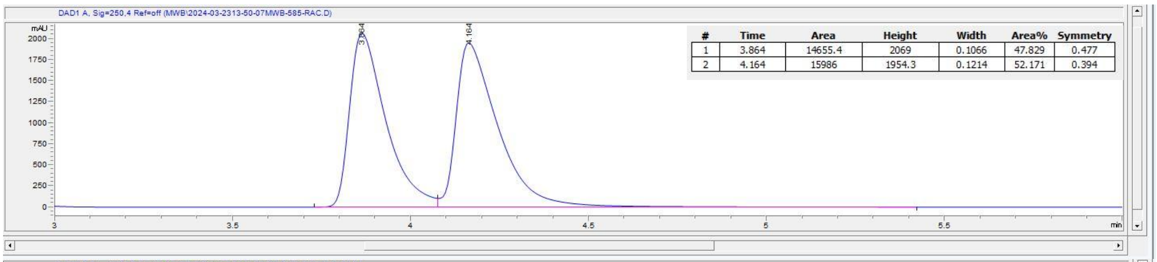

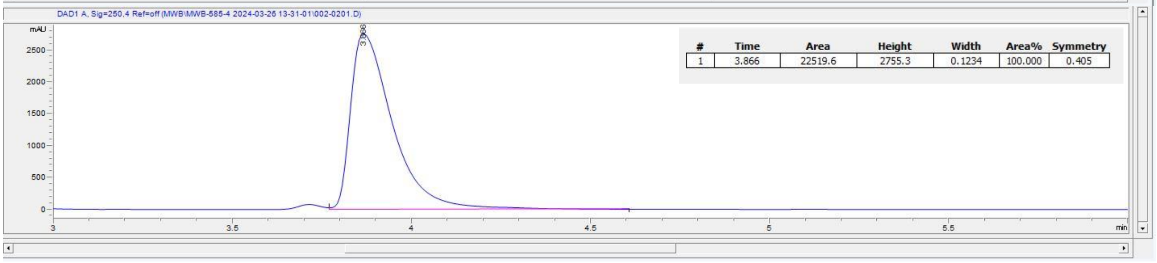

Characteristic signals for diastereomer **3ab'**

^{1}H NMR (500 MHz, $CDCl_3$, 298 K): 3.09 (dd, *J* = 11.8, 3.2 Hz, 1H), 1.71 (s, 3H).

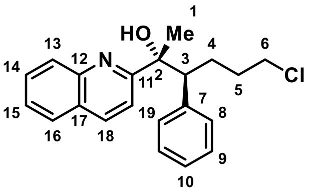

$C_{21}H_{22}ClNO$
M = 339.86

(2*R*,3*S*)-6-Chloro-3-phenyl-2-(quinolin-2-yl)hexan-2-ol (**3ac**, **GP 6**) was prepared from 1-(quinolin-2-yl)ethan-1-ol (**1a**, 17.3 mg, 0.10 mmol, 100 mol%) and (*E*)-(4-chlorobut-1-en-1-yl)benzene (**2c**, 41.7 mg, 0.25 mmol, 250 mol%), using $[Ir(cod)_2]$BARF (6.36 mg, 5.0 μmol, 5.0 mol%), and **L5** ((*R*)-DM-SEGPHOS, 3.61 mg, 5.0 μmol, 5.0 mol%) in DCB (0.2 mL) at 110 °C for 36 h. Purification by flash column chromatography on silica gel (hexane/ethyl acetate from 100/1 to 19/1) afforded **3ac** (24.3 mg, 72% yield, 98% e.e., >20:1 d.r., >20:1 r.r.) as a colorless solid. Diastereomer and regiomer ratios (6:1 d.r., >20:1 r.r.) were determined from the ^{1}H NMR spectrum of crude material.

R*f* = 0.3 (Hexane/EtOAc = 9/1).

M.P. 113–114 °C (Hexane/EtOAc).

^{1}H NMR (500 MHz, $CDCl_3$, 298 K): δ 8.20 (d, *J* = 8.7 Hz, 1H, H-18), 8.13 (d, *J* = 8.4 Hz, 1H, H-13), 7.86 (d, *J* = 8.1 Hz, 1H, H-16), 7.79–7.74 (m, 1H, H-14), 7.61–7.56 (m, 1H, H-15), 7.43 (d, *J* = 8.7 Hz, 1H, H-19), 7.43–7.39 (m, 2H, H-8), 7.37–7.32 (m, 2H, H-9), 7.30–7.26 (m, 1H, H-10), 6.10 (s, 1H, OH), 3.26 (t, *J* = 6.8 Hz, 2H, H-6), 2.99 (dd, *J* = 11.9, 3.2 Hz, 1H, H-3),

2.01–1.91 (m, 1H, H-4), 1.49–1.36 (m, 2H, H-5), 1.33 (s, 3H, H-1), 1.31–1.23 (m, 1H, H-4) ppm.

^{13}C NMR (125 MHz, $CDCl_3$, 298 K): δ 164.7 (C-11), 145.7 (C-12), 140.8 (C-7), 137.5 (C-18), 130.1 (C-8), 130.0 (C-14), 129.0 (C-13), 128.3 (C-9), 127.6 (C-16), 127.2 (C-17), 126.9 (C-15), 126.7 (C-10), 117.5 (C-19), 76.2 (C-2), 55.7 (C-3), 44.9 (C-6), 30.8 (C-5), 28.0 (C-1), 27.3 (C-4) ppm.

HRMS (ESI) m/z: $[M–H_2O–HCl+H]^+$ calcd for $C_{21}H_{20}N^+$ 286.1590, found 286.1595.

IR (thin film): ṽ 3355 (br), 2947 (s), 2863 (s), 1599 (s), 1504 (s), 1385 (s), 1141 (s), 1076 (s) cm^{-1}.

Specific rotation: $[\alpha]_D^{25}$ = −28.2 (*c* 1, CH_2Cl_2).

The **enantiomeric ratio** of **3ac** was determined by SFC analysis (CHIRALPACK SC (25 cm), column temperature 25 °C, solvent CO_2/MeOH = 90/10, flow rate = 2.0 mL/min): t_R = 5.6 min (major), t_R = 6.0 min (minor).

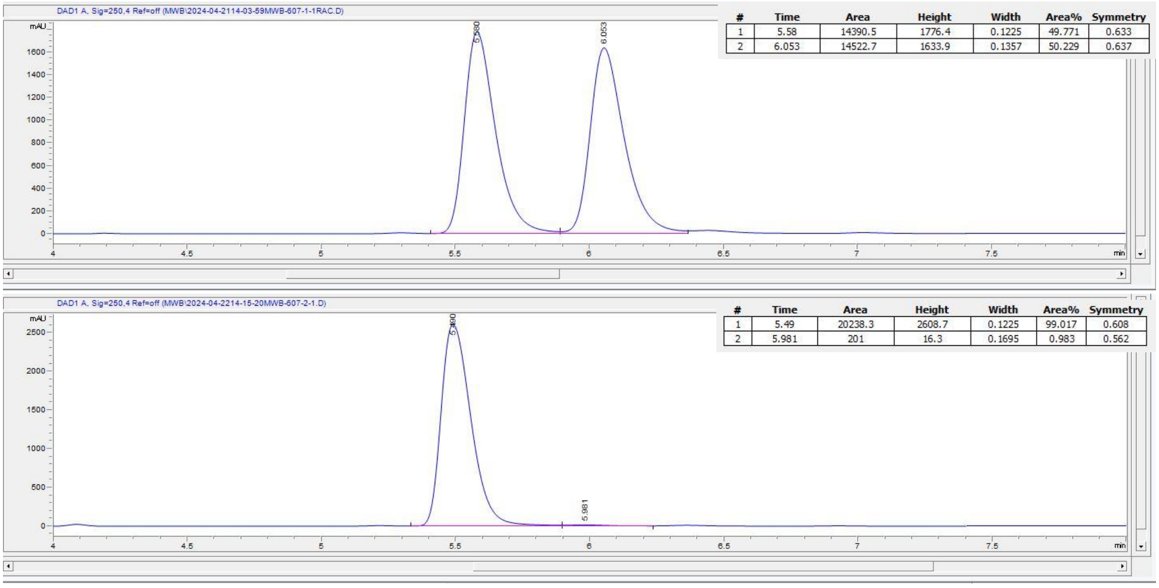

Characteristic signals for diastereomer **3ac'**

^{1}H NMR (500 MHz, $CDCl_3$, 298 K): 3.51 (t, *J* = 6.6 Hz, 2H), 3.12 (dd, *J* = 11.9, 3.4 Hz, 1H), 1.75 (s, 3H).

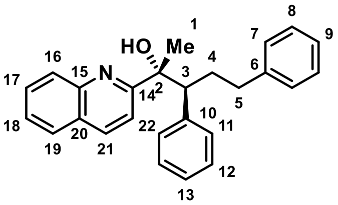

$C_{26}H_{25}NO$
M = 367.49

(2*R*,3*S*)-3,5-Diphenyl-2-(quinolin-2-yl)pentan-2-ol (**3ad**, **GP 6**) was prepared from 1-(quinolin-2-yl)ethan-1-ol (**1a**, 17.3 mg, 0.10 mmol, 100 mol%) and (*E*)-prop-1-ene-1,3-diyldibenzene (**2d**, 48.6 mg, 0.25 mmol, 250 mol%), using [Ir(cod)$_2$]BARF (6.36 mg, 5.0 µmol, 5.0 mol%), and **L5** ((*R*)-DM-SEGPHOS, 3.61 mg, 5.0 µmol, 5.0 mol%) in DCB (0.2 mL) at 110 °C for 36 h. Purification by flash column chromatography on silica gel (hexane/ethyl acetate from 100/1 to 19/1) afforded **3ad** (24.3 mg, 65% yield, 99% e.e., >20:1 d.r., >20:1 r.r.) as a colorless solid. Diastereomer and regiomer ratios (5:1 d.r., >20:1 r.r.) were determined from the ^{1}H NMR spectrum of crude material.

R*f* = 0.5 (Hexane/EtOAc = 9/1).

M.P. 147–148 °C (Hexane/EtOAc).

^{1}H NMR (500 MHz, $CDCl_3$, 298 K): δ 8.12 (d, *J* = 8.7 Hz, 1H, H-21), 8.10 (d, *J* = 8.4 Hz, 1H, H-16), 7.86 (d, *J* = 8.2 Hz, 1H, H-19), 7.78–7.74 (m, 1H, H-17), 7.61–7.56 (m, 1H, H-18), 7.51–7.44 (m, 2H, H-11), 7.42–7.36 (m, 2H, H-12), 7.35–7.29 (m, 1H, H-13), 7.24 (d, *J* = 8.7 Hz, 1H, H-22), 7.17–7.09 (m, 3H, H-8 and H-9), 6.85–6.81 (m, 2H, H-7), 6.12 (s, 1H, OH), 2.96 (dd, *J* = 11.3, 2.8 Hz, 1H, H-3), 2.42–2.34 (m, 1H, H-5), 2.25–2.16 (m, 1H, H-4), 2.16–2.08 (m, 1H, H-5), 1.51–1.43 (m, 1H, H-4), 1.29 (s, 3H, H-1) ppm.

^{13}C NMR (125 MHz, $CDCl_3$, 298 K): δ 164.9 (C-14), 145.7 (C-15), 142.2 (C-6), 141.2 (C-10), 137.4 (C-21), 130.3 (C-11), 130.1 (C-17), 129.0 (C-16), 128.5 (C-7), 128.2 (C-12), 128.2 (C-8), 127.6 (C-19), 127.2 (C-20), 126.8 (C-18), 126.7 (C-13), 125.6 (C-9), 117.5 (C-22), 76.3 (C-2), 55.2 (C-3), 33.5 (C-5), 31.6 (C-4), 28.2 (C-1) ppm.

HRMS (ESI) m/z: $[M–H_2O+H]^+$ calcd for $C_{26}H_{24}N^+$ 350.1903, found 350.1914.

IR (thin film): ṽ 3319 (s), 1600 (s), 1493 (s), 1391 (s), 1148 (m), 1080 (s) cm^{-1}.

Specific rotation: $[\alpha]_D^{25}$ = −48.3 (*c* 0.5, CH_2Cl_2).

The **enantiomeric ratio** of **3ad** was determined by SFC analysis (CHIRALPACK SC (25 cm), column temperature 25 °C, solvent CO_2/MeOH = 90/10, flow rate = 2.0 mL/min): t_R = 7.1 min (major), t_R = 7.8 min (minor).

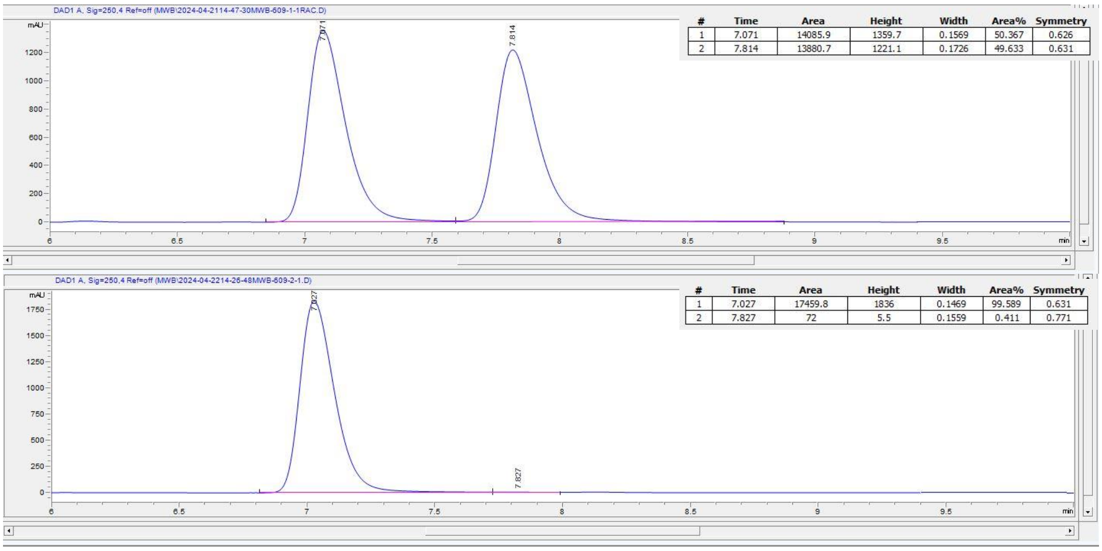

Characteristic signals for diastereomer **3ad'**

^{1}H NMR (500 MHz, $CDCl_3$, 298 K): 3.17 (dd, *J* = 11.7, 2.36 Hz, 1H), 1.74 (s, 3H).

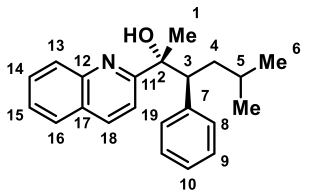

$C_{22}H_{25}NO$
M = 319.45

(2*R*,3*S*)-5-Methyl-3-phenyl-2-(quinolin-2-yl)hexan-2-ol (**3ae**, **GP 6**) was prepared from 1-(quinolin-2-yl)ethan-1-ol (**1a**, 17.3 mg, 0.10 mmol, 100 mol%) and (*E*)-(3-methylbut-1-en-1-yl)benzene (**2e**, 36.6 mg, 0.25 mmol, 250 mol%), using [Ir(cod)$_2$]BARF (6.36 mg, 5.0 µmol, 5.0 mol%), and **L5** ((*R*)-DM-SEGPHOS, 3.61 mg, 5.0 µmol, 5.0 mol%) in H_2O (0.2 mL) at 110 °C for 3 d. Purification by flash column chromatography on silica gel (hexane/ethyl acetate from 100/1 to 19/1) afforded **3ae** (22.5 mg, 71% yield, >99% e.e., >20:1 d.r., >20:1 r.r.) as a colorless solid. Diastereomer and regiomer ratios (>20:1 d.r., >20:1 r.r.) were determined from the ^{1}H NMR spectrum of crude material.

R*$_f$* = 0.4 (Hexane/EtOAc = 9/1).

M.**P**. 98–99 °C (Hexane/EtOAc).

^{1}H NMR (500 MHz, $CDCl_3$, 298 K): δ 8.19 (d, *J* = 8.6 Hz, 1H, H-18), 8.14 (d, *J* = 8.4 Hz, 1H, H-13), 7.86 (d, *J* = 8.1 Hz, 1H, H-16), 7.79–7.74 (m, 1H, H-14), 7.60–7.56 (m, 1H, H-15), 7.47–7.40 (m, 3H, H-8 and H-19), 7.36–7.31 (m, 2H, H-9), 7.29–7.24 (m, 1H, H-10), 6.06 (s, 1H, OH), 3.09 (dd, *J* = 12.1, 3.0 Hz, 1H, H-3), 1.95–1.88 (m, 1H, H-4), 1.31 (s, 3H, H-1), 1.15–1.05

(m, 1H, H-5), 0.77–0.69 (m, 1H, H-4), 0.63 (d, J = 6.7 Hz, 3H, H-6), 0.62 (d, J = 6.7 Hz, 3H, H-6) ppm.

^{13}C NMR (125 MHz, $CDCl_3$, 298 K): δ 165.2 (C-11), 145.7 (C-12), 141.6 (C-7), 137.4 (C-18), 130.1 (C-8), 130.0 (C-14), 129.0 (C-13), 128.1 (C-9), 127.6 (C-16), 127.2 (C-17), 126.6 (C-15), 126.6 (C-10), 117.5 (C-19), 76.4 (C-2), 54.0 (C-3), 39.2 (C-4), 28.3 (C-1), 25.2 (C-5), 24.2 (C-6), 21.0 (C-6) ppm.

HRMS (ESI) m/z: $[M+Na]^+$ calcd for $C_{22}H_{25}NNaO^+$ 342.1828, found 342.1842.

IR (thin film): ṽ 3332 (s), 2956 (s), 2927 (s), 1601 (s), 1452 (s), 1386 (s), 1153 (s), 1075 (s) cm^{-1}.

Specific rotation: $[\alpha]_D^{24}$ = −9.0 (c 1, CH_2Cl_2).

The **enantiomeric ratio** of **3ae** was determined by SFC analysis (CHIRALPACK OD-H (25 cm), column temperature 25 °C, solvent CO_2/MeOH = 90/10, flow rate = 2.0 mL/min): t_R = 5.3 min (minor), t_R = 5.8 min (major).

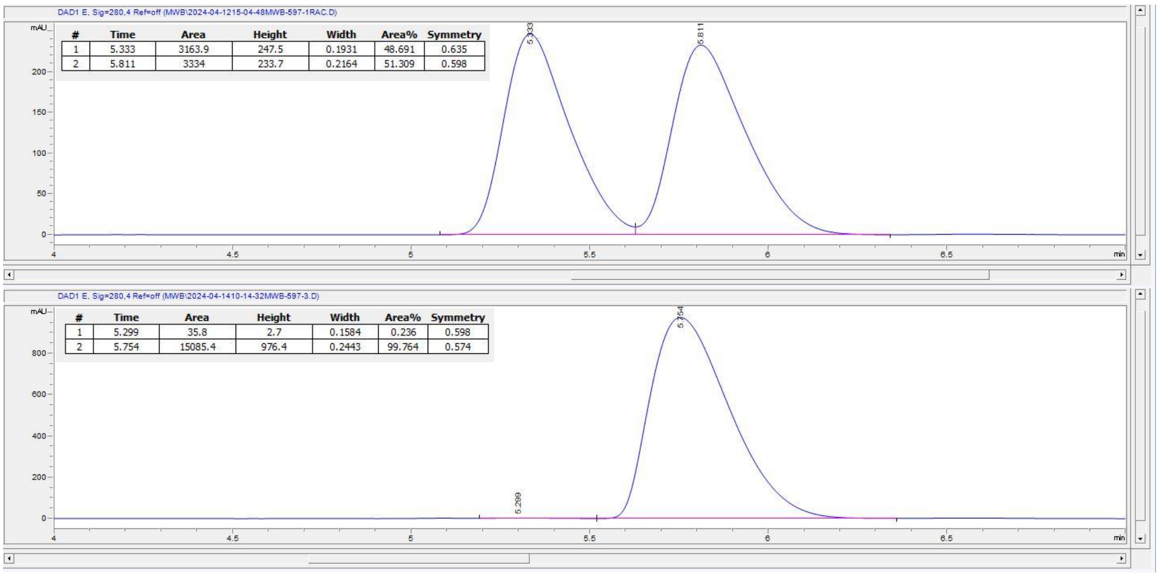

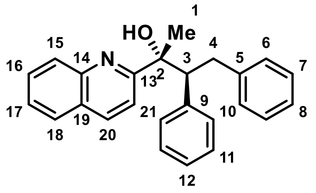

$C_{25}H_{23}NO$
M = 353.47

(2*R*,3*S*)-3,4-Diphenyl-2-(quinolin-2-yl)butan-2-ol (**3af**, **GP 6**) was prepared from 1-(quinolin-2-yl)ethan-1-ol (**1a**, 17.3 mg, 0.10 mmol, 100 mol%) and (E)-stilbene (**2f**, 90.1 mg, 0.50 mmol,

250 mol%), using [Ir(cod)$_2$]BARF (12.7 mg, 10 μmol, 10 mol%), and **L5** ((*R*)-DM-SEGPHOS, 7.23 mg, 10 μmol, 10 mol%) in DCB (0.2 mL) at 110 °C for 3 d. Purification by flash column chromatography on silica gel (hexane/ethyl acetate from 100/1 to 19/1) afforded **3af** (23.5 mg, 66% yield, 98% e.e., >20:1 d.r.) as a colorless solid. Diastereomer ratios (13:1 d.r.) were determined from the ^{1}H NMR spectrum of crude material.

R*f* = 0.3 (Hexane/EtOAc = 9/1).

M.P. 133–134 °C (Hexane/EtOAc).

^{1}H NMR (500 MHz, CDCl$_3$, 298 K): δ 8.20 (d, *J* = 8.6 Hz, 1H, H-20), 8.16 (d, *J* = 8.4 Hz, 1H, H-15), 7.87 (d, *J* = 8.2 Hz, 1H, H-18), 7.80–7.76 (m, 1H, H-16), 7.62–7.57 (m, 1H, H-17), 7.49 (d, *J* = 8.6 Hz, 1H, H-21), 7.44–7.32 (m, 2H, H-10), 7.30–7.24 (m, 2H, H-11), 7.24–7.19 (m, 1H, H-12), 6.97–6.91 (m, 3H, H-7 and H-8), 6.66–6.61 (m, 2H, H-6), 6.28 (s, 1H, OH), 3.26 (dd, *J* = 10.8, 3.3 Hz, 1H, H-3), 3.03 (dd, *J* = 13.9, 10.8 Hz, 1H, H-4), 2.56 (dd, *J* = 13.9, 3.3 Hz, 1H, H-4), 1.36 (s, 3H, H-1) ppm.

^{13}C NMR (125 MHz, CDCl$_3$, 298 K): δ 164.9 (C-13), 145.8 (C-14), 141.1 (C-5 or C-9), 141.0 (C-5 or C-9), 137.6 (C-20), 130.3 (C-10), 130.1 (C-16), 129.0 (C-6), 128.9 (C-15), 128.0 (C-11), 127.8 (C-7), 127.6 (C-18), 127.3 (C-19), 126.8 (C-17), 126.7 (C-12), 125.4 (C-8), 117.5 (C-21), 76.5 (C-2), 58.9 (C-3), 37.3 (C-4), 28.2 (C-1) ppm.

HRMS (ESI) m/z: [M–H$_2$O+H]$^+$ calcd for $C_{25}H_{22}N^+$ 336.1747, found 336.1759.

IR (thin film): ṽ 3341 (s), 1599 (s), 1492 (s), 1382 (s), 1156 (s), 1070 (s) cm^{-1}.

Specific rotation: $[\alpha]_D^{25}$ = −58.3 (*c* 0.5, CH_2Cl_2).

The **enantiomeric ratio** of **3af** was determined by SFC analysis (CHIRALPACK OD-H (25 cm), column temperature 25 °C, solvent CO_2/MeOH = 90/10, flow rate = 2.0 mL/min): t_R = 9.3 min (minor), t_R = 10.9 min (major).

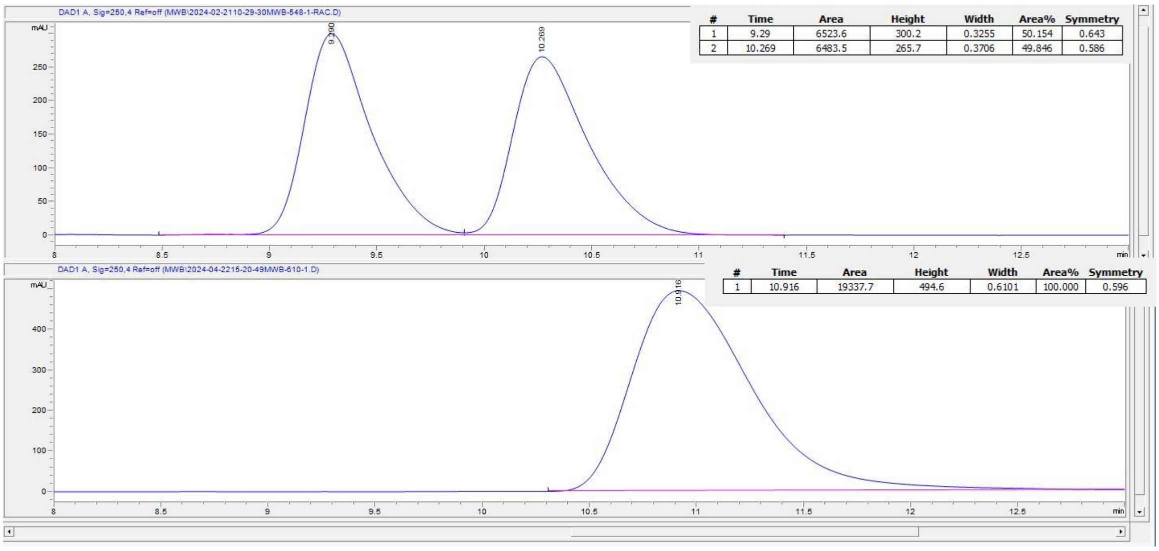

Characteristic signals for diastereomer **3af'**

^{1}H NMR (500 MHz, $CDCl_3$, 298 K): 3.69 (dd, *J* = 13.9, 3.2 Hz, 1H), 3.48 (dd, *J* = 11.3, 3.2 Hz, 1H).

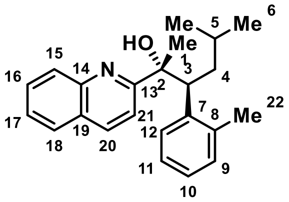

$C_{23}H_{27}NO$
M = 333.48

(2*R*,3*S*)-5-Methyl-2-(quinolin-2-yl)-3-(*o*-tolyl)hexan-2-ol (**3ag**, **GP 6**) was prepared from 1-(quinolin-2-yl)ethan-1-ol (**1a**, 17.3 mg, 0.10 mmol, 100 mol%) and (*E*)-1-methyl-2-(3-methylbut-1-en-1-yl)benzene (**2g**, 40.1 mg, 0.25 mmol, 250 mol%), using [Ir(cod)$_2$]BARF (12.7 mg, 10 µmol, 10 mol%), and **L5** ((*R*)-DM-SEGPHOS, 7.23 mg, 10 µmol, 10 mol%) in H_2O (0.2 mL) at 110 °C for 3 d. Then, *i*-PrOH (19.1 µL, 15.0 mg, 0.25 mmol, 250 mol%) was added, and the reaction mixture was stirred at 110 °C for 2 d. Purification by flash column chromatography on silica gel (hexane/ethyl acetate from 100/1 to 19/1) afforded **3ag** (27.0 mg, 81% yield, 95% e.e., >20:1 d.r., >20:1 r.r.) as a colorless solid. Diastereomer and regiomer ratios (17:1 d.r., >20:1 r.r.) were determined from the ^{1}H NMR spectrum of crude material.

R*f* = 0.5 (Hexane/EtOAc = 9/1).

M.P. 77–78 °C (Hexane/EtOAc).

^{1}H NMR (500 MHz, $CDCl_3$, 298 K): δ 8.16 (d, *J* = 8.6 Hz, 1H, H-20), 8.13 (d, *J* = 8.5 Hz, 1H, H-15), 7.86 (d, *J* = 8.1 Hz, 1H, H-18), 7.78–7.74 (m, 1H, H-16), 7.67 (d, *J* = 7.9 Hz, 1H, H-12), 7.60–7.55 (m, 1H, H-17), 7.33 (d, *J* = 8.6 Hz, 1H, H-21), 7.28–7.23 (m, 1H, H-11), 7.17–7.11

(m, 2H, H-9 and H-10), 6.11 (s, 1H, OH), 3.47 (dd, J = 11.8, 3.0 Hz, 1H, H-3), 2.31 (s, 3H, H-22), 1.96–1.89 (m, 1H, H-4), 1.33 (s, 3H, H-1), 1.13–1.03 (m, 1H, H-5), 0.97–0.89 (m, 1H, H-4), 0.64 (d, J = 6.7 Hz, 3H, H-6), 0.63 (d, J = 6.7 Hz, 3H, H-6) ppm.

^{13}C NMR (125 MHz, $CDCl_3$, 298 K): δ 165.3 (C-13), 145.7 (C-14), 140.3 (C-7), 137.3 (C-8), 137.2 (C-20), 130.0 (C-9 or C-16), 130.0 (C-9 or C-16), 129.1 (C-15), 128.8 (C-12), 127.6 (C-18), 127.2 (C-19), 126.6 (C-17), 126.3 (C-11), 126.2 (C-10), 117.6 (C-21), 76.8 (C-2), 47.4 (C-3), 40.0 (C-4), 26.9 (C-1), 25.4 (C-5), 24.3 (C-6), 21.7 (C-6), 20.6 (C-22) ppm.

HRMS (ESI) m/z: $[M+H]^+$ calcd for $C_{23}H_{28}NO^+$ 334.2165, found 334.2175.

IR (thin film): ṽ 3335 (s), 2955 (s), 2928 (s), 1602 (s), 1505 (s), 1457 (s), 1388 (s), 1153 (s), 1075 (s) cm^{-1}.

Specific rotation: $[\alpha]_D^{28}$ = −18.9 (c 1, CH_2Cl_2).

The **enantiomeric ratio** of **3ag** was determined by SFC analysis (CHIRALPACK IE (25 cm), column temperature 25 °C, solvent CO_2/MeOH = 90/10, flow rate = 2.0 mL/min): t_R = 6.4 min (major), t_R = 7.2 min (minor).

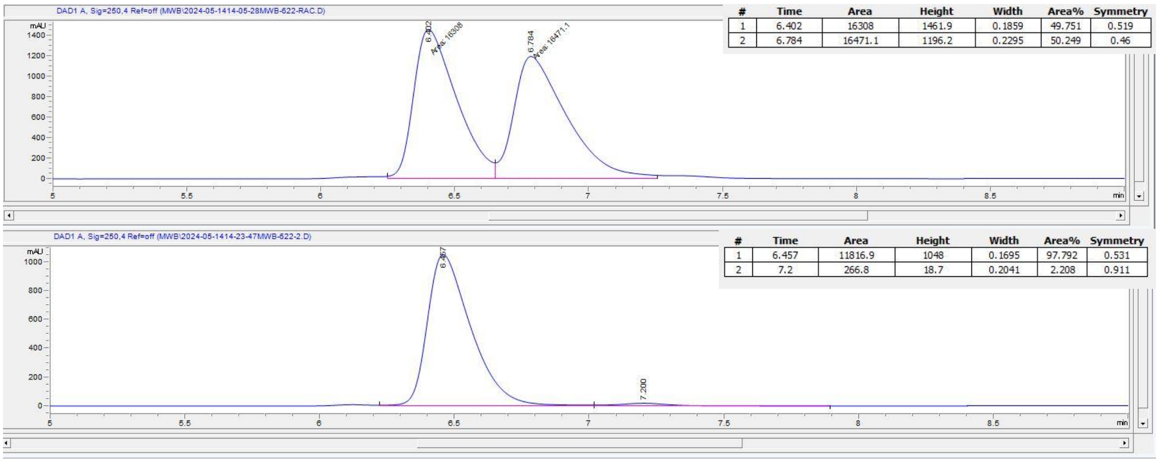

Characteristic signals for diastereomer **3ag'**

^{1}H NMR (500 MHz, $CDCl_3$, 298 K): 1.69 (s, 3H), 0.41 (d, J = 6.5 Hz, 3H), 0.37 (d, J = 6.5 Hz, 3H).

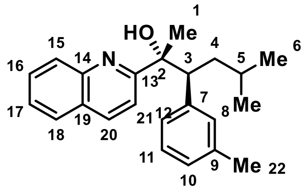

$C_{23}H_{27}NO$
M = 333.48

(2*R*,3*S*)-5-Methyl-2-(quinolin-2-yl)-3-(*m*-tolyl)hexan-2-ol (**3ah**, **GP 6**) was prepared from 1-(quinolin-2-yl)ethan-1-ol (**1a**, 17.3 mg, 0.10 mmol, 100 mol%) and (*E*)-1-methyl-3-(3-methylbut-1-en-1-yl)benzene (**2h**, 40.1 mg, 0.25 mmol, 250 mol%), using [Ir(cod)$_2$]BARF (12.7 mg, 10 µmol, 10 mol%), and **L5** ((*R*)-DM-SEGPHOS, 7.23 mg, 10 µmol, 10 mol%) in H_2O (0.2 mL) at 110 °C for 3 d. Then, *i*-PrOH (19.1 µL, 15.0 mg, 0.25 mmol, 250 mol%) was added, and the reaction mixture was stirred at 110 °C for 2 d. Purification by flash column chromatography on silica gel (hexane/ethyl acetate from 100/1 to 19/1) afforded **3ah** (30.6 mg, 92% yield, 96% e.e., >20:1 d.r., >20:1 r.r.) as a colorless solid. Diastereomer and regiomer ratios (15:1 d.r., >20:1 r.r.) were determined from the ^{1}H NMR spectrum of crude material.

R*f* = 0.5 (Hexane/EtOAc = 9/1).

M.P. 84–85 °C (Hexane/EtOAc).

^{1}H NMR (500 MHz, $CDCl_3$, 298 K): δ 8.19 (d, *J* = 8.6 Hz, 1H, H-20), 8.13 (d, *J* = 8.5 Hz, 1H, H-15), 7.86 (d, *J* = 8.1 Hz, 1H, H-18), 7.79–7.73 (m, 1H, H-16), 7.60–7.55 (m, 1H, H-17), 7.44 (d, *J* = 8.6 Hz, 1H, H-21), 7.25–7.19 (m, 3H, H-8, H-11 and H-12), 7.10–7.05 (m, 1H, H-10), 6.03 (s, 1H, OH), 3.04 (dd, *J* = 11.7, 3.0 Hz, 1H, H-3), 2.37 (s, 3H, H-22), 1.93–1.84 (m, 1H, H-4), 1.31 (s, 3H, H-1), 1.14–1.04 (m, 1H, H-5), 0.75–0.67 (m, 1H, H-4), 0.63 (d, *J* = 6.7 Hz, 3H, H-6), 0.61 (d, *J* = 6.7 Hz, 3H, H-6) ppm.

^{13}C NMR (125 MHz, $CDCl_3$, 298 K): δ 165.3 (C-13), 145.7 (C-14), 141.5 (C-7), 137.4 (C-9), 137.3 (C-20), 130.9 (C-8), 129.9 (C-16), 129.1 (C-15), 127.9 (Ar), 127.6 (C-18), 127.3 (Ar), 127.2 (C-19), 126.6 (C-17), 117.6 (C-21), 76.4 (C-2), 53.9 (C-3), 39.2 (C-4), 28.3 (C-1), 25.2 (C-5), 24.2 (C-6), 21.8 (C-22), 21.0 (C-6) ppm.

HRMS (ESI) m/z: $[M+H]^+$ calcd for $C_{23}H_{28}NO^+$ 334.2165, found 334.2175.

IR (thin film): ṽ 3327 (s), 2966 (s), 2927 (s), 1600 (s), 1504 (s), 1462 (s), 1386 (s), 1151 (s), 1076 (s) cm^{-1}.

Specific rotation: $[\alpha]_D^{29}$ = −11.6 (*c* 1, CH_2Cl_2).

The **enantiomeric ratio** of **3ah** was determined by SFC analysis (CHIRALPACK SC (25 cm), column temperature 25 °C, solvent CO_2/MeOH = 90/10, flow rate = 2.0 mL/min): t_R = 3.9 min (major), t_R = 4.2 min (minor).

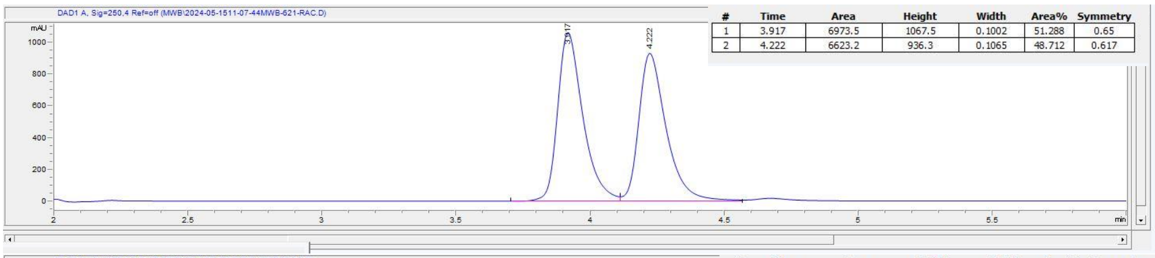

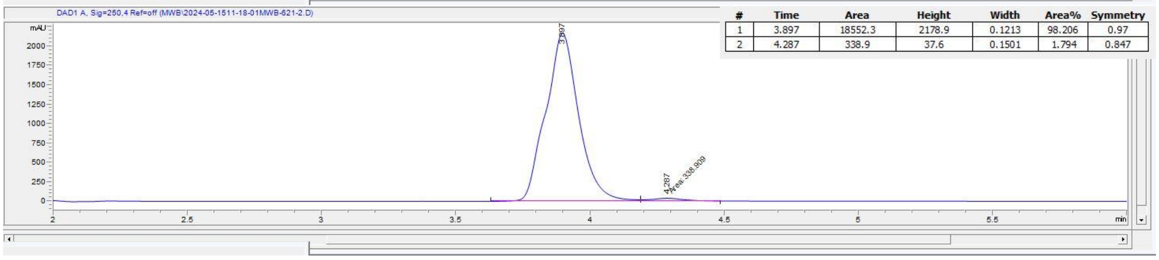

Characteristic signals for diastereomer **3ah'**

^{1}H NMR (500 MHz, $CDCl_3$, 298 K): 3.12 (dd, *J* = 12.1, 3.4 Hz, 1H), 2.11 (s, 3H).

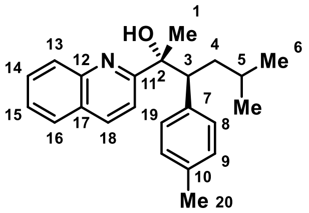

$C_{23}H_{27}NO$
M = 333.48

(2*R*,3*S*)-5-Methyl-2-(quinolin-2-yl)-3-(*p*-tolyl)hexan-2-ol (**3ai**, **GP 6**) was prepared from 1-(quinolin-2-yl)ethan-1-ol (**1a**, 17.3 mg, 0.10 mmol, 100 mol%) and (*E*)-1-methyl-4-(3-methylbut-1-en-1-yl)benzene (**2i**, 40.1 mg, 0.25 mmol, 250 mol%), using [Ir(cod)$_2$]BARF (12.7 mg, 10 μmol, 10 mol%), and **L5** ((*R*)-DM-SEGPHOS, 7.23 mg, 10 μmol, 10 mol%) in H_2O (0.2 mL) at 110 °C for 3 d. Then, *i*-PrOH (19.1 μL, 15.0 mg, 0.25 mmol, 250 mol%) was added, and the reaction mixture was stirred at 110 °C for 2 d. Purification by flash column chromatography on silica gel (hexane/acetone from 100/1 to 19/1) afforded **3ai** (27.0 mg, 81% yield, 99% e.e., >20:1 d.r., >20:1 r.r.) as a colorless solid. Diastereomer and regiomer ratios (14:1 d.r., >20:1 r.r.) were determined from the ^{1}H NMR spectrum of crude material.

R*f* = 0.6 (Hexane/EtOAc = 9/1).

M.P. 114–115 °C (Hexane/Acetone).

^{1}H NMR (500 MHz, $CDCl_3$, 298 K): δ 8.18 (d, *J* = 8.6 Hz, 1H, H-18), 8.13 (d, *J* = 8.4 Hz, 1H, H-13), 7.86 (d, *J* = 8.2 Hz, 1H, H-16), 7.78–7.73 (m, 1H, H-14), 7.60–7.55 (m, 1H, H-15), 7.43 (d, *J* = 8.6 Hz, 1H, H-19), 7.30 (d, *J* = 7.5 Hz, 2H, H-8), 7.14 (d, *J* = 7.5 Hz, 2H, H-9), 6.00 (s, 1H, OH), 3.04 (dd, *J* = 12.1, 3.0 Hz, 1H, H-3), 2.36 (s, 3H, H-20), 1.91–1.83 (m, 1H, H-4), 1.29

(s, 3H, H-1), 1.14–1.04 (m, 1H, H-5), 0.73–0.66 (m, 1H, H-4), 0.62 (d, *J* = 6.7 Hz, 3H, H-6), 0.60 (d, *J* = 6.7 Hz, 3H, H-6) ppm.

^{13}C NMR (125 MHz, $CDCl_3$, 298 K): δ 165.3 (C-11), 145.8 (C-12), 138.4 (C-7), 137.3 (C-18), 136.0 (C-10), 130.0 (C-14 or C-8), 129.9 (C-14 or C-8), 129.1 (C-13), 128.8 (C-9), 127.6 (C-16), 127.2 (C-17), 126.6 (C-15), 117.6 (C-19), 76.5 (C-2), 53.5 (C-3), 39.2 (C-4), 28.3 (C-1), 25.2 (C-5), 24.2 (C-6), 21.3 (C-6 or C-20), 21.0 (C-6 or C-20) ppm.

HRMS (ESI) m/z: $[M+H]^+$ calcd for $C_{23}H_{28}NO^+$ 334.2165, found 334.2176.

IR (thin film): ṽ 3355 (s), 2951 (s), 2928 (s), 1601(s), 1505 (s), 1383 (s), 1122 (s), 1077 (s) cm^{-1}.

Specific rotation: $[\alpha]_D^{30}$ = −10.7 (*c* 1, CH_2Cl_2).

The **enantiomeric ratio** of **3ai** was determined by SFC analysis (CHIRALPACK IE (25 cm), column temperature 25 °C, solvent CO_2/MeOH = 90/10, flow rate = 2.0 mL/min): t_R = 6.6 min (major), t_R = 7.3 min (minor).

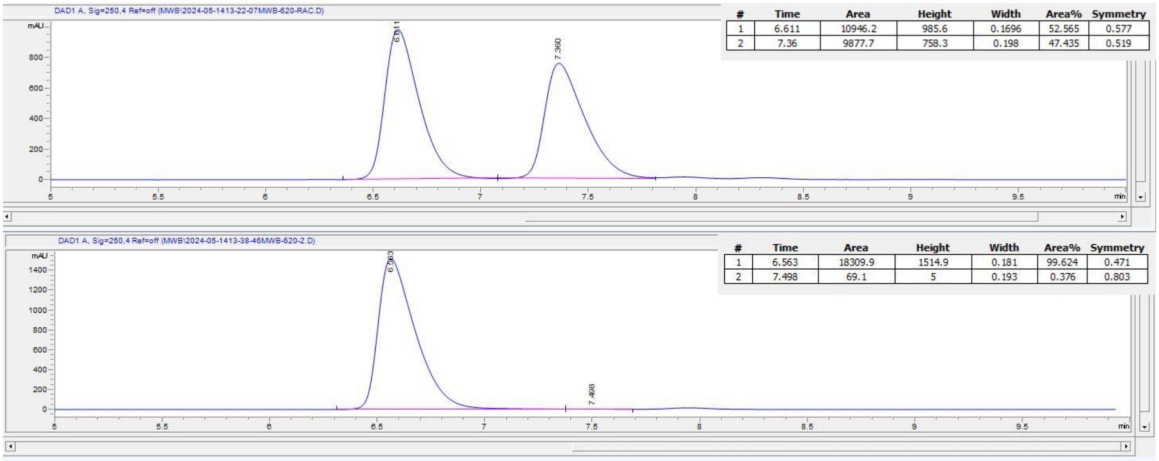

#	Time	Area	Height	Width	Area%	Symmetry
1	6.611	10946.2	985.6	0.1696	52.565	0.577
2	7.36	9877.7	758.3	0.198	47.435	0.519

#	Time	Area	Height	Width	Area%	Symmetry
1	6.563	18309.9	1514.9	0.181	99.624	0.471
2	7.498	69.1	5	0.193	0.376	0.803

Characteristic signals for diastereomer **3ai'**

^{1}H NMR (500 MHz, $CDCl_3$, 298 K): 3.18 (dd, *J* = 12.1, 3.4 Hz, 1H), 0.41 (d, *J* = 6.5 Hz, 3H), 0.39 (d, *J* = 6.5 Hz, 3H).

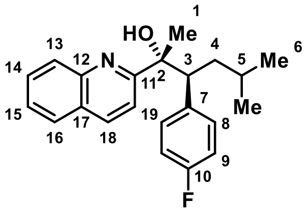

$C_{22}H_{24}FNO$
M = 337.44

(2*R*,3*S*)-3-(4-Fluorophenyl)-5-methyl-2-(quinolin-2-yl)hexan-2-ol (**3aj**, **GP 6**) was prepared from 1-(quinolin-2-yl)ethan-1-ol (**1a**, 17.3 mg, 0.10 mmol, 100 mol%) and (*E*)-1-fluoro-4-(3-methylbut-1-en-1-yl)benzene (**2j**, 41.1 mg, 0.25 mmol, 250 mol%), using $[Ir(cod)_2]BARF$ (12.7 mg, 10 μmol, 10 mol%), and **L5** ((*R*)-DM-SEGPHOS, 7.23 mg, 10 μmol, 10 mol%) in DCB (0.2 mL) at 110 °C for 3 d. Then, *i*-PrOH (19.1 μL, 15.0 mg, 0.25 mmol, 250 mol%) was added, and the reaction mixture was stirred at 110 °C for 2 d. Purification by flash column chromatography on silica gel (hexane/ethyl acetate from 100/1 to 19/1) afforded **3aj** (28.4 mg, 84% yield, 98% ee, >20:1 d.r., >20:1 r.r.) as a colorless solid. Diastereomer and regiomer ratios (>20:1 d.r., >20:1 r.r.) were determined from the ^1H NMR spectrum of crude material.

R*f* = 0.5 (Hexane/EtOAc = 15/1).

M.P. 82–83 °C (Hexane/EtOAc).

^{1}H NMR (500 MHz, $CDCl_3$, 298 K): δ 8.20 (d, *J* = 8.6 Hz, 1H, H-18), 8.13 (d, *J* = 8.4 Hz, 1H, H-13), 7.87 (d, *J* = 8.0 Hz, 1H, H-16), 7.79–7.74 (m, 1H, H-14), 7.60–7.56 (m, 1H, H-15), 7.44 (d, *J* = 8.6 Hz, 1H, H-19), 7.42–7.36 (m, 2H, H-8), 7.05–7.00 (m, 2H, H-9), 6.07 (s, 1H, OH), 3.07 (dd, *J* = 12.1, 3.0 Hz, 1H, H-3), 1.87–1.80 (m, 1H, H-4), 1.29 (s, 3H, H-1), 1.11–1.01 (m, 1H, H-5), 0.73–0.66 (m, 1H, H-4), 0.62 (d, *J* = 6.7 Hz, 3H, H-6), 0.60 (d, *J* = 6.7 Hz, 3H, H-6) ppm.

^{13}C NMR (125 MHz, $CDCl_3$, 298 K): δ 164.9 (C-11), 161.9 (d, $J_{C–F}$ = 241.3 Hz, C-10), 145.7 (C-12), 137.5 (C-18), 137.2 (d, $J_{C–F}$ = 3.8 Hz, C-7), 131.4 (d, $J_{C–F}$ = 7.5 Hz, C-8), 130.0 (C-14), 129.0 (C-13), 127.6 (C-16), 127.2 (C-17), 126.7 (C-15), 117.4 (C-19), 114.8 (d, $J_{C–F}$ = 20.0 Hz, C-9), 76.3 (C-2), 53.1 (C-3), 39.3 (C-4), 28.3 (C-1), 25.1 (C-5), 24.2 (C-6), 20.9 (C-6) ppm.

^{19}F NMR (471 MHz, $CDCl_3$, 298 K): δ –116.9—117.2 ppm.

HRMS (ESI) m/z: $[M+H]^+$ calcd for $C_{22}H_{25}FNO^+$ 338.1915, found 338.1920.

IR (thin film): ṽ 3324 (br), 2953 (s), 2930 (s), 1601 (s), 1505 (s), 1488 (s), 1371 (s), 1080 (s) cm^{-1}.

Specific rotation: $[\alpha]_D^{30}$ = −7.6 (*c* 1, CH_2Cl_2).

The **enantiomeric ratio** of **3aj** was determined by SFC analysis (CHIRALPACK IE (25 cm), column temperature 25 °C, solvent CO_2/MeOH = 90/10, flow rate = 2.0 mL/min): t_R = 4.9 min (major), t_R = 5.7 min (minor).

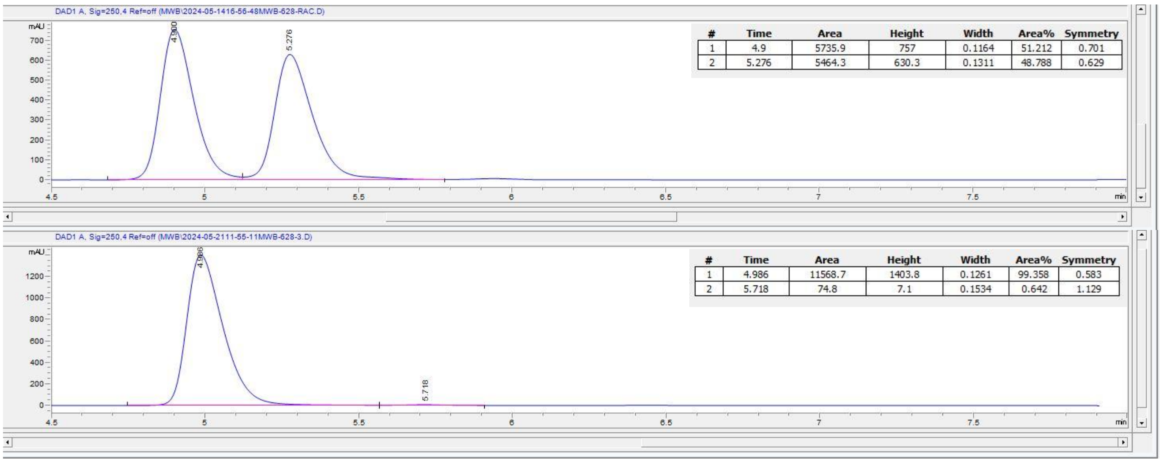

#	Time	Area	Height	Width	Area%	Symmetry
1	4.9	5735.9	757	0.1164	51.212	0.701
2	5.276	5464.3	630.3	0.1311	48.788	0.629

#	Time	Area	Height	Width	Area%	Symmetry
1	4.986	11568.7	1403.8	0.1261	99.358	0.583
2	5.718	74.8	7.1	0.1534	0.642	1.129

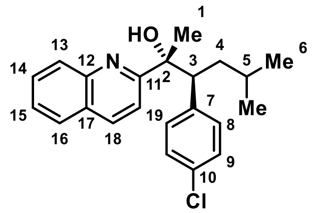

$C_{22}H_{24}ClNO$
M = 353.89

(2*R*,3*S*)-3-(4-Chlorophenyl)-5-methyl-2-(quinolin-2-yl)hexan-2-ol (**3ak**, **GP 6**) was prepared from 1-(quinolin-2-yl)ethan-1-ol (**1a**, 17.3 mg, 0.10 mmol, 100 mol%) and (*E*)-1-chloro-4-(3-methylbut-1-en-1-yl)benzene (**2k**, 45.2 mg, 0.25 mmol, 250 mol%), using [Ir(cod)$_2$]BARF (6.36 mg, 5.0 µmol, 5.0 mol%), and **L5** ((*R*)-DM-SEGPHOS, 3.61 mg, 5.0 µmol, 5.0 mol%) in DCB (0.2 mL) at 110 °C for 3 d. Then, *i*-PrOH (19.1 µL, 15.0 mg, 0.25 mmol, 250 mol%) was added, and the reaction mixture was stirred at 110 °C for 2 d. Purification by flash column chromatography on silica gel (hexane/ethyl acetate from 100/1 to 19/1) afforded **3ak** (29.3 mg, 83% yield, 96% e.e., >20:1 d.r., >20:1 r.r.) as a colorless solid. Diastereomer and regiomer ratios (16:1 d.r., >20:1 r.r.) were determined from the ^{1}H NMR spectrum of crude material.

R_f = 0.5 (Hexane/EtOAc = 9/1).

M.P. 99–100 °C (Hexane/EtOAc).

^{1}H NMR (500 MHz, $CDCl_3$, 298 K): δ 8.21 (d, *J* = 8.6 Hz, 1H, H-18), 8.13 (d, *J* = 8.4 Hz, 1H, H-13), 7.87 (d, *J* = 8.2 Hz, 1H, H-16), 7.79–7.74 (m, 1H, H-14), 7.61–7.56 (m, 1H, H-15), 7.44 (d, *J* = 8.6 Hz, 1H, H-19), 7.41–7.35 (m, 2H, H-8), 7.33–7.29 (m, 2H, H-9), 6.08 (s, 1H, OH), 3.07 (dd, *J* = 12.1, 3.0 Hz, 1H, H-3), 1.87–1.80 (m, 1H, H-4), 1.29 (s, 3H, H-1), 1.11–1.01 (m, 1H, H-5), 0.73–0.65 (m, 1H, H-4), 0.62 (d, *J* = 6.7 Hz, 3H, H-6), 0.60 (d, *J* = 6.7 Hz, 3H, H-6) ppm.

^{13}C NMR (125 MHz, $CDCl_3$, 298 K): δ 164.7 (C-11), 145.7 (C-12), 140.2 (C-7), 137.6 (C-18), 132.3 (C-10), 131.4 (C-8), 130.1 (C-14), 129.0 (C-13), 128.2 (C-9), 127.6 (C-16), 127.2 (C-17), 126.7 (C-15), 117.3 (C-19), 76.2 (C-2), 53.3 (C-3), 39.2 (C-4), 28.4 (C-1), 25.1 (C-5), 24.1 (C-6), 20.9 (C-6) ppm.

HRMS (ESI) m/z: $[M{-}H_2O{+}H]^+$ calcd for $C_{22}H_{23}{}^{35}ClN^+$ 336.1514, found 336.1520.

IR (thin film): ṽ 3325 (br), 2953 (s), 1619(s), 1466 (s), 1411 (s), 1371 (s), 1192 (s), 1012 (s) cm^{-1}.

Specific rotation: $[\alpha]_D^{30}$ = +1.0 (*c* 1, CH_2Cl_2).

The **enantiomeric ratio** of **3ak** was determined by SFC analysis (CHIRALPACK IE (25 cm), column temperature 25 °C, solvent CO_2/MeOH = 90/10, flow rate = 2.0 mL/min): t_R = 6.4 min (major), t_R = 7.8 min (minor).

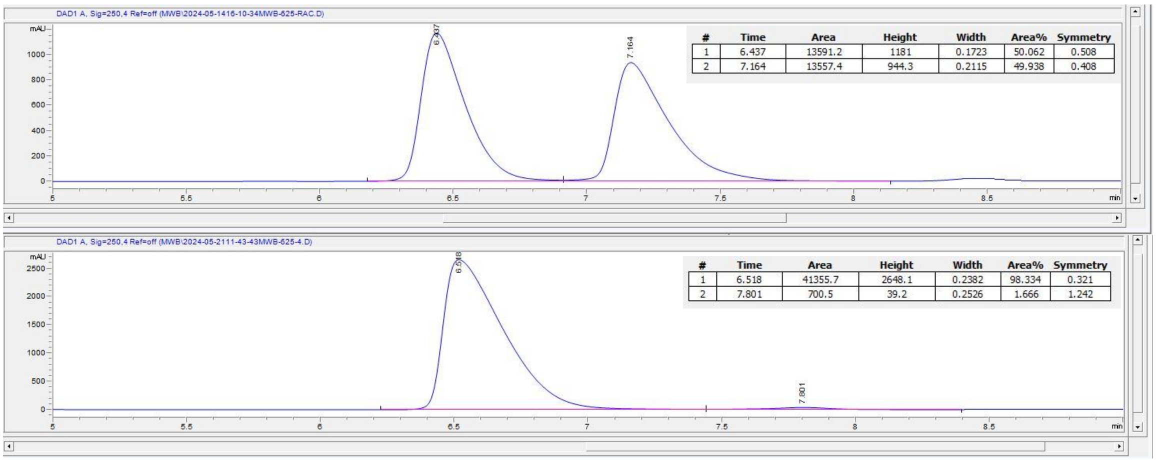

Characteristic signals for diastereomer **3ak'**

^{1}H NMR (500 MHz, $CDCl_3$, 298 K): 3.19 (dd, J = 12.0, 3.4 Hz, 1H), 0.44 (d, J = 6.5 Hz, 3H), 0.42 (d, J = 6.5 Hz, 3H).

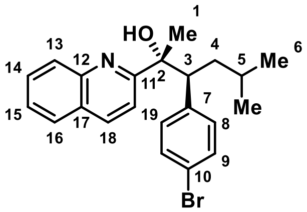

$C_{22}H_{24}BrNO$
M = 398.34

(2*R*,3*S*)-3-(4-Bromophenyl)-5-methyl-2-(quinolin-2-yl)hexan-2-ol (**3al**, **GP 6**) was prepared from 1-(quinolin-2-yl)ethan-1-ol (**1a**, 17.3 mg, 0.10 mmol, 100 mol%) and (*E*)-1-bromo-4-(3-methylbut-1-en-1-yl)benzene (**2l**, 56.3 mg, 0.25 mmol, 250 mol%), using $[Ir(cod)_2]$BARF (6.36

mg, 5.0 μmol, 5.0 mol%), and **L5** ((*R*)-DM-SEGPHOS, 3.61 mg, 5.0 μmol, 5.0 mol%) in DCB (0.2 mL) at 110 °C for 3 d. Then, *i*-PrOH (19.1 μL, 15.0 mg, 0.25 mmol, 250 mol%) was added, and the reaction mixture was stirred at 110 °C for 2 d. Purification by flash column chromatography on silica gel (hexane/ethyl acetate from 100/1 to 19/1) afforded **3al** (29.2 mg, 73% yield, 97% e.e., >20:1 d.r., >20:1 r.r.) as a colorless solid. Diastereomer and regiomer ratios (15:1 d.r., >20:1 r.r.) were determined from the ^{1}H NMR spectrum of crude material.

R*f* = 0.5 (Hexane/EtOAc = 9/1).

M.P. 121–122 °C (Hexane/EtOAc).

^{1}H NMR (500 MHz, $CDCl_3$, 298 K): δ 8.21 (d, *J* = 8.6 Hz, 1H, H-18), 8.13 (d, *J* = 8.5 Hz, 1H, H-13), 7.87 (d, *J* = 8.1 Hz, 1H, H-16), 7.79–7.74 (m, 1H, H-14), 7.61–7.56 (m, 1H, H-15), 7.48–7.41 (m, 3H, H-9 and H-19), 7.32 (d, *J* = 8.0 Hz, 2H, H-8), 6.07 (s, 1H, OH), 3.05 (dd, *J* = 12.1, 3.0 Hz, 1H, H-3), 1.86–1.79 (m, 1H, H-4), 1.29 (s, 3H, H-1), 1.10–1.01 (m, 1H, H-5), 0.72–0.65 (m, 1H, H-4), 0.62 (d, *J* = 6.7 Hz, 3H, H-6), 0.59 (d, *J* = 6.7 Hz, 3H, H-6) ppm.

^{13}C NMR (125 MHz, $CDCl_3$, 298 K): δ 164.7 (C-11), 145.7 (C-12), 140.7 (C-7), 137.6 (C-18), 131.9 (C-8), 131.2 (C-9), 130.1 (C-14), 129.0 (C-13), 127.6 (C-16), 127.2 (C-17), 126.8 (C-15), 120.5 (C-10), 117.3 (C-19), 76.1 (C-2), 53.4 (C-3), 39.2 (C-4), 28.4 (C-1), 25.1 (C-5), 24.1 (C-6), 20.9 (C-6) ppm.

HRMS (ESI) m/z: $[M+H]^+$ calcd for $C_{22}H_{25}{}^{79}BrNO^+$ 398.1114, found 398.1117.

IR (thin film): ṽ 3323 (br), 2963 (s), 2932 (s), 1619 (s), 1601 (s), 1505 (s), 1383 (s), 1151 (s), 1070 (s) cm^{-1}.

Specific rotation: $[\alpha]_D^{30}$ = +2.5 (*c* 1, CH_2Cl_2).

The **enantiomeric ratio** of **3al** was determined by SFC analysis (CHIRALPACK IE (25 cm), column temperature 25 °C, solvent CO_2/MeOH = 90/10, flow rate = 2.0 mL/min): t_R = 7.7 min (major), t_R = 9.1 min (minor).

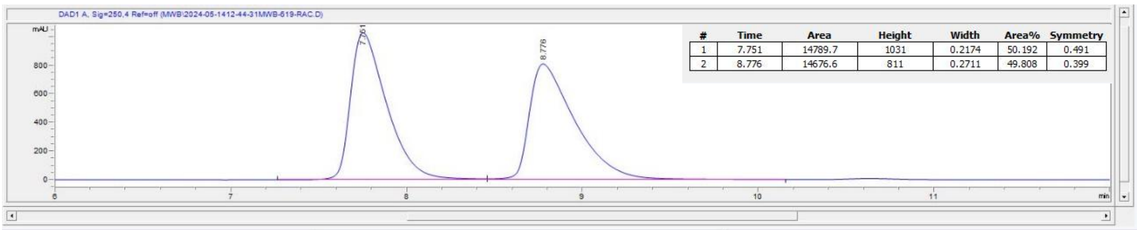

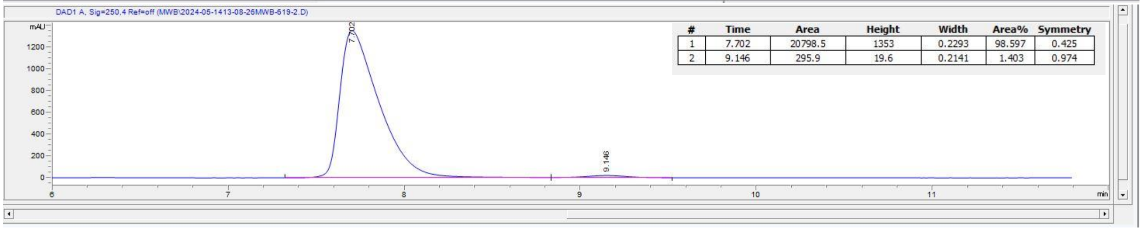

Crystal **3al** was obtained through recrystallization in the solution of DCM and methanol at room temperature. The absolute configuration of **3al** was confirmed unambiguously by **X-ray diffraction analysis**, and other compounds were assigned by analogy. CCDC 2480111 contains the supplementary crystallographic data for this compound.

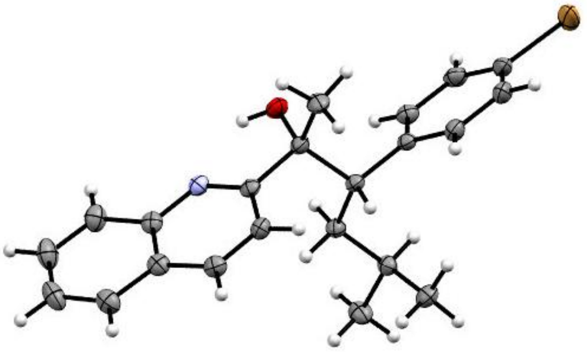

Figure S1. Molecular structure of **3al**. Thermal ellipsoids represent 50% probability level.

Characteristic signals for diastereomer **3al'**

^{1}H NMR (500 MHz, $CDCl_3$, 298 K): 3.17 (dd, *J* = 12.2, 3.3 Hz, 1H), 0.43 (d, *J* = 6.5 Hz, 3H), 0.41 (d, *J* = 6.5 Hz, 3H).

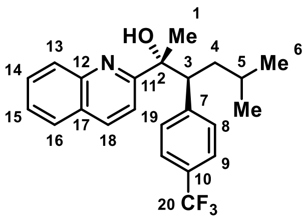

$C_{23}H_{24}F_3NO$
M = 387.45

(2*R*,3*S*)-5-Methyl-2-(quinolin-2-yl)-3-(4-(trifluoromethyl)phenyl)hexan-2-ol (**3am**, **GP 6**) was prepared from 1-(quinolin-2-yl)ethan-1-ol (**1a**, 17.3 mg, 0.10 mmol, 100 mol%) and (*E*)-1-

(3-methylbut-1-en-1-yl)-4-(trifluoromethyl)benzene (**2m**, 53.6 mg, 0.25 mmol, 250 mol%), using [Ir(cod)$_2$]BARF (12.7 mg, 10 μmol, 10 mol%), and **L5** ((*R*)-DM-SEGPHOS, 7.23 mg, 10 μmol, 10 mol%) in H_2O (0.2 mL) at 110 °C for 3 d. Then, *i*-PrOH (19.1 μL, 15.0 mg, 0.25 mmol, 250 mol%) was added, and the reaction mixture was stirred at 110 °C for 2 d. Purification by flash column chromatography on silica gel (hexane/ethyl acetate from 100/1 to 19/1) afforded **3am** (31.7 mg, 82% yield, 97% e.e., >20:1 d.r., >20:1 r.r.) as a colorless solid. Diastereomer and regiomer ratios (16:1 d.r., >20:1 r.r.) were determined from the ^{1}H NMR spectrum of crude material.

R*f* = 0.3 (Hexane/EtOAc = 9/1).

M.**P**. 158–159 °C (Hexane/EtOAc).

^{1}H NMR (500 MHz, $CDCl_3$, 298 K): δ 8.23 (d, *J* = 8.6 Hz, 1H, H-18), 8.13 (d, *J* = 8.4 Hz, 1H, H-13), 7.88 (d, *J* = 8.1 Hz, 1H, H-16), 7.80–7.75 (m, 1H, H-14), 7.62–7.55 (m, 5H, H-8, H-9 and H-15), 7.47 (d, *J* = 8.6 Hz, 1H, H-19), 6.13 (s, 1H, OH), 3.16 (dd, *J* = 12.1, 3.0 Hz, 1H, H-3), 1.92–1.84 (m, 1H, H-4), 1.28 (s, 3H, H-1), 1.08–0.96 (m, 1H, H-5), 0.73–0.66 (m, 1H, H-4), 0.62 (d, *J* = 6.7 Hz, 3H, H-6), 0.60 (d, *J* = 6.7 Hz, 3H, H-6) ppm.

^{13}C NMR (125 MHz, $CDCl_3$, 298 K): δ 164.5 (C-11), 146.0 (C-7 or C-12), 145.7 (C-7 or C-12), 137.7 (C-18), 130.4 (C-8), 130.2 (C-14), 129.0 (C-13), 128.9 (q, $J_{C\text{–}F}$ = 31.7 Hz, C-10), 127.7 (C-16), 127.2 (C-17), 126.8 (C-15), 125.0 (q, $J_{C\text{–}F}$ = 3.8 Hz, C-9), 124.6 (q, $J_{C\text{–}F}$ = 270.0 Hz, C-20), 117.2 (C-19), 76.1 (C-2), 53.2 (C-3), 39.2 (C-4), 28.5 (C-1), 25.1 (C-5), 24.1 (C-6), 20.9 (C-6) ppm.

^{19}F NMR (471 MHz, $CDCl_3$, 298 K): δ –62.3 ppm.

HRMS (ESI) m/z: $[M+H]^+$ calcd for $C_{23}H_{25}F_3NO^+$ 338.1883, found 338.1887.

IR (thin film): ṽ 3326 (br), 2958 (s), 2927 (s), 1617(s), 1601 (s), 1505 (s), 1324 (s), 1117 (s), 1067 (s) cm^{-1}.

Specific rotation: $[\alpha]_D^{30}$ = +3.8 (*c* 1, CH_2Cl_2).

The **enantiomeric ratio** of **3am** was determined by SFC analysis (CHIRALPACK IE (25 cm), column temperature 25 °C, solvent CO_2/MeOH = 90/10, flow rate = 2.0 mL/min): t_R = 3.3 min (major), t_R = 3.7 min (minor).

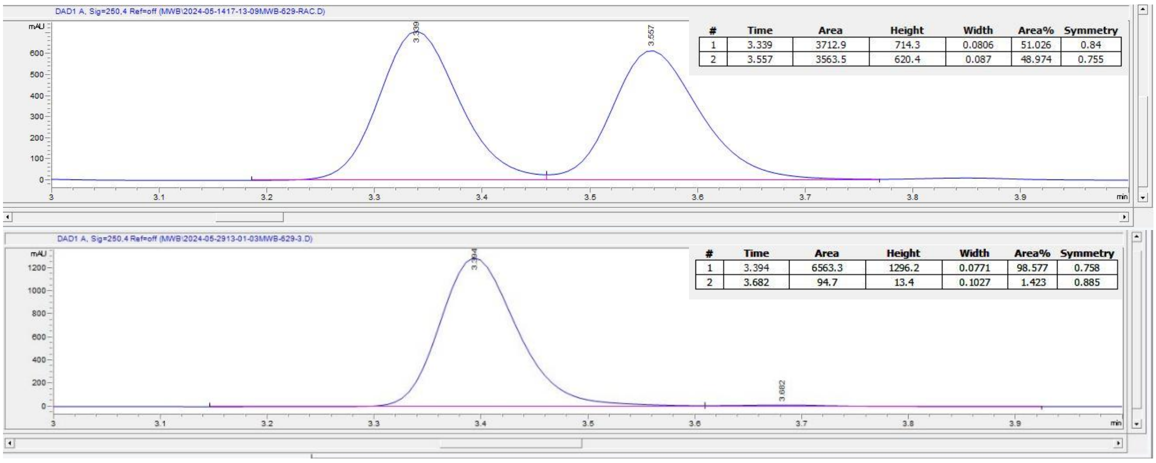

Characteristic signals for diastereomer **3am'**

^{1}H NMR (500 MHz, $CDCl_3$, 298 K): 0.43 (d, J = 6.5 Hz, 3H), 0.41 (d, J = 6.5 Hz, 3H).

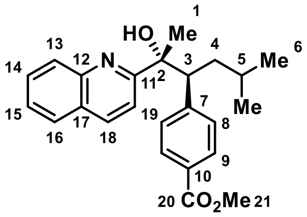

$C_{24}H_{27}NO_3$
M = 377.48

Methyl 4-((2*R*,3*S*)-2-hydroxy-5-methyl-2-(quinolin-2-yl)hexan-3-yl)benzoate (**3an**, **GP 6**) was prepared from 1-(quinolin-2-yl)ethan-1-ol (**1a**, 17.3 mg, 0.10 mmol, 100 mol%) and methyl (*E*)-4-(3-methylbut-1-en-1-yl)benzoate (**2n**, 51.1 mg, 0.25 mmol, 250 mol%), using [Ir(cod)$_2$]BARF (6.36 mg, 5.0 μmol, 5.0 mol%), and **L5** ((*R*)-DM-SEGPHOS, 3.61 mg, 5.0 μmol, 5.0 mol%) in DCB (0.2 mL) at 110 °C for 3 d. Then, *i*-PrOH (19.1 μL, 15.0 mg, 0.25 mmol, 250 mol%) was added, and the reaction mixture was stirred at 110 °C for 2 d. Purification by flash column chromatography on silica gel (hexane/ethyl acetate from 100/1 to 19/1) afforded **3an** (30.0 mg, 79% yield, 96% e.e., >20:1 d.r., >20:1 r.r.) as a colorless solid. Diastereomer and regiomer ratios (16:1 d.r., >20:1 r.r.) were determined from the ^{1}H NMR spectrum of crude material.

R$_f$ = 0.2 (Hexane/EtOAc = 9/1).

M.P. 116–117 °C (Hexane/EtOAc).

^{1}H NMR (500 MHz, $CDCl_3$, 298 K): δ 8.21 (d, J = 8.6 Hz, 1H, H-18), 8.13 (d, J = 8.5 Hz, 1H, H-13), 8.01 (d, J = 8.5 Hz, 2H, H-9), 7.86 (d, J = 8.2 Hz, 1H, H-16), 7.79–7.74 (m, 1H, H-14), 7.61–7.56 (m, 1H, H-15), 7.52 (d, J = 8.5 Hz, 2H, H-8), 7.45 (d, J = 8.6 Hz, 1H, H-19), 6.12 (s, 1H, OH), 3.92 (s, 3H, H-21), 3.16 (dd, J = 12.1, 3.0 Hz, 1H, H-3), 1.94–1.86 (m, 1H, H-4), 1.29

(s, 3H, H-1), 1.07–0.97 (m, 1H, H-5), 0.77–0.70 (m, 1H, H-4), 0.61 (d, J = 6.7 Hz, 3H, H-6), 0.60 (d, J = 6.7 Hz, 3H, H-6) ppm.

^{13}C NMR (125 MHz, $CDCl_3$, 298 K): δ 167.4 (C-20), 164.6 (C-11), 147.5 (C-7), 145.7 (C-12), 137.6 (C-18), 130.2 (C-8), 130.1 (C-14), 129.4 (C-9), 129.0 (C-13), 128.6 (C-10), 127.6 (C-16), 127.2 (C-17), 126.8 (C-15), 117.3 (C-19), 76.2 (C-2), 54.1 (C-3), 52.1 (C-21), 39.2 (C-4), 28.3 (C-1), 25.2 (C-5), 24.1 (C-6), 20.9 (C-6) ppm.

HRMS (ESI) m/z: $[M+H]^+$ calcd for $C_{24}H_{28}NO_3^+$ 378.2064, found 378.2068.

IR (thin film): ṽ 3335 (s), 2952 (s), 2931 (s), 1717 (s), 1610 (s), 1599 (s), 1504 (s), 1384 (s), 1273 (s), 1113 (s), 1079 (s) cm^{-1}.

Specific rotation: $[\alpha]_D^{30}$ = −3.7 (c 1, CH_2Cl_2).

The **enantiomeric ratio** of **3an** was determined by SFC analysis (CHIRALPACK IE (25 cm), column temperature 25 °C, solvent CO_2/MeOH = 90/10, flow rate = 2.0 mL/min): t_R = 13.9 min (major), t_R = 16.9 min (minor).

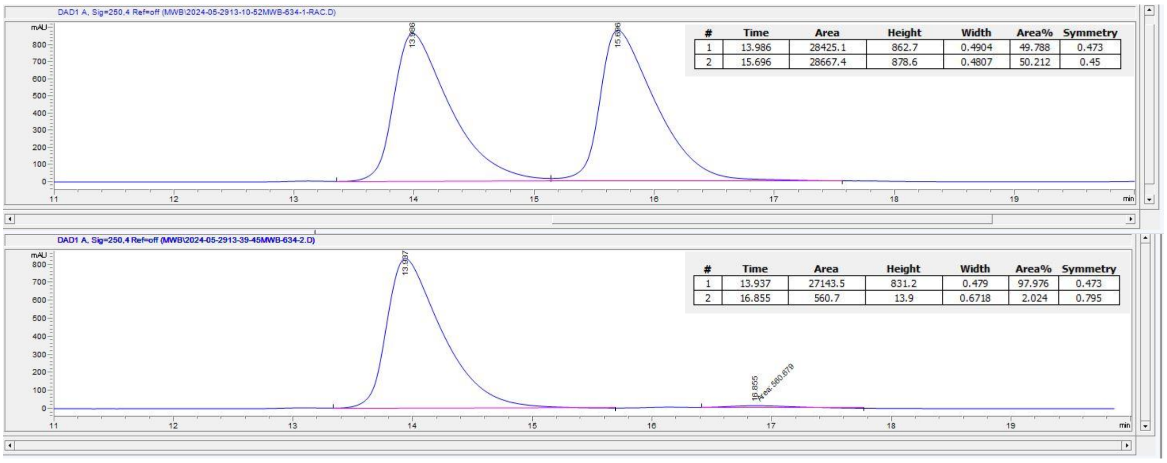

Characteristic signals for diastereomer **3an'**

^{1}H NMR (500 MHz, $CDCl_3$, 298 K): 3.29 (dd, J = 12.0, 3.3 Hz, 1H), 0.43 (d, J = 6.5 Hz, 3H), 0.41 (d, J = 6.5 Hz, 3H).

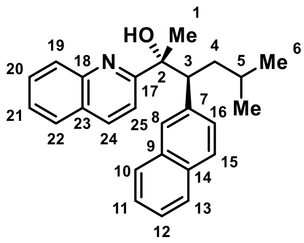

$C_{26}H_{27}NO$
M = 369.51

(2*R*,3*S*)-5-Methyl-3-(naphthalen-2-yl)-2-(quinolin-2-yl)hexan-2-ol (**3ao**, **GP 6**) was prepared from 1-(quinolin-2-yl)ethan-1-ol (**1a**, 17.3 mg, 0.10 mmol, 100 mol%) and (*E*)-2-(3-methylbut-1-en-1-yl)naphthalene (**2o**, 49.1 mg, 0.25 mmol, 250 mol%), using [Ir(cod)$_2$]BARF (6.36 mg, 5.0 μmol, 5.0 mol%), and **L5** ((*R*)-DM-SEGPHOS, 3.61 mg, 5.0 μmol, 5.0 mol%) in DCB (0.2 mL) at 110 °C for 3 d. Then, *i*-PrOH (19.1 μL, 15.0 mg, 0.25 mmol, 250 mol%) was added, and the reaction mixture was stirred at 110 °C for 2 d. Purification by flash column chromatography on silica gel (hexane/ethyl acetate from 100/1 to 19/1) afforded **3ao** (28.4 mg, 77% yield, 98% e.e., >20:1 d.r., >20:1 r.r.) as a colorless solid. Diastereomer and regiomer ratios (16:1 d.r., >20:1 r.r.) were determined from the ^{1}H NMR spectrum of crude material.

R*f* = 0.5 (Hexane/EtOAc = 9/1).

M.P. 153–154 °C (Hexane/ethyl acetate).

^{1}H NMR (500 MHz, $CDCl_3$, 298 K): δ 8.21 (d, *J* = 8.6 Hz, 1H, H-24), 8.16 (d, *J* = 8.4 Hz, 1H, H-19), 7.90–7.82 (m, 5H, Ar and H-22), 7.80–7.75 (m, 1H, H-20), 7.75–7.65 (m, 1H, Ar), 7.62–7.56 (m, 1H, H-21), 7.53–7.43 (m, 3H, Ar and H-25), 6.15 (s, 1H, OH), 3.28 (dd, *J* = 12.0, 3.0 Hz, 1H, H-3), 2.07–1.99 (m, 1H, H-4), 1.35 (s, 3H, H-1), 1.15–1.05 (m, 1H, H-5), 0.84–0.76 (m, 1H, H-4), 0.65 (d, *J* = 5.6 Hz, 3H, H-6), 0.59 (d, *J* = 5.6 Hz, 3H, H-6) ppm.

^{13}C NMR (125 MHz, $CDCl_3$, 298 K): δ 165.1 (C-17), 145.8 (C-18), 139.4 (C-7), 137.5 (C-24), 133.4 (C-9 or C-14), 132.7 (C-9 or C-14), 130.0 (C-20), 129.1 (C-19), 127.9 (Ar), 127.7 (Ar), 127.7 (Ar), 127.7 (Ar), 127.6 (C-22), 127.2 (C-23), 126.7 (C-21), 125.8 (Ar), 125.4 (Ar), 117.5 (C-25), 76.6 (C-2), 54.1 (C-3), 39.2 (C-4), 28.5 (C-1), 25.2 (C-5), 24.2 (C-6), 21.0 (C-6) ppm.

HRMS (ESI) m/z: $[M+H]^+$ calcd for $C_{26}H_{28}NO^+$ 370.2165, found 370.2170.

IR (thin film): ṽ 3330 (s), 2949 (s), 2925 (s), 1618(s), 1598 (s), 1504 (s), 1385 (s), 1152 (s), 1075 (s) cm^{-1}.

Specific rotation: $[\alpha]_D^{30}$ = −28.7 (*c* 1, CH_2Cl_2).

The **enantiomeric ratio** of **3ao** was determined by SFC analysis (CHIRALPACK OD-H (25 cm), column temperature 25 °C, solvent CO_2/MeOH = 90/10, flow rate = 2.0 mL/min): t_R = 9.2 min (major), t_R = 10.7 min (minor).

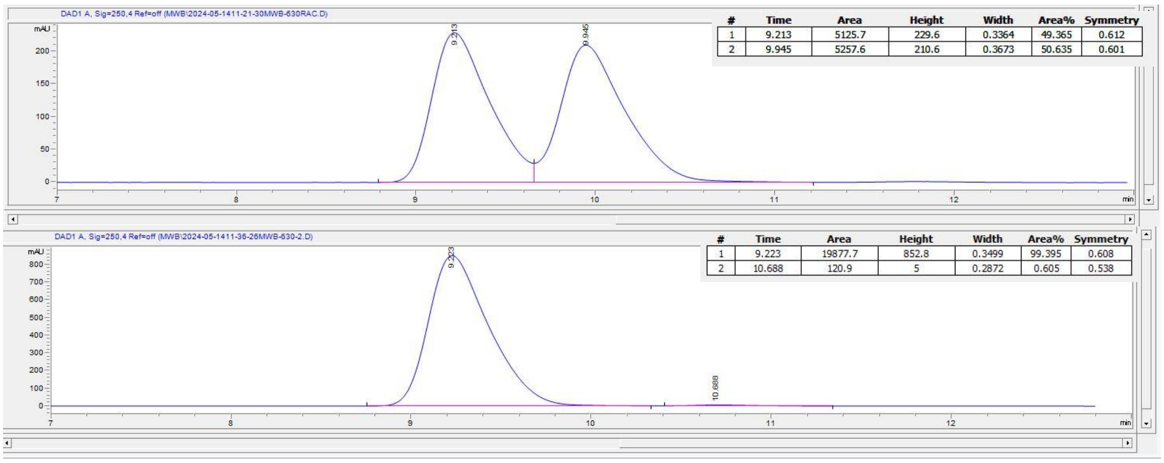

Characteristic signal for diastereomer **3ao'**

^{1}H NMR (500 MHz, $CDCl_3$, 298 K): 3.49 (dd, *J* = 12.0, 3.3 Hz, 1H).

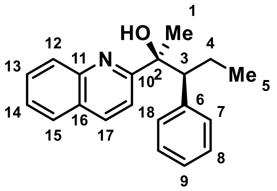

$C_{20}H_{21}NO$
M = 291.39

(2*S*,3*S*)-3-Phenyl-2-(quinolin-2-yl)pentan-2-ol (***ent*-3aa'**, **GP 6**) was prepared from 1-(quinolin-2-yl)ethan-1-ol (**1a**, 17.3 mg, 0.10 mmol, 100 mol%) and (*Z*)-1-propenylbenzene (***Z*-2a**, 32.4 µL, 29.5 mg, 0.25 mmol, 250 mol%), using [Ir(cod)$_2$]BARF (6.36 mg, 5.0 µmol, 5.0 mol%) and **L12** ((2*R*,2'*R*,3*R*,3'*R*)-BIBOP, 1.93 mg, 5.0 µmol, 5.0 mol%) in DCB (0.2 mL) at 110 °C for 36 h. Purification by flash column chromatography on silica gel (hexane/ethyl acetate from 100/1 to 19/1) afforded ***ent*-3aa'** (15.6 mg, 54% yield, 91% e.e., >20:1 d.r., >20:1 r.r.) as a colorless solid. Diastereomer and regiomer ratios (2.5:1 d.r., >20:1 r.r.) were determined from the ^{1}H NMR spectrum of crude material.

R_f = 0.4 (Hexane/EtOAc = 10/1).

M.P. 70–71 °C (Hexane/EtOAc).

^{1}H NMR (500 MHz, $CDCl_3$, 298 K): δ 8.04 (d, *J* = 8.6 Hz, 1H, H-17), 7.90 (d, *J* = 8.5 Hz, 1H, H-12), 7.74 (d, *J* = 8.1 Hz, 1H, H-15), 7.65–7.61 (m, 1H, H-13), 7.49–7.45 (m, 1H, H-14), 7.27 (d, *J* = 8.6 Hz, 1H, H-18), 7.05–7.00 (m, 4H, H-7 and H-8), 7.00–6.95 (m, 1H, H-9), 5.92 (s, 1H, OH), 2.95 (dd, *J* = 11.8, 3.3 Hz, 1H, H-3), 2.24–2.15 (m, 1H, H-4), 1.86–1.76 (m, 1H, H-4), 1.67 (s, 3H, H-1), 0.72 (t, *J* = 7.4 Hz, 3H, H-5) ppm.

^{13}C NMR (125 MHz, $CDCl_3$, 298 K): δ 164.1 (C-10), 145.6 (C-11), 140.7 (C-6), 136.4 (C-17), 129.7 (C-7), 129.6 (C-13), 128.9 (C-12), 127.5 (C-8), 127.4 (C-15), 126.9 (C-16), 126.3 (C-14), 126.2 (C-9), 118.6 (C-18), 76.2 (C-2), 58.5 (C-3), 27.5 (C-1), 22.2 (C-4), 12.8 (C-5) ppm.

HRMS (ESI) m/z: $[M+H]^+$ calcd for $C_{20}H_{22}NO^+$ 292.1696, found 292.1698.

IR (thin film): ṽ 3338 (s), 2970 (s), 2945 (s), 1601 (s), 1504 (s), 1383 (s), 1149 (s), 1085 (s) cm^{-1}.

Specific rotation: $[\alpha]_D^{25}$ = +25.9 (*c* 0.5, CH_2Cl_2).

The **enantiomeric ratio** of ***ent*-3aa**' was determined by SFC analysis (CHIRALPACK SC (25 cm), column temperature 25 °C, solvent CO_2/MeOH = 90/10, flow rate = 2.0 mL/min): t_R = 4.3 min (minor), t_R = 4.8 min (major).

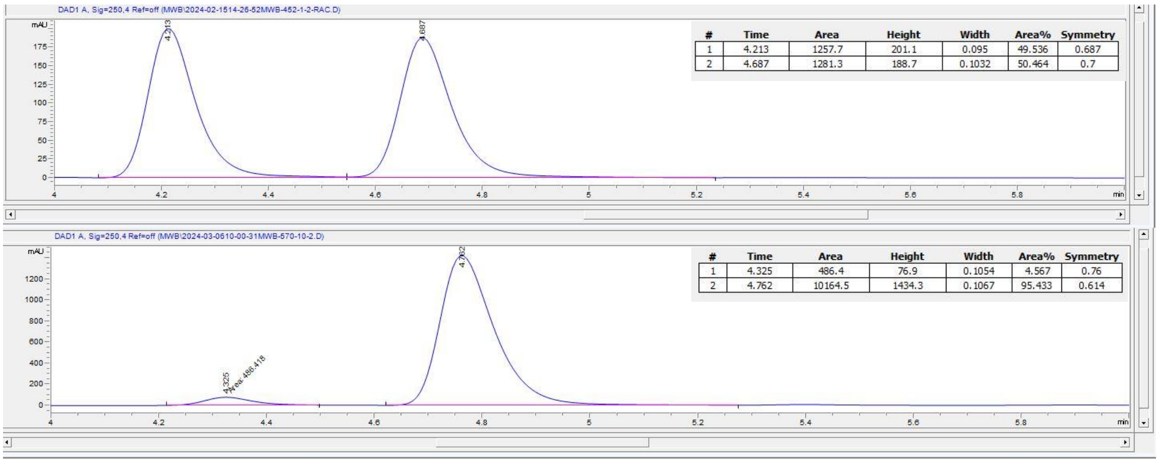

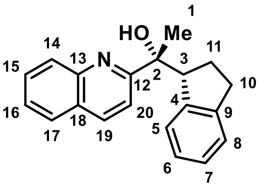

$C_{20}H_{19}NO$
M = 289.38

(*R*)-1-((*R*)-2,3-Dihydro-1*H*-inden-1-yl)-1-(quinolin-2-yl)ethan-1-ol (**3ap**, **GP 6**) was prepared from 1-(quinolin-2-yl)ethan-1-ol (**1a**, 17.3 mg, 0.10 mmol, 100 mol%) and indene (**2p**, 29.3 µL, 29.0 mg, 0.25 mmol, 250 mol%), using $[Ir(cod)_2]$BARF (6.36 mg, 5.0 µmol, 5.0 mol%), and **L11** ((*S*,*S*)-Ph-BPE, 2.53 mg, 5.0 µmol, 5.0 mol%) in H_2O (0.2 mL) at 110 °C for 24 h. Purification by flash column chromatography on silica gel (hexane/ethyl acetate from 100/1 to 19/1) afforded **3ap** (22.6 mg, 78% yield, 98% e.e., >20:1 d.r., >20:1 r.r.) as a colorless solid. Diastereomer and regiomer ratios (>20:1 d.r., >20:1 r.r.) were determined from the ^{1}H NMR spectrum of crude material.

R$_f$ = 0.3 (Hexane/EtOAc = 9/1).

M.P. 118–119 °C (Hexane/EtOAc).

^{1}H NMR (500 MHz, $CDCl_3$, 298 K): δ 8.09–8.04 (m, 2H, H-14 and H-19), 7.84 (d, *J* = 8.1 Hz, 1H, H-17), 7.76–7.71 (m, 1H, H-15), 7.59–7.54 (m, 1H, H-16), 7.14–7.08 (m, 3H, H-7, H-8 and H-20), 6.96–6.90 (m, 1H, H-6), 6.63 (d, *J* = 7.7 Hz, 1H, H-5), 5.95 (s, 1H, OH), 3.80–3.75 (m, 1H, H-3), 2.67–2.60 (m, 1H, H-10), 2.44–2.31 (m, 2H, H-10 and H-11), 2.25–2.15 (m, 1H, H-11), 1.75 (s, 3H, H-1) ppm.

^{13}C NMR (125 MHz, $CDCl_3$, 298 K): δ 163.9 (C-12), 146.1 (C-9 or C13), 145.8 (C-9 or C13), 143.1 (C-4), 136.7 (C-19), 129.9 (C-15), 129.1 (C-14), 127.6 (C-17), 127.2 (C-18), 126.9 (C-16), 126.6 (C-7), 125.7 (C-5 or C-6), 125.5 (C-5 or C-6), 124.6 (C-8), 118.3 (C-20), 76.2 (C-2), 56.4 (C-3), 31.5 (C-10), 27.4 (C-11), 27.3 (C-1) ppm.

HRMS (ESI) m/z: $[M–H_2O+H]^+$ calcd for $C_{20}H_{18}N^+$ 272.1434, found 272.1447.

IR (thin film): ṽ 3324 (s), 2966 (s), 2914 (s), 1618(s), 1600 (s), 1504 (s), 1383 (s), 1160 (s), 1078 (s) cm^{-1}.

Specific rotation: $[\alpha]_D^{24}$ = −62.1 (*c* 0.5, CH_2Cl_2).

The **enantiomeric ratio** of **3ap** was determined by SFC analysis (CHIRALPACK IE (25 cm), column temperature 25 °C, solvent CO_2/MeOH = 90/10, flow rate = 2.0 mL/min): t_R = 13.3 min (major), t_R = 16.4 min (minor).

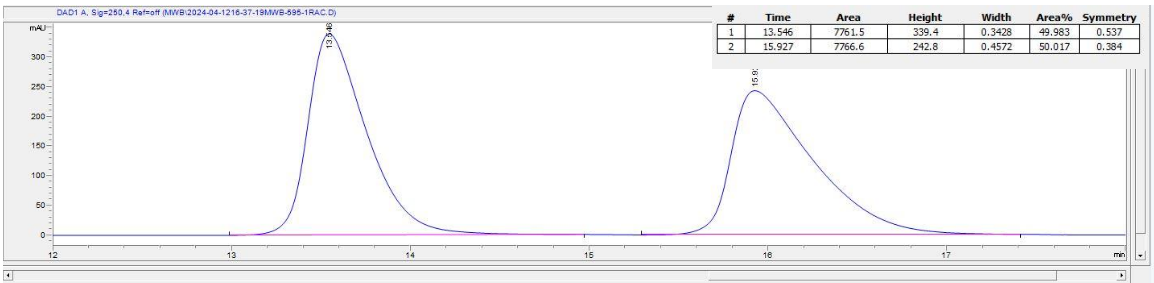

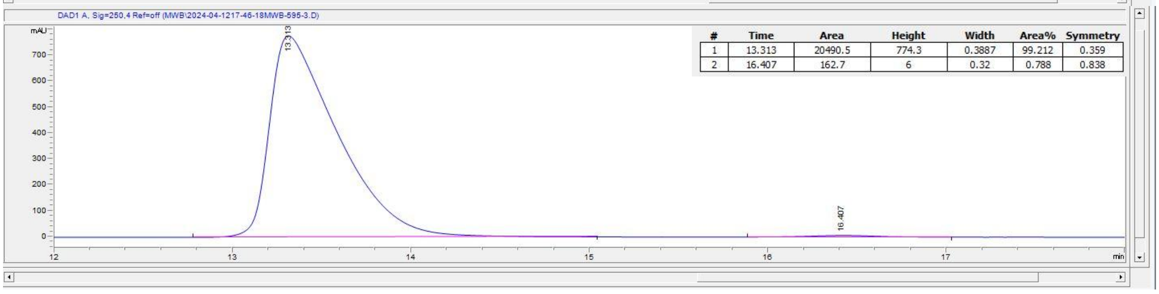

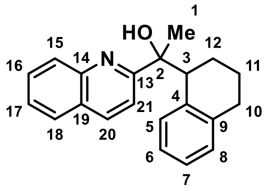

$C_{21}H_{21}NO$
M = 303.41

1-(Quinolin-2-yl)-1-(1,2,3,4-tetrahydronaphthalen-1-yl)ethan-1-ol (**3aq**, **GP 6**) was prepared from 1-(quinolin-2-yl)ethan-1-ol (**1a**, 17.3 mg, 0.10 mmol, 100 mol%) and 1,2-dihydronaphthalene (**2q**, 32.6 µL, 32.5 mg, 0.25 mmol, 250 mol%), using [Ir(cod)$_2$]BARF (6.36 mg, 5.0 µmol, 5.0 mol%), and **L1** ((*R*)-BINAP, 3.11 mg, 5.0 µmol, 5.0 mol%) in DCB (0.2 mL) at 110 °C for 24 h. Purification by flash column chromatography on silica gel (hexane/ethyl acetate from 100/1 to 19/1) afforded **3aq** (17.5 mg, 58% yield, 28%/94% e.e., 3:1 d.r., >20:1 r.r.) as a colorless solid. Diastereomer and regiomer ratios (3:1 d.r., >20:1 r.r.) were determined from the ^{1}H NMR spectrum of crude material.

R*f* = 0.3 (Hexane/EtOAc = 9/1).

M.**P**. 72–73 °C (Hexane/Ethyl acetate).

^{1}H NMR (500 MHz, CDCl$_3$, 298 K): δ 8.20 (minor, d, *J* = 8.6 Hz, 1H, H-20), 8.12 (minor, d, *J* = 8.4 Hz, 1H, H-15), 8.08 (major, d, *J* = 8.4 Hz, 1H, H-20), 8.02 (major, d, *J* = 8.6 Hz, 1H, H-15), 7.87 (minor, d, *J* = 8.1 Hz, 1H, H-18), 7.83 (major, d, *J* = 8.1 Hz, 1H, H-18), 7.78–7.74 (minor, m, 1H, H-16), 7.73–7.69 (major, m, 1H, H-16), 7.60–7.52 (minor, m, 2H, H-17 and H-21; Major, m, 1H, H-17), 7.29–7.25 (minor, 1H, H-5), 7.21–7.13 (minor, m, 3H, H-6, H-7 and H-8), 7.11 (major, d, *J* = 8.4 Hz, 1H, H-21), 7.09–7.03 (major, m, 2H, H-7 and H-8), 6.84–6.79 (major, m, 1H, H-6), 6.64 (major, d, *J* = 7.8 Hz, 1H, H-5), 5.72 (major, s, 1H, OH), 5.64 (minor, s, 1H, OH), 3.49 (major, dd, *J* = 6.4, 4.7Hz, 1H, H-3), 3.43 (minor, dd, *J* = 7.6, 4.4Hz, 1H, H-3), 2.95–2.85 (minor, m, 1H, H-10), 2.67–2.56 (minor, m, 1H, H-10; major, m, 1H, H-10), 2.50–2.43 (major, m, 1H, H-10), 2.25–2.18 (major, m, 1H, H-12), 2.09–2.00 (minor, m, 1H, H-11), 1.87–1.75 (major, m, 2H, H-11 and H-12), 1.73 (major, s, 3H, H-1), 1.61 (minor, s, 3H, H-1), 1.58–1.44 (minor, m, 2H, H-12; Major, m, 1H, H-11), 1.41–1.34 (minor, m, 1H, H-11) ppm.

^{13}C NMR (125 MHz, CDCl$_3$, 298 K): δ 165.2 (minor, C-13), 164.9 (major, C-13), 146.0 (minor, C-14), 145.8 (major, C-14), 141.3 (minor, C-9), 140.2 (major, C-9), 137.2 (minor, C-20), 136.6 (major, C-4 and C-20), 136.4 (minor, C-4), 131.4 (minor, C-5), 130.3 (major, C-5), 129.9 (minor, C-16), 129.8 (major, C-16), 129.0 (major, C-15 or C-8), 129.0 (minor, C- C-15 or C-8), 129.0 (minor, C- C-15 or C-8), 128.9 (major, C-15 or C-8), 127.6 (minor, C-18), 127.5 (major, C-18), 127.2 (minor, C-19), 127.1 (major, C-19), 126.6 (major and minor, C-17), 126.3 (minor, C-7), 126.1 (major, C-7), 124.6 (major, C-6), 124.6 (minor, C-6), 118.7 (major, C-21), 118.0 (minor,

C-21), 77.9 (minor, C-2), 77.4 (major, C-2), 48.3 (major, C-3), 47.0 (minor, C-3), 29.6 (major, C-10), 29.5 (minor, C-10), 29.0 (minor, C-1), 27.5 (major, C-1), 25.0 (minor, C-12), 24.8 (major, C-12), 21.4 (minor, C-11), 20.5 (major, C-11) ppm.

HRMS (ESI) m/z: $[M–H_2O+H]^+$ calcd for $C_{21}H_{20}N^+$ 286.1590, found 286.1601.

IR (thin film): ṽ 3318 (br), 2929 (s), 2865 (s), 1618 (s), 1599 (s), 1503 (s), 1383 (s), 1136 (s), 1077 (s) cm^{-1}.

Specific rotation: $[\alpha]_D^{24}$ = +16.6 (*c* 0.5, CH_2Cl_2).

The **enantiomeric ratio** of **3aq** (major diastereomer) was determined by SFC analysis (CHIRALPACK IE (25 cm), column temperature 25 °C, solvent CO_2/MeOH = 90/10, flow rate = 2.0 mL/min): t_R = 15.3 min (major), t_R = 16.3 min (minor).

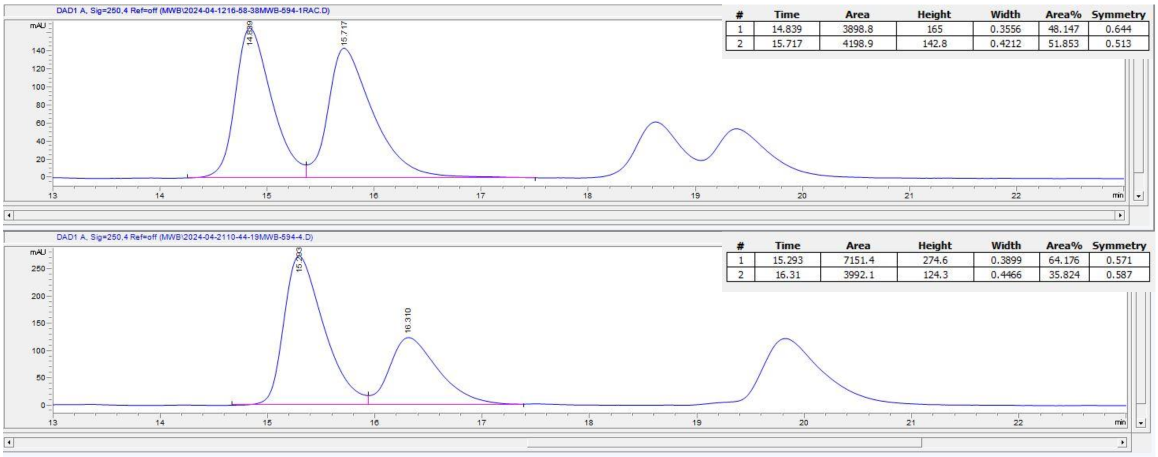

#	Time	Area	Height	Width	Area%	Symmetry
1	14.839	3898.8	165	0.3556	48.147	0.644
2	15.717	4198.9	142.8	0.4212	51.853	0.513

#	Time	Area	Height	Width	Area%	Symmetry
1	15.293	7151.4	274.6	0.3899	64.176	0.571
2	16.31	3992.1	124.3	0.4466	35.824	0.587

The **enantiomeric ratio** of **3aq** (minor diastereomer) was determined by SFC analysis (CHIRALPACK SC (25 cm), column temperature 25 °C, solvent CO_2/MeOH = 90/10, flow rate = 2.0 mL/min): t_R = 8.5 min (major), t_R = 9.6 min (minor).

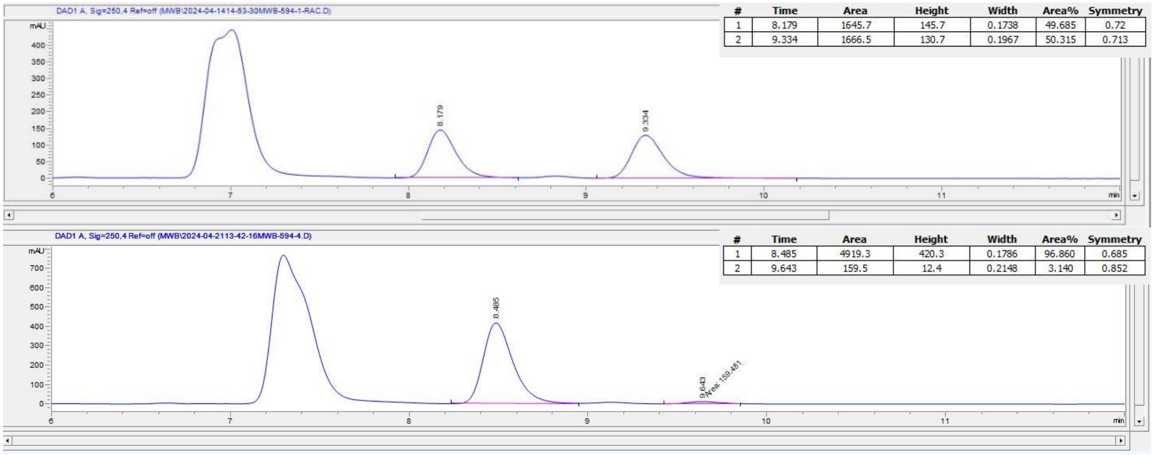

#	Time	Area	Height	Width	Area%	Symmetry
1	8.179	1645.7	145.7	0.1738	49.685	0.72
2	9.334	1666.5	130.7	0.1967	50.315	0.713

#	Time	Area	Height	Width	Area%	Symmetry
1	8.485	4919.3	420.3	0.1786	96.860	0.685
2	9.643	159.5	12.4	0.2148	3.140	0.852

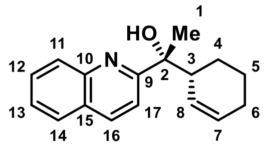

$C_{17}H_{19}NO$
M = 253.35

(*R*)-1-((*R*)-Cyclohex-2-en-1-yl)-1-(quinolin-2-yl)ethan-1-ol (**3ar**, **GP 6**) was prepared from 1-(quinolin-2-yl)ethan-1-ol (**1a**, 17.3 mg, 0.10 mmol, 100 mol%) and 1,3-cyclohexadiene (**2r**, 23.7 µL, 20.0 mg, 0.25 mmol, 250 mol%), using [Ir(cod)$_2$]BARF (6.36 mg, 5.0 µmol, 5.0 mol%) and **L11** ((*S*,*S*)-Ph-BPE, 2.53 mg, 5.0 µmol, 5.0 mol%) in DCB (0.2 mL) at 110 °C for 6 h. Purification by flash column chromatography on silica gel (hexane/ethyl acetate from 100/1 to 19/1) afforded **3ar** (23.6 mg, 93% yield, 97% e.e., >20:1 d.r., >20:1 r.r.) as a colorless solid. Diastereomer and regiomer ratios (>20:1 d.r., >20:1 r.r.) were determined from the ^{1}H NMR spectrum of crude material.

R*f* = 0.5 (Hexane/EtOAc = 9/1).

M.P. 92–93 °C (Hexane/EtOAc).

^{1}H NMR (500 MHz, $CDCl_3$, 298 K): δ 8.16 (d, *J* = 8.6 Hz, 1H, H-16), 8.08 (d, *J* = 8.4 Hz, 1H, H-11), 7.83 (d, *J* = 8.1 Hz, 1H, H-14), 7.75–7.70 (m, 1H, H-12), 7.57–7.52 (m, 1H, H-13), 7.45 (d, *J* = 8.7 Hz, 1H, H-17), 5.79 (s, 1H, OH), 5.77–5.71 (m, 1H, H-5), 5.32–5.27 (m, 1H, H-4), 2.72–2.65 (m, 1H, H-3), 1.99–1.91 (m, 3H, H-6 and H-8), 1.76–1.70 (m, 1H, H-7), 1.60 (s, 3H, H-1), 1.57–1.44 (m, 2H, H-7 and H-8) ppm.

^{13}C NMR (125 MHz, $CDCl_3$, 298 K): δ 164.7 (C-9), 145.9 (C-10), 137.0 (C-16), 130.1 (C-5), 129.9 (C-12), 129.0 (C-11), 127.7 (C-14), 127.6 (C-4), 127.2 (C-15), 126.6 (C-13), 118.1 (C-17), 75.7 (C-2), 46.6 (C-3), 26.1 (C-1), 25.2 (C-6), 24.0 (C-8), 22.2 (C-7) ppm.

HRMS (ESI) m/z: $[M+H]^+$ calcd for $C_{17}H_{20}NO^+$ 254.1540, found 254.1551.

IR (thin film): ṽ 3358 (s), 2937 (s), 2923 (s), 1617(s), 1598 (s), 1504 (s), 1381 (s), 1160 (s), 1087 (s) cm^{-1}.

Specific rotation: $[\alpha]_D^{23}$ = −16.6 (*c* 1, CH_2Cl_2).

The **enantiomeric ratio** of **3ar** was determined by SFC analysis (CHIRALPACK ID (25 cm), column temperature 25 °C, solvent CO_2/MeOH = 80/20, flow rate = 2.0 mL/min): t_R = 4.1 min (minor), t_R = 4.9 min (major).

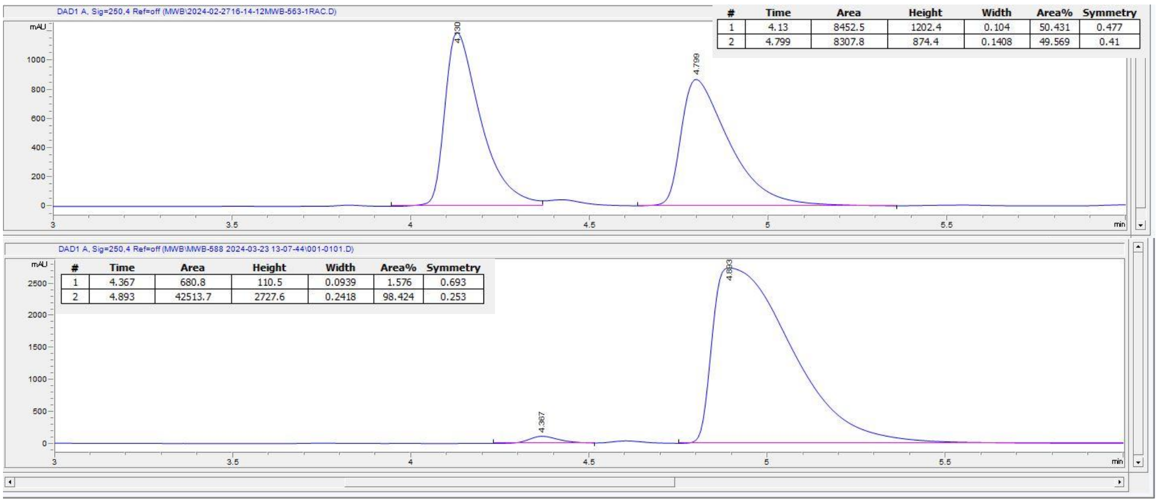

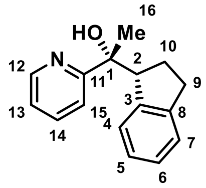

$C_{16}H_{17}NO$
M = 239.32

(*R*)-1-((*R*)-2,3-Dihydro-1*H*-inden-1-yl)-1-(pyridin-2-yl)ethan-1-ol (**3bp**, **GP 6**) was prepared from 1-(pyridin-2-yl)ethan-1-ol (**1b**, 12.3 mg, 0.10 mmol, 100 mol%) and indene (**2p**, 29.3 μL, 29.0 mg, 0.25 mmol, 250 mol%), using [Ir(cod)$_2$]BARF (6.36 mg, 5.0 μmol, 5.0 mol%), and **L10** ((*R*,*R*)-QuinoxP*, 1.67 mg, 5.0 μmol, 5.0 mol%) in H_2O (0.2 mL) at 110 °C for 5 d. Purification by flash column chromatography on silica gel (hexane/ethyl acetate from 100/1 to 9/1) afforded **3bp** (18.5 mg, 77% yield, 92% e.e., >20:1 d.r., >20:1 r.r.) as a colorless solid. Diastereomer and regiomer ratios (>20:1 d.r., >20:1 r.r.) were determined from the ^{1}H NMR spectrum of crude material.

R*f* = 0.2 (Hexane/EtOAc = 9/1).

M.**P**. 82–83 °C (Hexane/EtOAc).

^{1}H NMR (500 MHz, $CDCl_3$, 298 K): δ 8.53 (d, *J* = 4.9 Hz, 1H, H-12), 7.62–7.56 (m, 1H, H-14), 7.24–7.19 (m, 1H, H-13), 7.15–7.09 (m, 2H, H-6 and H-7), 7.02 (d, *J* = 8.0 Hz, 1H, H-15), 7.01–6.96 (m, 1H, H-5), 6.67 (d, *J* = 7.6 Hz, 1H, H-4), 5.22 (s, 1H, OH), 3.67 (dd, *J* = 8.6, 4.5 Hz, 1H, H-2), 2.67–2.59 (m, 1H, H-9), 2.45–2.36 (m, 1H, H-9), 2.25–2.17 (m, 1H, H-10), 2.16–2.06 (m, 1H, H-10), 1.64 (s, 3H, H-16) ppm.

^{13}C NMR (125 MHz, $CDCl_3$, 298 K): δ 163.9 (C-11), 147.3 (C-12), 146.1 (C-8), 143.1 (C-3), 136.6 (C-14), 127.0 (C-6), 125.7 (C-4 or C-5), 125.7 (C-4 or C-5), 124.5 (C-7), 122.2 (C-13), 120.3 (C-15), 76.1 (C-1), 56.7 (C-2), 31.4 (C-9), 27.4 (C-10), 26.8 (C-16) ppm.

HRMS (ESI) m/z: $[M+H]^+$ calcd for $C_{16}H_{18}NO^+$ 240.1383, found 240.1387.

IR (thin film): ṽ 3320 (br), 2941 (s), 1590 (s), 1454 (s), 1379 (s), 1151 (s), 1073 (s) cm^{-1}.

Specific rotation: $[\alpha]_D^{27}$ = +15.9 (*c* 1, CH_2Cl_2).

The **enantiomeric ratio** of **3bp** was determined by SFC analysis (CHIRALPACK AD (25 cm), column temperature 25 °C, solvent CO_2/MeOH = 90/10, flow rate = 2.0 mL/min): t_R = 6.6 min (major), t_R = 7.3 min (minor).

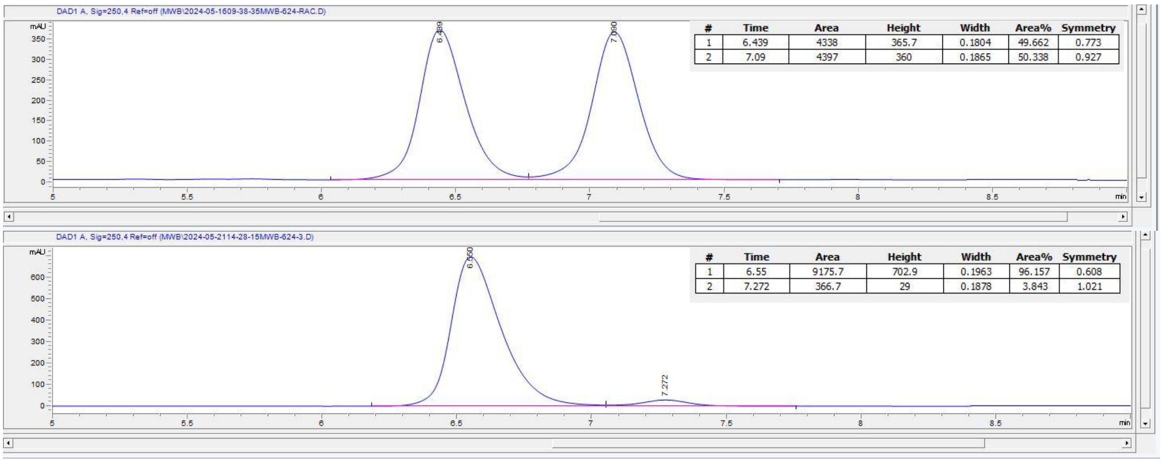

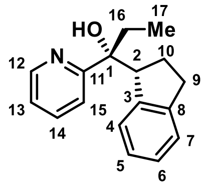

$C_{17}H_{19}NO$
M = 253.35

(*R*)-1-((*R*)-2,3-Dihydro-1*H*-inden-1-yl)-1-(pyridin-2-yl)propan-1-ol (**3cp**, **GP 6**) was prepared from 1-(pyridin-2-yl)propan-1-ol (**1c**, 13.7 mg, 0.10 mmol, 100 mol%) and indene (**2p**, 29.3 µL, 29.0 mg, 0.25 mmol, 250 mol%), using $[Ir(cod)_2]$BARF (12.7 mg, 10 µmol, 10 mol%), and **L10** ((*R*,*R*)-QuinoxP*, 3.34 mg, 10 µmol, 10 mol%) in H_2O (0.2 mL) at 110 °C for 5 d. Purification by flash column chromatography on silica gel (hexane/ethyl acetate from 100/1 to 19/1) afforded **3cp** (22.0 mg, 87% yield, 90% e.e., >20:1 d.r., >20:1 r.r.) as a colorless solid. Diastereomer and regiomer ratios (>20:1 d.r., >20:1 r.r.) were determined from the ^1H NMR spectrum of crude material.

R*f* = 0.2 (Hexane/EtOAc = 9/1).

M.P. 84–85 °C (Hexane/EtOAc).

^{1}H NMR (500 MHz, $CDCl_3$, 298 K): δ 8.52 (d, *J* = 4.8 Hz, 1H, H-12), 7.56–7.51 (m, 1H, H-14), 7.22–7.18 (m, 1H, H-13), 7.13–7.07 (m, 2H, H-6 and H-7), 7.00–6.95 (m, 1H, H-5), 6.83 (d, *J* = 8.0 Hz, 1H, H-15), 6.58 (d, *J* = 7.6 Hz, 1H, H-4), 5.45 (s, 1H, OH), 3.68 (dd, *J* = 8.7, 3.7 Hz, 1H, H-2), 2.61–2.53 (m, 1H, H-9), 2.31–2.10 (m, 4H, H-9, H-10 and H-16), 2.09–2.00 (m, 1H, H-16), 0.71 (t, *J* = 7.3 Hz, 3H, H-17) ppm.

^{13}C NMR (125 MHz, $CDCl_3$, 298 K): δ 162.0 (C-11), 147.1 (C-12), 146.4 (C-8), 143.1 (C-3), 136.3 (C-14), 126.9 (C-6), 125.6 (C-4 or C-5), 125.6 (C-4 or C-5), 124.6 (C-7), 122.0 (C-13), 120.7 (C-15), 78.4 (C-1), 56.1 (C-2), 32.1 (C-16), 31.4 (C-9), 27.2 (C-10), 7.8 (C-17) ppm.

HRMS (ESI) m/z: $[M+H]^+$ calcd for $C_{17}H_{20}NO^+$ 254.1539, found 254.1548.

IR (thin film): ṽ 3323 (s), 2962 (s), 2933 (s), 1592 (s), 1433 (s), 1388 (s), 1150 (s), 1076 (s) cm^{-1}.

Specific rotation: $[\alpha]_D^{30}$ = +3.2 (*c* 1, CH_2Cl_2).

The **enantiomeric ratio** of **3cp** was determined by SFC analysis (CHIRALPACK AD (25 cm), column temperature 25 °C, solvent CO_2/MeOH = 90/10, flow rate = 2.0 mL/min): t_R = 5.3 min (major), t_R = 6.2 min (minor).

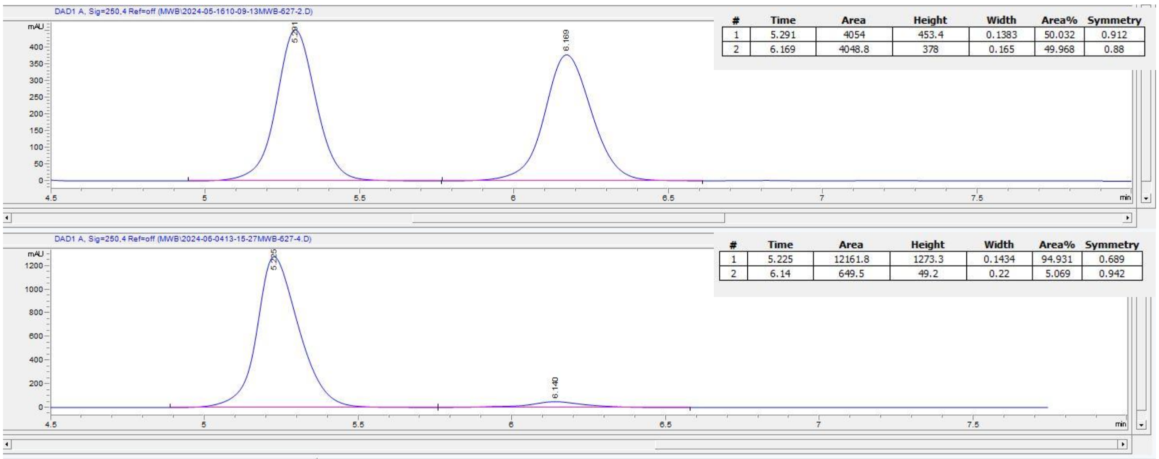

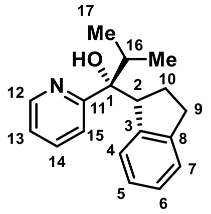

$C_{18}H_{21}NO$
M = 267.37

(*R*)-1-((*R*)-2,3-Dihydro-1*H*-inden-1-yl)-2-methyl-1-(pyridin-2-yl)propan-1-ol (**3dp**, **GP 6**) was prepared from 2-methyl-1-(pyridin-2-yl)propan-1-ol (**1d**, 15.1 mg, 0.10 mmol, 100 mol%)

and indene (**2p**, 29.3 μL, 29.0 mg, 0.25 mmol, 250 mol%), using [Ir(cod)$_2$]BARF (12.7 mg, 10 μmol, 10 mol%), and **L10** ((*R*,*R*)-QuinoxP, 3.34 mg, 10 μmol, 10 mol%) in H_2O (0.2 mL) at 110 °C for 5 d. Purification by flash column chromatography on silica gel (hexane/ethyl acetate from 100/1 to 19/1) afforded **3dp** (19.5 mg, 73% yield, 81% e.e., >20:1 d.r., >20:1 r.r.) as a colorless oil. Diastereomer and regiomer ratios (>20:1 d.r., >20:1 r.r.) were determined from the ^{1}H NMR spectrum of crude material.

R$_f$ = 0.5 (Hexane/EtOAc = 20/1).

^{1}H NMR (500 MHz, $CDCl_3$, 298 K): δ 8.50 (d, *J* = 4.6 Hz, 1H, H-12), 7.35–7.31 (m, 1H, H-14), 7.17–7.07 (m, 4H, H-4, H-5, H-6 and H-13), 7.02 (d, *J* = 6.2 Hz, 1H, H-7), 6.29 (d, *J* = 8.1 Hz, 1H, H-15), 5.86 (s, 1H, OH), 3.97 (dd, *J* = 7.6, 4.3 Hz, 1H, H-2), 2.49–2.37 (m, 2H, H-9 and H-16), 2.15–2.04 (m, 2H, H-10), 1.55–1.45 (m, 1H, H-9), 1.25 (d, *J* = 6.6 Hz, 3H, H-17), 0.63 (d, *J* = 6.7 Hz, 3H, H-17) ppm.

^{13}C NMR (125 MHz, $CDCl_3$, 298 K): δ 161.0 (C-11), 147.0 (C-8), 146.7 (C-12), 143.1 (C-3), 135.7 (C-14), 127.0 (C-6), 126.0 (C-5), 125.5 (C-4), 124.7 (C-7), 122.0 (C-13), 121.0 (C-15), 79.5 (C-1), 52.4 (C-2), 33.9 (C-16), 30.8 (C-9), 26.7 (C-10), 17.5 (C-17), 17.4 (C-17) ppm.

HRMS (ESI) m/z: $[M+H]^+$ calcd for $C_{18}H_{22}NO^+$ 268.1696, found 268.1699.

IR (thin film): ṽ 3366 (br), 2967 (s), 2935 (s), 1591 (s), 1433 (s), 1383 (s), 1154 (s), 1008 (s) cm^{-1}.

Specific rotation: $[\alpha]_D^{29}$ = –77.0 (*c* 1, CH_2Cl_2).

The **enantiomeric ratio** of **3dp** was determined by SFC analysis (CHIRALPACK AD (25 cm), column temperature 25 °C, solvent CO_2/MeOH = 90/10, flow rate = 2.0 mL/min): t_R = 4.1 min (major), t_R = 5.3 min (minor).

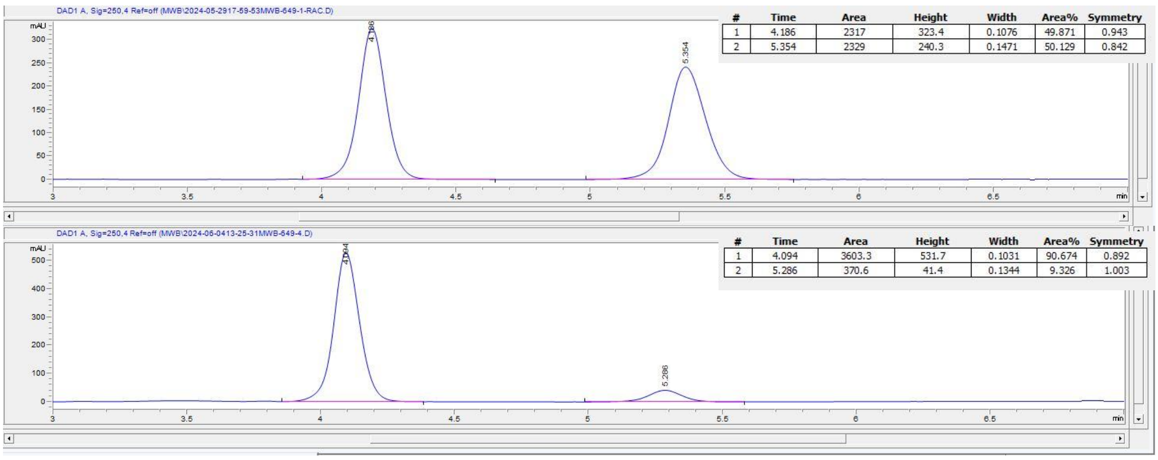

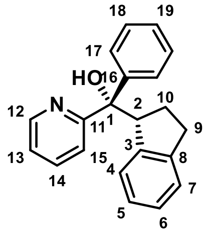

$C_{21}H_{19}NO$
M = 301.39

(*S*)-((*R*)-2,3-Dihydro-1*H*-inden-1-yl)(phenyl)(pyridin-2-yl)methanol (**3ep**, **GP 6**) was prepared from phenyl(pyridin-2-yl)methanol (**1e**, 18.5 mg, 0.10 mmol, 100 mol%) and indene (**2p**, 29.3 µL, 29.0 mg, 0.25 mmol, 250 mol%), using [Ir(cod)$_2$]BARF (6.36 mg, 5.0 µmol, 5.0 mol%), and **L1** ((*R*)-BINAP, 3.11 mg, 5.0 µmol, 5.0 mol%) in H_2O (0.2 mL) at 110 °C for 5 d. Purification by flash column chromatography on silica gel (hexane/ethyl acetate from 100/1 to 19/1) afforded **3ep** (22.5 mg, 75% yield, 95% e.e., >20:1 d.r., >20:1 r.r.) as a colorless solid. Diastereomer and regiomer ratios (>20:1 d.r., >20:1 r.r.) were determined from the ^{1}H NMR spectrum of crude material.

R*f* = 0.2 (Hexane/EtOAc = 9/1).

M.**P**. 109–110 °C (Hexane/EtOAc).

^{1}H NMR (500 MHz, $CDCl_3$, 298 K): δ 8.50 (d, *J* = 5.8 Hz, 1H, H-12), 7.73 (d, *J* = 7.8 Hz, 2H, H-17), 7.72–7.67 (m, 1H, H-14), 7.56 (d, *J* = 8.0 Hz, 1H, H-15), 7.40–7.35 (m, 2H, H-18), 7.28–7.24 (m, 1H, H-19), 7.24–7.19 (m, 2H, H-7 and H-13), 7.14–7.09 (m, 1H, H-6), 6.83–6.78 (m, 1H, H-5), 5.80 (d, *J* = 7.6 Hz, 1H, H-4), 5.37 (s, 1H, OH), 4.59 (dd, *J* = 8.5, 5.1 Hz, 1H, H-2), 3.13–3.04 (m, 1H, H-9), 2.84–2.76 (m, 1H, H-9), 2.26–2.13 (m, 2H, H-10) ppm.

^{13}C NMR (125 MHz, $CDCl_3$, 298 K): δ 163.3 (C-11), 147.2 (C-12), 147.0 (C-8), 146.3 (C-16), 142.1 (C-3), 137.0 (C-14), 128.4 (C-18), 127.0 (C-6 or C-19), 126.9 (C-6 or C-19), 126.5 (C-17), 125.3 (C-5), 125.0 (C-4), 124.7 (C-7), 122.2 (C-13), 121.6 (C-15), 80.6 (C-1), 54.1 (C-2), 32.0 (C-9), 28.6 (C-10) ppm.

HRMS (ESI) m/z: $[M+Na]^+$ calcd for $C_{21}H_{29}NNaO^+$ 324.1359, found 324.1364.

IR (thin film): ṽ 3324 (br), 2958 (s), 2936 (s), 1584 (s), 1431 (s), 1385 (s), 1154 (s), 1058 (s) cm^{-1}.

Specific rotation: $[\alpha]_D^{24}$ = +113.5 (*c* 1, CH_2Cl_2).

The **enantiomeric ratio** of **3ep** was determined by SFC analysis (CHIRALPACK SC (25 cm), column temperature 25 °C, solvent CO_2/MeOH = 90/10, flow rate = 2.0 mL/min): t_R = 6.7 min (minor), t_R = 7.5 min (major).

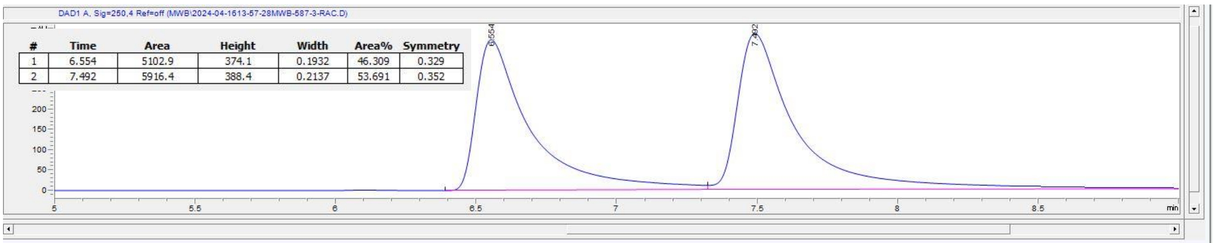

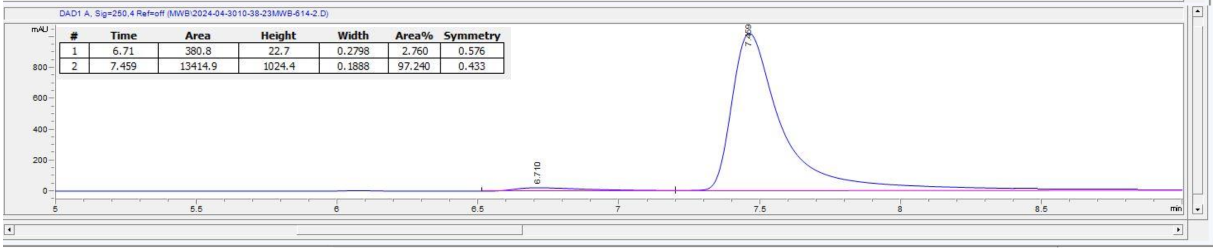

Crystal **3ep** was obtained through recrystallization in the solution of DCM and methanol at room temperature. The absolute configuration of **3ep** was confirmed unambiguously by **X-ray diffraction analysis**, and other compounds were assigned by analogy. CCDC 2480110 contains the supplementary crystallographic data for this compound.

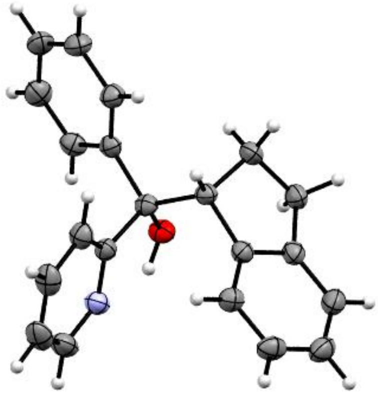

Figure S2. Molecular structure of **3ep**. Thermal ellipsoids represent 50% probability level.

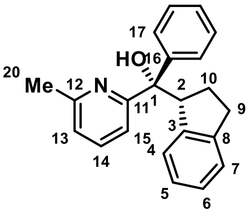

$C_{22}H_{21}NO$
M = 315.42

(*S*)-((*R*)-2,3-Dihydro-1*H*-inden-1-yl)(6-methylpyridin-2-yl)(phenyl)methanol (**3fp**, **GP 6**) was prepared from (6-methylpyridin-2-yl)(phenyl)methanol (**1f**, 19.9 mg, 0.10 mmol, 100 mol%) and indene (**2p**, 29.3 µL, 29.0 mg, 0.25 mmol, 250 mol%), using [Ir(cod)$_2$]BARF (6.36 mg, 5.0 µmol, 5.0 mol%), and **L1** ((*R*)-BINAP, 3.11 mg, 5.0 µmol, 5.0 mol%) in H_2O (0.2 mL) at 110 °C for 3 d. Purification by flash column chromatography on silica gel (hexane/ethyl

acetate from 100/1 to 19/1) afforded **3fp** (23.7 mg, 75% yield, 95% e.e., >20:1 d.r., >20:1 r.r.) as a colorless solid. Diastereomer and regiomer ratios (15:1 d.r., >20:1 r.r.) were determined from the ^{1}H NMR spectrum of crude material.

R*f* = 0.5 (Hexane/EtOAc = 15/1).

M.**P**. 112–113 °C (Hexane/EtOAc).

^{1}H NMR (500 MHz, $CDCl_3$, 298 K): δ 7.73 (d, *J* = 7.0 Hz, 2H, H-17), 7.60–7.56 (m, 1H, H-14), 7.40–7.35 (m, 2H, H-18), 7.34 (d, *J* = 7.9 Hz, 1H, H-15), 7.28–7.24 (m, 1H, H-19), 7.22 (d, *J* = 7.5 Hz, 1H, H-7), 7.13–7.09 (m, 1H, H-6), 7.06 (d, *J* = 7.5 Hz, 1H, H-13), 6.83–6.78 (m, 1H, H-5), 5.92 (s, 1H, OH), 5.79 (d, *J* = 7.6 Hz, 1H, H-4), 4.51 (dd, *J* = 8.2, 5.2 Hz, 1H, H-2), 3.13–3.05 (m, 1H, H-9), 2.83–2.76 (m, 1H, H-9), 2.50 (s, 3H, H-20), 2.26–2.13 (m, 2H, H-10) ppm.

^{13}C NMR (125 MHz, $CDCl_3$, 298 K): δ 162.1 (C-11), 156.1 (C-12), 146.9 (C-8 or C-16), 146.7 (C-8 or C-16), 142.4 (C-3), 137.2 (C-14), 128.3 (C-18), 126.9 (C-6 or C-19), 126.8 (C-6 or C-19), 126.6 (C-17), 125.2 (C-5), 125.1 (C-4), 124.6 (C-7), 121.5 (C-13), 118.5 (C-15), 80.1 (C-1), 54.2 (C-2), 32.0 (C-9), 28.7 (C-10), 24.3 (C-20) ppm.

HRMS (ESI) m/z: $[M+H]^+$ calcd for $C_{22}H_{22}NO^+$ 316.1696, found 316.1702.

IR (thin film): ṽ 3311 (br), 2958 (s), 2939 (s), 1592 (s), 1456 (s), 1375 (s), 1186 (s), 1070 (s) cm^{-1}.

Specific rotation: $[\alpha]_D^{27}$ = +107.7 (*c* 1, CH_2Cl_2).

The **enantiomeric ratio** of **3fp** was determined by SFC analysis (CHIRALPACK IE (25 cm), column temperature 25 °C, solvent CO_2/MeOH = 90/10, flow rate = 2.0 mL/min): t_R = 8.2 min (minor), t_R = 8.8 min (major).

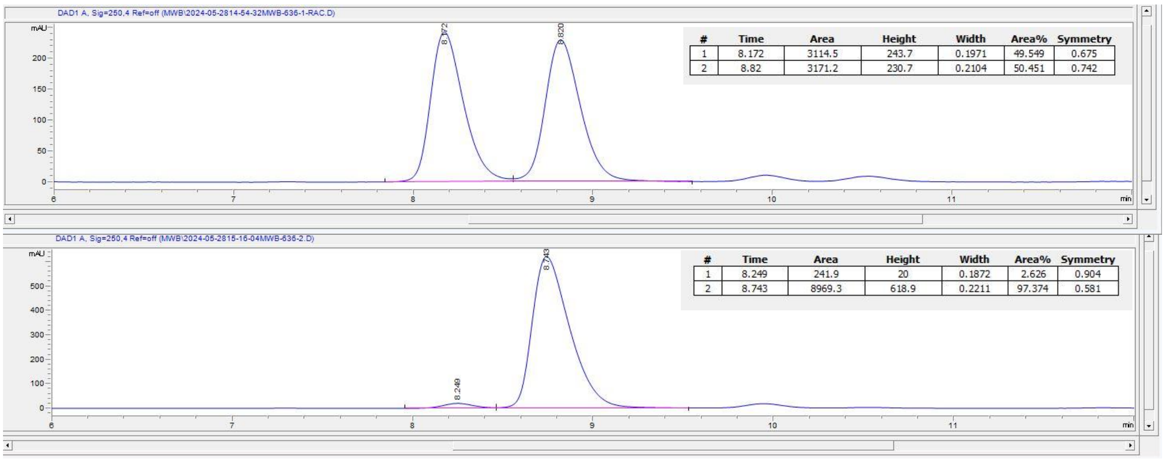

Crystal **3fp** was obtained through recrystallization in the solution of DCM and methanol at room temperature. The absolute configuration of **3fp** was confirmed unambiguously by **X-ray diffraction analysis**, and other compounds were assigned by analogy. CCDC 2480112 contains the supplementary crystallographic data for this compound.

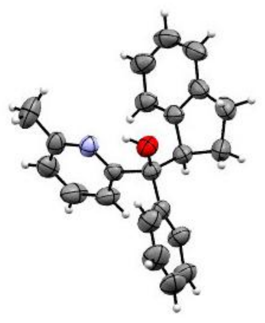

Figure S3. Molecular structure of **3fp**. Thermal ellipsoids represent 50% probability level.

Characteristic signals for diastereomer **3fp'**

^{1}H NMR (500 MHz, $CDCl_3$, 298 K): 6.37 (d, *J* = 7.7 Hz, 1H), 2.56 (s, 3H).

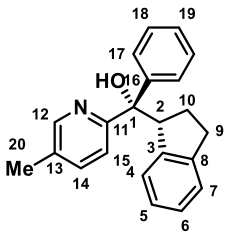

$C_{22}H_{21}NO$
M = 315.42

(*S*)-((*R*)-2,3-Dihydro-1*H*-inden-1-yl)(5-methylpyridin-2-yl)(phenyl)methanol (**3gp**, **GP 6**) was prepared from (5-methylpyridin-2-yl)(phenyl)methanol (**1g**, 19.9 mg, 0.10 mmol, 100 mol%) and indene (**2p**, 29.3 μL, 29.0 mg, 0.25 mmol, 250 mol%), using [Ir(cod)$_2$]BARF (12.7 mg, 10 μmol, 10 mol%), and **L10** ((*R*,*R*)-QuinoxP*, 3.34 mg, 10 μmol, 10 mol%) in H_2O (0.2 mL) at 110 °C for 3 d. Purification by flash column chromatography on silica gel (hexane/ethyl acetate from 100/1 to 19/1) afforded **3gp** (25.5 mg, 81% yield, 95% e.e., >20:1 d.r., >20:1 r.r.) as a colorless solid. Diastereomer and regiomer ratios (>20:1 d.r., >20:1 r.r.) were determined from the ^{1}H NMR spectrum of crude material.

R*f* = 0.5 (Hexane/EtOAc = 9/1).

M.P. 85–86 °C (Hexane/EtOAc).

^{1}H NMR (500 MHz, $CDCl_3$, 298 K): δ 8.32 (s, 1H, H-12), 7.71 (d, *J* = 7.7 Hz, 2H, H-17), 7.50 (d, *J* = 8.1 Hz, 1H, H-14), 7.45 (d, *J* = 8.2 Hz, 1H, H-15), 7.39–7.34 (m, 2H, H-18), 7.25 (d, *J* =

8.0 Hz, 1H, H-19), 7.22 (d, *J* = 7.4 Hz, 1H, H-7), 7.14–7.09 (m, 1H, H-6), 6.85–6.80 (m, 1H, H-5), 5.86 (d, *J* = 7.7 Hz, 1H, H-4), 5.38 (s, 1H, OH), 4.58 (dd, *J* = 8.4, 5.4 Hz, 1H, H-2), 3.11–3.03 (m, 1H, H-9), 2.83–2.75 (m, 1H, H-9), 2.35 (s, 3H, H-20), 2.24–2.11 (m, 2H, H-10) ppm.

^{13}C NMR (125 MHz, $CDCl_3$, 298 K): δ 160.5 (C-11), 147.4 (C-12), 147.0 (C-8), 146.6 (C-16), 142.3 (C-3), 137.7 (C-14), 131.7 (C-13), 128.3 (C-18), 126.9 (C-6 or C-19), 126.8 (C-6 or C-19), 126.4 (C-17), 125.3 (C-5), 125.0 (C-4), 124.7 (C-7), 121.0 (C-15), 80.3 (C-1), 54.0 (C-2), 31.9 (C-9), 28.6 (C-10), 18.2 (C-20) ppm.

HRMS (ESI) m/z: $[M+H]^+$ calcd for $C_{22}H_{22}NO^+$ 316.1696, found 316.1699.

IR (thin film): $\tilde{\nu}$ 3378 (br), 2948 (s), 1597 (s), 1478 (s), 1376 (s), 1158 (s), 1069 (s) cm^{-1}.

Specific rotation: $[\alpha]_D^{27}$ = +116.2 (*c* 1, CH_2Cl_2).

The **enantiomeric ratio** of **3gp** was determined by SFC analysis (CHIRALPACK AD (25 cm), column temperature 25 °C, solvent CO_2/MeOH = 90/10, flow rate = 2.0 mL/min): t_R = 12.4 min (minor), t_R = 16.1 min (major).

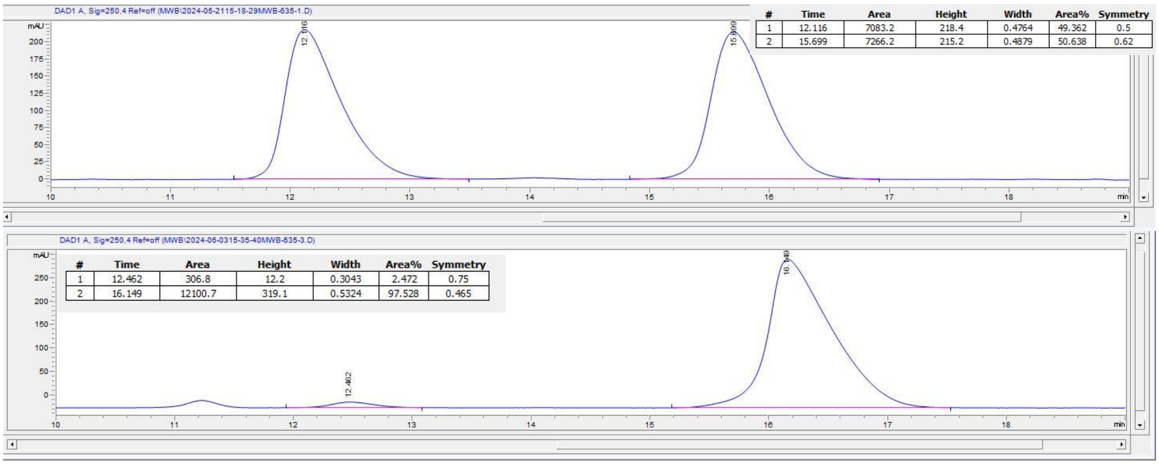

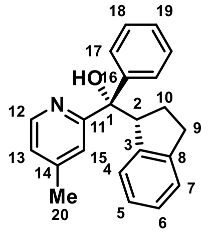

$C_{22}H_{21}NO$
M = 315.42

(*S*)-((*R*)-2,3-Dihydro-1*H*-inden-1-yl)(4-methylpyridin-2-yl)(phenyl)methanol (**3hp**, **GP 6**) was prepared from (4-methylpyridin-2-yl)(phenyl)methanol (**1h**, 19.9 mg, 0.10 mmol, 100 mol%) and indene (**2p**, 29.3 µL, 29.0 mg, 0.25 mmol, 250 mol%), using [Ir(cod)$_2$]BARF (6.36 mg, 5.0 µmol, 5.0 mol%), and **L1** ((*R*)-BINAP, 3.11 mg, 5.0 µmol, 5.0 mol%) in H_2O (0.2 mL)

at 110 °C for 3 d. Purification by flash column chromatography on silica gel (hexane/ethyl acetate from 100/1 to 19/1) afforded **3hp** (22.2 mg, 70% yield, 92% e.e., >20:1 d.r., >20:1 r.r.) as a colorless solid. Diastereomer and regiomer ratios (>20:1 d.r., >20:1 r.r.) were determined from the ^{1}H NMR spectrum of crude material.

$\mathbf{R_f}$ = 0.6 (Hexane/EtOAc = 15/1).

M.P. 90–91 °C (Hexane/EtOAc).

^{1}H NMR (500 MHz, $CDCl_3$, 298 K): δ 8.35 (d, J = 5.1 Hz,1H, H-12), 7.74 (d, J = 7.4 Hz, 2H, H-17), 7.40–7.35 (m, 3H, H-15 and H-18), 7.28–7.24 (m, 1H, H-19), 7.22 (d, J = 7.5 Hz, 1H, H-7), 7.14–7.09 (m, 1H, H-6), 7.03 (d, J = 5.1 Hz, 1H, H-13), 6.84–6.80 (m, 1H, H-5), 5.82 (d, J = 7.7 Hz, 1H, H-4), 5.41 (s, 1H, OH), 4.59 (dd, J = 8.4, 5.3 Hz, 1H, H-2), 3.11–3.03 (m, 1H, H-9), 2.83–2.76 (m, 1H, H-9), 2.36 (s, 3H, H-20), 2.24–2.11 (m, 2H, H-10) ppm.

^{13}C NMR (125 MHz, $CDCl_3$, 298 K): δ 163.2 (C-11), 148.1 (C-14), 147.0 (C-8), 146.8 (C-12), 146.5 (C-16), 142.2 (C-3), 128.4 (C-18), 127.0 (C-6 or C-19), 126.8 (C-6 or C-19), 126.5 (C-17), 125.3 (C-5), 125.0 (C-4), 124.7 (C-7), 123.4 (C-13), 122.2 (C-15), 80.3 (C-1), 54.1 (C-2), 31.9 (C-9), 28.6 (C-10), 21.5 (C-20) ppm.

HRMS (ESI) m/z: $[M+H]^+$ calcd for $C_{22}H_{22}NO^+$ 316.1696, found 316.1701.

IR (thin film): ṽ 3271 (br), 2962 (s), 2954 (s), 1604 (s), 1444 (s), 1383 (s), 1188 (s), 1069 (s) cm^{-1}.

Specific rotation: $[\alpha]_D^{28}$ = +92.1 (c 1, CH_2Cl_2).

The **enantiomeric ratio** of **3hp** was determined by SFC analysis (CHIRALPACK AD (25 cm), column temperature 25 °C, solvent CO_2/MeOH = 90/10, flow rate = 2.0 mL/min): t_R = 9.0 min (minor), t_R = 10.9 min (major).

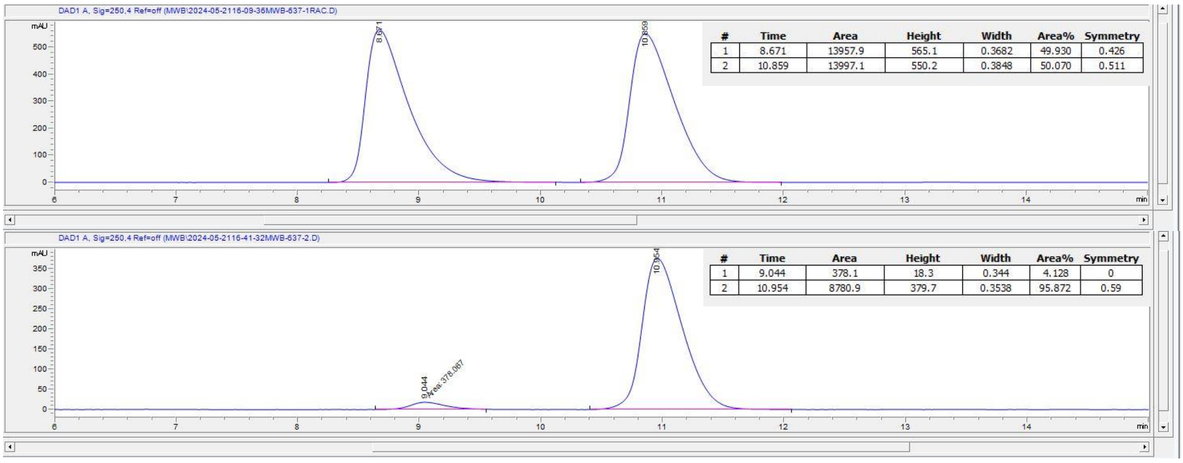

#	Time	Area	Height	Width	Area%	Symmetry
1	8.671	13957.9	565.1	0.3682	49.930	0.426
2	10.859	13997.1	550.2	0.3848	50.070	0.511

#	Time	Area	Height	Width	Area%	Symmetry
1	9.044	378.1	18.3	0.344	4.128	0
2	10.954	8780.9	379.7	0.3538	95.872	0.59

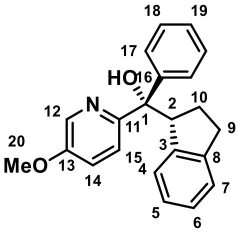

$C_{22}H_{21}NO_2$
M = 331.42

(*S*)-((*R*)-2,3-Dihydro-1*H*-inden-1-yl)(5-methoxypyridin-2-yl)(phenyl)methanol (**3ip**, **GP 6**) was prepared from (5-methoxypyridin-2-yl)(phenyl)methanol (**1i**, 21.5 mg, 0.10 mmol, 100 mol%) and indene (**2p**, 29.3 µL, 29.0 mg, 0.25 mmol, 250 mol%), using [Ir(cod)$_2$]BARF (12.7 mg, 10 µmol, 10 mol%), and **L10** ((*R*,*R*)-QuinoxP*, 3.34 mg, 10 µmol, 10 mol%) in H_2O (0.2 mL) at 110 °C for 5 d. Purification by flash column chromatography on silica gel (hexane/ethyl acetate from 100/1 to 19/1) afforded **3ip** (21.6 mg, 65% yield, 92% e.e., >20:1 d.r., >20:1 r.r.) as a colorless solid. Diastereomer and regiomer ratios (>20:1 d.r., >20:1 r.r.) were determined from the ^{1}H NMR spectrum of crude material.

R*f* = 0.3 (Hexane/EtOAc = 9/1).

M.**P**. 90–91 °C (Hexane/EtOAc).

^{1}H NMR (500 MHz, $CDCl_3$, 298 K): δ 8.18 (d, *J* = 2.9 Hz, 1H, H-12), 7.68 (d, *J* = 7.3 Hz, 2H, H-17), 7.44 (d, *J* = 8.7 Hz, 1H, H-15), 7.37–7.32 (m, 2H, H-18), 7.25–7.18 (m, 3H, H-7, H-14 and H-19), 7.13–7.08 (m, 1H, H-6), 6.85–6.80 (m, 1H, H-5), 5.87 (d, *J* = 7.6 Hz, 1H, H-4), 5.04 (s, 1H, OH), 4.57 (dd, *J* = 8.5, 5.3 Hz, 1H, H-2), 3.87 (s, 3H, H-20), 3.07–2.99 (m, 1H, H-9), 2.81–2.73 (m, 1H, H-9), 2.20–2.07 (m, 2H, H-10) ppm.

^{13}C NMR (125 MHz, $CDCl_3$, 298 K): δ 155.6 (C-11), 154.7 (C-13), 147.0 (C-8), 146.6 (C-16), 142.3 (C-3), 133.9 (C-12), 128.3 (C-18), 127.0 (C-6 or C-19), 126.8 (C-6 or C-19), 126.3 (C-17), 125.4 (C-5), 125.1 (C-4), 124.7 (C-7), 122.4 (C-14), 121.8 (C-15), 80.3 (C-1), 55.9 (C-20), 54.2 (C-2), 31.9 (C-9), 28.5 (C-10) ppm.

HRMS (ESI) m/z: $[M+H]^+$ calcd for $C_{22}H_{22}NO_2^+$ 332.1645, found 332.1646.

IR (thin film): ṽ 3347 (s), 2938 (s), 2838 (s), 1571 (s), 1481 (s), 1376 (s), 1273 (s), 1025 (s) cm^{-1}.

Specific rotation: $[\alpha]_D^{29}$ = +92.1 (*c* 1, CH_2Cl_2).

The **enantiomeric ratio** of **3ip** was determined by SFC analysis (CHIRALPACK AD (25 cm), column temperature 25 °C, solvent CO_2/MeOH = 80/20, flow rate = 2.0 mL/min): t_R = 7.8 min (minor), t_R = 9.4 min (major).

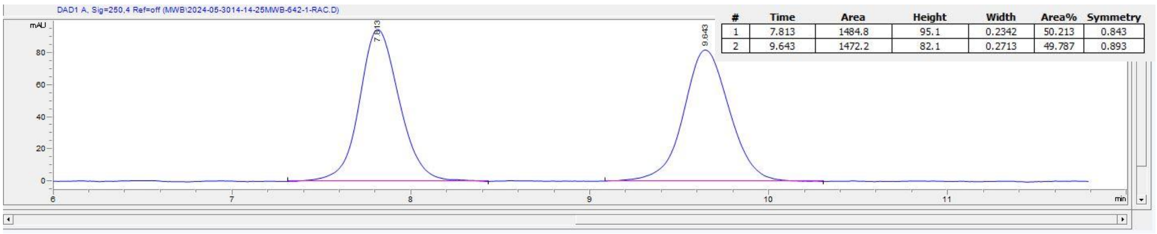

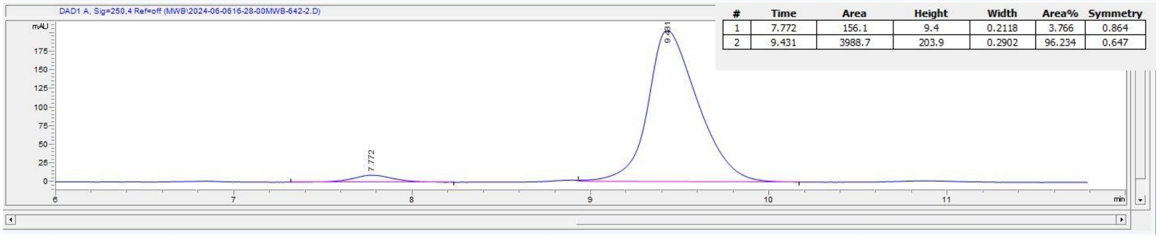

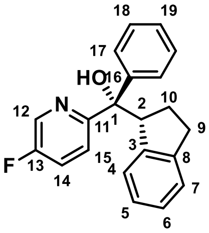

$C_{21}H_{18}FNO$
M = 319.38

(*S*)-((*R*)-2,3-Dihydro-1*H*-inden-1-yl)(5-fluoropyridin-2-yl)(phenyl)methanol (**3jp**, **GP 6**) was prepared from (5-fluoropyridin-2-yl)(phenyl)methanol (**1j**, 20.3 mg, 0.10 mmol, 100 mol%) and indene (**2p**, 29.3 µL, 29.0 mg, 0.25 mmol, 250 mol%), using [Ir(cod)$_2$]BARF (12.7 mg, 10 µmol, 10 mol%), and **L10** ((*R*,*R*)-QuinoxP*, 3.34 mg, 10 µmol, 10 mol%) in H_2O (0.2 mL) at 110 °C for 3 d. Purification by flash column chromatography on silica gel (hexane/ethyl acetate from 100/1 to 19/1) afforded **3jp** (26.2 mg, 82% yield, 95% e.e., >20:1 d.r., >20:1 r.r.) as a colorless solid. Diastereomer and regiomer ratios (>20:1 d.r., >20:1 r.r.) were determined from the ^{1}H NMR spectrum of crude material.

R_f = 0.5 (Hexane/EtOAc = 19/1).

M.P. 98–99 °C (Hexane/EtOAc).

^{1}H NMR (500 MHz, $CDCl_3$, 298 K): δ 8.38 (d, *J* = 2.7 Hz, 1H, H-12), 7.70 (d, *J* = 7.5 Hz, 2H, H-17), 7.56 (dd, *J* = 8.9, 4.3 Hz, 1H, H-15), 7.43–7.38 (m, 1H, H-14), 7.39–7.34 (m, 2H, H-18), 7.28–7.25 (m, 1H, H-19), 7.25–7.21 (m, 1H, H-7), 7.16–7.11 (m, 1H, H-6), 6.88–6.83 (m, 1H, H-5), 5.88 (d, *J* = 7.7 Hz, 1H, H-4), 4.66 (dd, *J* = 8.7, 5.1 Hz, 1H, H-2), 4.54 (s, 1H, OH), 3.09–3.01 (m, 1H, H-9), 2.83–2.75 (m, 1H, H-9), 2.20–2.05 (m, 2H, H-10) ppm.

^{13}C NMR (125 MHz, $CDCl_3$, 298 K): δ 159.8 (d, *J* = 3.8 Hz, C-11), 158.6 (d, *J* = 253.8 Hz, C-13), 147.1 (C-8), 145.8 (C-16), 141.8 (C-3), 135.4 (d, *J* = 23.8 Hz, C-12), 128.4 (C-18), 127.2 (C-6 or C-19), 127.0 (C-6 or C-19), 126.3 (C-17), 125.6 (C-5), 125.0 (C-4), 125.0 (C-7), 124.0

(d, J = 18.8 Hz, C-14), 122.5 (d, J = 3.8 Hz, C-15), 80.7 (C-1), 54.1 (C-2), 31.9 (C-9), 28.5 (C-10) ppm.

^{19}F NMR (471 MHz, $CDCl_3$, 298 K): δ –129.7—129.8 ppm.

HRMS (ESI) m/z: $[M+Na]^+$ calcd for $C_{21}H_{18}FNNaO^+$ 342.1265, found 342.1271.

IR (thin film): ṽ 3373 (s), 2969 (s), 2850 (s), 1582 (s), 1477 (s), 1377 (s), 1231 (s), 1068 (s) cm^{-1}.

Specific rotation: $[\alpha]_D^{28}$ = +73.3 (c 1, CH_2Cl_2).

The **enantiomeric ratio** of **3jp** was determined by SFC analysis (CHIRALPACK AD (25 cm), column temperature 25 °C, solvent CO_2/MeOH = 90/10, flow rate = 2.0 mL/min): t_R = 9.5 min (minor), t_R = 10.9 min (major).

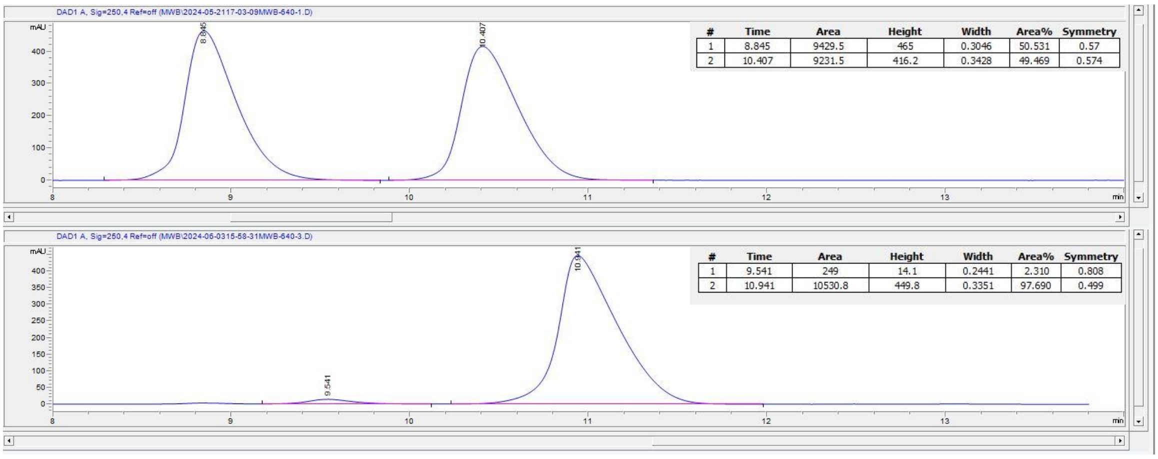

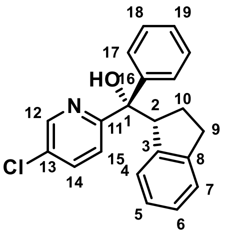

$C_{21}H_{18}ClNO$
M = 335.83

(*S*)-(5-Chloropyridin-2-yl)((*R*)-2,3-dihydro-1*H*-inden-1-yl)(phenyl)methanol (**3kp**, **GP 6**) was prepared from (5-chloropyridin-2-yl)(phenyl)methanol (**1k**, 22.0 mg, 0.10 mmol, 100 mol%) and indene (**2p**, 29.3 μL, 29.0 mg, 0.25 mmol, 250 mol%), using $[Ir(cod)_2]$BARF (12.7 mg, 10 μmol, 10 mol%), and **L10** ((*R*,*R*)-QuinoxP*, 3.34 mg, 10 μmol, 10 mol%) in H_2O (0.2 mL) at 110 °C for 3 d. Purification by flash column chromatography on silica gel (hexane/ethyl acetate from 100/1 to 19/1) afforded **3kp** (30.7 mg, 91% yield, 95% e.e., >20:1 d.r., >20:1 r.r.)

as a colorless oil. Diastereomer and regiomer ratios (>20:1 d.r., >20:1 r.r.) were determined from the ^{1}H NMR spectrum of crude material.

R$_f$ = 0.5 (Hexane/EtOAc = 15/1).

^{1}H NMR (500 MHz, $CDCl_3$, 298 K): δ 8.50 (d, *J* = 2.5 Hz, 1H, H-12), 7.72–7.68 (m, 2H, H-17), 7.65 (dd, *J* = 8.5, 2.5 Hz, 1H, H-14), 7.53 (d, *J* = 8.5 Hz, 1H, H-15), 7.39–7.34 (m, 2H, H-18), 7.28–7.25 (m, 1H, H-19), 7.25–7.22 (m, 1H, H-7), 7.16–7.12 (m, 1H, H-6), 6.90–6.85 (m, 1H, H-5), 5.94 (d, *J* = 7.6 Hz, 1H, H-4), 4.68 (dd, *J* = 8.6, 5.2 Hz, 1H, H-2), 4.43 (s, 1H, OH), 3.09–3.01 (m, 1H, H-9), 2.84–2.76 (m, 1H, H-9), 2.21–2.05 (m, 2H, H-10) ppm.

^{13}C NMR (125 MHz, $CDCl_3$, 298 K): δ 162.3 (C-11), 147.1 (C-8), 146.4 (C-12), 145.6 (C-16), 141.7 (C-3), 136.7 (C-14), 130.6 (C-13), 128.4 (C-18), 127.3 (C-6 or C-19), 127.1 (C-6 or C-19), 126.3 (C-17), 125.7 (C-5), 125.0 (C-4), 124.9 (C-7), 122.4 (C-15), 80.7 (C-1), 53.9 (C-2), 31.9 (C-9), 28.4 (C-10) ppm.

HRMS (ESI) m/z: $[M+H]^+$ calcd for $C_{21}H_{18}{}^{35}ClNO^+$ 336.1150, found 336.1149.

IR (thin film): ṽ 3384 (br), 2986 (s), 2847 (s), 1558 (s), 1446 (s), 1366 (s), 1109 (s), 1013 (s) cm^{-1}.

Specific rotation: $[\alpha]_D^{30}$ = +92.7 (*c* 1, CH_2Cl_2).

The **enantiomeric ratio** of **3kp** was determined by SFC analysis (CHIRALPACK IE (25 cm), column temperature 25 °C, solvent CO_2/MeOH = 90/10, flow rate = 2.0 mL/min): t_R = 9.4 min (minor), t_R = 9.8 min (major).

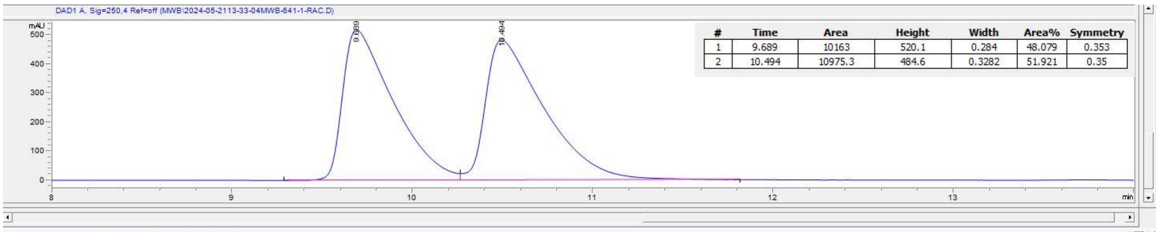

#	Time	Area	Height	Width	Area%	Symmetry
1	9.689	10163	520.1	0.284	48.079	0.353
2	10.494	10975.3	484.6	0.3282	51.921	0.35

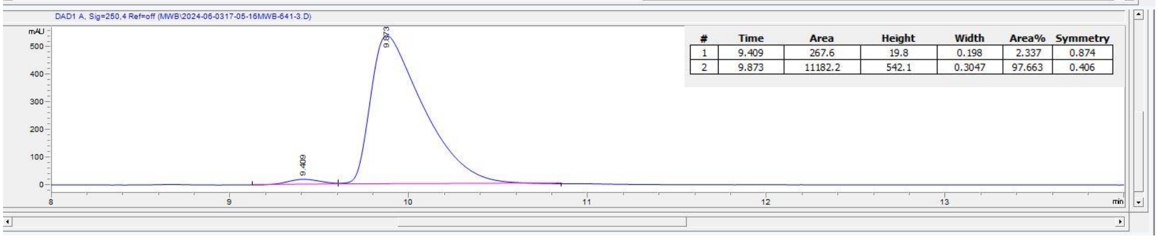

#	Time	Area	Height	Width	Area%	Symmetry
1	9.409	267.6	19.8	0.198	2.337	0.874
2	9.873	11182.2	542.1	0.3047	97.663	0.406

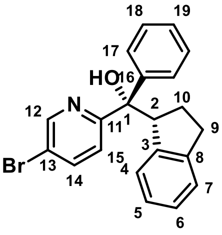

$C_{21}H_{18}BrNO$
M = 380.29

(*S*)-(5-Bromopyridin-2-yl)((*R*)-2,3-dihydro-1*H*-inden-1-yl)(phenyl)methanol (**3lp**, **GP 6**) was prepared from (5-bromopyridin-2-yl)(phenyl)methanol (**1l**, 26.4 mg, 0.10 mmol, 100 mol%) and indene (**2p**, 29.3 μL, 29.0 mg, 0.25 mmol, 250 mol%), using [Ir(cod)$_2$]BARF (12.7 mg, 10 μmol, 10 mol%), and **L10** ((*R*,*R*)-QuinoxP*, 3.34 mg, 10 μmol, 10 mol%) in H_2O (0.2 mL) at 110 °C for 3 d. Purification by flash column chromatography on silica gel (hexane/ethyl acetate from 100/1 to 19/1) afforded **3lp** (31.5 mg, 83% yield, 95% e.e., >20:1 d.r., >20:1 r.r.) as a colorless solid. Diastereomer and regiomer ratios (>20:1 d.r., >20:1 r.r.) were determined from the ^{1}H NMR spectrum of crude material.

R*f* = 0.2 (Hexane/EtOAc = 9/1).

M.P. 126–127 °C (Hexane/EtOAc).

^{1}H NMR (500 MHz, $CDCl_3$, 298 K): δ 8.60 (s, 1H, H-12), 7.79 (dd, *J* = 8.5, 2.3 Hz, 1H, H-14), 7.70 (d, *J* = 7.8 Hz, 2H, H-17), 7.48 (d, *J* = 8.5 Hz, 1H, H-15), 7.40–7.34 (m, 2H, H-18), 7.29–7.21 (m, 2H, H-7 and H-19), 7.17–7.12 (m, 1H, H-6), 6.91–6.85 (m, 1H, H-5), 5.95 (d, *J* = 7.6 Hz, 1H, H-4), 4.68 (dd, *J* = 8.7, 5.2 Hz, 1H, H-2), 4.41 (s, 1H, OH), 3.10–3.00 (m, 1H, H-9), 2.85–2.76 (m, 1H, H-9), 2.21–2.05 (m, 2H, H-10) ppm.

^{13}C NMR (125 MHz, $CDCl_3$, 298 K): δ 162.7 (C-11), 148.6 (C-12), 147.1 (C-8), 145.5 (C-16), 141.6 (C-3), 139.5 (C-14), 128.4 (C-18), 127.3 (C-6 or C-19), 127.1 (C-6 or C-19), 126.2 (C-17), 125.7 (C-5), 125.0 (C-4), 124.9 (C-7), 122.9 (C-15), 119.2 (C-13), 80.8 (C-1), 53.8 (C-2), 31.8 (C-9), 28.4 (C-10) ppm.

HRMS (ESI) m/z: $[M+H]^+$ calcd for $C_{21}H_{19}{}^{79}BrNO^+$ 380.0645, found 380.0646.

IR (thin film): ṽ 3392 (s), 2950 (s), 2918 (s), 1573 (s), 1445 (s), 1357 (s), 1158 (s), 1097 (s) cm^{-1}.

Specific rotation: $[\alpha]_D^{30}$ = +94.0 (*c* 1, CH_2Cl_2).

The **enantiomeric ratio** of **3lp** was determined by SFC analysis (CHIRALPACK IE (25 cm), column temperature 25 °C, solvent CO_2/MeOH = 90/10, flow rate = 2.0 mL/min): t_R = 12.0 min (minor), t_R = 13.0 min (major).

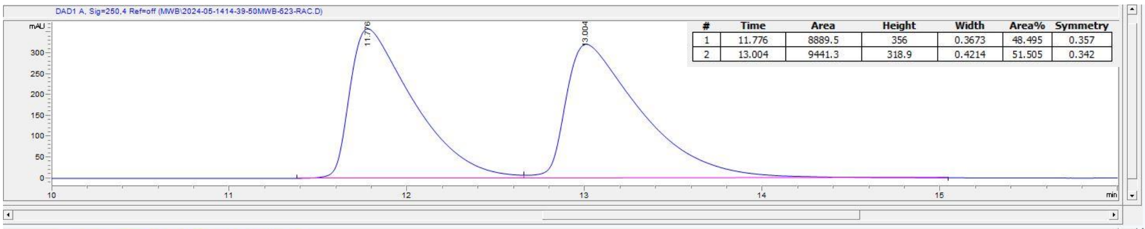

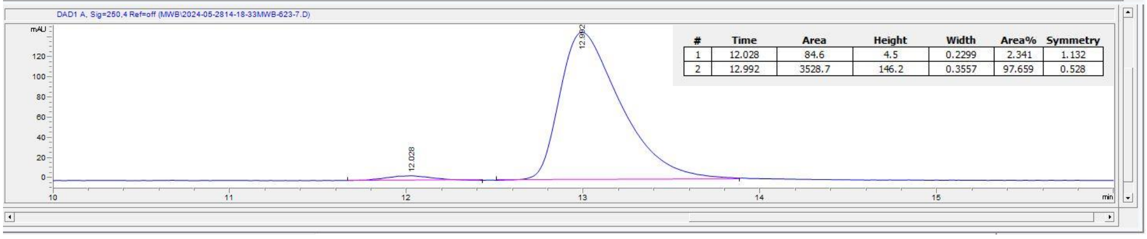

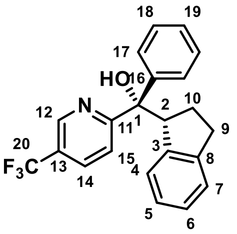

$C_{22}H_{18}F_3NO$
M = 369.39

(*S*)-((*R*)-2,3-Dihydro-1*H*-inden-1-yl)(phenyl)(5-(trifluoromethyl)pyridin-2-yl)methanol (**3mp**, **GP 6**) was prepared from phenyl(5-(trifluoromethyl)pyridin-2-yl)methanol (**1m**, 25.3 mg, 0.10 mmol, 100 mol%) and indene (**2p**, 29.3 µL, 29.0 mg, 0.25 mmol, 250 mol%), using [Ir(cod)$_2$]BARF (12.7 mg, 10 µmol, 10 mol%), and **L10** ((*R*,*R*)-QuinoxP*, 3.34 mg, 10 µmol, 10 mol%) in H_2O (0.2 mL) at 110 °C for 3 d. Purification by flash column chromatography on silica gel (hexane/ethyl acetate from 100/1 to 19/1) afforded **3mp** (28.0 mg, 76% yield, 93% e.e., >20:1 d.r., >20:1 r.r.) as a colorless solid. Diastereomer and regiomer ratios (>20:1 d.r., >20:1 r.r.) were determined from the ^{1}H NMR spectrum of crude material.

R*f* = 0.5 (Hexane/EtOAc = 19/1).

M.**P**. 108–109 °C (Hexane/EtOAc).

^{1}H NMR (500 MHz, $CDCl_3$, 298 K): δ 8.81 (s, 1H, H-12), 7.91 (dd, *J* = 8.4, 2.3 Hz, 1H, H-14), 7.75–7.69 (m, 3H, H-15 and H-17), 7.39–7.34 (m, 2H, H-18), 7.27 (d, *J* = 7.7 Hz, 1H, H-19), 7.23 (d, *J* = 7.5 Hz, 1H, H-7), 7.16–7.11 (m, 1H, H-6), 6.87–6.82 (m, 1H, H-5), 5.85 (d, *J* = 7.6 Hz, 1H, H-4), 4.76 (dd, *J* = 8.7, 5.2 Hz, 1H, H-2), 4.33 (s, 1H, OH), 3.09–3.01 (m, 1H, H-9), 2.84–2.75 (m, 1H, H-9), 2.21–2.13 (m, 1H, H-10), 2.13–2.05 (m, 1H, H-10) ppm.

^{13}C NMR (125 MHz, $CDCl_3$, 298 K): δ 168.1 (C-11), 147.1 (C-8), 145.1 (C-16), 144.8 ($J_{C–F}$ = 12.5 Hz, C-12), 141.4 (C-3), 134.1 ($J_{C–F}$ = 3.3 Hz, C-14), 128.5 (C-18), 127.4 (C-6 or C-19), 127.3 (C-6 or C-19), 126.3 (C-17), 125.7 (C-5), 125.2 ($J_{C–F}$ = 32.9 Hz, C-13), 125.1 (C-4),

124.8 (C-7), 123.7 ($J_{C\text{–}F}$ = 270.8 Hz, C-20), 121.3 (C-15), 81.2 (C-1), 53.9 (C-2), 31.8 (C-9), 28.4 (C-10) ppm.

^{19}F NMR (471 MHz, $CDCl_3$, 298 K): δ –62.1 ppm.

HRMS (ESI) m/z: $[M+H]^+$ calcd for $C_{22}H_{19}F_3NO^+$ 370.1413, found 370.1410.

IR (thin film): ṽ 3382 (s), 2955 (s), 2922 (s), 1607 (s), 1446 (s), 1334 (s), 1113 (s), 1014 (s) cm^{-1}.

Specific rotation: $[\alpha]_D^{30}$ = +51.1 (*c* 1, CH_2Cl_2).

The **enantiomeric ratio** of **3mp** was determined by SFC analysis (CHIRALPACK ID (25 cm), column temperature 25 °C, solvent CO_2/MeOH = 90/10, flow rate = 2.0 mL/min): t_R = 3.8 min (major), t_R = 4.5 min (minor).

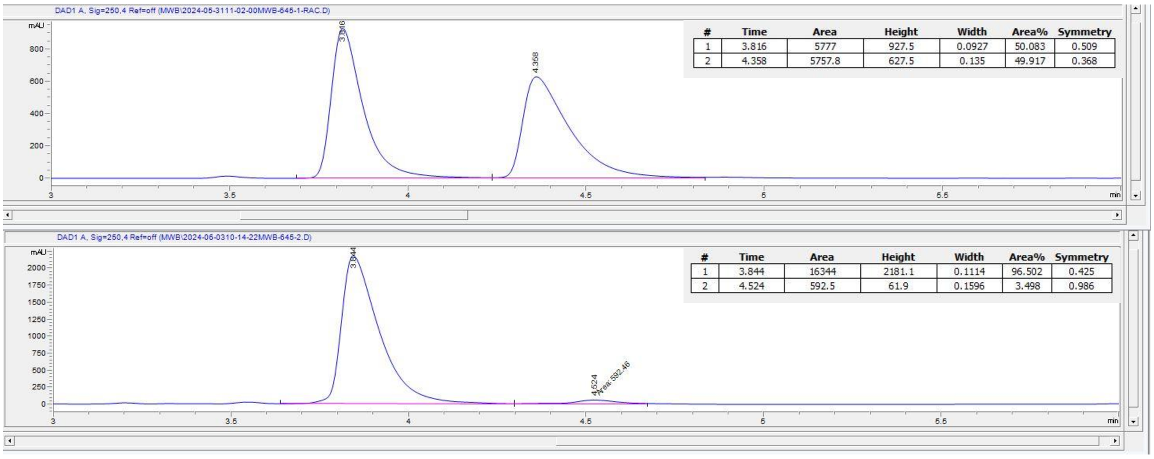

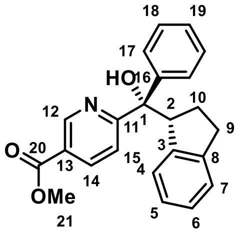

$C_{23}H_{21}NO_3$
M = 359.43

Methyl 6-((*S*)-((*R*)-2,3-dihydro-1*H*-inden-1-yl)(hydroxy)(phenyl)methyl)nicotinate (**3np**, **GP 6**) was prepared from methyl 6-(hydroxy(phenyl)methyl)nicotinate (**1n**, 24.3 mg, 0.10 mmol, 100 mol%) and indene (**2p**, 29.3 µL, 29.0 mg, 0.25 mmol, 250 mol%), using [Ir(cod)$_2$]BARF (12.7 mg, 10 µmol, 10 mol%), and **L10** ((*R*,*R*)-QuinoxP*, 3.34 mg, 10 µmol, 10 mol%) in H_2O (0.2 mL) at 110 °C for 3 d. Purification by flash column chromatography on silica gel (hexane/ethyl acetate from 100/1 to 19/1) afforded **3np** (24.9 mg, 69% yield, 94% e.e.,

>20:1 d.r., >20:1 r.r.) as a colorless solid. Diastereomer and regiomer ratios (>20:1 d.r., >20:1 r.r.) were determined from the ^{1}H NMR spectrum of crude material.

R$_f$ = 0.5 (Hexane/EtOAc = 9/1).

M.P. 124–125 °C (Hexane/EtOAc).

^{1}H NMR (500 MHz, $CDCl_3$, 298 K): δ 9.13 (d, *J* = 2.2 Hz, 1H, H-12), 8.28 (dd, *J* = 8.3, 2.2 Hz, 1H, H-14), 7.72 (d, *J* = 7.0 Hz, 2H, H-17), 7.67 (d, *J* = 8.4 Hz, 1H, H-15), 7.39–7.34 (m, 2H, H-18), 7.28–7.23 (m, 1H, H-19), 7.22 (d, *J* = 7.5 Hz, 1H, H-7), 7.14–7.10 (m, 1H, H-6), 6.84–6.79 (m, 1H, H-5), 5.83 (d, *J* = 7.6 Hz, 1H, H-4), 4.74 (s, 1H, OH), 4.70 (dd, *J* = 8.7, 5.0 Hz, 1H, H-2), 3.96 (s, 3H, H-21), 3.11–3.03 (m, 1H, H-9), 2.84–2.76 (m, 1H, H-9), 2.23–2.09 (m, 2H, H-10) ppm.

^{13}C NMR (125 MHz, $CDCl_3$, 298 K): δ 168.1 (C-11), 165.7 (C-20), 148.9 (C-12), 147.0 (C-8), 145.4 (C-16), 141.6 (C-3), 138.0 (C-14), 128.5 (C-18), 127.3 (C-6 or C-19), 127.2 (C-6 or C-19), 126.4 (C-17), 125.6 (C-5), 124.9 (C-4), 124.9 (C-7), 124.6 (C-13), 121.2 (C-15), 81.2 (C-1), 53.9 (C-2), 52.6 (C-21), 31.9 (C-9), 28.5 (C-10) ppm.

HRMS (ESI) m/z: $[M+Na]^+$ calcd for $C_{23}H_{22}NO_3^+$ 360.1594, found 360.1594.

IR (thin film): ṽ 3380 (br), 2956 (s), 2935 (s), 1725 (s), 1595 (s), 1435 (s), 1274 (s), 1116 (s) cm^{-1}.

Specific rotation: $[\alpha]_D^{28}$ = +120.4 (*c* 1, CH_2Cl_2).

The **enantiomeric ratio** of **3np** was determined by SFC analysis (CHIRALPACK AD (25 cm), column temperature 25 °C, solvent CO_2/MeOH = 80/20, flow rate = 2.0 mL/min): t_R = 9.9 min (minor), t_R = 11.7 min (major).

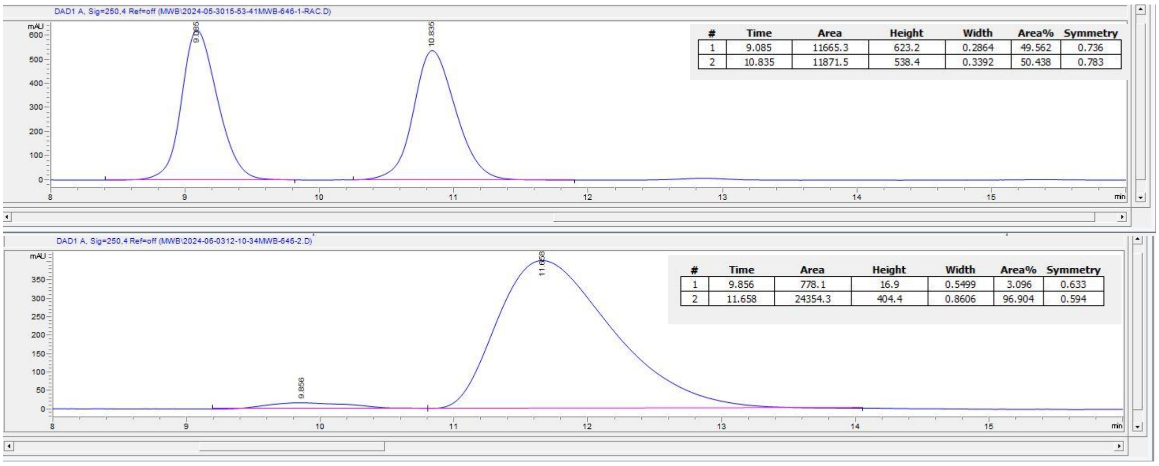

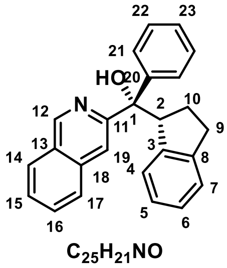

(*S*)-((*R*)-2,3-Dihydro-1*H*-inden-1-yl)(isoquinolin-3-yl)(phenyl)methanol (**3op**, **GP 6**) was prepared from isoquinolin-3-yl(phenyl)methanol (**1o**, 23.5 mg, 0.10 mmol, 100 mol%) and indene (**2p**, 29.3 µL, 29.0 mg, 0.25 mmol, 250 mol%), using [Ir(cod)$_2$]BARF (12.7 mg, 10 µmol, 10 mol%), and **L10** ((*R*,*R*)-QuinoxP*, 3.34 mg, 10 µmol, 10 mol%) in H_2O (0.2 mL) at 110 °C for 3 d. Purification by flash column chromatography on silica gel (hexane/ethyl acetate from 100/1 to 19/1) afforded **3op** (29.6 mg, 84% yield, 99% e.e., >20:1 d.r., >20:1 r.r.) as a colorless solid. Diastereomer and regiomer ratios (>20:1 d.r., >20:1 r.r.) were determined from the ^{1}H NMR spectrum of crude material.

R*f* = 0.5 (Hexane/EtOAc = 9/1).

M.P. 182–183 °C (Hexane/EtOAc).

^{1}H NMR (500 MHz, $CDCl_3$, 298 K): δ 9.22 (s, 1H, H-12), 7.99 (d, *J* = 8.3 Hz, 1H, H-14), 7.95 (s, 1H, H-19), 7.83–7.78 (m, 3H, H-17 and H-21), 7.71–7.66 (m, 1H, H-16), 7.62–7.57 (m, 1H, H-15), 7.38–7.34 (m, 2H, H-22), 7.25–7.21 (m, 2H, H-7 and H-23), 7.12–7.07 (m, 1H, H-6), 6.76–6.71 (m, 1H, H-5), 5.91 (d, *J* = 7.6 Hz, 1H, H-4), 4.86 (t, *J* = 7.1 Hz, 1H, H-2), 4.67 (s, 1H, OH), 3.09–3.01 (m, 1H, H-9), 2.86–2.78 (m, 1H, H-9), 2.20–2.14 (m, 2H, H-10) ppm.

^{13}C NMR (125 MHz, $CDCl_3$, 298 K): δ 157.7 (C-11), 150.7 (C-12), 147.0 (C-8), 146.7 (C-20), 142.2 (C-3), 136.6 (C-18), 130.7 (C-16), 128.3 (C-22), 127.7 (C-13 or C-14), 127.7 (C-13 or C-14), 127.3 (Ar), 127.2 (Ar), 127.0 (Ar), 126.7 (Ar), 126.3 (C-21), 125.5 (C-5), 125.3 (C-4), 124.8 (C-7), 117.4 (C-19), 80.7 (C-1), 54.2 (C-2), 31.9 (C-9), 28.6 (C-10) ppm.

HRMS (ESI) m/z: $[M+H]^+$ calcd for $C_{25}H_{22}NO^+$ 352.1696, found 352.1692.

IR (thin film): ṽ 3320 (s), 2912 (s), 2840 (s), 1627 (s), 1446 (s), 1379 (s), 1191 (s), 1068 (s) cm^{-1}.

Specific rotation: $[\alpha]_D^{28}$ = +86.5 (*c* 1, CH_2Cl_2).

The **enantiomeric ratio** of **3op** was determined by SFC analysis (CHIRALPACK AD (25 cm), column temperature 25 °C, solvent CO_2/MeOH = 70/30, flow rate = 2.0 mL/min): t_R = 8.3 min (minor), t_R = 11.7 min (major).

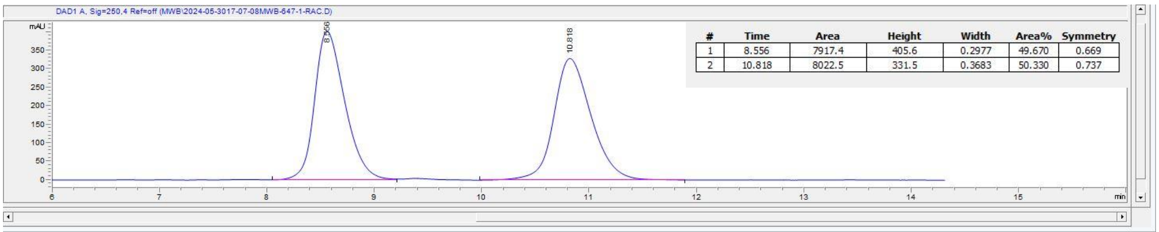

#	Time	Area	Height	Width	Area%	Symmetry
1	8.556	7917.4	405.6	0.2977	49.670	0.669
2	10.818	8022.5	331.5	0.3683	50.330	0.737

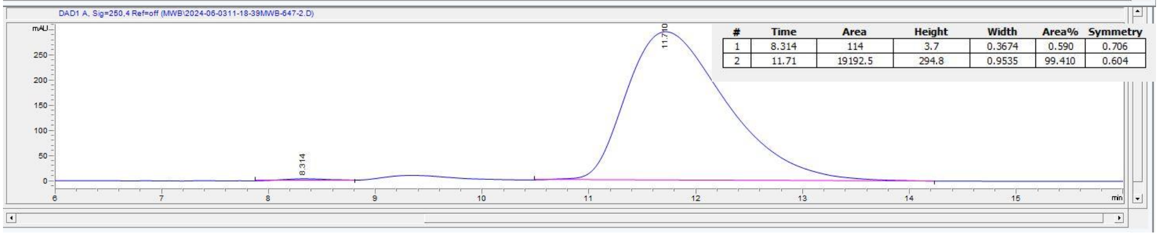

#	Time	Area	Height	Width	Area%	Symmetry
1	8.314	114	3.7	0.3674	0.590	0.706
2	11.71	19192.5	294.8	0.9535	99.410	0.604

4 Gram Scale Reaction

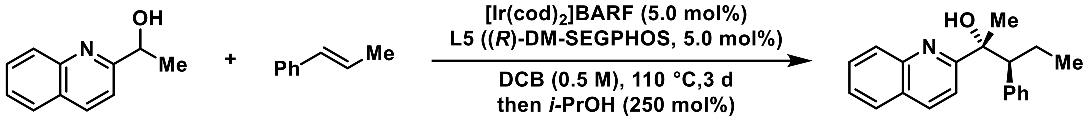

1a (1.04 g, 6.0 mmol) + 2a (250 mol%)

3aa: 73% & 14% Yield
>99% & >99% e.e., 5:1 d.r.
>20:1 r.r.

Scheme S12. Gram-scale reaction for **3aa**.

A gram-scale reaction was conducted according to **GP 6**, using 1-(quinolin-2-yl)ethan-1-ol (**1a**, 1.04 g, 6.00 mmol, 100 mol%), (*E*)-1-propenylbenzene (**2a**, 1.95 mL, 1.77 g, 15.0 mmol, 250 mol%), [Ir(cod)$_2$]BARF (382 mg, 0.30 mmol, 5.0 mol%) and **L5** ((*R*)-DM-SEGPHOS, 217 mg, 0.30 mmol, 5.0 mol%) in DCB (12 mL) at 110 °C for 3 d. Then, *i*-PrOH (1.15 mL, 901 mg, 15 mmol, 250 mol%) was added, and the reaction mixture was stirred at 110 °C for 2 d. Purification by flash column chromatography on silica gel (hexane/ethyl acetate from 100/1 to 19/1) afforded **3aa** (1.27 g, 73% yield, >99% e.e., >20:1 d.r., >20:1 r.r.) as a colorless solid and **3aa'** (248 mg, 14% yield, >99% e.e., >20:1 d.r., >20:1 r.r.) as a colorless oil. Diastereomer and regiomer ratios (5:1 d.r., >20:1 r.r.) were determined from the ^{1}H NMR spectrum of crude material.

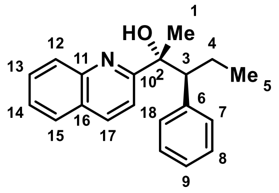

$C_{20}H_{21}NO$
M = 291.39

(2*R*,3*S*)-3-Phenyl-2-(quinolin-2-yl)pentan-2-ol (3aa)

R*f* = 0.4 (Hexane/EtOAc = 15/1).

M.P. 151–152 °C (Hexane/EtOAc).

^{1}**H NMR** (500 MHz, $CDCl_3$, 298 K): δ 8.18 (d, *J* = 8.6 Hz, 1H, H-17), 8.13 (d, *J* = 8.5 Hz, 1H, H-12), 7.86 (d, *J* = 8.1 Hz, 1H, H-15), 7.78–7.74 (m, 1H, H-13), 7.60–7.55 (m, 1H, H-14), 7.46–7.40 (m, 3H, H-7 and H-18), 7.36–7.31 (m, 2H, H-8), 7.29–7.24 (m, 1H, H-9), 6.08 (s, 1H, OH), 2.85 (dd, *J* = 11.7, 3.1 Hz, 1H, H-3), 1.87–1.75 (m, 1H, H-4), 1.34 (s, 3H, H-1), 1.22–1.13 (m, 1H, H-4), 0.54 (t, *J* = 7.4 Hz, 3H, H-5) ppm.

^{13}**C NMR** (125 MHz, $CDCl_3$, 298 K): δ 165.2 (C-10), 145.7 (C-11), 141.4 (C-6), 137.4 (C-17), 130.2 (C-7), 130.0 (C-13), 129.0 (C-12), 128.1 (C-8), 127.6 (C-15), 127.2 (C-16), 126.6 (C-14), 126.6 (C-9), 117.6 (C-18), 76.3 (C-2), 58.7 (C-3), 28.0 (C-1), 23.1 (C-4), 12.6 (C-5) ppm.

The **enantiomeric ratio** of **3aa** was determined by SFC analysis (CHIRALPACK OD-H (25 cm), column temperature 25 °C, solvent CO_2/MeOH = 90/10, flow rate = 2.0 mL/min): t_R = 5.6 min (minor), t_R = 6.4 min (major).

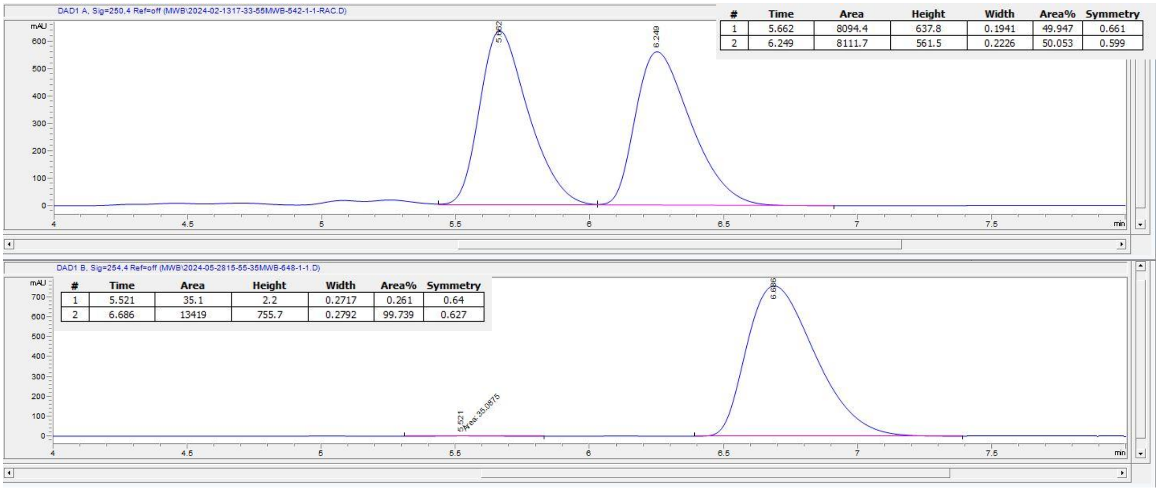

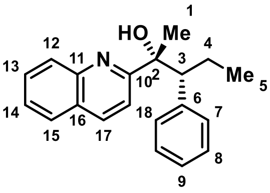

$C_{20}H_{21}NO$
M = 291.39

(2*R*,3*R*)-3-Phenyl-2-(quinolin-2-yl)pentan-2-ol (3aa')

$\mathbf{R_f}$ = 0.4 (Hexane/EtOAc = 10/1).

M.P. 70–71 °C (Hexane/EtOAc).

^{1}H NMR (500 MHz, $CDCl_3$, 298 K): δ 8.04 (d, *J* = 8.6 Hz, 1H, H-17), 7.90 (d, *J* = 8.5 Hz, 1H, H-12), 7.74 (d, *J* = 8.1 Hz, 1H, H-15), 7.65–7.61 (m, 1H, H-13), 7.49–7.45 (m, 1H, H-14), 7.27 (d, *J* = 8.6 Hz, 1H, H-18), 7.05–7.00 (m, 4H, H-7 and H-8), 7.00–6.95 (m, 1H, H-9), 5.92 (s, 1H, OH), 2.95 (dd, *J* = 11.8, 3.3 Hz, 1H, H-3), 2.24–2.15 (m, 1H, H-4), 1.86–1.76 (m, 1H, H-4), 1.67 (s, 3H, H-1), 0.72 (t, *J* = 7.4 Hz, 3H, H-5) ppm.

^{13}C NMR (125 MHz, $CDCl_3$, 298 K): δ 164.1 (C-10), 145.6 (C-11), 140.7 (C-6), 136.4 (C-17), 129.7 (C-7), 129.6 (C-13), 128.9 (C-12), 127.5 (C-8), 127.4 (C-15), 126.9 (C-16), 126.3 (C-14), 126.2 (C-9), 118.6 (C-18), 76.2 (C-2), 58.5 (C-3), 27.5 (C-1), 22.2 (C-4), 12.8 (C-5) ppm.

Specific rotation: $[\alpha]_D^{30}$ = −28.7 (*c* 0.5, CH_2Cl_2).

The **enantiomeric ratio** of **3aa'** was determined by SFC analysis (CHIRALPACK SC (25 cm), column temperature 25 °C, solvent CO_2/MeOH = 90/10, flow rate = 2.0 mL/min): t_R = 4.3 min (minor), t_R = 4.8 min (major).

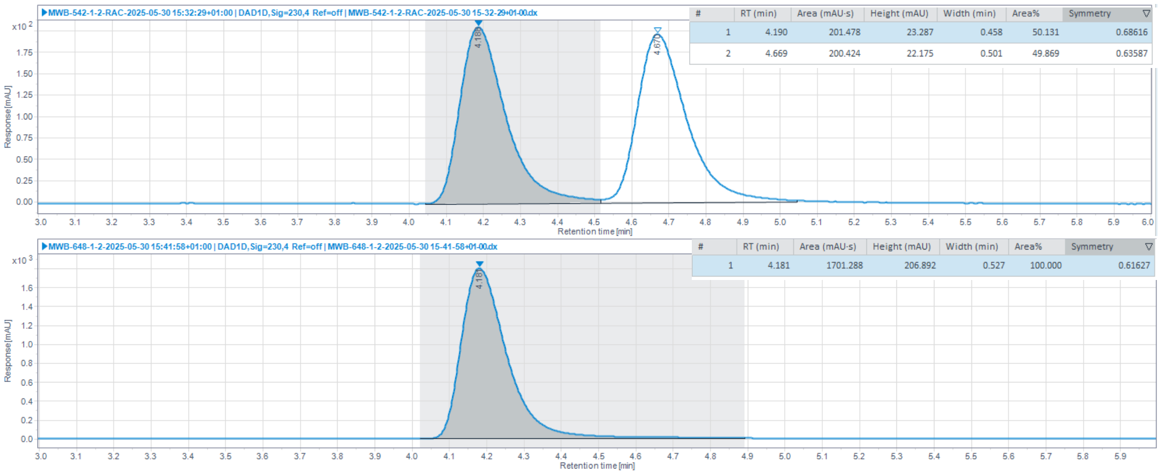

Crystal **3aa'** was obtained through recrystallization in the solution of DCM and methanol at room temperature. The absolute configuration of **3aa'** was confirmed unambiguously by **X-ray diffraction analysis**, and other compounds were assigned by analogy. CCDC 2480109 contains the supplementary crystallographic data for this compound.

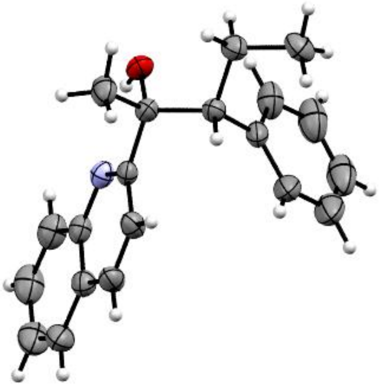

Figure S4. Molecular structure of **3aa'**. Thermal ellipsoids represent 50% probability level.

5 Derivatizations

5.1 Stereoselective Quinoline Reduction

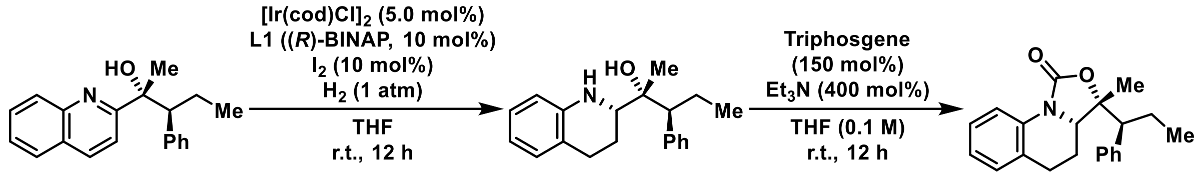

Scheme S13. Stereoselective quinoline reduction.

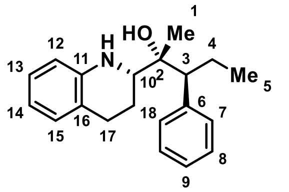

$C_{20}H_{25}NO$
M = 295.43

(2*R*,3*S*)-3-Phenyl-2-((*S*)-1,2,3,4-tetrahydroquinolin-2-yl)pentan-2-ol (**4a**) was prepared according to a reported procedure.[10a] A Schlenk tube was charged with (2*R*,3*S*)-3-Phenyl-2-(quinolin-2-yl)pentan-2-ol (**3aa**, >99% ee, >20:1 d.r., 29.1 mg, 0.10 mmol, 100 mol%), $[Ir(cod)Cl]_2$ (3.36 mg, 5.0 µmol, 5.0 mol%), **L1** ((*R*)-BINAP, 6.23 mg, 10 µmol, 10 mol%) and I_2 (2.54 mg, 10 µmol, 10 mol%). The Schlenk tube was evacuated and backfilled with H_2 for three times. Then THF (1 mL, 0.1 M) was added. The reaction mixture was stirred at room temperature for 12 h. Purification by flash column chromatography on silica gel (hexane/ethyl acetate from 100/1 to 19/1) afforded **4a** (27.1 mg, 92% yield, 99% e.e., >20:1 d.r.) as a colorless oil. Diastereomer ratios (>20:1 d.r.) were determined from the ^{1}H NMR spectrum of crude material.

Racemic **3-phenyl-2-(1,2,3,4-tetrahydroquinolin-2-yl)pentan-2-ol** was prepared from *rac*-3aa according to the procedure above, using (±)-BINAP as the ligand.

R*f* = 0.3 (Hexane/EtOAc = 9/1).

^{1}H NMR (500 MHz, $CDCl_3$, 298 K): δ 7.35–7.30 (m, 2H, H-8), 7.29–7.24 (m, 3H, H-7 and H-9), 6.99–6.93 (m, 2H, H-13 and H-15), 6.66–6.60 (m, 1H, H-14), 6.45 (d, *J* = 7.9 Hz, 1H, H-12), 3.72 (s, 1H, NH), 3.25 (dd, *J* = 10.5, 2.7 Hz, 1H, H-10), 2.85–2.69 (m, 3H, H-3, and H-17), 2.23–2.11 (m, 2H, H-4, and H-18), 1.87–1.72 (m, 3H, H-4, H-18, and OH), 1.18 (s, 3H, H-1), 0.71 (t, *J* = 7.3 Hz, 3H, H-5) ppm.

^{13}C NMR (125 MHz, $CDCl_3$, 298 K): δ 144.9 (C-11), 141.1 (C-6), 129.7 (C-7), 129.2 (C-15), 128.5 (C-8), 126.9 (C-9 or C-13), 126.8 (C-9 or C-13), 122.3 (C-16), 117.7 (C-14), 115.1 (C-12), 75.7 (C-2), 58.8 (C-10), 54.8 (C-3), 27.1 (C-17), 22.8 (C-18), 22.1 (C-4), 22.0 (C-1), 12.8 (C-5) ppm.

HRMS (ESI) m/z: $[M–H]^+$ calcd for $C_{20}H_{24}NO^+$ 296.2009, found 296.2011.

IR (thin film): ṽ 3407 (br), 2963 (s), 2832 (s), 1605 (s), 1481 (s), 1309 (s), 1106 (s) cm^{-1}.

Specific rotation: $[\alpha]_D^{29}$ = –20.7 (*c* 1, CH_2Cl_2).

The **enantiomeric ratio** of **4a** was determined by SFC analysis (CHIRALPACK IE (25 cm), column temperature 25 °C, solvent CO_2/*i*PrOH = 80/20, flow rate = 2.0 mL/min): t_R = 4.7 min (minor), t_R = 5.0 min (major).

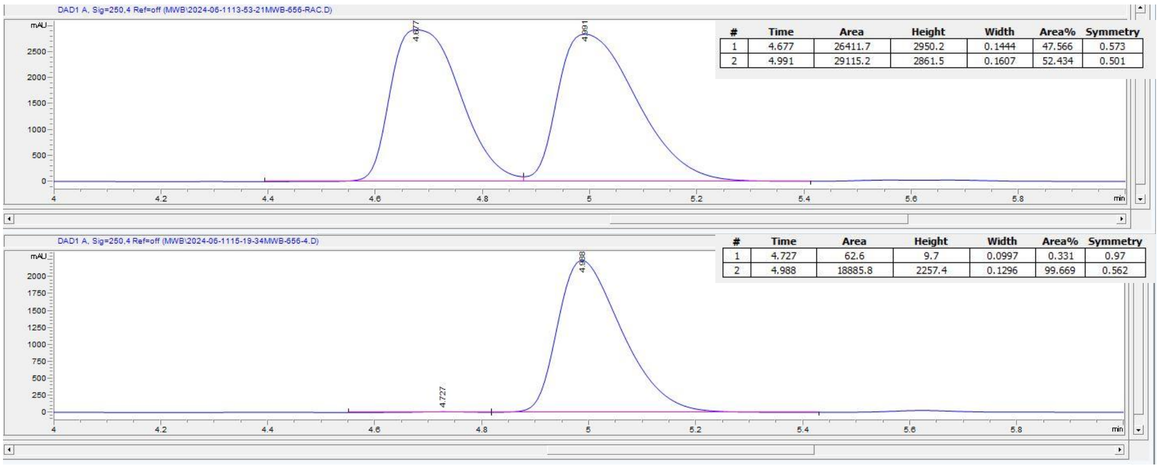

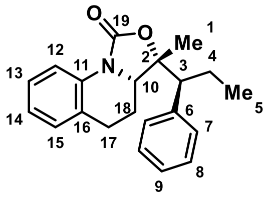

$C_{21}H_{23}NO_2$
M = 321.42

(3*R*,3a*S*)-3-Methyl-3-((*S*)-1-phenylpropyl)-3,3a,4,5-tetrahydro-1*H*-oxazolo[3,4-*a*]quinolin-1-one (**8a**) was prepared according to a reported procedure.[10b] A Schlenk tube was charged with (2*R*,3*S*)-3-phenyl-2-((*S*)-1,2,3,4-tetrahydroquinolin-2-yl)pentan-2-ol (**4a**, 29.5 mg, 0.10 mmol, 100 mol%) and triphosgene (44.5 mg, 0.15 mmol, 150 mol%). The Schlenk tube was evacuated and backfilled with N_2 for three times. Then THF (1 mL, 0.1 M) and Et_3N (55.7 µL, 40.4 mg, 0.40 mmol, 400 mol%) were added. The reaction mixture was stirred at room temperature for 12 h. The reaction was quenched by water (10 mL) and extracted by ethyl acetate (3 × 10 mL). The combined organic phase was dried by anhydrous $MgSO_4$, filtered

and concentrated under reduced pressure. Purification by flash column chromatography on silica gel (hexane/ethyl acetate from 100/1 to 9/1) afforded **8a** (30.5 mg, 95% yield, 99% e.e., >20:1 d.r.) as a colorless oil. Diastereomer ratios (>20:1 d.r.) were determined from the ^{1}H NMR spectrum of crude material.

R$_f$ = 0.3 (Hexane/EtOAc = 9/1).

^{1}H NMR (500 MHz, $CDCl_3$, 298 K): δ 8.48 (d, J = 8.2 Hz, 1H, H-12), 7.35–7.30 (m, 4H, H-7 and H-8), 7.30–7.22 (m, 2H, H-9 and H-13), 7.16 (d, J = 7.5 Hz, 1H, H-15), 7.04–6.99 (m, 1H, H-14), 3.99 (dd, J = 12.5, 3.0 Hz, 1H, H-10), 3.03–2.90 (m, 2H, H-17), 2.56 (dd, J = 11.4, 2.5 Hz, 1H, H-3), 2.17–2.11 (m, 1H, H-18), 2.08–1.99 (m, 1H, H-18), 1.99–1.90 (m, 1H, H-4), 1.88–1.77 (m, 1H, H-4), 1.24 (s, 3H, H-1), 0.64 (t, J = 7.3 Hz, 3H, H-5) ppm.

^{13}C NMR (125 MHz, $CDCl_3$, 298 K): δ 154.3 (C-19), 140.8 (C-6), 135.3 (C-11), 129.6 (C-7), 129.2 (C-15), 128.5 (C-8), 127.6 (C-13), 127.1 (C-9), 124.2 (C-16), 123.0 (C-14), 117.5 (C-12), 83.6 (C-2), 66.0 (C-10), 54.2 (C-3), 27.9 (C-17), 26.8 (C-1), 23.8 (C-4), 21.2 (C-18), 12.1 (C-5) ppm.

HRMS (ESI) m/z: $[M+H]^+$ calcd for $C_{21}H_{24}NO_2^+$ 322.1802, found 322.1801.

IR (thin film): ṽ 2967 (s), 2936 (s), 1737 (s), 1603 (m), 1492 (s), 1369 (s), 1224 (s), 1007 (s), 753 (s) cm^{-1}.

Specific rotation: $[\alpha]_D^{29}$ = +132.8 (c 1, CH_2Cl_2).

The **enantiomeric ratio** of **8a** was determined by SFC analysis (CHIRALPACK IE (25 cm), column temperature 25 °C, solvent CO_2/iPrOH = 80/20, flow rate = 2.0 mL/min): t_R = 8.7 min (major), t_R = 9.7 min (minor).

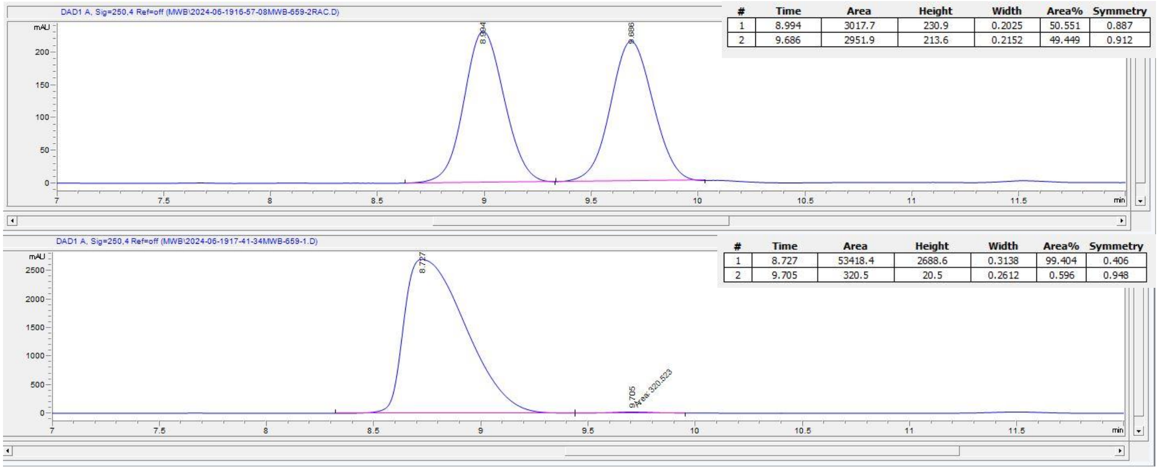

#	Time	Area	Height	Width	Area%	Symmetry
1	8.994	3017.7	230.9	0.2025	50.551	0.887
2	9.686	2951.9	213.6	0.2152	49.449	0.912

#	Time	Area	Height	Width	Area%	Symmetry
1	8.727	53418.4	2688.6	0.3138	99.404	0.406
2	9.705	320.5	20.5	0.2612	0.596	0.948

H,H-NOESY Spectrum **of 8a**

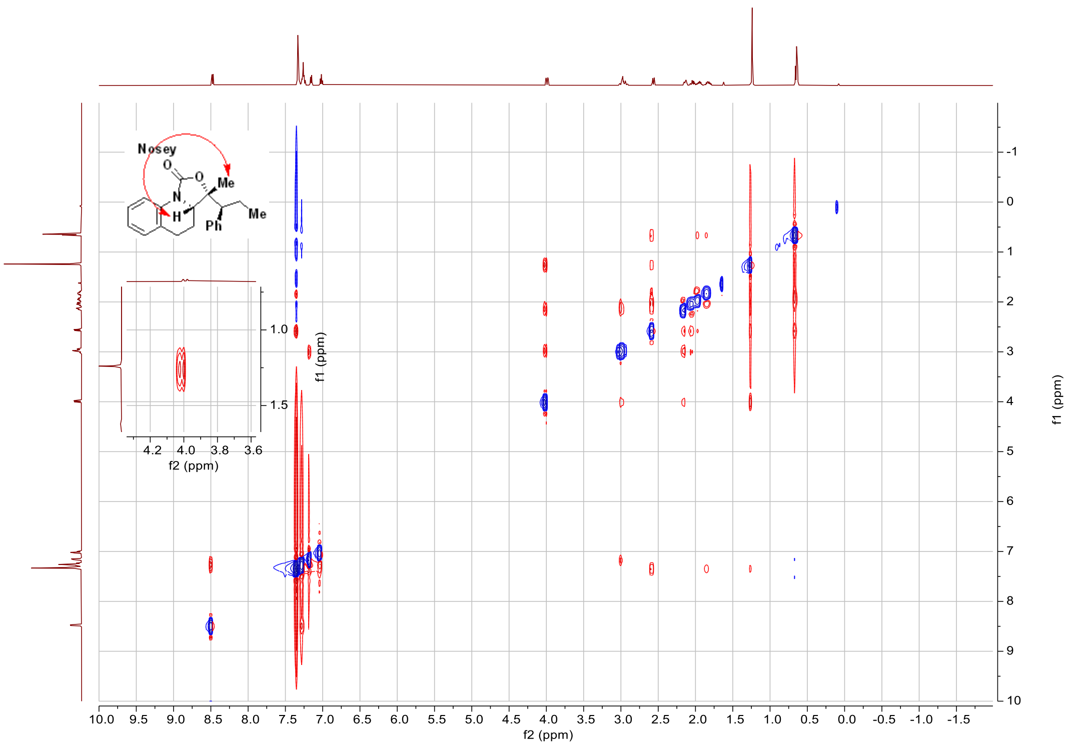

From the H,H-NOESY spectrum above, the correlation between H-10 and H-1 (3.99 ppm and 1.24 ppm) confirms the relative stereochemistry.

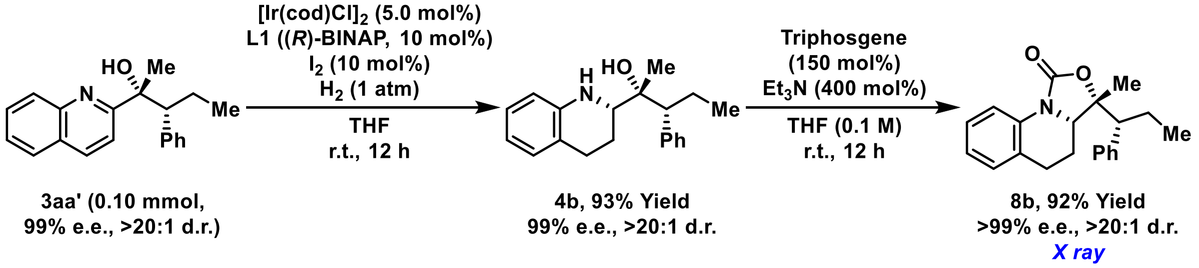

Scheme S14. Stereoselective quinoline reduction.

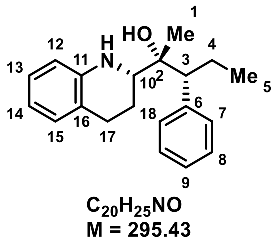

(2*R*,3*R*)-3-Phenyl-2-((*S*)-1,2,3,4-tetrahydroquinolin-2-yl)pentan-2-ol (**4b**) was prepared according to a reported procedure.[12a] A Schlenk tube was charged with (2*R*,3*R*)-3-phenyl-2-(quinolin-2-yl)pentan-2-ol (**3aa'**, >99% ee, >20:1 d.r., 29.1 mg, 0.10 mmol, 100 mol%), $[Ir(cod)Cl]_2$ (3.36 mg, 5.0 µmol, 5.0 mol%), **L1** ((*R*)-BINAP, 6.23 mg, 10 µmol, 10 mol%) and I_2 (2.54 mg, 10 µmol, 10 mol%). The Schlenk tube was evacuated and backfilled with H_2 for three times. Then THF (1 mL, 0.1 M) was added. The reaction mixture was stirred at room

temperature for 12 h. Purification by flash column chromatography on silica gel (hexane/ethyl acetate from 100/1 to 19/1) afforded **4b** (27.4 mg, 93% yield, 99% e.e., >20:1 d.r.) as a colorless oil. Diastereomer ratios (>20:1 d.r.) were determined from the ^{1}H NMR spectrum of crude material.

Racemic **3-phenyl-2-(1,2,3,4-tetrahydroquinolin-2-yl)pentan-2-ol** was prepared from *rac*-3aa' according to the procedure above, using (±)-BINAP as the ligand.

R*f* = 0.3 (Hexane/EtOAc = 9/1).

^{1}H NMR (500 MHz, $CDCl_3$, 298 K): δ 7.36–7.29 (m, 4H, H-7 and H-8), 7.28–7.23 (m, 1H, H-9), 7.00–6.96 (m, 1H, H-13), 6.95 (d, *J* = 7.6 Hz, 1H, H-15), 6.65–6.61 (m, 1H, H-14), 6.53 (d, *J* = 8.1 Hz, 1H, H-12), 3.98 (s, 1H, NH), 3.17 (dd, *J* = 10.9, 2.9 Hz, 1H, H-10), 2.85 (dd, *J* = 11.6, 3.3 Hz, 1H, H-3), 2.79–2.72 (m, 2H, H-17), 2.10–2.04 (m, 1H, H-18), 1.90–1.74 (m, 3H, H-4, and H-18), 1.65 (s, 1H, OH), 1.30 (s, 3H, H-1), 0.74 (t, *J* = 7.3 Hz, 3H, H-5) ppm.

^{13}C NMR (125 MHz, $CDCl_3$, 298 K): δ 145.0 (C-11), 140.4 (C-6), 130.0 (C-7), 129.1 (C-15), 128.5 (C-8), 127.0 (C-9 or C-13), 126.9 (C-9 or C-13), 122.2 (C-16), 117.6 (C-14), 115.2 (C-12), 75.4 (C-2), 58.8 (C-10), 53.3 (C-3), 27.2 (C-17), 22.9 (C-4), 22.7 (C-18), 21.4 (C-1), 12.9 (C-5) ppm.

HRMS (ESI) m/z: $[M–H]^+$ calcd for $C_{20}H_{24}NO^+$ 296.2009, found 296.2010.

IR (thin film): ṽ 3406 (br), 2963 (s), 2832 (s), 1605 (s), 1479 (s), 1310 (s), 1111 (s), 702 (s) cm^{-1}.

Specific rotation: $[\alpha]_D^{29}$ = +90.8 (*c* 1, CH_2Cl_2).

The **enantiomeric ratio** of **4b** was determined by SFC analysis (CHIRALPACK IE (25 cm), column temperature 25 °C, solvent CO_2/*i*-PrOH = 80/20, flow rate = 2.0 mL/min): t_R = 5.6 min (major), t_R = 6.5 min (minor).

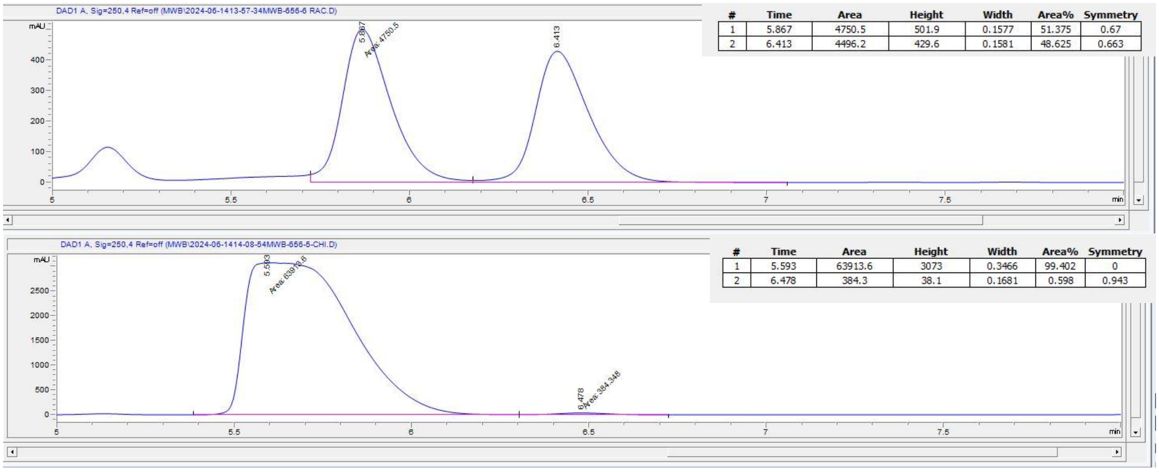

#	Time	Area	Height	Width	Area%	Symmetry
1	5.867	4750.5	501.9	0.1577	51.375	0.67
2	6.413	4496.2	429.6	0.1581	48.625	0.663

#	Time	Area	Height	Width	Area%	Symmetry
1	5.593	63913.6	3073	0.3466	99.402	0
2	6.478	384.3	38.1	0.1681	0.598	0.943

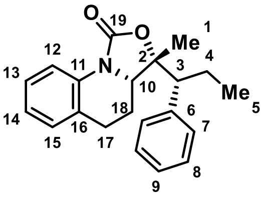

$C_{21}H_{23}NO_2$
M = 321.42

(3*R*,3a*S*)-3-Methyl-3-((*R*)-1-phenylpropyl)-3,3a,4,5-tetrahydro-1*H*-oxazolo[3,4-*a*]quinolin-1-one (**8b**) was prepared according to a reported procedure.[12b] A Schlenk tube was charged with (2*R*,3*R*)-3-phenyl-2-((*S*)-1,2,3,4-tetrahydroquinolin-2-yl)pentan-2-ol (**4b**, 29.5 mg, 0.10 mmol, 100 mol%) and triphosgene (44.5 mg, 0.15 mmol, 150 mol%). The Schlenk tube was evacuated and backfilled with N_2 for three times. Then THF (1 mL, 0.1 M) and Et_3N (55.7 µL, 40.4 mg, 0.40 mmol, 400 mol%) were added. The reaction mixture was stirred at room temperature for 12 h. The reaction was quenched by water (10 mL) and extracted by ethyl acetate (3 × 10 mL). The combined organic phase was dried by anhydrous $MgSO_4$, filtered and concentrated under reduced pressure. Purification by flash column chromatography on silica gel (hexane/ethyl acetate from 100/1 to 9/1) afforded **8b** (29.5 mg, 92% yield, >99% e.e., >20:1 d.r.) as a colorless solid. Diastereomer ratios (>20:1 d.r.) were determined from the ^1H NMR spectrum of crude material.

R_f = 0.3 (Hexane/EtOAc = 9/1).

M.P. 100–101 °C (Hexane/EtOAc).

^{1}H NMR (500 MHz, $CDCl_3$, 298 K): δ 7.90 (d, *J* = 8.2 Hz, 1H, H-12), 7.35–7.30 (m, 2H, H-8), 7.29–7.25 (m, 3H, H-7 and H-9), 7.25–7.19 (m, 1H, H-13), 7.13 (d, *J* = 7.5 Hz, 1H, H-15), 7.07–7.02 (m, 1H, H-14), 3.79 (dd, *J* = 12.2, 2.2 Hz, 1H, H-10), 3.04–2.89 (m, 3H, H-3 and H-17), 2.20–2.15 (m, 1H, H-18), 2.01–1.87 (m, 2H, H-4 and H-18), 1.68–1.59 (m, 1H, H-4), 1.51 (s, 3H, H-1), 0.82 (t, *J* = 7.3 Hz, 3H, H-5) ppm.

^{13}C NMR (125 MHz, $CDCl_3$, 298 K): δ 154.6 (C-19), 138.0 (C-6), 135.8 (C-11), 130.1 (C-7), 128.9 (C-15), 128.3 (C-8), 127.2 (C-9 or C-13), 127.1 (C-9 or C-13), 125.5 (C-16), 123.9 (C-14), 120.8 (C-12), 83.0 (C-2), 65.0 (C-10), 51.0 (C-3), 27.3 (C-17), 23.5 (C-4), 22.7 (C-18), 21.7 (C-1), 12.3 (C-5) ppm.

HRMS (ESI) m/z: $[M+H]^+$ calcd for $C_{21}H_{24}NO_2^+$ 322.1802, found 322.1803.

IR (thin film): ṽ 2973 (s), 2875 (s), 1745 (s), 1493 (s), 1369 (s), 1221 (s), 1091 (s), 762 (s) cm^{-1}.

Specific rotation: $[\alpha]_D^{22}$ = +89.5 (c 1, CH_2Cl_2).

The **enantiomeric ratio** of **8b** was determined by SFC analysis (CHIRALPACK IE (25 cm), column temperature 25 °C, solvent CO_2/MeOH = 80/20, flow rate = 2.0 mL/min): t_R = 11.4 min (minor), t_R = 11.9 min (major).

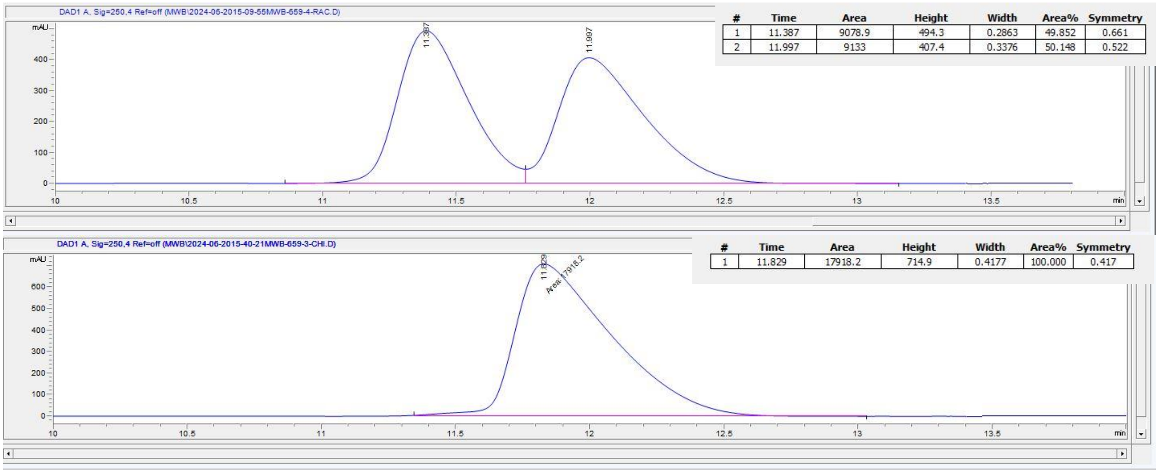

H,H-NOESY Spectrum **of 8b**

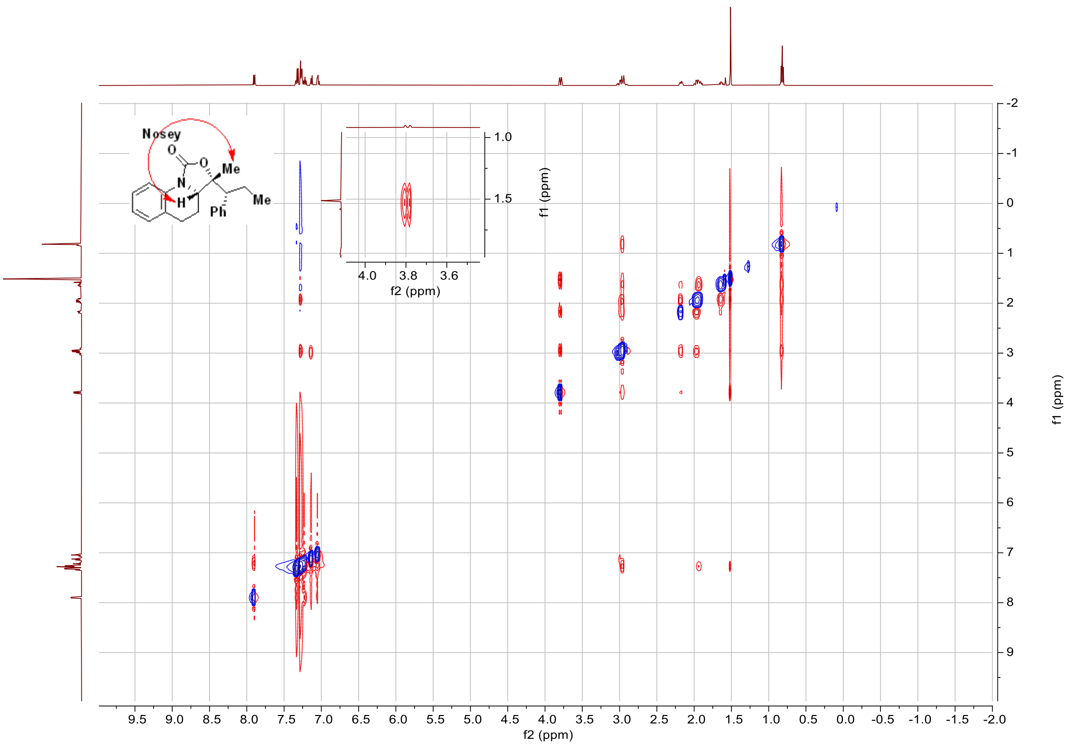

From the H,H-NOESY spectrum above, the correlation between H-10 and H-1 (3.79 ppm and 1.51 ppm) confirms the relative stereochemistry.

Crystal **8b** was obtained through recrystallization in the solution of DCM and methanol at room temperature. The absolute configuration of **8b** was confirmed unambiguously by **X-ray diffraction analysis**. CCDC 2480113 contains the supplementary crystallographic data for this compound.

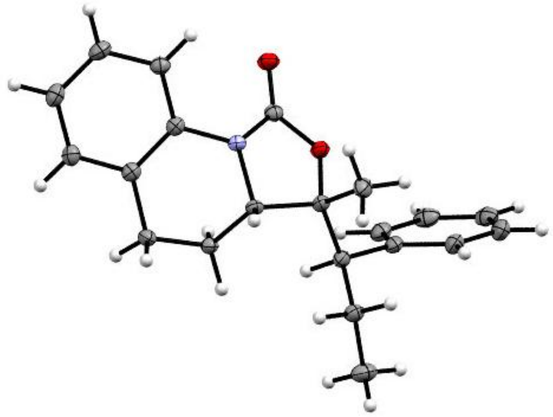

Figure S5. Molecular structure of **8b**. Thermal ellipsoids represent 50% probability level.

5.2 Intramolecular Cylization of 3ac

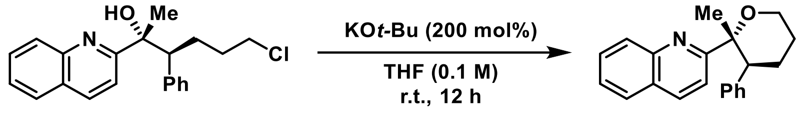

3ac
(0.10 mmol, 98% ee; >20:1 d.r.)

5, 91% Yield, 99% e.e., >20:1 d.r.

Scheme S15. Intramolecular Cylization of **3ac**.

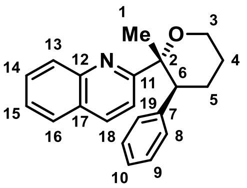

$C_{21}H_{21}NO$
M = 303.41

2-((2*R*,3*S*)-2-Methyl-3-phenyltetrahydro-2H-pyran-2-yl)quinoline (**5**) was prepared according to a reported procedure.[11] A flame-dried Schlenk tube was charged with (2*R*,3*S*)-6-chloro-3-phenyl-2-(quinolin-2-yl)hexan-2-ol (**3ac**, 98% ee, >20:1 d.r., 34.0 mg, 0.10 mmol, 100 mol%, 98% *ee*, > 20:1 *d.r.*) and KO*t*Bu (22.4 mg, 0.2 mmol, 200 mol%). The Schlenk tube was evacuated and backfilled with N_2 for three times. Then THF (1 mL, 0.1 M) was added. After stirring at room temperature for 12 h, the solvent was removed under reduced pressure. Purification by flash column chromatography on silica gel (hexane/ethyl acetate from 100/1 to 19/1) afforded **5** (27.7 mg, 91% yield, 99% ee, >20:1 d.r.) as a colorless oil. Diastereomer ratios (>20:1 d.r.) were determined from the ^{1}H NMR spectrum of crude material.

Racemic **2-(2-methyl-3-phenyltetrahydro-2H-pyran-2-yl)quinoline** was prepared according to the procedure above, using *racemic* 6-chloro-3-phenyl-2-(quinolin-2-yl)hexan-2-ol ((*rac*)-**3ac**, 34.0 mg, 0.10 mmol, 100 mol%) as the starting material.

R_f = 0.5 (Hexane/EtOAc = 9/1).

^{1}H NMR (500 MHz, $CDCl_3$, 298 K): δ 8.19 (d, *J* = 8.6 Hz, 1H, H-18), 8.04 (d, *J* = 8.4 Hz, 1H, H-13), 7.84 (d, *J* = 8.6 Hz, 1H, H-19), 7.82 (d, *J* = 8.4 Hz, 1H, H-16), 7.71–7.66 (m, 1H, H-14), 7.55–7.51 (m, 1H, H-15), 7.38 (d, *J* = 6.9 Hz, 2H, H-8), 7.27–7.18 (m, 3H, H-9 and H-10), 4.09–4.01 (m, 2H, H-3 and H-6), 3.80–3.73 (m, 1H, H-3), 2.09–1.99 (m, 2H, H-4 and H-5), 1.96–1.89 (m, 1H, H-5), 1.47 (s, 3H, H-1), 1.51–1.43 (m, 1H, H-4) ppm.

^{13}C NMR (125 MHz, $CDCl_3$, 298 K): δ 165.1 (C-11), 147.3 (C-12), 143.2 (C-7), 136.4 (C-18), 130.0 (C-8), 129.8 (C-13), 129.1 (C-14), 127.9 (C-9), 127.4 (C-16), 127.1 (C-17), 126.3 (C-15), 126.1 (C-10), 119.2 (C-19), 80.9 (C-2), 63.4 (C-3), 46.0 (C-6), 26.7 (C-5), 24.6 (C-1), 22.4 (C-4) ppm.

HRMS (ESI) m/z: $[M+H]^+$ calcd for $C_{21}H_{22}NO^+$ 304.1696, found 304.1697.

IR (thin film): ṽ 2930 (s), 2860 (s), 1600 (s), 1499 (s), 1369 (s), 1112 (s), 1072 (s) cm^{-1}.

Specific rotation: $[\alpha]_D^{29}$ = –143.4 (*c* 1, CH_2Cl_2).

The **enantiomeric ratio** of **5** was determined by SFC analysis (CHIRALPACK OD-H (25 cm), column temperature 25 °C, solvent CO_2/MeOH = 90/10, flow rate = 2.0 mL/min): t_R = 8.1 min (minor), t_R = 10.6 min (major).

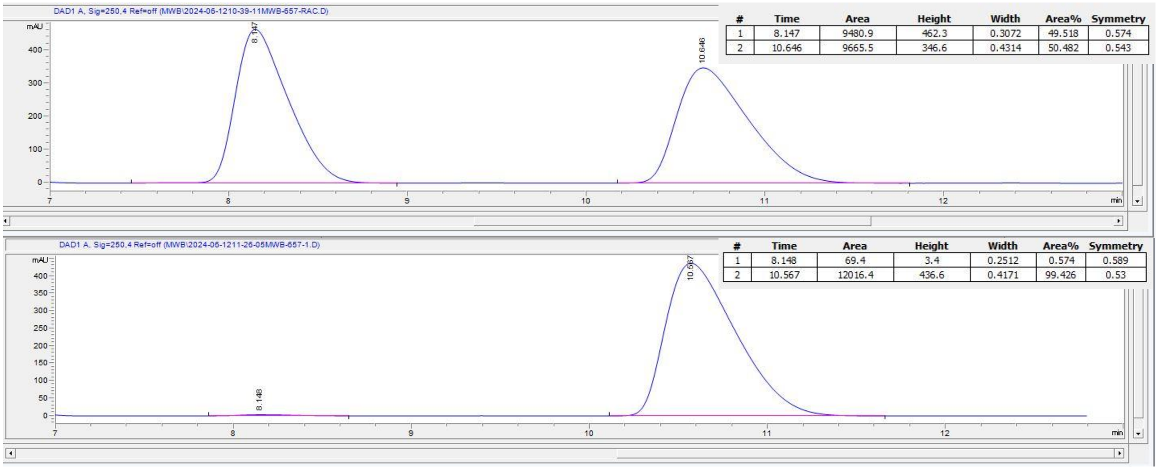

#	Time	Area	Height	Width	Area%	Symmetry
1	8.147	9480.9	462.3	0.3072	49.518	0.574
2	10.646	9665.5	346.6	0.4314	50.482	0.543

#	Time	Area	Height	Width	Area%	Symmetry
1	8.148	69.4	3.4	0.2512	0.574	0.589
2	10.567	12016.4	436.6	0.4171	99.426	0.53

6 Mechanistic Studies

6.1 Control Experiments

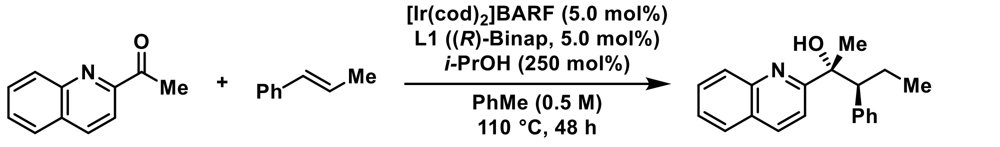

1a-H$_2$ (0.10 mmol) 2a (250 mol%) 3aa, 53% Yield, 98% e.e., 4:1 d.r., > 20:1 r.r.

Scheme S16. Ketones as starting materials in the transformation.

(2*R*,3*S*)-3-Phenyl-2-(quinolin-2-yl)pentan-2-ol (**3aa**, **GP6**) was prepared from 1-(quinolin-2-yl)ethan-1-one (**1a-H$_2$**,17.1 mg, 0.10 mmol, 100 mol%) and (*E*)-1-propenylbenzene (**2a**, 32.4 µL, 29.5 mg, 0.25 mmol, 250 mol%), using [Ir(cod)$_2$]BARF (6.36 mg, 5.0 µmol, 5.0 mol%), **L1** ((*R*)-BINAP, 3.11 mg, 5.0 µmol, 5.0 mol%) and *i*-PrOH (19.1 µL, 15.0 mg, 0.25 mmol, 250 mol%) in toluene (0.2 mL) at 110 °C for 48 h. Purification by flash column chromatography on silica gel (hexane/ethyl acetate from 100/1 to 19/1) afforded **3aa** (15.3 mg, 53% yield, 98% ee, >20:1 d.r., >20:1 r.r.) as a colorless solid. Diastereomer and regiomer ratios (4:1 d.r., >20:1 r.r.) were determined from the ^{1}H NMR spectrumof crude material.

R*f* = 0.4 (Hexane/EtOAc = 9/1).

M.P. 151–152 °C (Hexane/EtOAc).

^{1}H NMR (500 MHz, $CDCl_3$, 298 K): δ 8.18 (d, *J* = 8.6 Hz, 1H, H-17), 8.13 (d, *J* = 8.5 Hz, 1H, H-12), 7.86 (d, *J* = 8.1 Hz, 1H, H-15), 7.78–7.74 (m, 1H, H-13), 7.60–7.55 (m, 1H, H-14), 7.46–7.40 (m, 3H, H-7 and H-18), 7.36–7.31 (m, 2H, H-8), 7.29–7.24 (m, 1H, H-9), 6.08 (s, 1H, OH), 2.85 (dd, *J* = 11.7, 3.1 Hz, 1H, H-3), 1.87–1.75 (m, 1H, H-4), 1.34 (s, 3H, H-1), 1.22–1.13 (m, 1H, H-4), 0.54 (t, *J* = 7.4 Hz, 3H, H-5) ppm.

^{13}C NMR (125 MHz, $CDCl_3$, 298 K): δ 165.2 (C-10), 145.7 (C-11), 141.4 (C-6), 137.4 (C-17), 130.2 (C-7), 130.0 (C-13), 129.0 (C-12), 128.1 (C-8), 127.6 (C-15), 127.2 (C-16), 126.6 (C-14), 126.6 (C-9), 117.6 (C-18), 76.3 (C-2), 58.7 (C-3), 28.0 (C-1), 23.1 (C-4), 12.6 (C-5) ppm.

The **enantiomeric ratio** of **3aa** was determined by SFC analysis (CHIRALPACK OD-H (25 cm), column temperature 25 °C, solvent CO_2/MeOH = 90/10, flow rate = 2.0 mL/min): t_R = 5.6 min (minor), t_R = 6.4 min (major).

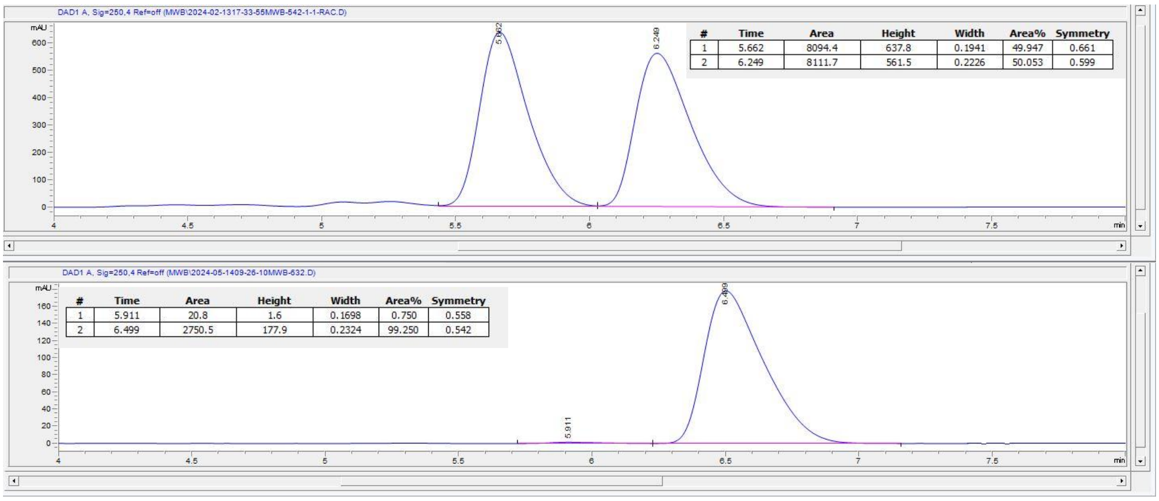

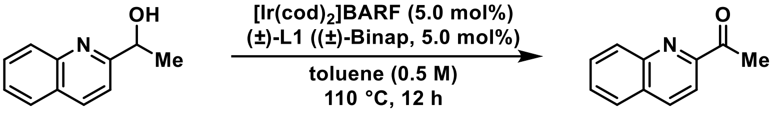

1a (0.10 mmol)
65% recovered

1a-H_2, 33% Yield

Scheme S17. Trandformation of alcohols to ketones under Ir catalysis.

GP 6: A flame-dried Schlenk tube was charged with 1-(quinolin-2-yl)ethan-1-ol (**1a**, 17.3 mg, 0.10 mmol, 100 mol%), [Ir(cod)$_2$]BARF (6.36 mg, 5.0 μmol, 5.0 mol%) and (±)-**L1** ((±)-BINAP, 3.11 mg, 5.0 μmol, 5.0 mol%). The Schlenk tube was evacuated and backfilled with N_2 for three times. Then toluene (0.2 mL, 0.5 M) was added. The tube was sealed and heated at 110 °C in a heating plate for 12 h. After cooling to room temperature, the solvent was removed under reduced pressure. Purification by flash column chromatography on silica gel (hexane/ethyl acetate from 100/1 to 19/1) afforded **1a-H_2** (5.68 mg, 33% yield) as a colorless solid.

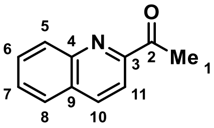

$C_{11}H_9NO$
M = 171.20

1-(Quinolin-2-yl)ethan-1-one (**1a-H_2**)

R_f = 0.45 (Hexane/ethyl acetate = 15/1).

^{1}H NMR (500 MHz, $CDCl_3$, 298 K): δ 8.24 (d, J = 8.5 Hz, 1H, H-10), 8.19 (d, J = 8.5 Hz, 1H, H-5), 8.11 (d, J = 8.5 Hz, 1H, H-11), 7.85 (d, J = 8.2 Hz, 1H, H-8), 7.80–7.75 (m, 1H, H-6), 7.66–7.61 (m, 1H, H-7), 2.86 (s, 3H, H-1) ppm.

^{13}C NMR (125 MHz, $CDCl_3$, 298 K): δ 200.8 (C-2), 153.3 (C-3), 147.3 (C-4), 137.0 (C-10), 130.7 (C-5), 130.1 (C-6), 129.7 (C-9), 128.7 (C-7), 127.8 (C-8), 118.1 (C-11), 25.7 (C-1) ppm.

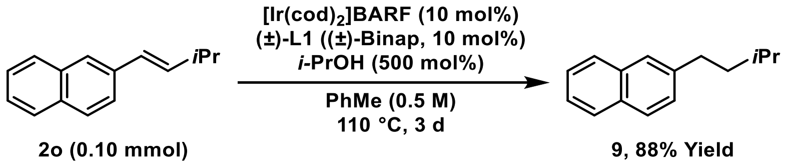

Scheme S18. Reduction of alkenes to alkanes under Ir catalysis.

GP 6: A flame-dried Schlenk tube was charged with (*E*)-2-(3-methylbut-1-en-1-yl)naphthalene (**2o**, 19.6 mg, 0.10 mmol, 100 mol%), [Ir(cod)$_2$]BARF (12.7 mg, 10 μmol, 10 mol%) and (±)-**L1** ((±)-BINAP, 6.23 mg, 10 μmol, 10 mol%). The Schlenk tube was evacuated and backfilled with N_2 for three times. Then toluene (0.2 mL, 0.5 M) was added, followed by the addition of *i*-PrOH (38.3 μL, 30.0 mg, 0.50 mmol, 500 mol%). The tube was sealed and heated at 110 °C for 3 d. After cooling to room temperature, the solvent was removed under reduced pressure. Purification by flash column chromatography on silica gel (hexane) afforded **9** (17.4 mg, 88% yield) as a colorless oil.

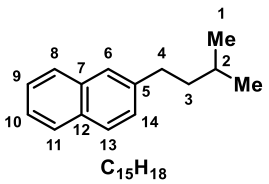

$C_{15}H_{18}$
M = 198.31

2-*iso*-Pentylnaphthalene (**9**)

R*f* = 0.6 (Hexane).

^{1}H NMR (500 MHz, $CDCl_3$, 298 K): δ 7.84–7.77 (m, 3H, ArH and H-13), 7.64 (s, 1H, H-6), 7.49–7.45 (m, 1H, ArH), 7.45–7.41 (m, 1H, ArH), 7.38–7.34 (m, 1H, H-14), 2.80 (t, *J* = 7.8 Hz, 2H, H-4), 1.71–1.59 (m, 3H, H-2 and H-3), 1.00 (d, *J* = 6.2 Hz, 6H, H-1) ppm.

^{13}C NMR (125 MHz, $CDCl_3$, 298 K): δ 140.8 (C-5), 133.8 (C-7), 132.0 (C-12), 127.9 (Ar), 127.7 (Ar), 127.6 (Ar), 127.5 (Ar), 126.3 (Ar), 125.9 (Ar), 125.1 (Ar), 40.9 (C-3), 34.1 (C-4), 27.9 (C-2), 22.7 (C-1) ppm.

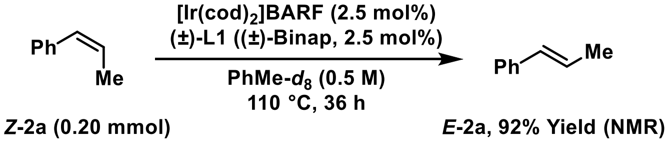

Scheme S19. *Z* to *E* isomerization under Ir catalysis.

GP 6: A flame-dried Schlenk tube was charged [Ir(cod)$_2$]BARF (6.36 mg, 5.0 μmol, 2.5 mol%) and (±)-**L1** ((±)-BINAP, 3.11 mg, 5.0 μmol, 2.5 mol%). The Schlenk tube was evacuated and backfilled with N_2 for three times. Then toluene (0.2 mL, 0.5 M) and with (*Z*)-prop-1-en-1-ylbenzene (**Z-2a**, 25.9 μL, 23.6 mg, 0.2 mmol, 100 mol%) was added. The tube was sealed

and heated at 110 °C for 36 h. The reaction mixture was then analyzed by ^{1}H NMR directly, showing 92% yield of **2a** was formed using 1,3,5-trimethoxybenzene as the internal standard.

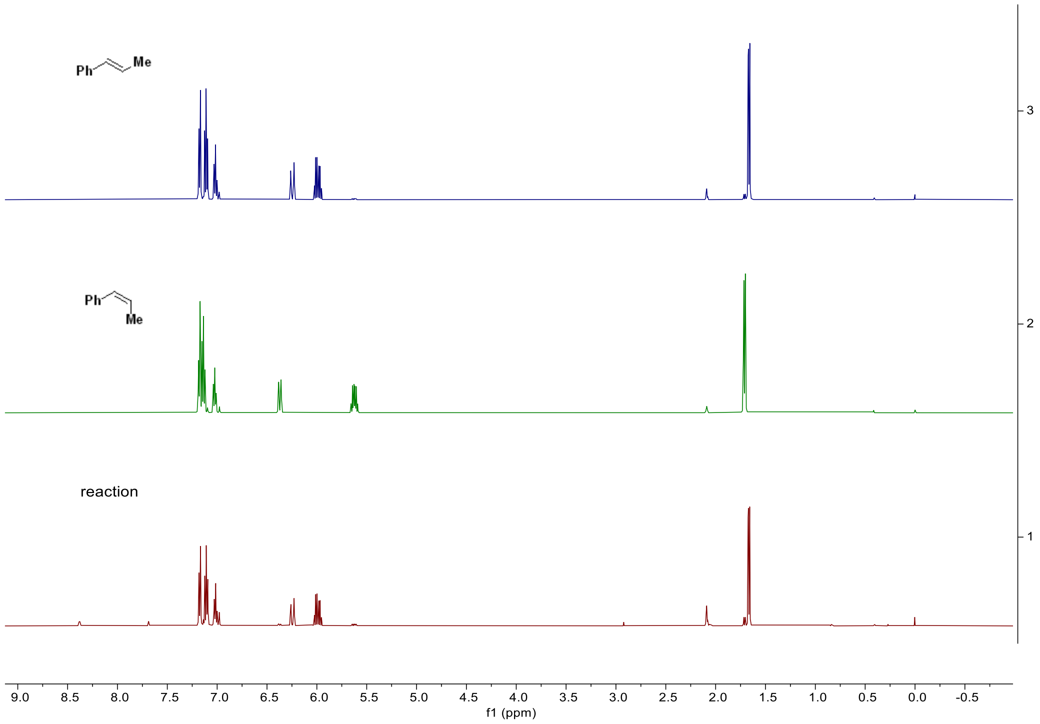

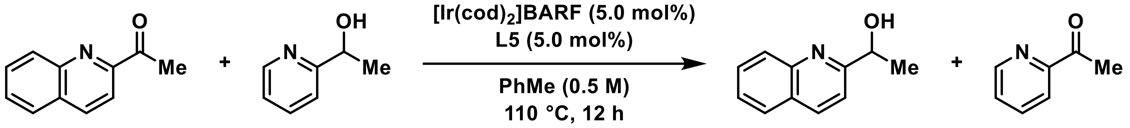

**1a–H$_2$**	**1b**	**1a**	**1b–H$_2$**
**(0.1 mmol)**	**(0.1 mmol)**	50% Yield	50% Yield
50% left	40% left		

Scheme S20. A cross-over reaction between alcohol and ketone substrates shows that alcohol dehydrogenation is fast and reversible.

GP 6: A flame-dried Schlenk tube was charged [Ir(cod)$_2$]BARF (6.36 mg, 5.0 μmol, 2.5 mol%), **L5** ((*R*)-DM-SEGPHOS, 3.61 mg, 5.0 μmol, 2.5 mol%), 1-(quinolin-2-yl)ethan-1-one (1a-H2,17.1 mg, 0.10 mmol, 100 mol%) and 1-(pyridin-2-yl)ethan-1-ol (**1b**, 12.3 mg, 0.10 mmol, 100 mol%). The Schlenk tube was evacuated and backfilled with N_2 for three times. Then toluene (0.2 mL, 0.5 M) was added. The tube was sealed and heated at 110 °C for 36 h. The reaction mixture was then analyzed by ^{1}H NMR directly, giving the results in **Scheme S20**, as analyzed using 1,3,5-trimethoxybenzene as an internal standard.

6.2 Deuterium Exchange Experiments

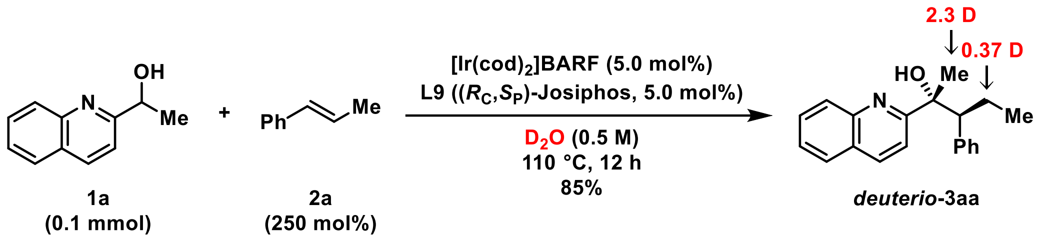

Scheme S21. Deuterium exchange experiment with **1a** and **2a**.

GP6: A flame-dried Schlenk tube was charged with 1-(quinolin-2-yl)ethan-1-ol (**1a**, 17.3 mg, 0.10 mmol, 100 mol%), [Ir(cod)$_2$]BARF (6.36 mg, 5.0 µmol, 5.0 mol%) and **L9** ((*R*$_C$,*S*$_P$)-Josiphos, 3.20 mg, 5.0 µmol, 5.0 mol%). The Schlenk tube was evacuated and backfilled with N_2 for three times. Then D_2O (0.2 mL, 0.5 M) was added, followed by the addition of (*E*)-1-propenylbenzene (**2a**, 32.4 µL, 29.5 mg, 0.25 mmol, 250 mol%). The tube was sealed and heated at 110 °C for 12 h. After cooling to room temperature, the solvent was removed under reduced pressure. Purification by flash column chromatography on silica gel (hexane/ethyl acetate from 100/1 to 19/1) afforded ***deuterio*-3aa** (24.6 mg, 85% yield) as a colorless solid. The obtained products were analyzed by ^{1}H NMR and ^{2}H NMR spectroscopy using $CDCl_3$ as solvent.

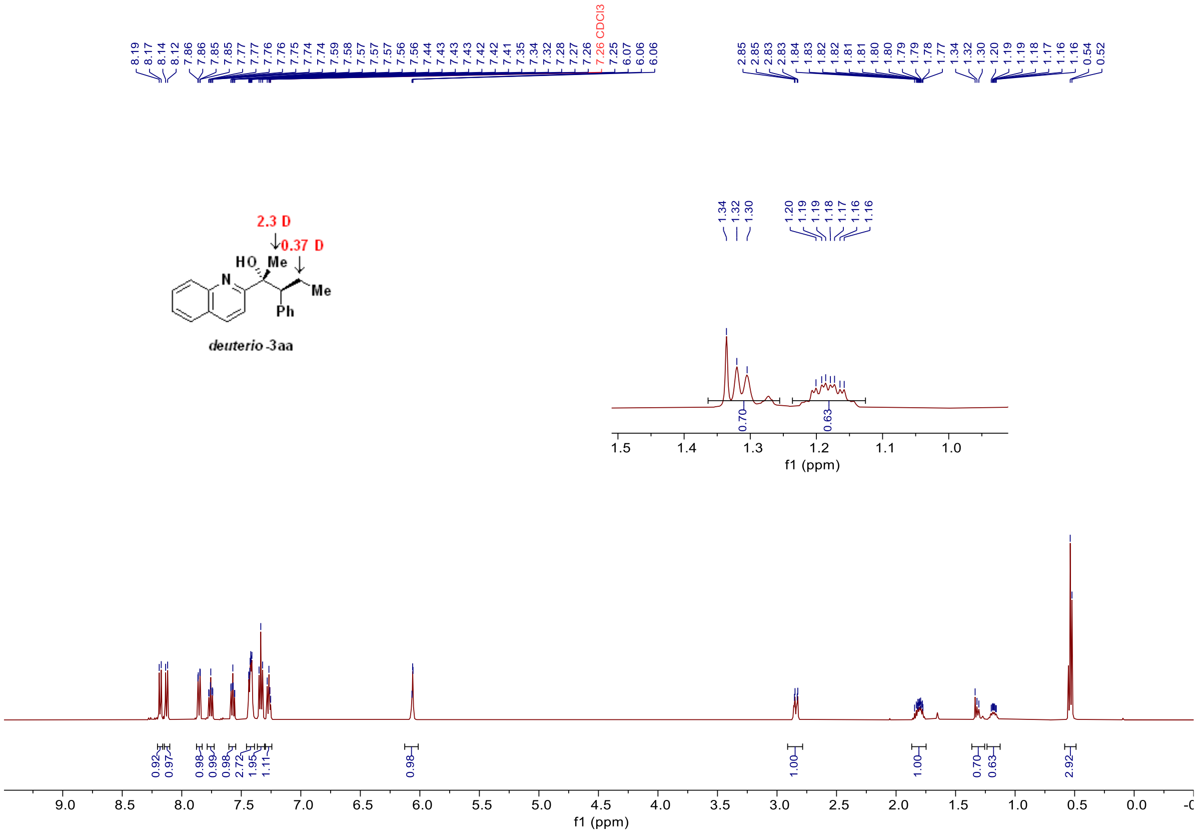

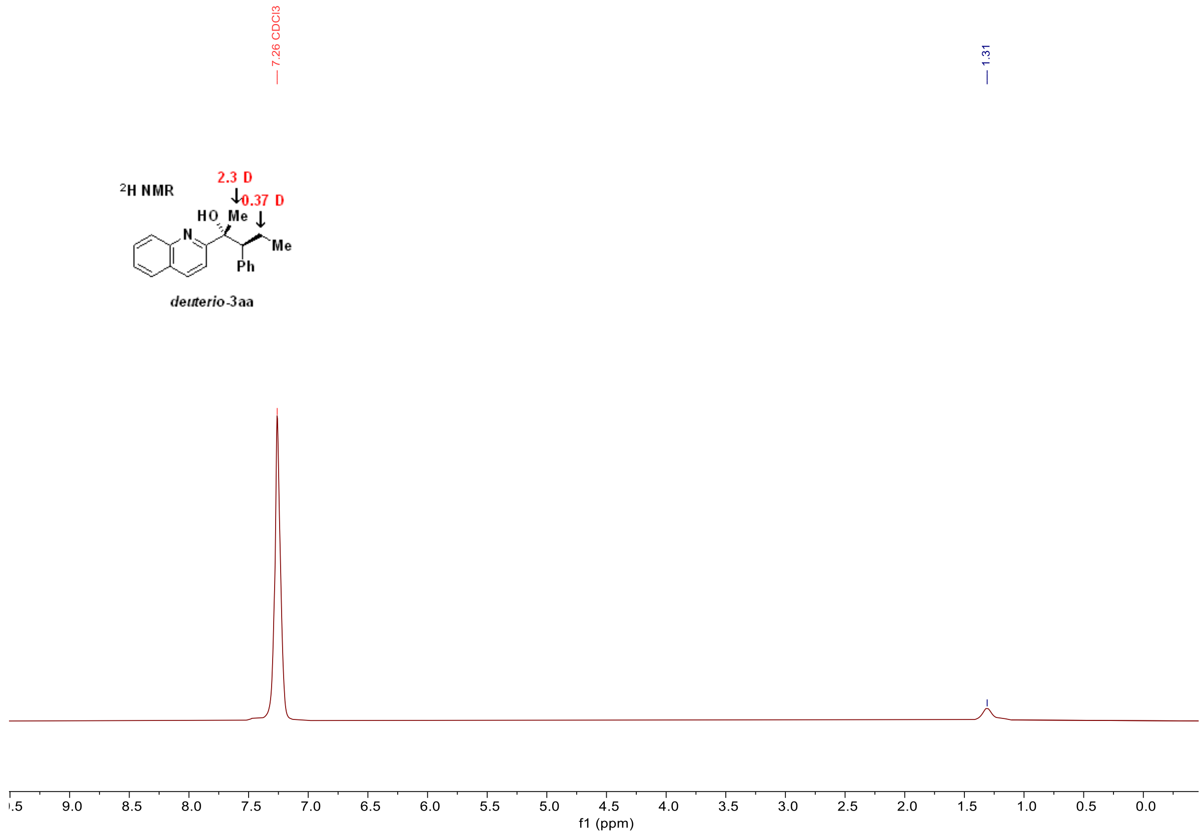

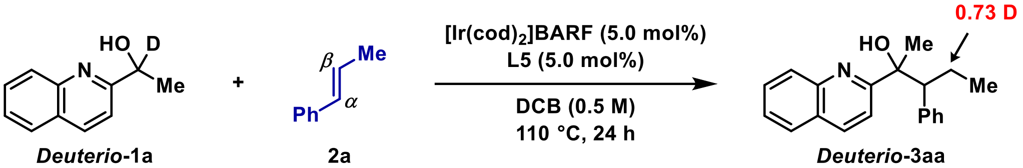

Scheme S22. Deuterium labelling experiment with *deuterio*-**1a** and **2a**.

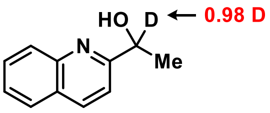

***Deuterio*-1a**

GP 3: *Deuterio*-**1a** (97% *D*) was prepared from 1-(quinolin-2-yl)ethan-1-one (**1a–H$_2$**, 171 mg, 1.0 mmol, 100 mol%) and $NaBD_4$ (62.8 mg, 1.5 mmol, 150 mol%) in MeOH (10 mL, 0.1 M) at r.t. for 1 h. Purification by flash column chromatography on silica gel (hexane/ethyl acetate from 100/1 to 3/1) afforded *Deuterio*-**1a** (148 mg, 85% yield, 97% *D*) as a colorless solid.

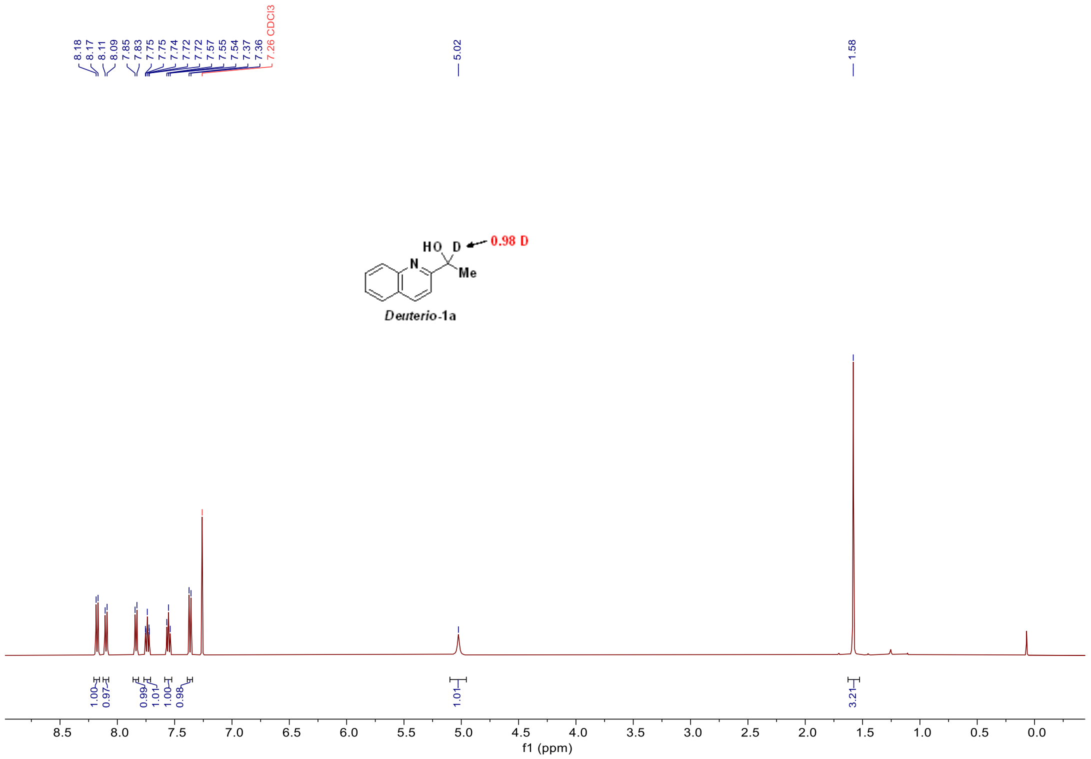

^{1}H NMR of *deuterio*-**1a** in $CDCl_3$

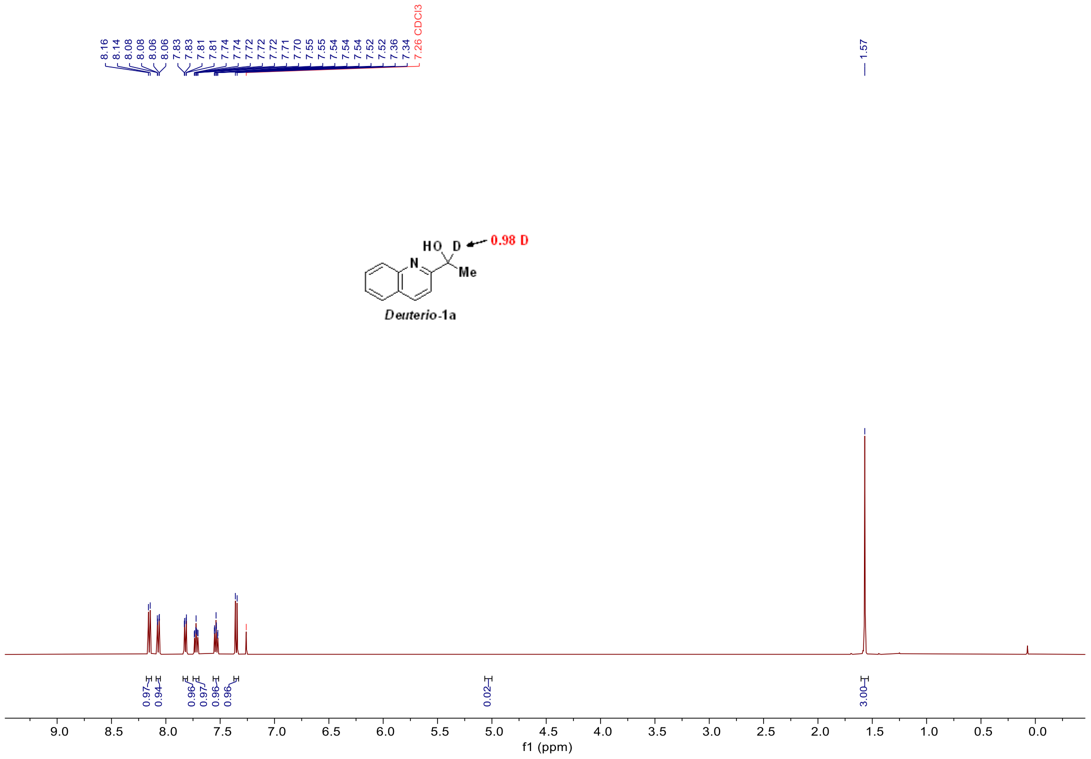

^{1}H NMR of *deuterio*-**1a** with D_2O in $CDCl_3$

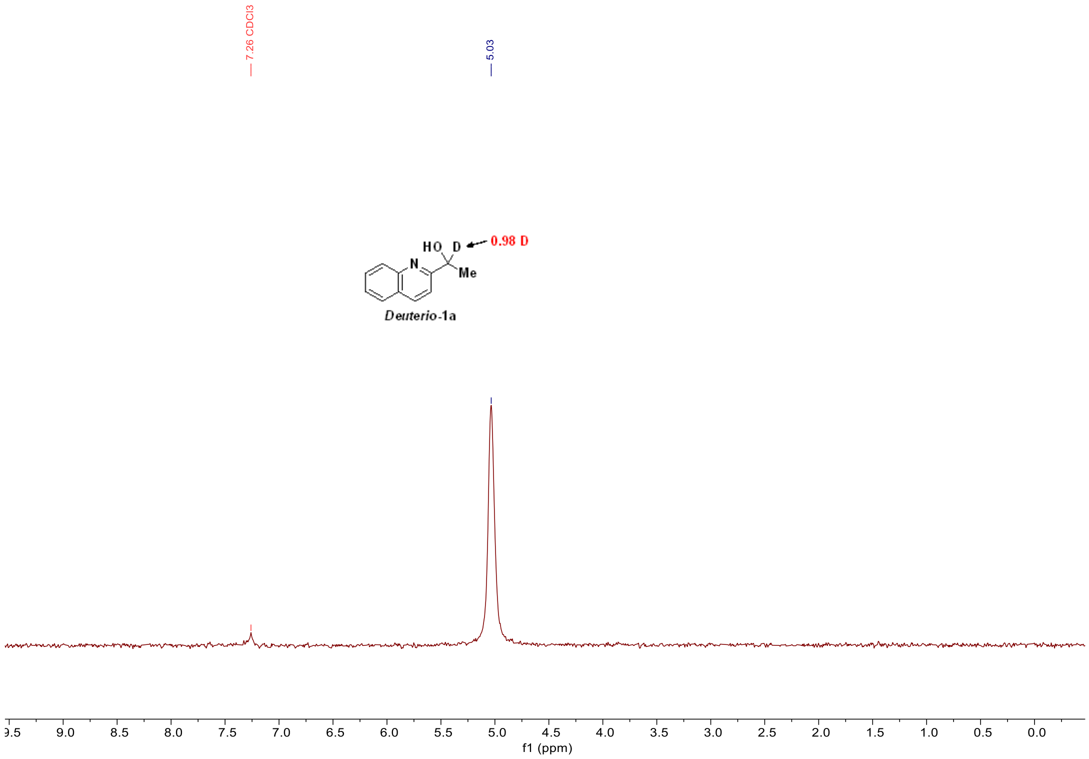

^{2}H NMR of *deuterio*-**1a** in $CHCl_3$

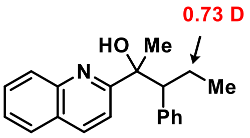

***Deuterio*-3aa**

GP6: A flame-dried Schlenk tube was charged with *deuterio*-1-(quinolin-2-yl)ethan-1-ol (*deuterio*-**1a**, 17.3 mg, 0.10 mmol, 100 mol%, 0.98 D), [Ir(cod)$_2$]BARF (6.36 mg, 5.0 μmol, 5.0 mol%) and **L5** ((*R*)-DM-SEGPHOS, 3.61 mg, 5.0 μmol, 5.0 mol%). The Schlenk tube was evacuated and backfilled with N_2 for three times. Then DCB (0.2 mL, 0.5 M) was added, followed by the addition of (*E*)-1-propenylbenzene (**2a**, 32.4 μL, 29.5 mg, 0.25 mmol, 250 mol%). The tube was sealed and heated at 110 °C for 24 h. After cooling to room temperature, the solvent was removed under reduced pressure. Purification by flash column chromatography on silica gel (hexane/ethyl acetate from 100/1 to 19/1) afforded ***deuterio*-3aa** (19.6 mg, 64% yield) as a colorless solid.

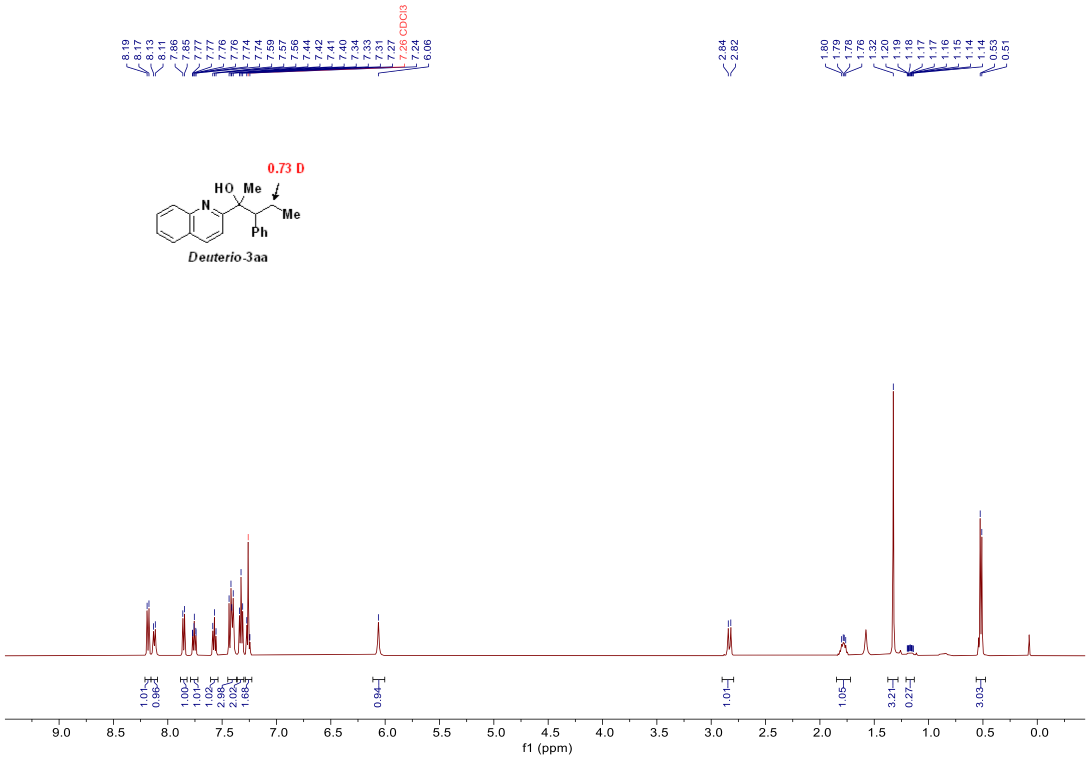

^{1}H NMR of *deuterio*-**3aa** in $CDCl_3$

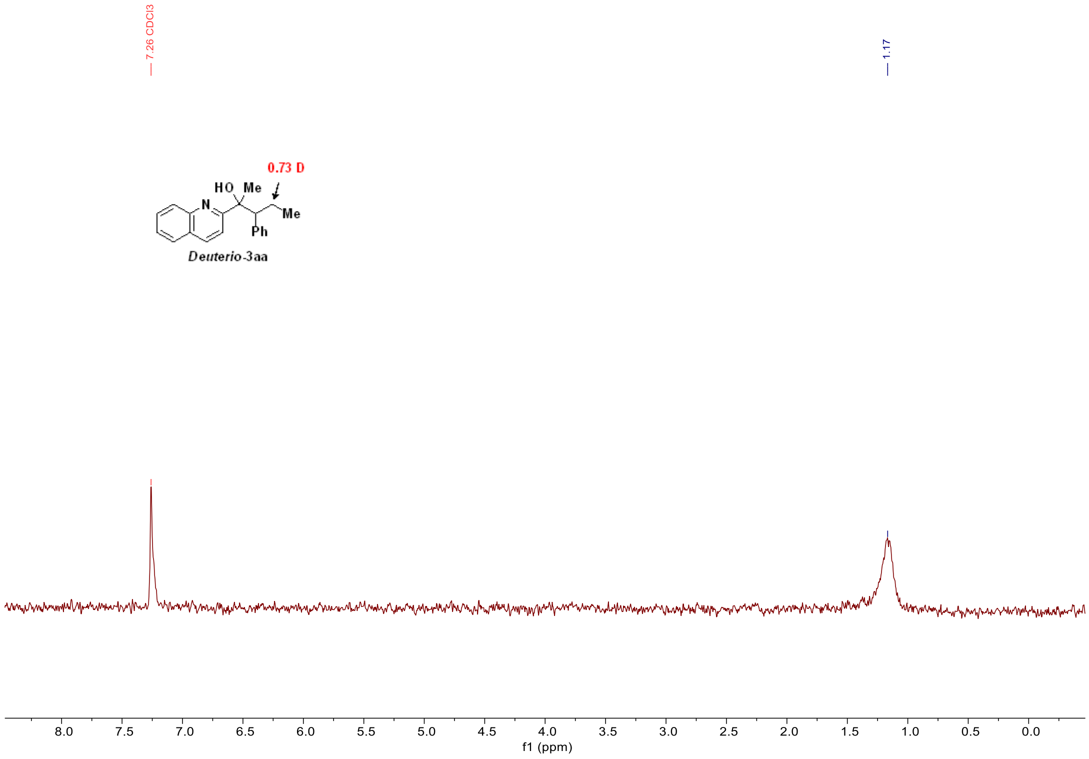

^{2}H NMR of *deuterio*-**3aa** in $CHCl_3$

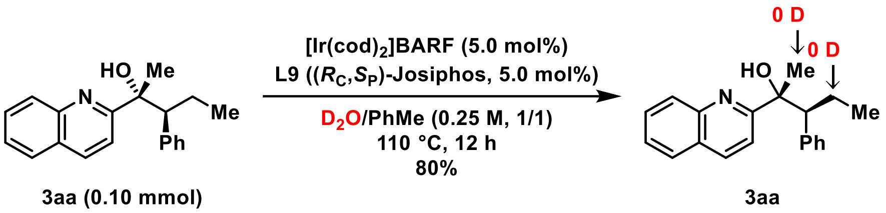

Scheme S23. Deuterium exchange experiment with **3aa**.

GP6: A flame-dried Schlenk tube was charged with (2*R*,3*S*)-3-phenyl-2-(quinolin-2-yl)pentan-2-ol (**3aa**, 29.1 mg, 0.10 mmol, 100 mol%), $[Ir(cod)_2]BARF$ (6.36 mg, 5.0 µmol, 5.0 mol%) and **L9** ((*R*C,*S*P)-Josiphos, 3.20 mg, 5.0 µmol, 5.0 mol%). The Schlenk tube was evacuated and backfilled with N_2 for three times, then toluene (0.2 mL, 0.5 M) and D_2O (0.2 mL, 0.5 M) was added. The tube was sealed and heated at 110 °C for 12 h. After cooling to room temperature, the solvent was removed under reduced pressure. Purification by flash column chromatography on silica gel (hexane/ethyl acetate from 100/1 to 19/1) afforded **3aa** (23.3 mg, 80% yield) as a colorless solid. The obtained products were analyzed by ^1H NMR using $CDCl_3$ as solvent.

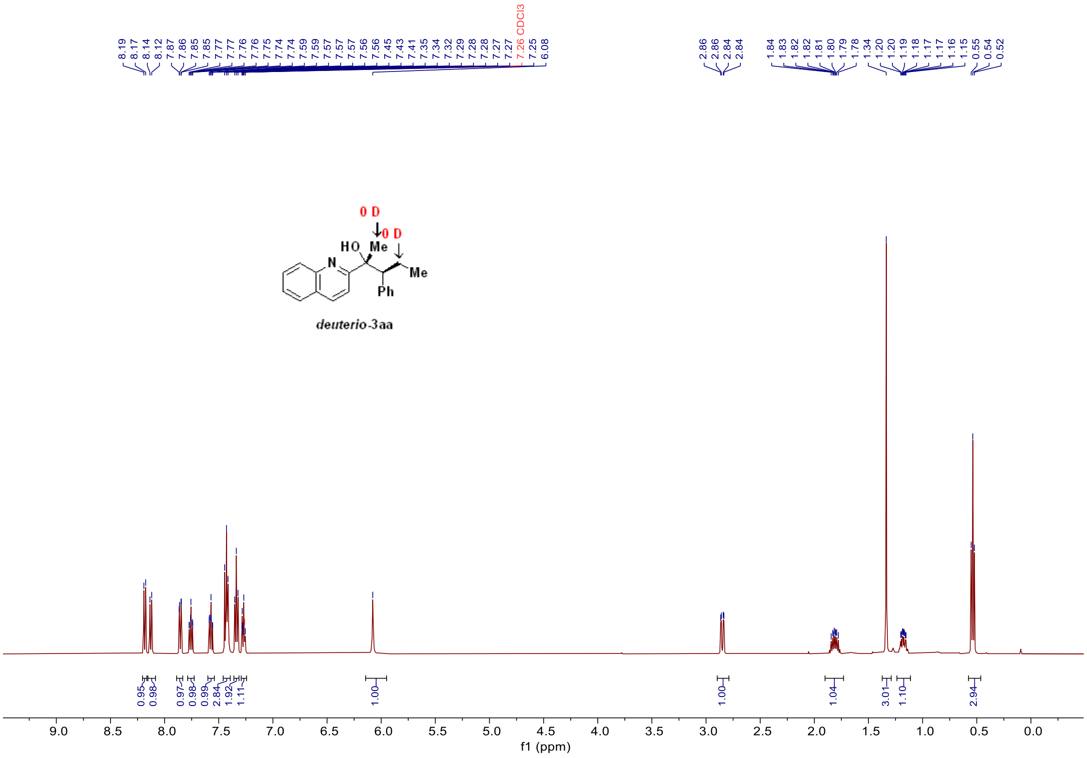

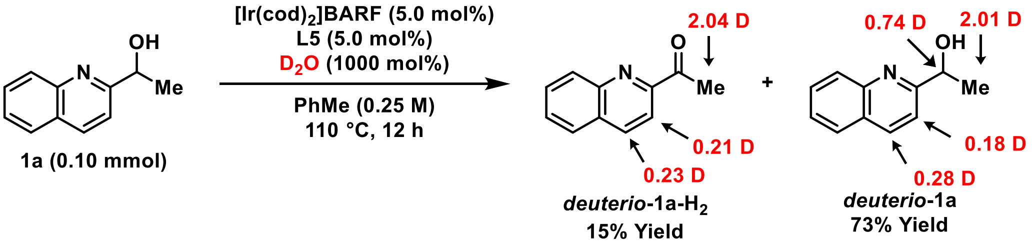

Scheme S24. Deuterium exchange experiment with **alcohol 1a**.

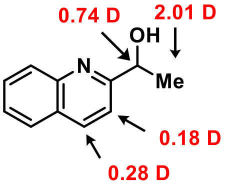

***deuterio*-1a**

GP6: A flame-dried Schlenk tube was charged with 1-(quinolin-2-yl)ethan-1-ol (**1a**, 17.3 mg, 0.10 mmol, 100 mol%), [Ir(cod)$_2$]BARF (6.36 mg, 5.0 μmol, 5.0 mol%) and **L5** ((*R*)-DM-SEGPHOS, 3.61 mg, 5.0 μmol, 5.0 mol%). The Schlenk tube was evacuated and backfilled with N_2 for three times, then toluene (0.2 mL) and D_2O (18 μL, 20.0 mg, 1.0 mmol, 1000 mol%) was added. The tube was sealed and heated at 110 °C for 12 h. After cooling to room temperature, the solvent was removed under reduced pressure. Purification by flash column chromatography on silica gel (hexane/ethyl acetate from 100/1 to 19/1) afforded ***deuterio*-1a** (12.6 mg, 73% yield) as a colorless solid and ***deuterio*-1a-H$_2$** (2.5 mg, 15% yield) as a colorless solid. The obtained products were analyzed by ^{1}H NMR and ^{2}H NMR spectroscopy using $CDCl_3$ as solvent.

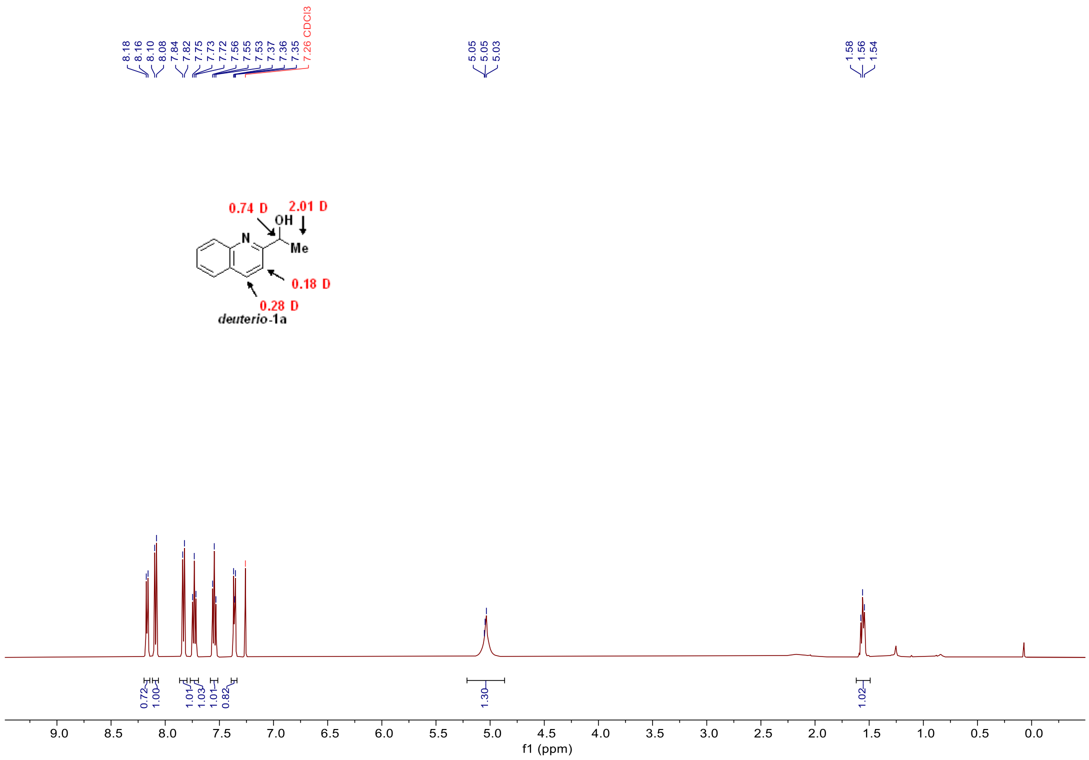

^{1}H NMR of *deuterio*-**1a** in $CDCl_3$

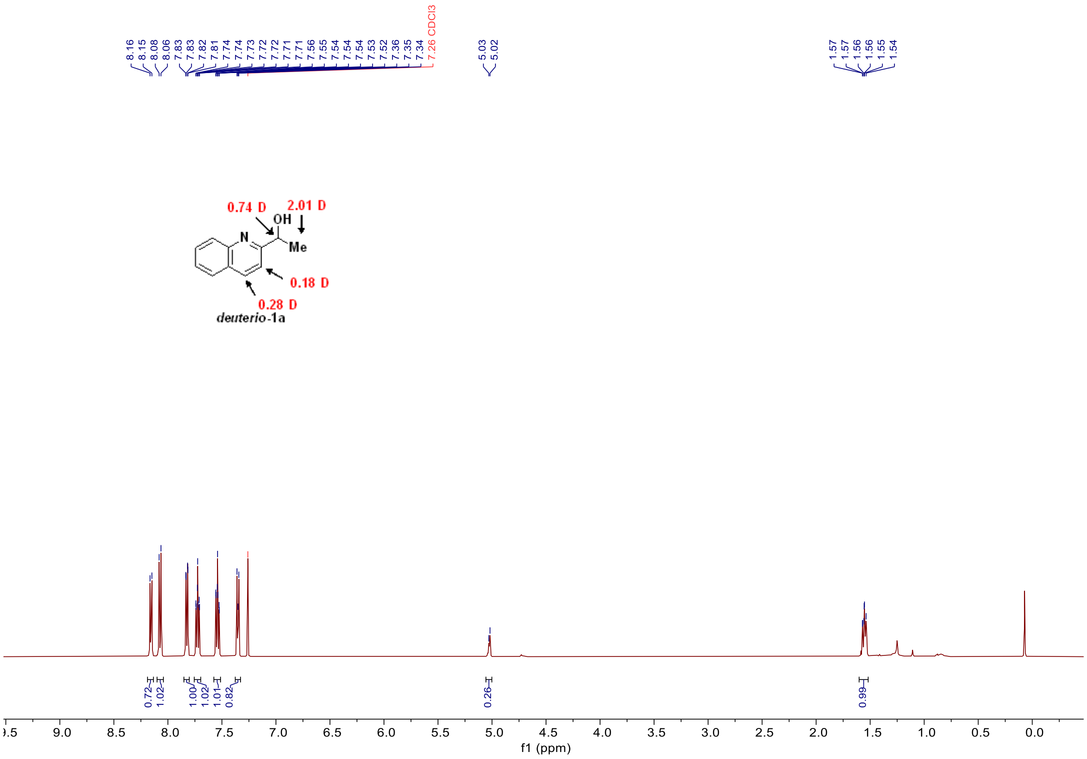

^{1}H NMR of *deuterio*-**1a** with D_2O in $CDCl_3$

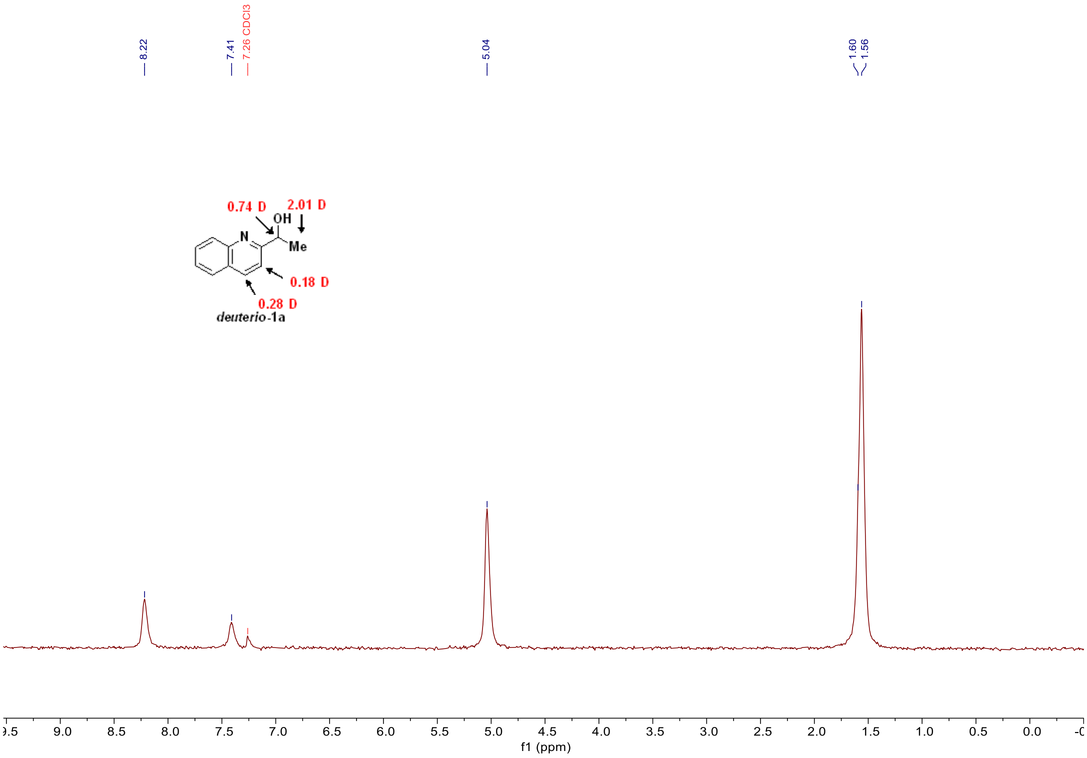

^{2}H NMR of *deuterio*-**1a** in $CHCl_3$

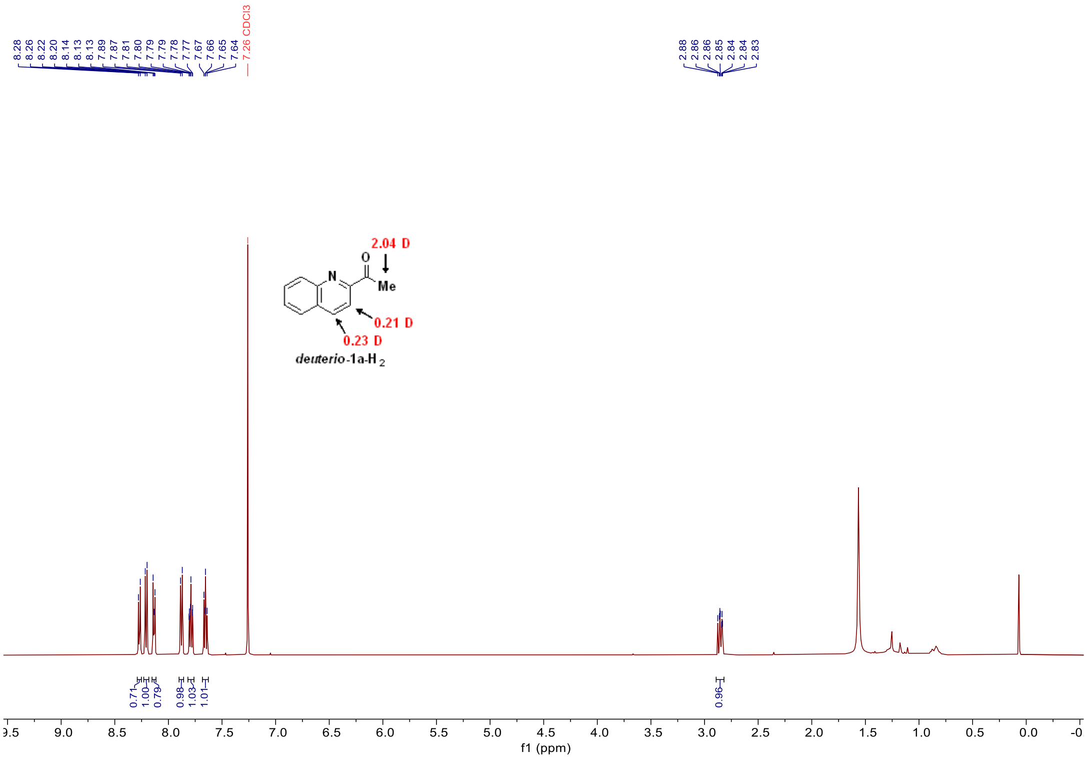

^{1}H NMR of *deuterio*-**1a–H$_2$** in $CDCl_3$

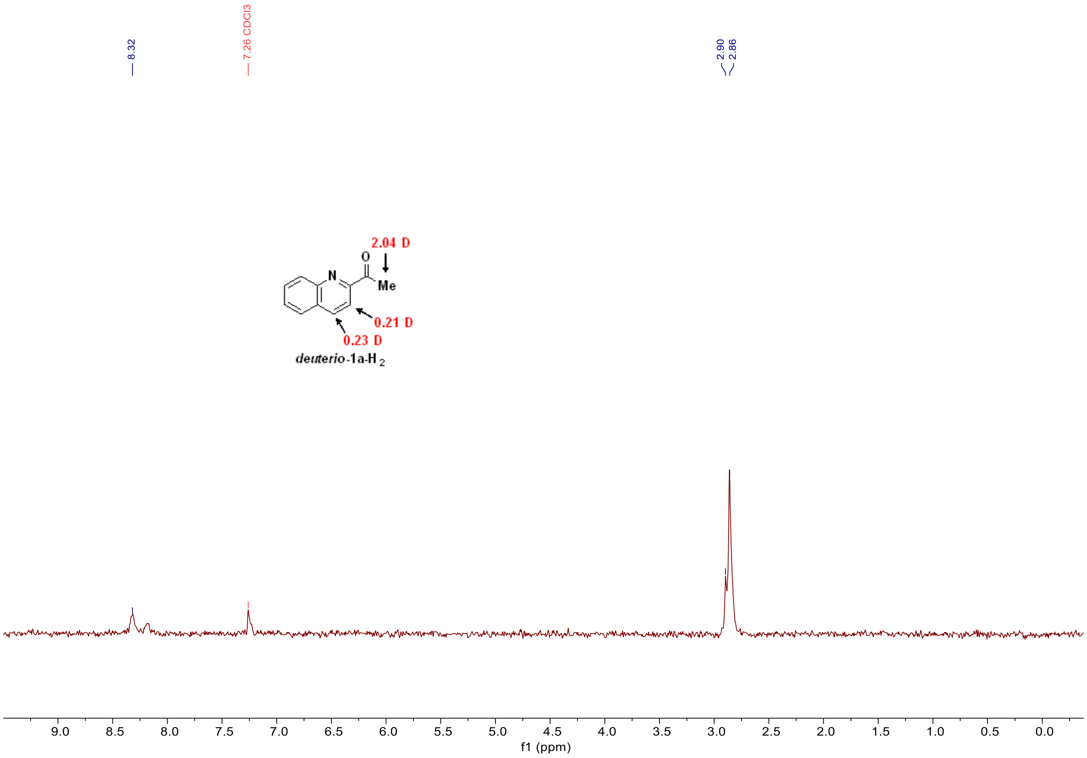

^{2}H NMR of *deuterio*-**1a** in $CHCl_3$

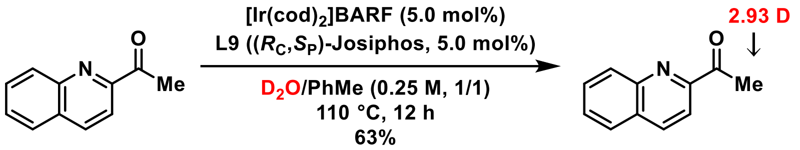

1a-H$_2$ (0.10 mmol) ***deuterio*-1a-H$_2$**

Scheme S25. Deuterium exchange experiment with **ketone 1a-H$_2$**.

GP6: A flame-dried Schlenk tube was charged with 1-(quinolin-2-yl)ethan-1-one (**1a-H$_2$**, 17.1 mg, 0.10 mmol, 100 mol%), [Ir(cod)$_2$]BARF (6.36 mg, 5.0 µmol, 5.0 mol%) and **L9** ((R_C,S_P)-Josiphos, 3.20 mg, 5.0 µmol, 5.0 mol%). The Schlenk tube was evacuated and backfilled with N_2 for three times, then toluene (0.2 mL) and D_2O (0.2 mL) was added. The tube was sealed and heated at 110 °C for 12 h. After cooling to room temperature, the solvent was removed under reduced pressure. Purification by flash column chromatography on silica gel (hexane/ethyl acetate from 100/1 to 19/1) afforded ***deuterio*-1a-H$_2$** (10.9 mg, 63% yield) as a colorless solid. The obtained products were analyzed by ^{1}H NMR and ^{2}H NMR spectroscopy using $CDCl_3$ as solvent.

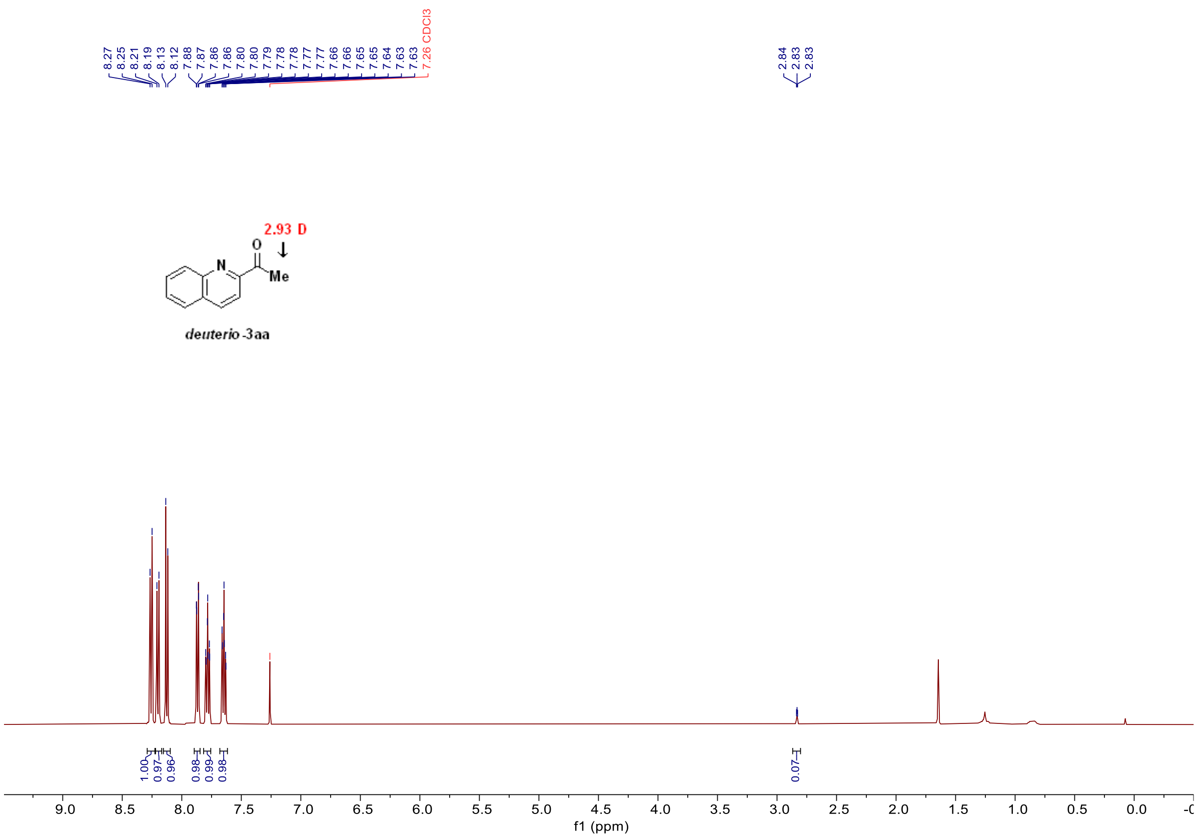

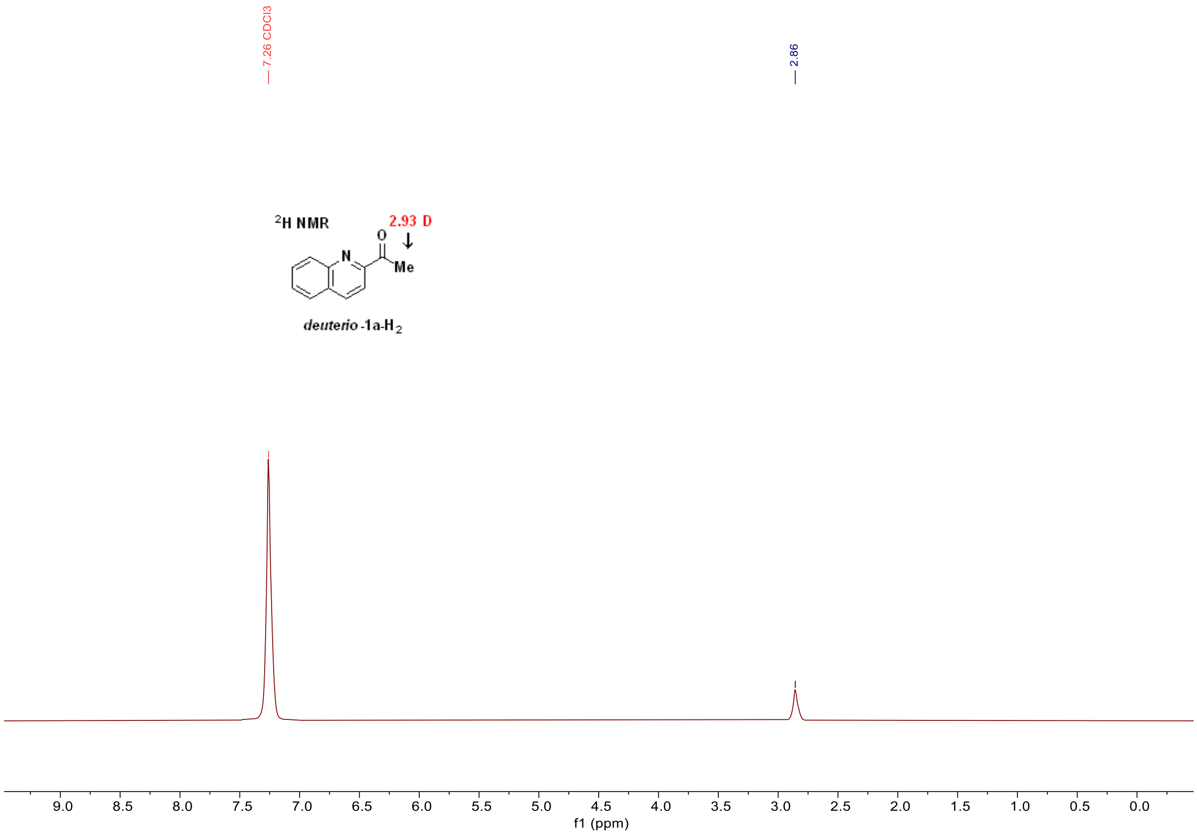

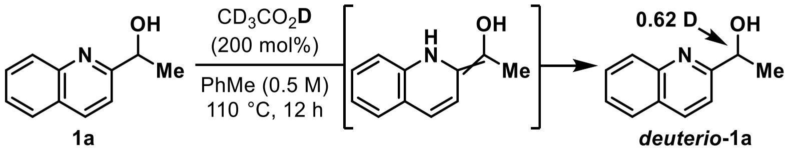

Scheme S26. Aza-enolization is viable under acidic conditions.

The reaction was conducted according to a modified porcedure.[12] A flame-dried Schlenk tube was charged with 1-(quinolin-2-yl)ethan-1-ol (**1a**, 17.3 mg, 0.10 mmol, 100 mol%). The Schlenk tube was evacuated and backfilled with N_2 for three times. Then toluene (0.2 mL, 0.5 M) was added, followed by the addition of CD_3CO_2D (11.5 μL, 12.2 mg, 0.20 mmol, 200 mol%). The tube was sealed and heated at 110 °C for 12 hours. After cooling to room temperature, the solvent was removed under reduced pressure. Purification by flash column chromatography on silica gel (hexane/ethyl acetate from 100/1 to 19/1) afforded ***deuterio*-1a** (14.5 mg, 84% yield) as a colorless solid. The obtained products were analyzed by ^1H NMR and ^2H NMR spectroscopy using $CDCl_3$ as solvent.

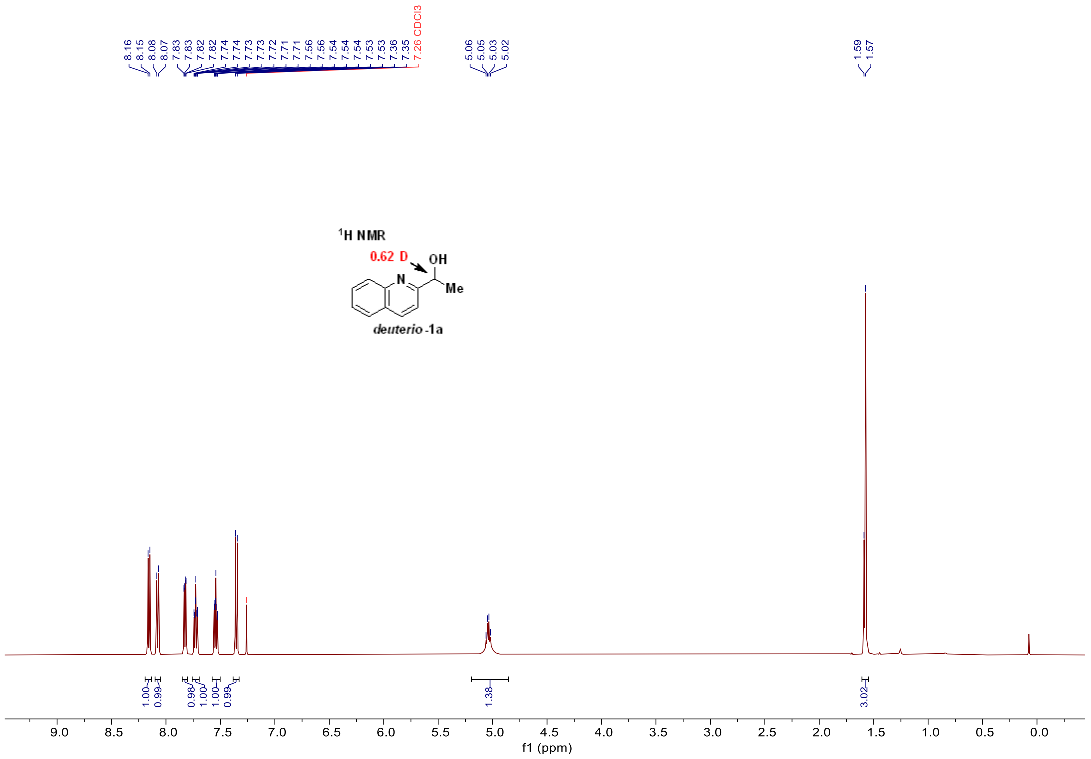

^{1}H NMR of *deuterio*-**1a** in $CDCl_3$

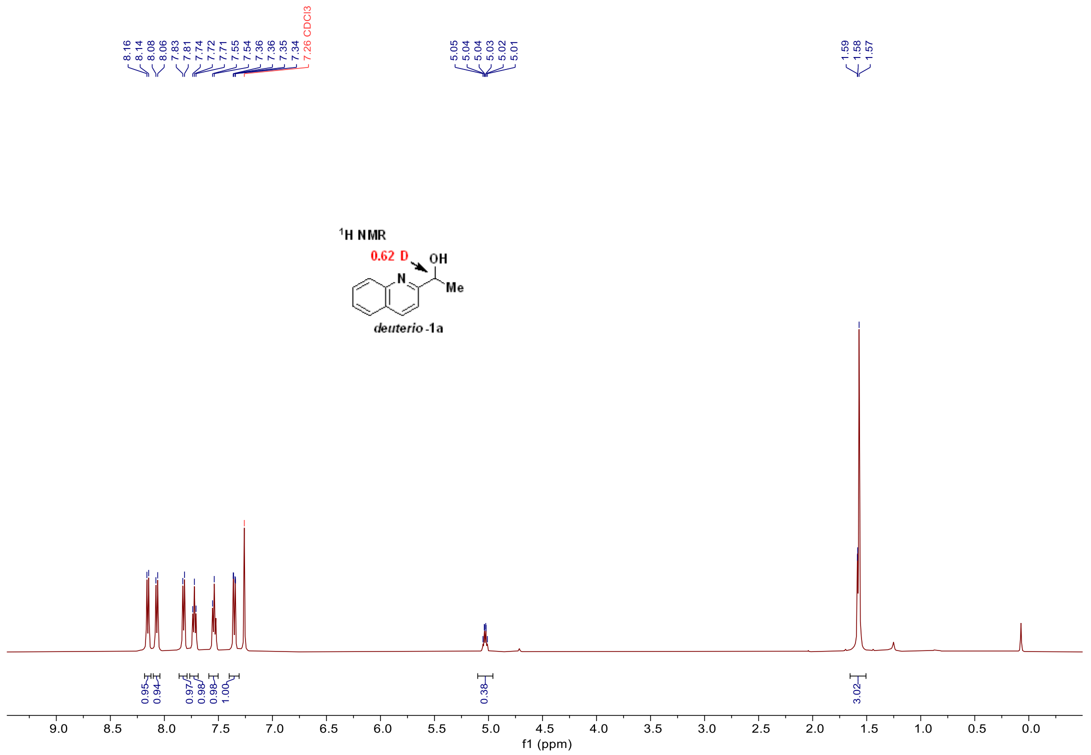

^{1}H NMR of *deuterio*-**1a** with D_2O in $CDCl_3$

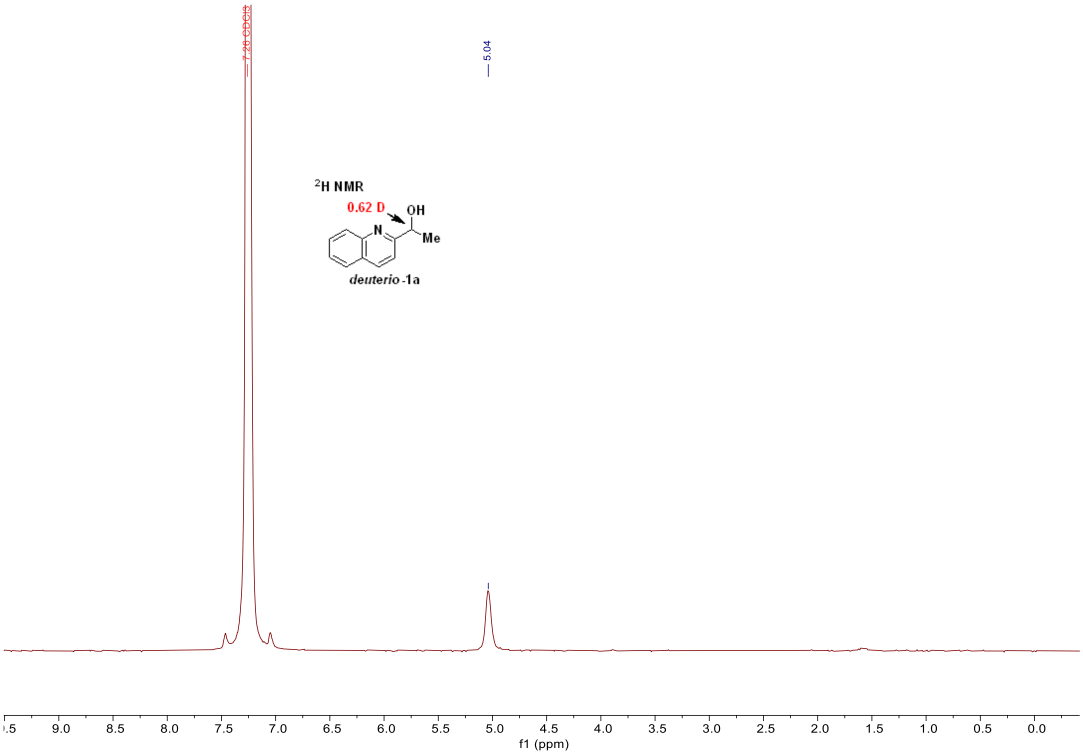
7.26 CDCl3
5.04
2H NMR
0.62 D
OH
N
Me
deuterio-1a
9.0
8.5
8.0
7.5
7.0
6.5
6.0
5.5
5.0
4.5
4.0
3.5
3.0
2.5
2.0
1.5
1.0
0.5
0.0
f1 (ppm)

6.3 Visual Kinetic Analysis

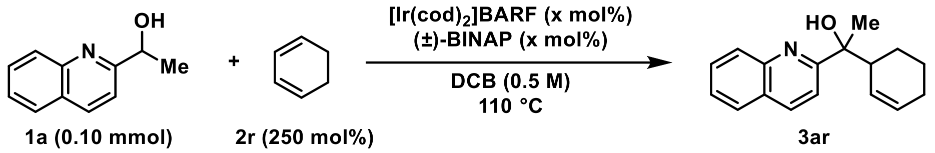

Scheme S27. Experiments about visual kinetic analysis using **1a** and **2r**.

The reaction kinetic was determined using Burés' normalized time scale method.[13] Five parallel experiments of 1-(quinolin-2-yl)ethan-1-ol (**1a**, 17.3 mg, 0.10 mmol, 100 mol%) and 1,3-cyclohexadiene (**2r**, 23.7 μL, 20.0 mg, 0.25 mmol, 250 mol%) were conducted, using [Ir(cod)$_2$]BARF (6.36 mg, 5.0 μmol, 5.0 mol%) and (±)-BINAP (3.11 mg, 5.0 μmol, 5.0 mol%) in DCB (0.2 mL). The concentrations of **3ar** at 0 h, 0.5 h, 1 h, 2 h and 2.5 h were determined by ^{1}H NMR analysis using 1,3,5-trimethoxybenzene as the internal standard. Another five parallel experiments used [Ir(cod)$_2$]BARF (8.90 mg, 7.0 μmol, 7.0 mol%) and (±)-BINAP (4.36 mg, 7.0 μmol, 7.0 mol%). The concentrations of **3ar** at 0 h, 0.5 h, 1 h, 1.5 h and 2 h were determined by ^{1}H NMR analysis using 1,3,5-trimethoxybenzene as the internal standard. The results are listed as below:

5 mol% [Ir(cod)$_2$]BARF 0.01923 mol/L		7 mol% [Ir(cod)$_2$]BARF 0.02593 mol/L	
time (h)	concentration (**3ar**, mol/L)	time (h)	concentration (**3ar**, mol/L)
0	0	0	0
0.5	0.075	0.5	0.09643
1	0.1464	1	0.1821
2	0.2607	1.5	0.2607
2.5	0.3	2	0.3393

Graphical kinetic analysis: the order in catalyst ([Ir(cod)$_2$]BARF/(±)-BINAP) is approximately **1**.

Experiment 1;	[Cat] =	0.025	5 mol%	[Ir] (0.025 M)	Order in cat =	0.5	change this value to check other orders
time (h)		[3ar]	[cat]^l2	t[cat]^l2			
	0	0	0.158114	0			
	0.5	0.075	0.158114	0.079057			
	1	0.1464	0.158114	0.158114			
	2	0.2607	0.158114	0.316228			
	2.5	0.3	0.158114	0.395285			

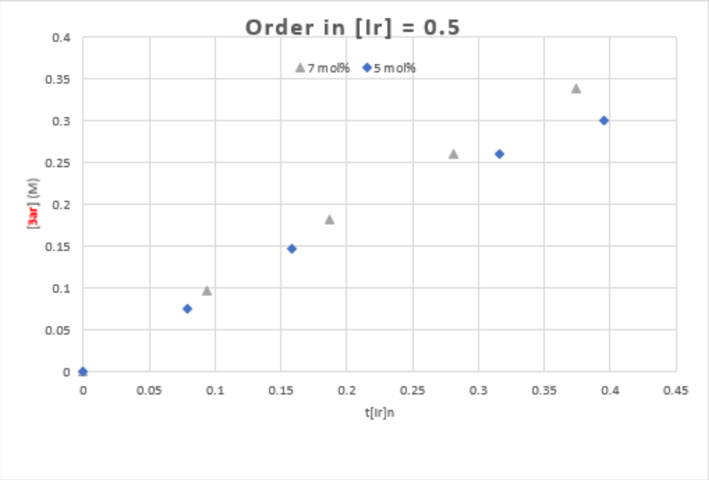

Experiment 2;	[Cat] =	0.035	7 mol%	[Ir] (0.035 M)
time (h)		[3ar]	[cat]^l2	t[cat]^l2
	0	0	0.187083	0
	0.5	0.09643	0.187083	0.093541
	1	0.1821	0.187083	0.187083
	1.5	0.2607	0.187083	0.280624
	2	0.3393	0.187083	0.374166

Experiment 1;	[Cat] =	0.025	5 mol%	[Ir] (0.025 M)	Order in cat =	1	change this value to check other orders
time (h)		[3ar]	[cat]^l2	t[cat]^l2			
	0	0	0.025	0			
	0.5	0.075	0.025	0.0125			
	1	0.1464	0.025	0.025			
	2	0.2607	0.025	0.05			
	2.5	0.3	0.025	0.0625			

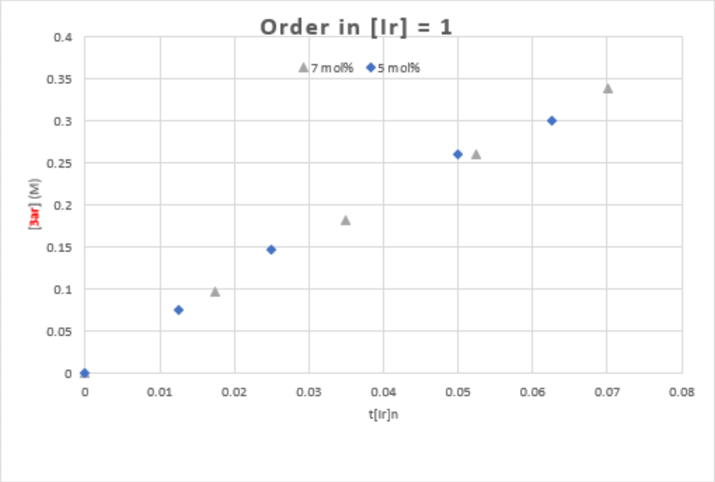

Experiment 2;	[Cat] =	0.035	7 mol%	[Ir] (0.035 M)
time (h)		[3ar]	[cat]^l2	t[cat]^l2
	0	0	0.035	0
	0.5	0.09643	0.035	0.0175
	1	0.1821	0.035	0.035
	1.5	0.2607	0.035	0.0525
	2	0.3393	0.035	0.07

Experiment 1;	[Cat] =	0.025	5 mol%	[Ir] (0.025 M)	Order in cat =	1.5	change this value to check other orders
time (h)		[3ar]	[cat]^I2	t[cat]^I2			
0		0	0.003953	0			
0.5		0.075	0.003953	0.001976			
1		0.1464	0.003953	0.003953			
2		0.2607	0.003953	0.007906			
2.5		0.3	0.003953	0.009882			

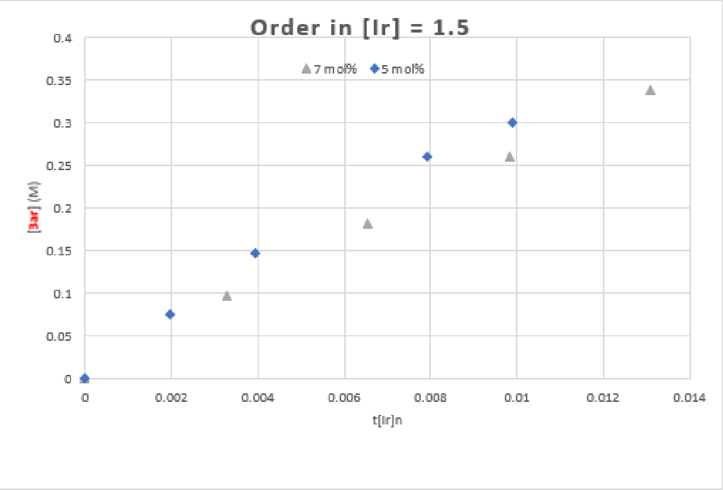

Experiment 2;	[Cat] =	0.035	7 mol%	[Ir] (0.035 M)
time (h)		[3ar]	[cat]^I2	t[cat]^I2
0		0	0.006548	0
0.5		0.09643	0.006548	0.003274
1		0.1821	0.006548	0.006548
1.5		0.2607	0.006548	0.009822
2		0.3393	0.006548	0.013096

6.4 Preparation of 7

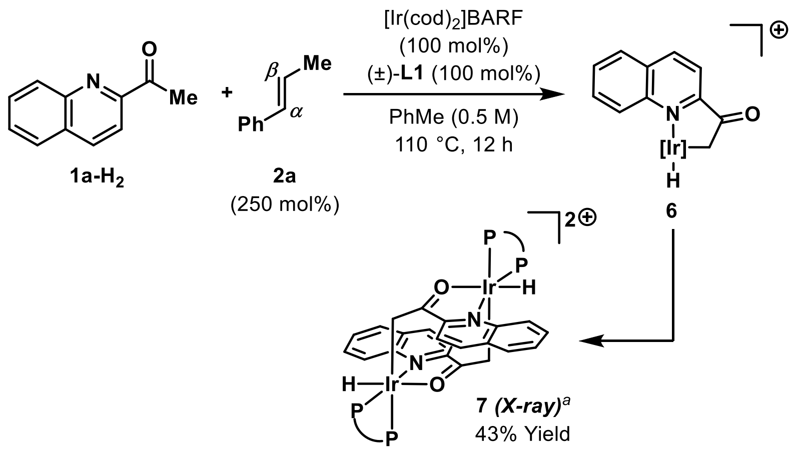

Scheme S28. Preparation of **7**. For clarity, the P,P-ligands ((±)-**L1**) are simplified and the BARF counterions are omitted on the structure of **7.**

7 (**GP6**) was prepared from 1-(quinolin-2-yl)ethan-1-one (**1a**, 17.1 mg, 0.10 mmol, 100 mol%) and (*E*)-1-propenylbenzene (**2a**, 32.4 μL, 29.5 mg, 0.25 mmol, 250 mol%), using [Ir(cod)$_2$]BARF (127 mg, 0.10 mmol, 100 mol%) and (±)-**L1** ((±)-BINAP, 62.3 mg, 0.10 mmol,, 100 mol%) in toluene (0.2 mL) at 110 °C for 12 h. The reaction mixture was filtered and washed by toluene to afford **7** (79.5 mg, 43% yield) as a yellow solid.

Characteristic signals for **7**

^{1}H NMR (400 MHz, DCM-*d*$_2$, 298 K): δ 9.48 (d, *J* = 8.9 Hz, 2H, Ar), 4.13–4.05 (m, 1H, Ir–CH$_2$), 3.52–3.44 (m, 2H, Ir–CH$_2$), –23.30 (d, *J* = 8.4 Hz, 1H, Ir–H), –23.38 (d, *J* = 8.4 Hz, 1H, Ir–H) ppm.

Crystal **7** was obtained through recrystallization in the solution of DCM and methanol at room temperature. CCDC 2480114 contains the supplementary crystallographic data for this compound.

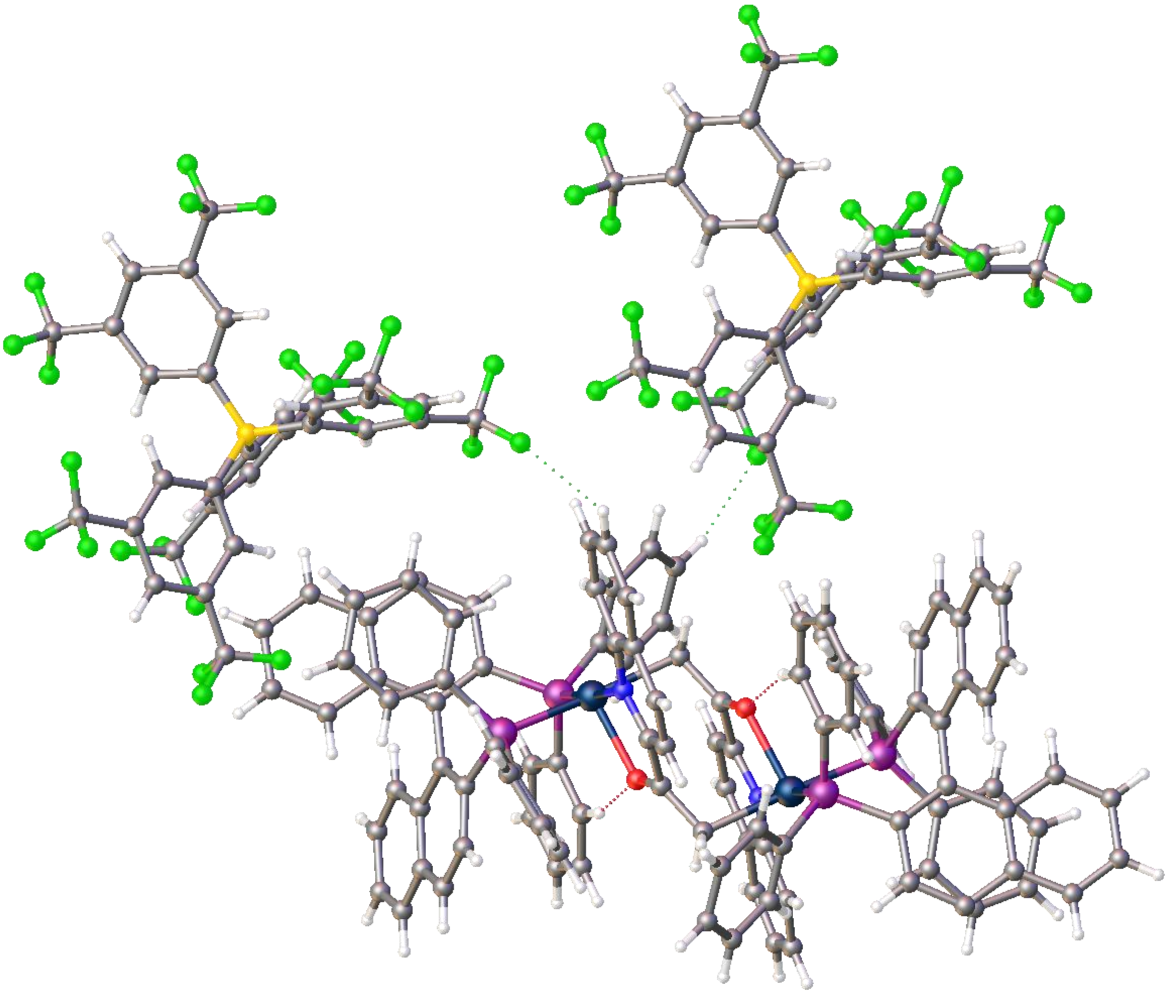

Figure S6. Molecular structure of **7**. Thermal ellipsoids represent 50% probability level. Due to large Ir absorption, the hydride ligand could not be modelled; however, it is observed in the ^{1}H NMR spectrum (see above).

Experimental and tables for **7**

exp_1880_auto

Table 1 Crystal data and structure refinement for exp_1880_auto.

Identification code	exp_1880_auto
Empirical formula	$C_{87}H_{51}BF_{24.02}IrNOP_2$
Formula weight	1847.61
Temperature/K	100.0(5)
Crystal system	triclinic
Space group	P-1

a/Å	14.18320(10)
b/Å	17.0432(2)
c/Å	17.74540(10)
α/°	102.7360(10)
β/°	107.1920(10)
γ/°	90.7360(10)
Volume/Å^3	3983.03(6)
Z	2
ρ_{calc}g/cm^3	1.541
μ/mm^{-1}	1.820
F(000)	1832.0
Crystal size/mm^3	0.12 × 0.01 × 0.01
Radiation	Mo Kα (λ = 0.71073)
2Θ range for data collection/°	3.262 to 53
Index ranges	-17 ≤ h ≤ 17, -21 ≤ k ≤ 21, -22 ≤ l ≤ 22
Reflections collected	162727
Independent reflections	16505 [R_{int} = 0.1001, R_{sigma} = 0.0409]
Data/restraints/parameters	16505/0/1092
Goodness-of-fit on F^2	1.048
Final R indexes [I>=2σ (I)]	R_1 = 0.0343, wR_2 = 0.0858
Final R indexes [all data]	R_1 = 0.0364, wR_2 = 0.0871
Largest diff. peak/hole / e Å^{-3}	1.79/-1.50

Table 2 Fractional Atomic Coordinates (×10^4) and Equivalent Isotropic Displacement Parameters (Å^2×10^3) for exp_1880_auto. U_{eq} is defined as 1/3 of of the trace of the orthogonalised U_{IJ} tensor.

**Atom**	***x***	***y***	***z***	**U(eq)**
Ir(1)	9367.7(2)	9864.5(2)	6188.8(2)	10.61(4)

Table 2 Fractional Atomic Coordinates (×10⁴) and Equivalent Isotropic Displacement Parameters (Å²×10³) for exp_1880_auto. U_{eq} is defined as 1/3 of of the trace of the orthogonalised U_{IJ} tensor.

Atom	*x*	*y*	*z*	U(eq)
$P^{(1)}$	7734.6(6)	10041.7(4)	6173.4(4)	12.24(15)
$P^{(2)}$	9924.2(6)	10731.7(4)	7425.4(4)	12.13(15)
$O^{(1)}$	9269.3(15)	10539.1(12)	5264.8(12)	13.0(4)
$N^{(1)}$	8881.7(18)	8971.7(14)	5068.4(14)	12.3(5)
$F^{(14)}$	3781.7(17)	8180.5(13)	9097.2(15)	40.4(6)
$F^{(13)}$	4545.0(19)	8819.3(13)	8513.0(14)	39.0(6)
$F^{(21)}$	3531(2)	4835.8(17)	10357.3(14)	50.5(7)
$F^{(19)}$	3319(2)	5890.1(16)	9888.8(17)	56.4(8)
$F^{(20)}$	2232(2)	4885(2)	9388.0(17)	61.4(8)
$C^{(1)}$	8700(2)	9258.0(18)	4400.1(17)	13.8(6)
$F^{(91)}$	4089(2)	2267.9(14)	7959.7(18)	55.0(7)
$F^{(30)}$	7456(2)	5087.5(19)	5384.0(16)	56.4(8)
$F^{(22)}$	5128(3)	2630.4(16)	9107.0(16)	70.9(11)
$C^{(39)}$	8285(2)	10838.3(17)	8016.4(17)	14.7(6)
$C^{(10)}$	9024(2)	10132.7(17)	4542.1(17)	12.1(6)
$C^{(11)}$	9149(2)	10458.4(18)	3896.8(17)	14.2(6)
$F^{(29)}$	3213(3)	7479(2)	5479(3)	73.9(17)
$F^{(24)}$	5512(3)	2678.8(17)	8039(3)	83.1(13)
$C^{(57)}$	6469(2)	10070(2)	3765.1(19)	20.0(7)
$C^{(24)}$	9936(2)	11809.1(17)	7490.2(18)	15.1(6)
$C^{(49)}$	7171(2)	9392.2(17)	6640.5(17)	15.5(6)
$C^{(40)}$	7854(2)	11374.8(18)	7456.5(18)	15.2(6)
$C^{(48)}$	7483(2)	11070.1(17)	6624.9(18)	14.3(6)
$C^{(38)}$	7694(2)	10633.0(17)	8489.1(17)	15.9(6)
$C^{(25)}$	10183(2)	12361.7(19)	8243.6(19)	19.5(6)

Table 2 Fractional Atomic Coordinates (×10^4) and Equivalent Isotropic Displacement Parameters (Å^2×10^3) for exp_1880_auto. U_{eq} is defined as 1/3 of of the trace of the orthogonalised U_{IJ} tensor.

Atom	*x*	*y*	*z*	U(eq)
F(32)	1612(2)	7514(2)	5267(2)	77.9(16)
C(30)	9210(2)	10551.9(17)	8092.2(17)	15.3(6)
C(18)	11200(2)	10581.6(19)	7985.9(17)	16.4(6)
C(55)	6930(2)	9809.1(18)	5113.0(17)	14.1(6)
C(23)	11463(2)	9794.8(19)	7993.3(17)	17.0(6)
C(50)	7687(2)	8803.8(19)	6991.9(19)	18.9(6)
C(46)	7317(3)	12714.2(19)	7300(2)	20.2(7)
C(9)	8709(2)	8152.4(18)	4978.1(18)	15.8(6)
C(2)	8249(2)	8777.2(19)	3614.7(18)	17.3(6)
F(33)	7658(2)	5908.1(19)	6520(2)	70.9(10)
C(31)	9563(2)	10042.2(18)	8635.8(18)	17.3(6)
C(19)	11939(2)	11217.6(19)	8332.1(17)	18.0(6)
C(4)	8278(2)	7636.8(19)	4194.8(19)	18.7(6)
F(31)	4896(3)	2524.3(18)	5041(2)	47.5(12)
C(41)	7775(2)	12201.3(18)	7812.5(19)	17.2(6)
C(56)	7004(2)	10297.9(19)	4587.8(18)	16.9(6)
C(29)	9691(2)	12092.6(18)	6783.9(19)	17.5(6)
C(47)	6975(2)	11582.3(19)	6134.3(18)	17.9(6)
C(32)	9007(2)	9831.7(19)	9081.2(18)	19.4(6)
C(20)	12923(2)	11063(2)	8660.7(18)	20.9(7)
C(92)	1549(3)	6305(2)	6056(2)	24.1(7)
F(18)	8086(3)	6183(2)	9184(2)	39.4(14)
C(3)	8026(2)	7975(2)	3515.3(19)	21.2(7)
C(93)	2445(3)	6651.5(19)	6073.2(19)	21.1(7)
C(8)	8985(2)	7807.9(19)	5657.8(19)	19.1(6)

Table 2 Fractional Atomic Coordinates (×10^4) and Equivalent Isotropic Displacement Parameters (Å^2×10^3) for exp_1880_auto. U_{eq} is defined as 1/3 of of the trace of the orthogonalised U_{IJ} tensor.

**Atom**	***x***	***y***	***z***	**U(eq)**
C(54)	6200(2)	9481.8(19)	6648(2)	20.3(6)
C(33)	8066(2)	10120.5(18)	9027.4(18)	17.8(6)
C(60)	6337(2)	9084.4(19)	4792.9(19)	18.9(6)
C(94)	3336(2)	6369.7(19)	6455.8(18)	18.6(6)
C(42)	8150(3)	12540(2)	8661(2)	22.8(7)
C(01Q)	6906(3)	12376(2)	6463(2)	21.8(7)
F(28)	2616(3)	8081.6(17)	6331(2)	58.4(14)
F(26)	8019(2)	4706(3)	6469(3)	88.9(13)
C(37)	6748(3)	10913.7(19)	8437.6(19)	20.3(7)
C(76)	4627(2)	4108.0(18)	7910.9(19)	18.3(6)
C(87)	4675(2)	6983.3(19)	8251.0(18)	19.2(6)
C(71)	4511(2)	4342.7(18)	6104.1(18)	18.5(6)
C(5)	8124(3)	6795(2)	4124(2)	26.1(7)
C(21)	13174(3)	10279(2)	8647.8(19)	22.0(7)
C(34)	7493(3)	9932(2)	9504.1(19)	22.5(7)
C(51)	7231(3)	8315(2)	7334(2)	23.0(7)
C(59)	5805(3)	8861(2)	3972(2)	23.8(7)
C(74)	6330(3)	4897.2(19)	6081(2)	20.5(7)
C(70)	4986(2)	5044.5(18)	6689.7(18)	16.0(6)
C(88)	3371(2)	5744.7(17)	6862.7(17)	16.2(6)
C(58)	5879(3)	9352(2)	3459.7(19)	23.5(7)
C(89)	2461(2)	5408.6(18)	6839(2)	20.1(7)
C(80)	3849(2)	5177.1(19)	8529.5(19)	20.8(7)
C(75)	5898(2)	5311.1(18)	6647.9(19)	18.2(6)
C(22)	12443(2)	9642(2)	8324.0(18)	21.1(7)

Table 2 Fractional Atomic Coordinates (×10^4) and Equivalent Isotropic Displacement Parameters (Å^2×10^3) for exp_1880_auto. U_{eq} is defined as 1/3 of of the trace of the orthogonalised U_{IJ} tensor.

**Atom**	***x***	***y***	***z***	**U(eq)**
$C^{(28)}$	9704(3)	12925(2)	6835(2)	25.8(7)
$C^{(27)}$	9968(3)	13462(2)	7585(2)	26.9(8)
$C^{(73)}$	5847(3)	4208(2)	5513.1(19)	21.5(7)
$C^{(45)}$	7268(3)	13540(2)	7646(2)	27.3(8)
$C^{(35)}$	6592(3)	10226(2)	9449(2)	23.9(7)
$C^{(52)}$	6271(3)	8410(2)	7337(2)	24.3(7)
$C^{(90)}$	1565(3)	5677.4(19)	6442(2)	25.2(7)
$C^{(81)}$	4299(2)	4881.5(19)	7931.8(18)	17.8(6)
$C^{(53)}$	5752(3)	8993(2)	6996(2)	25.3(7)
$C^{(82)}$	5109(2)	6281.0(18)	8008.6(18)	16.7(6)
$C^{(43)}$	8083(3)	13342(2)	8974(2)	28.1(8)
$C^{(77)}$	4500(2)	3662.1(19)	8452(2)	21.7(7)
$C^{(26)}$	10210(3)	13187.1(19)	8287(2)	24.4(7)
$C^{(36)}$	6217(3)	10721(2)	8905(2)	22.9(7)
$C^{(86)}$	5213(3)	7641.2(19)	8853.5(19)	20.0(6)
$C^{(72)}$	4929(3)	3935.8(19)	5529.1(19)	20.2(7)
$C^{(78)}$	4050(3)	3970(2)	9035(2)	24.8(7)
$C^{(7)}$	8821(3)	6993(2)	5562(2)	27.0(8)
$C^{(101)}$	4831(3)	2825(2)	8398(2)	27.8(8)
$C^{(79)}$	3722(3)	4737(2)	9066(2)	24.2(7)
$F^{(23)}$	4451(4)	3177(2)	4165(2)	64.1(16)
$C^{(83)}$	6115(3)	6269(2)	8424.8(19)	21.9(7)
$C^{(84)}$	6660(3)	6934(2)	9000(2)	27.1(8)
$C^{(6)}$	8390(3)	6481(2)	4787(2)	29.1(8)
$F^{(25)}$	3518(3)	3005(3)	4890(3)	85(2)

Table 2 Fractional Atomic Coordinates (×10^4) and Equivalent Isotropic Displacement Parameters (Å^2×10^3) for exp_1880_auto. U_{eq} is defined as 1/3 of of the trace of the orthogonalised U_{IJ} tensor.

**Atom**	***x***	***y***	***z***	**U(eq)**
$C^{(100)}$	4409(3)	3185(2)	4924(2)	25.5(7)
$C^{(97)}$	4683(3)	8368(2)	9063(2)	26.0(7)
$C^{(85)}$	6217(3)	7631(2)	9225.8(19)	23.8(7)
$C^{(96)}$	2462(3)	7381(3)	5731(2)	35.6(9)
$C^{(44)}$	7642(3)	13846(2)	8462(2)	32.1(9)
$C^{(99)}$	7352(3)	5159(2)	6110(2)	31.3(8)
$C^{(102)}$	3206(3)	5084(3)	9670(2)	34.0(9)
$C^{(98)}$	7737(3)	6908(3)	9413(3)	44.3(11)
$C^{(95)}$	610(3)	5315(2)	6469(3)	43.4(11)
$B^{(1)}$	4450(3)	5480(2)	7357(2)	16.2(7)
$F^{(12)}$	-32(5)	5117(5)	5614(5)	65(3)
$F^{(27)}$	153(4)	5794(3)	6813(6)	51(2)
$F^{(15)}$	5169.5(18)	8859.9(13)	9782.0(13)	36.4(5)
$F^{(51)}$	3650(6)	3356(4)	4361(5)	25(2)
$F^{(50)}$	4039(8)	2729(5)	5325(6)	42(3)
$F^{(52)}$	4949(8)	2758(6)	4598(6)	46(3)
$F^{(58)}$	579(6)	5561(5)	7337(5)	62(3)
$F^{(59)}$	-189(4)	5553(6)	6115(8)	70(4)
$F^{(56)}$	2302(11)	6946(8)	4821(8)	80(6)
$F^{(57)}$	545(4)	4534(3)	6357(5)	27.5(14)
$F^{(11)}$	689(4)	4623(3)	6663(4)	32.6(13)
$F^{(54)}$	8211(8)	6560(7)	8877(7)	85(4)
$F^{(17)}$	8289(5)	7486(4)	9375(4)	70.2(17)
$F^{(55)}$	8234(6)	7663(5)	9698(6)	45(2)
$F^{(16)}$	7884(3)	6899(3)	10218(3)	58.7(16)

Table 2 Fractional Atomic Coordinates (×10^4) and Equivalent Isotropic Displacement Parameters (Å^2×10^3) for exp_1880_auto. U_{eq} is defined as 1/3 of of the trace of the orthogonalised U_{IJ} tensor.

**Atom**	***x***	***y***	***z***	**U(eq)**
F(53)	8072(13)	6581(12)	9859(12)	68(7)

Table 3 Anisotropic Displacement Parameters (Å^2×10^3) for exp_1880_auto. The Anisotropic displacement factor exponent takes the form: $-2\pi^2[h^2a^{*2}U_{11}+2hka^*b^*U_{12}+\ldots]$.

**Atom**	**U_{11}**	**U_{22}**	**U_{33}**	**U_{23}**	**U_{13}**	**U_{12}**
Ir(1)	9.78(7)	12.72(6)	10.31(6)	3.83(4)	3.72(4)	0.66(4)
P(1)	11.7(4)	14.3(3)	12.7(3)	5.1(3)	5.1(3)	1.4(3)
P(2)	12.0(4)	14.1(3)	11.2(3)	4.4(3)	4.0(3)	0.9(3)
O(1)	10.8(10)	17.0(10)	12.7(9)	4.4(8)	5.3(8)	2.1(8)
N(1)	9.5(12)	14.8(12)	14.5(11)	4.7(9)	5.6(9)	-0.5(9)
F(14)	28.8(12)	32.5(12)	58.2(15)	-6.5(10)	23.6(11)	1.8(9)
F(13)	50.4(15)	31.4(11)	38.8(12)	11.4(9)	15.9(11)	16.8(10)
F(21)	70.4(19)	66.4(17)	30.0(12)	22.7(12)	29.4(13)	22.2(14)
F(19)	94(2)	42.3(14)	56.4(16)	11.9(12)	56.5(17)	20.4(14)
F(20)	34.2(15)	104(2)	50.0(16)	7.9(15)	26.0(13)	6.9(15)
C(1)	9.5(14)	18.2(14)	15.8(13)	5.3(11)	6.2(11)	0.7(11)
F(91)	51.4(17)	25.0(12)	74.5(19)	2.0(12)	5.5(14)	-3.2(11)
F(30)	51.3(17)	78(2)	48.8(15)	5.3(14)	36.4(14)	-18.2(14)
F(22)	124(3)	34.9(14)	38.7(14)	15.7(11)	-3.5(16)	30.1(16)
C(39)	19.3(16)	13.7(13)	12.2(13)	3.4(10)	6.2(11)	0.8(11)
C(10)	4.1(13)	19.8(14)	13.9(13)	6.4(11)	2.9(10)	3.5(10)
C(11)	11.9(15)	17.3(14)	13.9(13)	3.9(11)	4.4(11)	1.1(11)
F(29)	77(3)	73(2)	128(4)	78(2)	74(3)	43(2)
F(24)	87(2)	43.1(16)	184(4)	67(2)	107(3)	42.6(16)
C(57)	14.1(16)	30.6(17)	19.2(15)	11.0(13)	6.8(12)	8.6(13)
C(24)	13.7(15)	13.1(13)	18.3(14)	3.1(11)	5.0(12)	0.4(11)

Table 3 Anisotropic Displacement Parameters (Å^2×10^3) for exp_1880_auto. The Anisotropic displacement factor exponent takes the form: $-2\pi^2[h^2a^{*2}U_{11}+2hka^*b^*U_{12}+\ldots]$.

**Atom**	**U_{11}**	**U_{22}**	**U_{33}**	**U_{23}**	**U_{13}**	**U_{12}**
C(49)	16.5(16)	15.9(14)	14.8(13)	3.8(11)	5.7(12)	-0.9(11)
C(40)	15.5(15)	16.5(14)	19.2(14)	8.7(11)	10.2(12)	3.1(11)
C(48)	12.6(15)	15.8(14)	18.2(14)	6.8(11)	8.1(12)	2.4(11)
C(38)	19.7(16)	14.8(13)	14.9(13)	3.6(11)	7.7(12)	0.5(11)
C(25)	20.4(17)	21.5(15)	16.6(14)	4.2(12)	6.0(12)	1.3(12)
F(32)	55(2)	69(2)	107(3)	67(2)	-13.3(18)	2.8(16)
C(30)	16.0(16)	17.2(14)	12.2(13)	3.1(11)	3.7(11)	-0.2(11)
C(18)	15.1(16)	24.6(15)	9.9(12)	4.5(11)	4.0(11)	2.3(12)
C(55)	7.1(14)	19.0(14)	15.4(13)	3.6(11)	2.5(11)	3.1(11)
C(23)	15.5(16)	23.4(15)	11.6(13)	4.7(11)	2.8(11)	1.0(12)
C(50)	17.6(16)	20.6(15)	22.8(15)	8.9(12)	9.8(13)	3.1(12)
C(46)	23.7(18)	21.5(15)	25.3(16)	10.9(13)	17.9(14)	8.2(13)
C(9)	13.3(15)	15.3(14)	20.6(15)	2.6(11)	8.8(12)	-0.4(11)
C(2)	14.1(15)	25.4(15)	12.7(13)	3.7(11)	5.4(11)	-0.2(12)
F(33)	52.8(18)	62.9(18)	93(2)	-23.6(16)	48.5(17)	-33.6(15)
C(31)	18.3(16)	19.4(14)	15.8(14)	7.2(11)	5.0(12)	4.0(12)
C(19)	20.0(17)	20.6(15)	13.5(13)	3.8(11)	5.5(12)	1.0(12)
C(4)	13.7(16)	20.3(15)	21.3(15)	0.8(12)	7.5(12)	-2.1(12)
F(31)	44(2)	17.0(15)	62(2)	3.2(15)	-8.1(17)	-2.8(13)
C(41)	19.3(16)	17.6(14)	20.4(15)	6.9(12)	12.6(13)	2.9(12)
C(56)	11.6(15)	22.8(15)	18.3(14)	5.5(12)	6.8(12)	4.7(12)
C(29)	16.4(16)	18.9(15)	18.1(14)	5.4(11)	5.8(12)	-2.1(12)
C(47)	19.1(16)	23.0(15)	16.6(14)	8.9(12)	9.6(12)	5.6(12)
C(32)	21.7(17)	21.5(15)	16.6(14)	8.0(12)	6.0(12)	1.0(12)
C(20)	17.1(17)	29.3(17)	14.8(14)	3.6(12)	3.8(12)	-3.1(13)
C(92)	20.9(18)	21.7(16)	26.5(16)	5.1(13)	2.7(14)	5.1(13)

Table 3 Anisotropic Displacement Parameters (Å^2×10^3) for exp_1880_auto. The Anisotropic displacement factor exponent takes the form: -2π^2[h^{2}a*^{2}U$_{11}$+2hka*b*U$_{12}$+…].

Atom	U_{11}	U_{22}	U_{33}	U_{23}	U_{13}	U_{12}
C(3)	16.7(16)	25.7(16)	18.3(15)	-0.9(12)	5.7(12)	-4.2(12)
C(93)	24.5(18)	21.9(15)	18.1(14)	6.2(12)	6.9(13)	6.6(13)
C(8)	19.7(17)	18.7(15)	21.4(15)	5.7(12)	9.5(13)	-1.7(12)
C(54)	17.6(16)	23.7(16)	24.9(16)	10.9(13)	10.6(13)	3.9(12)
C(33)	20.3(17)	20.1(15)	12.5(13)	2.9(11)	5.0(12)	0.3(12)
C(60)	14.4(16)	21.8(15)	21.4(15)	5.4(12)	6.6(12)	1.3(12)
C(94)	20.1(17)	21.6(15)	16.5(14)	6.0(12)	8.4(12)	2.1(12)
C(42)	27.6(19)	21.6(16)	22.8(16)	5.6(13)	12.8(14)	5.9(13)
C(01Q)	24.4(18)	23.8(16)	26.0(16)	14.6(13)	14.0(14)	11.4(13)
F(28)	101(3)	23.0(16)	59(2)	19.1(14)	30(2)	4.5(16)
F(26)	23.0(15)	130(3)	150(4)	103(3)	28.6(18)	16.5(17)
C(37)	23.9(18)	21.8(15)	19.0(14)	7.5(12)	10.2(13)	5.2(13)
C(76)	14.8(16)	19.5(15)	20.1(15)	4.6(12)	4.9(12)	0.4(12)
C(87)	17.5(16)	23.8(16)	17.6(14)	5.8(12)	6.4(12)	2.7(12)
C(71)	15.4(16)	20.2(15)	20.3(15)	5.9(12)	5.2(12)	2.5(12)
C(5)	31(2)	19.4(16)	27.7(17)	-2.3(13)	13.9(15)	-6.6(13)
C(21)	13.9(16)	33.6(18)	18.6(15)	7.8(13)	3.6(12)	4.8(13)
C(34)	30.6(19)	23.3(16)	17.4(14)	8.4(12)	10.6(13)	2.1(13)
C(51)	24.9(18)	22.5(16)	27.1(16)	12.7(13)	11.2(14)	3.2(13)
C(59)	16.9(17)	25.1(16)	25.2(16)	0.9(13)	4.1(13)	-1.0(13)
C(74)	20.9(17)	22.8(16)	23.5(15)	12.1(12)	10.3(13)	3.4(13)
C(70)	16.1(16)	16.0(14)	17.9(14)	7.9(11)	5.4(12)	3.6(11)
C(88)	19.6(16)	14.5(13)	14.3(13)	1.2(11)	6.3(12)	2.9(11)
C(58)	18.1(17)	32.1(18)	16.3(14)	3.8(13)	0.3(13)	6.4(13)
C(89)	20.4(17)	13.4(14)	25.6(16)	4.1(12)	6.0(13)	1.1(12)
C(80)	21.3(17)	21.9(15)	21.3(15)	6.7(12)	8.4(13)	3.8(13)

Table 3 Anisotropic Displacement Parameters (Å^2×10^3) for exp_1880_auto. The Anisotropic displacement factor exponent takes the form: $-2\pi^2[h^2a^{*2}U_{11}+2hka^*b^*U_{12}+\ldots]$.

Atom	U_{11}	U_{22}	U_{33}	U_{23}	U_{13}	U_{12}
C(75)	19.0(16)	16.8(14)	20.5(15)	8.1(11)	5.8(12)	1.3(12)
C(22)	19.0(17)	26.6(16)	17.3(14)	7.0(12)	3.6(13)	5.6(13)
C(28)	30(2)	22.8(16)	25.8(17)	11.7(13)	5.5(15)	1.5(14)
C(27)	32(2)	15.0(15)	33.6(18)	6.7(13)	9.5(15)	-0.1(13)
C(73)	24.2(18)	24.5(16)	20.5(15)	9.0(13)	10.8(13)	8.2(13)
C(45)	38(2)	20.7(16)	33.8(18)	11.2(14)	22.6(16)	10.9(14)
C(35)	30.1(19)	26.1(17)	21.8(15)	6.9(13)	16.5(14)	-0.7(14)
C(52)	28.1(19)	24.2(16)	26.4(16)	10.1(13)	14.0(14)	-1.9(14)
C(90)	17.4(17)	18.3(15)	35.9(18)	4.0(13)	4.0(14)	-0.1(12)
C(81)	14.4(15)	20.6(15)	18.2(14)	5.8(12)	3.7(12)	1.4(12)
C(53)	18.7(18)	30.6(18)	34.5(18)	13.1(14)	15.7(15)	3.3(14)
C(82)	18.1(16)	19.4(14)	14.8(13)	6.3(11)	6.9(12)	0.4(12)
C(43)	38(2)	25.6(17)	23.2(16)	1.0(13)	16.8(15)	5.6(15)
C(77)	18.0(17)	20.5(15)	24.8(16)	7.5(12)	2.2(13)	-0.2(12)
C(26)	28.1(19)	18.3(15)	24.6(16)	0.1(12)	8.7(14)	-0.5(13)
C(36)	21.2(18)	26.7(17)	26.6(16)	8.2(13)	14.3(14)	5.2(13)
C(86)	22.2(17)	21.3(15)	18.4(14)	3.7(12)	9.8(13)	1.5(13)
C(72)	21.4(17)	20.9(15)	18.5(14)	7.2(12)	4.5(13)	5.5(13)
C(78)	24.9(19)	27.0(17)	25.9(16)	13.3(13)	7.6(14)	-0.2(14)
C(7)	34(2)	22.3(16)	28.7(17)	6.9(13)	14.5(15)	2.2(14)
C(101)	32(2)	23.1(17)	32.6(18)	11.8(14)	11.7(16)	1.1(14)
C(79)	24.6(19)	29.6(17)	21.7(16)	7.9(13)	10.6(14)	3.3(14)
F(23)	123(4)	40(2)	22.2(16)	-2.0(14)	20(2)	-25(2)
C(83)	21.8(18)	25.0(16)	19.2(15)	6.6(12)	5.4(13)	5.7(13)
C(84)	24.1(19)	34.4(19)	19.3(15)	4.6(14)	2.6(14)	2.9(15)
C(6)	36(2)	17.4(16)	37(2)	3.3(14)	19.7(17)	-4.1(14)

Table 3 Anisotropic Displacement Parameters (Å^2×10^3) for exp_1880_auto. The Anisotropic displacement factor exponent takes the form: $-2\pi^2[h^2a^{*2}U_{11}+2hka^*b^*U_{12}+\ldots]$.

Atom	U_{11}	U_{22}	U_{33}	U_{23}	U_{13}	U_{12}
$F^{(25)}$	19(2)	84(3)	111(4)	-72(3)	28(2)	-21(2)
$C^{(100)}$	26.1(19)	26.3(17)	22.6(16)	3.5(13)	6.9(14)	4.9(14)
$C^{(97)}$	27.7(19)	23.5(17)	24.5(16)	-0.8(13)	9.2(14)	-0.7(14)
$C^{(85)}$	22.9(18)	27.8(17)	17.2(15)	0.8(12)	4.2(13)	-3.3(13)
$C^{(96)}$	31(2)	50(2)	41(2)	31.0(19)	19.8(18)	21.1(18)
$C^{(44)}$	51(3)	18.2(16)	35.7(19)	5.4(14)	26.5(18)	11.0(16)
$C^{(99)}$	28(2)	34.8(19)	38(2)	14.1(16)	16.7(16)	1.2(15)
$C^{(102)}$	38(2)	44(2)	29.8(19)	13.9(16)	20.3(17)	8.2(18)
$C^{(98)}$	30(2)	46(2)	40(2)	-1.9(19)	-6.3(18)	7.1(19)
$C^{(95)}$	19(2)	24.2(18)	89(3)	21(2)	13(2)	2.9(15)
$B^{(1)}$	17.4(18)	16.3(16)	16.6(15)	4.8(12)	6.9(13)	2.7(13)
$F^{(12)}$	25(3)	84(5)	70(4)	24(4)	-13(3)	-25(3)
$F^{(27)}$	29(4)	40(3)	98(7)	19(3)	39(4)	6(2)
$F^{(15)}$	39.6(13)	28.0(11)	30.5(11)	-10.8(9)	7.3(10)	0.1(9)
$F^{(58)}$	57(5)	74(5)	60(5)	-9(4)	43(4)	-23(4)
$F^{(59)}$	17(3)	76(7)	134(11)	75(8)	14(4)	4(3)

Table 4 Bond Lengths for exp_1880_auto.

Atom	Atom	Length/Å	Atom	Atom	Length/Å
$Ir^{(1)}$	$P^{(1)}$	2.3327(8)	$C^{(93)}$	$C^{(96)}$	1.502(5)
$Ir^{(1)}$	$P^{(2)}$	2.2669(7)	$C^{(8)}$	$C^{(7)}$	1.370(5)
$Ir^{(1)}$	$O^{(1)}$	2.175(2)	$C^{(54)}$	$C^{(53)}$	1.387(5)
$Ir^{(1)}$	$N^{(1)}$	2.138(2)	$C^{(33)}$	$C^{(34)}$	1.414(5)
$Ir^{(1)}$	$C^{(11)1}$	2.221(3)	$C^{(60)}$	$C^{(59)}$	1.390(4)
$P^{(1)}$	$C^{(49)}$	1.824(3)	$C^{(94)}$	$C^{(88)}$	1.406(4)
$P^{(1)}$	$C^{(48)}$	1.846(3)	$C^{(42)}$	$C^{(43)}$	1.373(5)

Table 4 Bond Lengths for exp_1880_auto.

Atom	Atom	Length/Å	Atom	Atom	Length/Å
P(1)	C(55)	1.841(3)	F(28)	C(96)	1.379(5)
P(2)	C(24)	1.814(3)	F(26)	C(99)	1.332(5)
P(2)	C(30)	1.838(3)	C(37)	C(36)	1.358(5)
P(2)	C(18)	1.841(3)	C(76)	C(81)	1.400(4)
O(1)	C(10)	1.259(3)	C(76)	C(77)	1.398(5)
N(1)	C(1)	1.337(4)	C(87)	C(82)	1.395(4)
N(1)	C(9)	1.380(4)	C(87)	C(86)	1.396(4)
F(14)	C(97)	1.336(4)	C(71)	C(70)	1.408(4)
F(13)	C(97)	1.343(4)	C(71)	C(72)	1.389(5)
F(21)	C(102)	1.334(4)	C(5)	C(6)	1.354(5)
F(19)	C(102)	1.336(5)	C(21)	C(22)	1.388(5)
F(20)	C(102)	1.333(5)	C(34)	C(35)	1.363(5)
C(1)	C(10)	1.499(4)	C(51)	C(52)	1.375(5)
C(1)	C(2)	1.407(4)	C(59)	C(58)	1.388(5)
F(91)	C(101)	1.334(4)	C(74)	C(75)	1.395(5)
F(30)	C(99)	1.320(5)	C(74)	C(73)	1.384(5)
F(22)	C(101)	1.322(4)	C(74)	C(99)	1.493(5)
C(39)	C(40)	1.501(4)	C(70)	C(75)	1.394(5)
C(39)	C(38)	1.437(4)	C(70)	B(1)	1.640(5)
C(39)	C(30)	1.386(4)	C(88)	C(89)	1.392(5)
C(10)	C(11)	1.431(4)	C(88)	B(1)	1.647(5)
F(29)	C(96)	1.294(5)	C(89)	C(90)	1.396(5)
F(24)	C(101)	1.304(5)	C(80)	C(81)	1.400(5)
C(57)	C(56)	1.395(4)	C(80)	C(79)	1.384(5)
C(57)	C(58)	1.381(5)	C(28)	C(27)	1.382(5)
C(24)	C(25)	1.399(4)	C(27)	C(26)	1.376(5)

Table 4 Bond Lengths for exp_1880_auto.

Atom	Atom	Length/Å	Atom	Atom	Length/Å
C(24)	C(29)	1.392(4)	C(73)	C(72)	1.387(5)
C(49)	C(50)	1.398(4)	C(45)	C(44)	1.362(5)
C(49)	C(54)	1.390(5)	C(35)	C(36)	1.412(5)
C(40)	C(48)	1.386(4)	C(52)	C(53)	1.385(5)
C(40)	C(41)	1.431(4)	C(90)	C(95)	1.500(5)
C(48)	C(47)	1.421(4)	C(81)	B(1)	1.645(5)
C(38)	C(33)	1.427(4)	C(82)	C(83)	1.403(5)
C(38)	C(37)	1.414(5)	C(82)	B(1)	1.645(4)
C(25)	C(26)	1.391(5)	C(43)	C(44)	1.406(5)
F(32)	C(96)	1.298(5)	C(77)	C(78)	1.382(5)
C(30)	C(31)	1.425(4)	C(77)	C(101)	1.499(5)
C(18)	C(23)	1.398(4)	C(86)	C(97)	1.490(4)
C(18)	C(19)	1.396(4)	C(86)	C(85)	1.384(5)
C(55)	C(56)	1.404(4)	C(72)	C(100)	1.495(5)
C(55)	C(60)	1.392(4)	C(78)	C(79)	1.388(5)
C(23)	C(22)	1.393(4)	C(7)	C(6)	1.410(5)
C(50)	C(51)	1.386(5)	C(79)	C(102)	1.493(5)
C(46)	C(41)	1.424(4)	F(23)	C(100)	1.362(5)
C(46)	C(01Q)	1.407(5)	C(83)	C(84)	1.387(5)
C(46)	C(45)	1.418(4)	C(84)	C(85)	1.388(5)
C(9)	C(4)	1.422(4)	C(84)	C(98)	1.492(5)
C(9)	C(8)	1.411(4)	F(25)	C(100)	1.278(5)
C(2)	C(3)	1.360(5)	C(100)	F(51)	1.319(8)
F(33)	C(99)	1.318(5)	C(100)	F(50)	1.358(10)
C(31)	C(32)	1.364(5)	C(100)	F(52)	1.241(10)
C(19)	C(20)	1.396(5)	C(97)	F(15)	1.342(4)

Table 4 Bond Lengths for exp_1880_auto.

Atom	Atom	Length/Å	Atom	Atom	Length/Å
C(4)	C(3)	1.404(5)	C(96)	F(56)	1.569(14)
C(4)	C(5)	1.421(5)	C(98)	F(54)	1.359(12)
F(31)	C(100)	1.352(5)	C(98)	F(17)	1.277(8)
C(41)	C(42)	1.421(4)	C(98)	F(55)	1.374(10)
C(29)	C(28)	1.401(4)	C(98)	F(16)	1.386(7)
C(47)	C(01Q)	1.367(4)	C(98)	F(53)	1.069(16)
C(32)	C(33)	1.412(5)	C(95)	F(12)	1.482(8)
C(20)	C(21)	1.386(5)	C(95)	F(27)	1.219(7)
C(92)	C(93)	1.384(5)	C(95)	F(58)	1.518(9)
C(92)	C(90)	1.389(5)	C(95)	F(59)	1.243(7)
F(18)	C(98)	1.360(6)	C(95)	F(57)	1.298(6)
C(93)	C(94)	1.393(4)	C(95)	F(11)	1.296(6)

^{1}2-X,2-Y,1-Z

Table 5 Bond Angles for exp_1880_auto.

Atom	Atom	Atom	Angle/˚	Atom	Atom	Atom	Angle/˚
P(2)	Ir(1)	P(1)	90.32(3)	C(88)	C(89)	C(90)	122.3(3)
O(1)	Ir(1)	P(1)	92.91(6)	C(79)	C(80)	C(81)	122.8(3)
O(1)	Ir(1)	P(2)	108.53(6)	C(70)	C(75)	C(74)	122.4(3)
O(1)	Ir(1)	C(11)^{1}	88.30(9)	C(21)	C(22)	C(23)	119.6(3)
N(1)	Ir(1)	P(1)	91.09(7)	C(27)	C(28)	C(29)	120.0(3)
N(1)	Ir(1)	P(2)	175.50(7)	C(26)	C(27)	C(28)	120.7(3)
N(1)	Ir(1)	O(1)	75.66(8)	C(74)	C(73)	C(72)	118.2(3)
N(1)	Ir(1)	C(11)^{1}	82.28(10)	C(44)	C(45)	C(46)	120.8(3)
C(11)^{1}	Ir(1)	P(1)	172.75(8)	C(34)	C(35)	C(36)	119.9(3)
C(11)^{1}	Ir(1)	P(2)	96.08(8)	C(51)	C(52)	C(53)	120.1(3)
C(49)	P(1)	Ir(1)	117.51(10)	C(92)	C(90)	C(89)	120.8(3)

Table 5 Bond Angles for exp_1880_auto.

Atom	Atom	Atom	Angle/˚	Atom	Atom	Atom	Angle/˚
$C^{(49)}$	$P^{(1)}$	$C^{(48)}$	104.19(14)	$C^{(92)}$	$C^{(90)}$	$C^{(95)}$	119.4(3)
$C^{(49)}$	$P^{(1)}$	$C^{(55)}$	103.44(14)	$C^{(89)}$	$C^{(90)}$	$C^{(95)}$	119.7(3)
$C^{(48)}$	$P^{(1)}$	$Ir^{(1)}$	116.05(10)	$C^{(76)}$	$C^{(81)}$	$C^{(80)}$	115.8(3)
$C^{(55)}$	$P^{(1)}$	$Ir^{(1)}$	108.93(10)	$C^{(76)}$	$C^{(81)}$	$B^{(1)}$	126.1(3)
$C^{(55)}$	$P^{(1)}$	$C^{(48)}$	105.39(13)	$C^{(80)}$	$C^{(81)}$	$B^{(1)}$	118.0(3)
$C^{(24)}$	$P^{(2)}$	$Ir^{(1)}$	119.05(10)	$C^{(52)}$	$C^{(53)}$	$C^{(54)}$	120.0(3)
$C^{(24)}$	$P^{(2)}$	$C^{(30)}$	105.00(14)	$C^{(87)}$	$C^{(82)}$	$C^{(83)}$	115.7(3)
$C^{(24)}$	$P^{(2)}$	$C^{(18)}$	104.38(14)	$C^{(87)}$	$C^{(82)}$	$B^{(1)}$	121.8(3)
$C^{(30)}$	$P^{(2)}$	$Ir^{(1)}$	111.25(10)	$C^{(83)}$	$C^{(82)}$	$B^{(1)}$	122.1(3)
$C^{(30)}$	$P^{(2)}$	$C^{(18)}$	103.23(14)	$C^{(42)}$	$C^{(43)}$	$C^{(44)}$	120.7(3)
$C^{(18)}$	$P^{(2)}$	$Ir^{(1)}$	112.47(10)	$C^{(76)}$	$C^{(77)}$	$C^{(101)}$	120.2(3)
$C^{(10)}$	$O^{(1)}$	$Ir^{(1)}$	116.29(18)	$C^{(78)}$	$C^{(77)}$	$C^{(76)}$	121.3(3)
$C^{(1)}$	$N^{(1)}$	$Ir^{(1)}$	115.15(19)	$C^{(78)}$	$C^{(77)}$	$C^{(101)}$	118.5(3)
$C^{(1)}$	$N^{(1)}$	$C^{(9)}$	118.4(2)	$C^{(27)}$	$C^{(26)}$	$C^{(25)}$	120.0(3)
$C^{(9)}$	$N^{(1)}$	$Ir^{(1)}$	126.4(2)	$C^{(37)}$	$C^{(36)}$	$C^{(35)}$	120.7(3)
$N^{(1)}$	$C^{(1)}$	$C^{(10)}$	115.3(2)	$C^{(87)}$	$C^{(86)}$	$C^{(97)}$	118.1(3)
$N^{(1)}$	$C^{(1)}$	$C^{(2)}$	123.0(3)	$C^{(85)}$	$C^{(86)}$	$C^{(87)}$	121.0(3)
$C^{(2)}$	$C^{(1)}$	$C^{(10)}$	121.7(3)	$C^{(85)}$	$C^{(86)}$	$C^{(97)}$	120.8(3)
$C^{(38)}$	$C^{(39)}$	$C^{(40)}$	116.9(3)	$C^{(71)}$	$C^{(72)}$	$C^{(100)}$	120.8(3)
$C^{(30)}$	$C^{(39)}$	$C^{(40)}$	122.8(3)	$C^{(73)}$	$C^{(72)}$	$C^{(71)}$	120.8(3)
$C^{(30)}$	$C^{(39)}$	$C^{(38)}$	120.4(3)	$C^{(73)}$	$C^{(72)}$	$C^{(100)}$	118.4(3)
$O^{(1)}$	$C^{(10)}$	$C^{(1)}$	116.6(3)	$C^{(77)}$	$C^{(78)}$	$C^{(79)}$	118.0(3)
$O^{(1)}$	$C^{(10)}$	$C^{(11)}$	122.1(3)	$C^{(8)}$	$C^{(7)}$	$C^{(6)}$	121.1(3)
$C^{(11)}$	$C^{(10)}$	$C^{(1)}$	120.9(3)	$F^{(91)}$	$C^{(101)}$	$C^{(77)}$	111.6(3)
$C^{(10)}$	$C^{(11)}$	$Ir^{(1)1}$	105.07(19)	$F^{(22)}$	$C^{(101)}$	$F^{(91)}$	103.4(3)
$C^{(58)}$	$C^{(57)}$	$C^{(56)}$	120.0(3)	$F^{(22)}$	$C^{(101)}$	$C^{(77)}$	113.4(3)

Table 5 Bond Angles for exp_1880_auto.

Atom	Atom	Atom	Angle/˚	Atom	Atom	Atom	Angle/˚
C(25)	C(24)	P(2)	120.7(2)	F(24)	C(101)	F(91)	104.2(3)
C(29)	C(24)	P(2)	119.9(2)	F(24)	C(101)	F(22)	109.1(4)
C(29)	C(24)	C(25)	119.4(3)	F(24)	C(101)	C(77)	114.2(3)
C(50)	C(49)	P(1)	121.8(2)	C(80)	C(79)	C(78)	120.6(3)
C(54)	C(49)	P(1)	119.3(2)	C(80)	C(79)	C(102)	119.5(3)
C(54)	C(49)	C(50)	118.9(3)	C(78)	C(79)	C(102)	119.9(3)
C(48)	C(40)	C(39)	121.2(3)	C(84)	C(83)	C(82)	122.1(3)
C(48)	C(40)	C(41)	120.8(3)	C(83)	C(84)	C(85)	121.3(3)
C(41)	C(40)	C(39)	117.8(3)	C(83)	C(84)	C(98)	120.6(3)
C(40)	C(48)	P(1)	119.6(2)	C(85)	C(84)	C(98)	118.0(3)
C(40)	C(48)	C(47)	118.7(3)	C(5)	C(6)	C(7)	119.9(3)
C(47)	C(48)	P(1)	121.5(2)	F(31)	C(100)	C(72)	112.3(3)
C(33)	C(38)	C(39)	119.1(3)	F(31)	C(100)	F(23)	98.9(3)
C(37)	C(38)	C(39)	122.5(3)	F(23)	C(100)	C(72)	112.1(3)
C(37)	C(38)	C(33)	118.3(3)	F(25)	C(100)	F(31)	105.7(4)
C(26)	C(25)	C(24)	120.2(3)	F(25)	C(100)	C(72)	116.8(3)
C(39)	C(30)	P(2)	120.8(2)	F(25)	C(100)	F(23)	109.6(4)
C(39)	C(30)	C(31)	119.2(3)	F(51)	C(100)	C(72)	111.0(4)
C(31)	C(30)	P(2)	119.7(2)	F(51)	C(100)	F(50)	107.1(7)
C(23)	C(18)	P(2)	118.8(2)	F(50)	C(100)	C(72)	107.4(5)
C(19)	C(18)	P(2)	122.2(2)	F(52)	C(100)	C(72)	114.7(6)
C(19)	C(18)	C(23)	118.6(3)	F(52)	C(100)	F(51)	109.7(7)
C(56)	C(55)	P(1)	120.8(2)	F(52)	C(100)	F(50)	106.6(7)
C(60)	C(55)	P(1)	119.8(2)	F(14)	C(97)	F(13)	105.7(3)
C(60)	C(55)	C(56)	118.8(3)	F(14)	C(97)	C(86)	112.7(3)
C(22)	C(23)	C(18)	121.1(3)	F(14)	C(97)	F(15)	106.4(3)

Table 5 Bond Angles for exp_1880_auto.

Atom	Atom	Atom	Angle/˚	Atom	Atom	Atom	Angle/˚
$C^{(51)}$	$C^{(50)}$	$C^{(49)}$	120.3(3)	$F^{(13)}$	$C^{(97)}$	$C^{(86)}$	112.2(3)
$C^{(01Q)}$	$C^{(46)}$	$C^{(41)}$	118.5(3)	$F^{(15)}$	$C^{(97)}$	$F^{(13)}$	106.2(3)
$C^{(01Q)}$	$C^{(46)}$	$C^{(45)}$	122.1(3)	$F^{(15)}$	$C^{(97)}$	$C^{(86)}$	113.0(3)
$C^{(45)}$	$C^{(46)}$	$C^{(41)}$	119.5(3)	$C^{(86)}$	$C^{(85)}$	$C^{(84)}$	117.6(3)
$N^{(1)}$	$C^{(9)}$	$C^{(4)}$	120.5(3)	$F^{(29)}$	$C^{(96)}$	$F^{(32)}$	115.6(4)
$N^{(1)}$	$C^{(9)}$	$C^{(8)}$	120.6(3)	$F^{(29)}$	$C^{(96)}$	$C^{(93)}$	115.2(3)
$C^{(8)}$	$C^{(9)}$	$C^{(4)}$	118.8(3)	$F^{(29)}$	$C^{(96)}$	$F^{(28)}$	99.4(4)
$C^{(3)}$	$C^{(2)}$	$C^{(1)}$	119.4(3)	$F^{(32)}$	$C^{(96)}$	$C^{(93)}$	114.8(4)
$C^{(32)}$	$C^{(31)}$	$C^{(30)}$	121.3(3)	$F^{(32)}$	$C^{(96)}$	$F^{(28)}$	98.2(3)
$C^{(18)}$	$C^{(19)}$	$C^{(20)}$	120.3(3)	$C^{(93)}$	$C^{(96)}$	$F^{(56)}$	99.1(6)
$C^{(3)}$	$C^{(4)}$	$C^{(9)}$	119.0(3)	$F^{(28)}$	$C^{(96)}$	$C^{(93)}$	110.9(3)
$C^{(3)}$	$C^{(4)}$	$C^{(5)}$	122.1(3)	$C^{(45)}$	$C^{(44)}$	$C^{(43)}$	120.2(3)
$C^{(5)}$	$C^{(4)}$	$C^{(9)}$	119.0(3)	$F^{(30)}$	$C^{(99)}$	$F^{(26)}$	104.9(4)
$C^{(46)}$	$C^{(41)}$	$C^{(40)}$	119.2(3)	$F^{(30)}$	$C^{(99)}$	$C^{(74)}$	113.1(3)
$C^{(42)}$	$C^{(41)}$	$C^{(40)}$	122.7(3)	$F^{(33)}$	$C^{(99)}$	$F^{(30)}$	107.2(3)
$C^{(42)}$	$C^{(41)}$	$C^{(46)}$	118.1(3)	$F^{(33)}$	$C^{(99)}$	$F^{(26)}$	105.3(4)
$C^{(57)}$	$C^{(56)}$	$C^{(55)}$	120.4(3)	$F^{(33)}$	$C^{(99)}$	$C^{(74)}$	114.2(3)
$C^{(24)}$	$C^{(29)}$	$C^{(28)}$	119.8(3)	$F^{(26)}$	$C^{(99)}$	$C^{(74)}$	111.5(3)
$C^{(01Q)}$	$C^{(47)}$	$C^{(48)}$	121.1(3)	$F^{(21)}$	$C^{(102)}$	$F^{(19)}$	105.8(3)
$C^{(31)}$	$C^{(32)}$	$C^{(33)}$	121.1(3)	$F^{(21)}$	$C^{(102)}$	$C^{(79)}$	112.7(3)
$C^{(21)}$	$C^{(20)}$	$C^{(19)}$	120.4(3)	$F^{(19)}$	$C^{(102)}$	$C^{(79)}$	113.0(3)
$C^{(93)}$	$C^{(92)}$	$C^{(90)}$	118.0(3)	$F^{(20)}$	$C^{(102)}$	$F^{(21)}$	106.3(3)
$C^{(2)}$	$C^{(3)}$	$C^{(4)}$	119.4(3)	$F^{(20)}$	$C^{(102)}$	$F^{(19)}$	106.1(3)
$C^{(92)}$	$C^{(93)}$	$C^{(94)}$	120.9(3)	$F^{(20)}$	$C^{(102)}$	$C^{(79)}$	112.2(3)
$C^{(92)}$	$C^{(93)}$	$C^{(96)}$	119.5(3)	$F^{(18)}$	$C^{(98)}$	$C^{(84)}$	113.5(4)
$C^{(94)}$	$C^{(93)}$	$C^{(96)}$	119.4(3)	$F^{(18)}$	$C^{(98)}$	$F^{(16)}$	97.6(4)

Table 5 Bond Angles for exp_1880_auto.

Atom	Atom	Atom	Angle/˚	Atom	Atom	Atom	Angle/˚
C(7)	C(8)	C(9)	120.3(3)	F(54)	C(98)	C(84)	111.4(6)
C(53)	C(54)	C(49)	120.4(3)	F(54)	C(98)	F(55)	100.6(7)
C(32)	C(33)	C(38)	118.9(3)	F(17)	C(98)	F(18)	111.0(5)
C(32)	C(33)	C(34)	122.0(3)	F(17)	C(98)	C(84)	114.4(5)
C(34)	C(33)	C(38)	119.1(3)	F(17)	C(98)	F(16)	109.4(5)
C(59)	C(60)	C(55)	120.5(3)	F(55)	C(98)	C(84)	112.5(5)
C(93)	C(94)	C(88)	122.0(3)	F(16)	C(98)	C(84)	109.6(4)
C(43)	C(42)	C(41)	120.7(3)	F(53)	C(98)	C(84)	127.9(11)
C(47)	C(01Q)	C(46)	121.6(3)	F(53)	C(98)	F(54)	95.1(14)
C(36)	C(37)	C(38)	121.0(3)	F(53)	C(98)	F(55)	105.0(11)
C(77)	C(76)	C(81)	121.5(3)	C(90)	C(95)	F(58)	108.0(4)
C(82)	C(87)	C(86)	122.2(3)	F(12)	C(95)	C(90)	104.5(5)
C(72)	C(71)	C(70)	122.3(3)	F(27)	C(95)	C(90)	113.8(4)
C(6)	C(5)	C(4)	121.0(3)	F(27)	C(95)	F(12)	103.2(6)
C(20)	C(21)	C(22)	120.0(3)	F(27)	C(95)	F(11)	116.1(6)
C(35)	C(34)	C(33)	120.9(3)	F(59)	C(95)	C(90)	119.7(5)
C(52)	C(51)	C(50)	120.3(3)	F(59)	C(95)	F(58)	99.6(7)
C(58)	C(59)	C(60)	120.2(3)	F(59)	C(95)	F(57)	111.1(6)
C(75)	C(74)	C(99)	120.5(3)	F(57)	C(95)	C(90)	114.9(4)
C(73)	C(74)	C(75)	120.8(3)	F(57)	C(95)	F(58)	100.3(5)
C(73)	C(74)	C(99)	118.6(3)	F(11)	C(95)	C(90)	113.3(4)
C(71)	C(70)	B(1)	119.2(3)	F(11)	C(95)	F(12)	104.0(5)
C(75)	C(70)	C(71)	115.6(3)	C(70)	B(1)	C(88)	108.2(2)
C(75)	C(70)	B(1)	125.2(3)	C(70)	B(1)	C(81)	112.0(2)
C(94)	C(88)	B(1)	119.8(3)	C(70)	B(1)	C(82)	113.2(3)
C(89)	C(88)	C(94)	115.9(3)	C(81)	B(1)	C(88)	110.3(3)

Table 5 Bond Angles for exp_1880_auto.

Atom	Atom	Atom	Angle/˚	Atom	Atom	Atom	Angle/˚
C(89)	C(88)	B(1)	124.2(3)	C(81)	B(1)	C(82)	104.0(2)
C(57)	C(58)	C(59)	120.1(3)	C(82)	B(1)	C(88)	109.2(2)

[1]2-X,2-Y,1-Z

Table 6 Torsion Angles for exp_1880_auto.

A	B	C	D	Angle/˚	A	B	C	D	Angle/˚
Ir(1)	P(1)	C(49)	C(50)	-0.4(3)	C(8)	C(9)	C(4)	C(5)	1.1(5)
Ir(1)	P(1)	C(49)	C(54)	179.7(2)	C(8)	C(7)	C(6)	C(5)	-0.7(6)
Ir(1)	P(1)	C(48)	C(40)	-69.8(3)	C(54)	C(49)	C(50)	C(51)	0.6(5)
Ir(1)	P(1)	C(48)	C(47)	105.6(3)	C(33)	C(38)	C(37)	C(36)	1.9(5)
Ir(1)	P(1)	C(55)	C(56)	-66.6(2)	C(33)	C(34)	C(35)	C(36)	0.6(5)
Ir(1)	P(1)	C(55)	C(60)	104.5(2)	C(60)	C(55)	C(56)	C(57)	1.8(4)
Ir(1)	P(2)	C(24)	C(25)	173.8(2)	C(60)	C(59)	C(58)	C(57)	-1.0(5)
Ir(1)	P(2)	C(24)	C(29)	-5.7(3)	C(94)	C(93)	C(96)	F(29)	30.5(5)
Ir(1)	P(2)	C(30)	C(39)	-78.9(2)	C(94)	C(93)	C(96)	F(32)	168.5(4)
Ir(1)	P(2)	C(30)	C(31)	94.5(2)	C(94)	C(93)	C(96)	F(28)	-81.3(4)
Ir(1)	P(2)	C(18)	C(23)	-44.3(3)	C(94)	C(93)	C(96)	F(56)	97.9(6)
Ir(1)	P(2)	C(18)	C(19)	128.9(2)	C(94)	C(88)	C(89)	C(90)	-0.5(4)
Ir(1)	O(1)	C(10)	C(1)	8.0(3)	C(94)	C(88)	B(1)	C(70)	-69.1(3)
Ir(1)	O(1)	C(10)	C(11)	-165.3(2)	C(94)	C(88)	B(1)	C(81)	168.1(3)
Ir(1)	N(1)	C(1)	C(10)	9.7(3)	C(94)	C(88)	B(1)	C(82)	54.5(4)
Ir(1)	N(1)	C(1)	C(2)	-171.1(2)	C(42)	C(43)	C(44)	C(45)	0.5(6)
Ir(1)	N(1)	C(9)	C(4)	172.7(2)	C(01Q)	C(46)	C(41)	C(40)	3.6(5)
Ir(1)	N(1)	C(9)	C(8)	-9.3(4)	C(01Q)	C(46)	C(41)	C(42)	-177.3(3)
P(1)	C(49)	C(50)	C(51)	-179.3(2)	C(01Q)	C(46)	C(45)	C(44)	177.8(4)
P(1)	C(49)	C(54)	C(53)	179.7(3)	C(37)	C(38)	C(33)	C(32)	179.6(3)
P(1)	C(48)	C(47)	C(01Q)	-171.3(3)	C(37)	C(38)	C(33)	C(34)	-1.8(4)

Table 6 Torsion Angles for exp_1880_auto.

A	B	C	D	Angle/˚	A	B	C	D	Angle/˚
$P^{(1)}$	$C^{(55)}$	$C^{(56)}$	$C^{(57)}$	173.1(2)	$C^{(76)}$	$C^{(81)}$	$B^{(1)}$	$C^{(70)}$	3.5(4)
$P^{(1)}$	$C^{(55)}$	$C^{(60)}$	$C^{(59)}$	-173.2(2)	$C^{(76)}$	$C^{(81)}$	$B^{(1)}$	$C^{(88)}$	124.1(3)
$P^{(2)}$	$C^{(24)}$	$C^{(25)}$	$C^{(26)}$	178.7(3)	$C^{(76)}$	$C^{(81)}$	$B^{(1)}$	$C^{(82)}$	-119.0(3)
$P^{(2)}$	$C^{(24)}$	$C^{(29)}$	$C^{(28)}$	-179.8(3)	$C^{(76)}$	$C^{(77)}$	$C^{(78)}$	$C^{(79)}$	0.3(5)
$P^{(2)}$	$C^{(30)}$	$C^{(31)}$	$C^{(32)}$	-173.5(2)	$C^{(76)}$	$C^{(77)}$	$C^{(101)}$	$F^{(91)}$	-95.2(4)
$P^{(2)}$	$C^{(18)}$	$C^{(23)}$	$C^{(22)}$	172.2(2)	$C^{(76)}$	$C^{(77)}$	$C^{(101)}$	$F^{(22)}$	148.5(4)
$P^{(2)}$	$C^{(18)}$	$C^{(19)}$	$C^{(20)}$	-171.8(2)	$C^{(76)}$	$C^{(77)}$	$C^{(101)}$	$F^{(24)}$	22.7(5)
$O^{(1)}$	$C^{(10)}$	$C^{(11)}$	$Ir^{(1)1}$	91.9(3)	$C^{(87)}$	$C^{(82)}$	$C^{(83)}$	$C^{(84)}$	-4.3(5)
$N^{(1)}$	$C^{(1)}$	$C^{(10)}$	$O^{(1)}$	-12.0(4)	$C^{(87)}$	$C^{(82)}$	$B^{(1)}$	$C^{(70)}$	138.5(3)
$N^{(1)}$	$C^{(1)}$	$C^{(10)}$	$C^{(11)}$	161.4(3)	$C^{(87)}$	$C^{(82)}$	$B^{(1)}$	$C^{(88)}$	17.9(4)
$N^{(1)}$	$C^{(1)}$	$C^{(2)}$	$C^{(3)}$	-3.3(5)	$C^{(87)}$	$C^{(82)}$	$B^{(1)}$	$C^{(81)}$	-99.8(3)
$N^{(1)}$	$C^{(9)}$	$C^{(4)}$	$C^{(3)}$	-0.3(5)	$C^{(87)}$	$C^{(86)}$	$C^{(97)}$	$F^{(14)}$	41.6(4)
$N^{(1)}$	$C^{(9)}$	$C^{(4)}$	$C^{(5)}$	179.1(3)	$C^{(87)}$	$C^{(86)}$	$C^{(97)}$	$F^{(13)}$	-77.7(4)
$N^{(1)}$	$C^{(9)}$	$C^{(8)}$	$C^{(7)}$	-179.3(3)	$C^{(87)}$	$C^{(86)}$	$C^{(97)}$	$F^{(15)}$	162.2(3)
$C^{(1)}$	$N^{(1)}$	$C^{(9)}$	$C^{(4)}$	-4.7(4)	$C^{(87)}$	$C^{(86)}$	$C^{(85)}$	$C^{(84)}$	-2.4(5)
$C^{(1)}$	$N^{(1)}$	$C^{(9)}$	$C^{(8)}$	173.3(3)	$C^{(71)}$	$C^{(70)}$	$C^{(75)}$	$C^{(74)}$	-2.0(4)
$C^{(1)}$	$C^{(10)}$	$C^{(11)}$	$Ir^{(1)1}$	-81.1(3)	$C^{(71)}$	$C^{(70)}$	$B^{(1)}$	$C^{(88)}$	-60.0(3)
$C^{(1)}$	$C^{(2)}$	$C^{(3)}$	$C^{(4)}$	-2.0(5)	$C^{(71)}$	$C^{(70)}$	$B^{(1)}$	$C^{(81)}$	61.8(4)
$C^{(39)}$	$C^{(40)}$	$C^{(48)}$	$P^{(1)}$	-12.1(4)	$C^{(71)}$	$C^{(70)}$	$B^{(1)}$	$C^{(82)}$	178.9(3)
$C^{(39)}$	$C^{(40)}$	$C^{(48)}$	$C^{(47)}$	172.4(3)	$C^{(71)}$	$C^{(72)}$	$C^{(100)}$	$F^{(31)}$	-112.8(4)
$C^{(39)}$	$C^{(40)}$	$C^{(41)}$	$C^{(46)}$	-176.4(3)	$C^{(71)}$	$C^{(72)}$	$C^{(100)}$	$F^{(23)}$	136.9(4)
$C^{(39)}$	$C^{(40)}$	$C^{(41)}$	$C^{(42)}$	4.6(5)	$C^{(71)}$	$C^{(72)}$	$C^{(100)}$	$F^{(25)}$	9.4(6)
$C^{(39)}$	$C^{(38)}$	$C^{(33)}$	$C^{(32)}$	0.2(4)	$C^{(71)}$	$C^{(72)}$	$C^{(100)}$	$F^{(51)}$	74.0(6)
$C^{(39)}$	$C^{(38)}$	$C^{(33)}$	$C^{(34)}$	178.7(3)	$C^{(71)}$	$C^{(72)}$	$C^{(100)}$	$F^{(50)}$	-42.7(6)
$C^{(39)}$	$C^{(38)}$	$C^{(37)}$	$C^{(36)}$	-178.7(3)	$C^{(71)}$	$C^{(72)}$	$C^{(100)}$	$F^{(52)}$	-161.0(7)
$C^{(39)}$	$C^{(30)}$	$C^{(31)}$	$C^{(32)}$	0.0(4)	$C^{(5)}$	$C^{(4)}$	$C^{(3)}$	$C^{(2)}$	-175.8(3)

Table 6 Torsion Angles for exp_1880_auto.

A	B	C	D	Angle/˚	A	B	C	D	Angle/˚
C(10)	C(1)	C(2)	C(3)	175.8(3)	C(34)	C(35)	C(36)	C(37)	-0.5(5)
C(24)	P(2)	C(30)	C(39)	51.1(3)	C(51)	C(52)	C(53)	C(54)	0.2(5)
C(24)	P(2)	C(30)	C(31)	-135.5(2)	C(74)	C(73)	C(72)	C(71)	-0.5(5)
C(24)	P(2)	C(18)	C(23)	-174.7(2)	C(74)	C(73)	C(72)	C(100)	-179.2(3)
C(24)	P(2)	C(18)	C(19)	-1.5(3)	C(70)	C(71)	C(72)	C(73)	0.4(5)
C(24)	C(25)	C(26)	C(27)	1.6(5)	C(70)	C(71)	C(72)	C(100)	179.1(3)
C(24)	C(29)	C(28)	C(27)	0.6(5)	C(88)	C(89)	C(90)	C(92)	-0.7(5)
C(49)	P(1)	C(48)	C(40)	61.0(3)	C(88)	C(89)	C(90)	C(95)	-177.3(3)
C(49)	P(1)	C(48)	C(47)	-123.5(3)	C(58)	C(57)	C(56)	C(55)	-1.4(5)
C(49)	P(1)	C(55)	C(56)	167.6(2)	C(89)	C(88)	B(1)	C(70)	113.4(3)
C(49)	P(1)	C(55)	C(60)	-21.2(3)	C(89)	C(88)	B(1)	C(81)	-9.3(4)
C(49)	C(50)	C(51)	C(52)	-0.5(5)	C(89)	C(88)	B(1)	C(82)	-123.0(3)
C(49)	C(54)	C(53)	C(52)	-0.1(5)	C(89)	C(90)	C(95)	F(12)	-131.7(5)
C(40)	C(39)	C(38)	C(33)	179.6(3)	C(89)	C(90)	C(95)	F(27)	116.5(6)
C(40)	C(39)	C(38)	C(37)	0.2(4)	C(89)	C(90)	C(95)	F(58)	67.3(6)
C(40)	C(39)	C(30)	P(2)	-6.3(4)	C(89)	C(90)	C(95)	F(59)	-179.9(8)
C(40)	C(39)	C(30)	C(31)	-179.7(3)	C(89)	C(90)	C(95)	F(57)	-43.7(7)
C(40)	C(48)	C(47)	C(01Q)	4.2(5)	C(89)	C(90)	C(95)	F(11)	-19.1(7)
C(40)	C(41)	C(42)	C(43)	178.2(3)	C(80)	C(81)	B(1)	C(70)	-180.0(3)
C(48)	P(1)	C(49)	C(50)	-130.4(3)	C(80)	C(81)	B(1)	C(88)	-59.5(4)
C(48)	P(1)	C(49)	C(54)	49.7(3)	C(80)	C(81)	B(1)	C(82)	57.5(4)
C(48)	P(1)	C(55)	C(56)	58.5(3)	C(80)	C(79)	C(102)	F(21)	-149.2(4)
C(48)	P(1)	C(55)	C(60)	-130.3(3)	C(80)	C(79)	C(102)	F(19)	-29.3(5)
C(48)	C(40)	C(41)	C(46)	-0.6(5)	C(80)	C(79)	C(102)	F(20)	90.8(4)
C(48)	C(40)	C(41)	C(42)	-179.6(3)	C(75)	C(74)	C(73)	C(72)	-0.7(5)
C(48)	C(47)	C(01Q)	C(46)	-1.2(5)	C(75)	C(74)	C(99)	F(30)	-142.5(3)

Table 6 Torsion Angles for exp_1880_auto.

A	B	C	D	Angle/˚	A	B	C	D	Angle/˚
C(38)	C(39)	C(40)	C(48)	-105.6(3)	C(75)	C(74)	C(99)	F(33)	-19.6(5)
C(38)	C(39)	C(40)	C(41)	70.2(4)	C(75)	C(74)	C(99)	F(26)	99.5(4)
C(38)	C(39)	C(30)	P(2)	174.3(2)	C(75)	C(70)	B(1)	C(88)	120.2(3)
C(38)	C(39)	C(30)	C(31)	0.9(4)	C(75)	C(70)	B(1)	C(81)	-118.0(3)
C(38)	C(33)	C(34)	C(35)	0.6(5)	C(75)	C(70)	B(1)	C(82)	-0.9(4)
C(38)	C(37)	C(36)	C(35)	-0.8(5)	C(28)	C(27)	C(26)	C(25)	-0.4(6)
C(25)	C(24)	C(29)	C(28)	0.7(5)	C(73)	C(74)	C(75)	C(70)	2.0(5)
C(30)	P(2)	C(24)	C(25)	48.5(3)	C(73)	C(74)	C(99)	F(30)	41.4(5)
C(30)	P(2)	C(24)	C(29)	-131.0(3)	C(73)	C(74)	C(99)	F(33)	164.3(3)
C(30)	P(2)	C(18)	C(23)	75.7(3)	C(73)	C(74)	C(99)	F(26)	-76.5(4)
C(30)	P(2)	C(18)	C(19)	-111.1(3)	C(73)	C(72)	C(100)	F(31)	65.9(4)
C(30)	C(39)	C(40)	C(48)	75.0(4)	C(73)	C(72)	C(100)	F(23)	-44.3(5)
C(30)	C(39)	C(40)	C(41)	-109.2(3)	C(73)	C(72)	C(100)	F(25)	-171.9(5)
C(30)	C(39)	C(38)	C(33)	-1.0(4)	C(73)	C(72)	C(100)	F(51)	-107.3(5)
C(30)	C(39)	C(38)	C(37)	179.6(3)	C(73)	C(72)	C(100)	F(50)	136.0(6)
C(30)	C(31)	C(32)	C(33)	-0.8(5)	C(73)	C(72)	C(100)	F(52)	17.8(7)
C(18)	P(2)	C(24)	C(25)	-59.8(3)	C(45)	C(46)	C(41)	C(40)	-177.9(3)
C(18)	P(2)	C(24)	C(29)	120.7(3)	C(45)	C(46)	C(41)	C(42)	1.2(5)
C(18)	P(2)	C(30)	C(39)	160.2(2)	C(45)	C(46)	C(01Q)	C(47)	178.7(3)
C(18)	P(2)	C(30)	C(31)	-26.4(3)	C(90)	C(92)	C(93)	C(94)	0.9(5)
C(18)	C(23)	C(22)	C(21)	-0.4(5)	C(90)	C(92)	C(93)	C(96)	-174.3(3)
C(18)	C(19)	C(20)	C(21)	-0.1(5)	C(81)	C(76)	C(77)	C(78)	-0.4(5)
C(55)	P(1)	C(49)	C(50)	119.6(3)	C(81)	C(76)	C(77)	C(101)	178.1(3)
C(55)	P(1)	C(49)	C(54)	-60.3(3)	C(81)	C(80)	C(79)	C(78)	0.5(5)
C(55)	P(1)	C(48)	C(40)	169.6(3)	C(81)	C(80)	C(79)	C(102)	-178.1(3)
C(55)	P(1)	C(48)	C(47)	-15.0(3)	C(82)	C(87)	C(86)	C(97)	179.3(3)

Table 6 Torsion Angles for exp_1880_auto.

A	B	C	D	Angle/˚	A	B	C	D	Angle/˚
C(55)	C(60)	C(59)	C(58)	1.5(5)	C(82)	C(87)	C(86)	C(85)	1.6(5)
C(23)	C(18)	C(19)	C(20)	1.5(4)	C(82)	C(83)	C(84)	C(85)	3.7(6)
C(50)	C(49)	C(54)	C(53)	-0.2(5)	C(82)	C(83)	C(84)	C(98)	-178.1(4)
C(50)	C(51)	C(52)	C(53)	0.2(5)	C(77)	C(76)	C(81)	C(80)	0.5(5)
C(46)	C(41)	C(42)	C(43)	-0.9(5)	C(77)	C(76)	C(81)	B(1)	177.1(3)
C(46)	C(45)	C(44)	C(43)	-0.2(6)	C(77)	C(78)	C(79)	C(80)	-0.3(5)
C(9)	N(1)	C(1)	C(10)	-172.6(3)	C(77)	C(78)	C(79)	C(102)	178.3(3)
C(9)	N(1)	C(1)	C(2)	6.6(4)	C(86)	C(87)	C(82)	C(83)	1.7(5)
C(9)	C(4)	C(3)	C(2)	3.6(5)	C(86)	C(87)	C(82)	B(1)	174.5(3)
C(9)	C(4)	C(5)	C(6)	-0.7(5)	C(72)	C(71)	C(70)	C(75)	0.8(4)
C(9)	C(8)	C(7)	C(6)	1.1(6)	C(72)	C(71)	C(70)	B(1)	-179.0(3)
C(2)	C(1)	C(10)	O(1)	168.8(3)	C(78)	C(77)	C(101)	F(91)	83.4(4)
C(2)	C(1)	C(10)	C(11)	-17.8(4)	C(78)	C(77)	C(101)	F(22)	-33.0(5)
C(31)	C(32)	C(33)	C(38)	0.7(5)	C(78)	C(77)	C(101)	F(24)	-158.7(4)
C(31)	C(32)	C(33)	C(34)	-177.8(3)	C(78)	C(79)	C(102)	F(21)	32.1(5)
C(19)	C(18)	C(23)	C(22)	-1.2(5)	C(78)	C(79)	C(102)	F(19)	152.1(4)
C(19)	C(20)	C(21)	C(22)	-1.5(5)	C(78)	C(79)	C(102)	F(20)	-87.9(4)
C(4)	C(9)	C(8)	C(7)	-1.3(5)	C(101)	C(77)	C(78)	C(79)	-178.3(3)
C(4)	C(5)	C(6)	C(7)	0.5(6)	C(79)	C(80)	C(81)	C(76)	-0.6(5)
C(41)	C(40)	C(48)	P(1)	172.3(2)	C(79)	C(80)	C(81)	B(1)	-177.4(3)
C(41)	C(40)	C(48)	C(47)	-3.3(5)	C(83)	C(82)	B(1)	C(70)	-49.2(4)
C(41)	C(46)	C(01Q)	C(47)	-2.8(5)	C(83)	C(82)	B(1)	C(88)	-169.8(3)
C(41)	C(46)	C(45)	C(44)	-0.7(6)	C(83)	C(82)	B(1)	C(81)	72.5(4)
C(41)	C(42)	C(43)	C(44)	0.0(6)	C(83)	C(84)	C(85)	C(86)	-0.2(5)
C(56)	C(57)	C(58)	C(59)	1.0(5)	C(83)	C(84)	C(98)	F(18)	-2.0(6)
C(56)	C(55)	C(60)	C(59)	-1.9(5)	C(83)	C(84)	C(98)	F(54)	43.6(8)

Table 6 Torsion Angles for exp_1880_auto.

A	B	C	D	Angle/˚	A	B	C	D	Angle/˚
C(29)	C(24)	C(25)	C(26)	-1.8(5)	C(83)	C(84)	C(98)	F(17)	126.9(6)
C(29)	C(28)	C(27)	C(26)	-0.7(6)	C(83)	C(84)	C(98)	F(55)	155.7(6)
C(32)	C(33)	C(34)	C(35)	179.1(3)	C(83)	C(84)	C(98)	F(16)	-109.9(4)
C(20)	C(21)	C(22)	C(23)	1.8(5)	C(83)	C(84)	C(98)	F(53)	-71.7(17)
C(92)	C(93)	C(94)	C(88)	-2.2(5)	C(97)	C(86)	C(85)	C(84)	180.0(3)
C(92)	C(93)	C(96)	F(29)	-154.2(4)	C(85)	C(86)	C(97)	F(14)	-140.7(3)
C(92)	C(93)	C(96)	F(32)	-16.1(5)	C(85)	C(86)	C(97)	F(13)	100.0(4)
C(92)	C(93)	C(96)	F(28)	94.0(4)	C(85)	C(86)	C(97)	F(15)	-20.0(5)
C(92)	C(93)	C(96)	F(56)	-86.7(6)	C(85)	C(84)	C(98)	F(18)	176.3(4)
C(92)	C(90)	C(95)	F(12)	51.6(6)	C(85)	C(84)	C(98)	F(54)	-138.1(7)
C(92)	C(90)	C(95)	F(27)	-60.2(7)	C(85)	C(84)	C(98)	F(17)	-54.9(7)
C(92)	C(90)	C(95)	F(58)	-109.4(6)	C(85)	C(84)	C(98)	F(55)	-26.0(7)
C(92)	C(90)	C(95)	F(59)	3.3(10)	C(85)	C(84)	C(98)	F(16)	68.4(5)
C(92)	C(90)	C(95)	F(57)	139.5(5)	C(85)	C(84)	C(98)	F(53)	106.6(16)
C(92)	C(90)	C(95)	F(11)	164.2(5)	C(96)	C(93)	C(94)	C(88)	173.1(3)
C(3)	C(4)	C(5)	C(6)	178.7(3)	C(99)	C(74)	C(75)	C(70)	-174.0(3)
C(93)	C(92)	C(90)	C(89)	0.4(5)	C(99)	C(74)	C(73)	C(72)	175.4(3)
C(93)	C(92)	C(90)	C(95)	177.1(3)	C(98)	C(84)	C(85)	C(86)	-178.5(4)
C(93)	C(94)	C(88)	C(89)	1.9(4)	B(1)	C(70)	C(75)	C(74)	177.8(3)
C(93)	C(94)	C(88)	B(1)	-175.8(3)	B(1)	C(88)	C(89)	C(90)	177.1(3)
C(8)	C(9)	C(4)	C(3)	-178.4(3)	B(1)	C(82)	C(83)	C(84)	-177.1(3)

[1]2-X,2-Y,1-Z

Table 7 Hydrogen Atom Coordinates (Å×10^4) and Isotropic Displacement Parameters (Å^2×10^3) for exp_1880_auto.

Atom	*x*	*y*	*z*	U(eq)
H(11A)	9121.32	11051.56	4021.85	17

Table 7 Hydrogen Atom Coordinates (Å×10^4) and Isotropic Displacement Parameters (Å^2×10^3) for exp_1880_auto.

**Atom**	***x***	***y***	***z***	**U(eq)**
H(11B)	8626.24	10218.38	3381.22	17
H(57)	6510.46	10409.6	3415.14	24
H(25)	10333.74	12172.81	8727.67	23
H(23)	10964.96	9357.5	7769.13	20
H(50)	8352.27	8738.53	6995.89	23
H(2)	8101.32	9010.56	3158.11	21
H(31)	10197.01	9845.17	8688.99	21
H(19)	11773.12	11757.41	8344.37	22
H(56)	7420.29	10786.74	4794.12	20
H(29)	9515.01	11722.64	6268.74	21
H(47)	6679.03	11369.83	5568.38	22
H(20)	13422.2	11499.16	8894.52	25
H(92)	940.2	6491.77	5788.54	29
H(3)	7702.9	7646.12	2990.51	25
H(8)	9286.7	8142.47	6183.88	23
H(54)	5841.56	9880.48	6413.05	24
H(60)	6295.3	8739.57	5137.75	23
H(94)	3938.67	6606.9	6441.35	22
H(42)	8450.61	12208.2	9015.71	27
H(01Q)	6573.17	12706.3	6118.96	26
H(37)	6479.27	11242.06	8070.28	24
H(76)	4942.62	3880.75	7519.74	22
H(87)	3989.77	7015.24	7997.78	23
H(71)	3882.63	4140.44	6102.2	22
H(5)	7829.31	6447.01	3603.58	31
H(21)	13846.53	10176.65	8860.45	26

Table 7 Hydrogen Atom Coordinates (Å×10^4) and Isotropic Displacement Parameters (Å^2×10^3) for exp_1880_auto.

Atom	*x*	*y*	*z*	U(eq)
H(34)	7739.39	9595.19	9867.61	27
H(51)	7584.02	7913.42	7568.01	28
H(59)	5390.63	8371.24	3761.7	29
H(58)	5522.93	9193.04	2897.73	28
H(89)	2448.07	4981.68	7102.32	24
H(80)	3621.3	5701.97	8568.5	25
H(75)	6238.71	5792.15	7018.85	22
H(22)	12610.91	9104.56	8327.91	25
H(28)	9531.04	13120.35	6354.46	31
H(27)	9983.56	14025.65	7617.6	32
H(73)	6136.82	3927.86	5123.11	26
H(45)	6969.22	13884.75	7304.5	33
H(35)	6217.51	10098.66	9776.59	29
H(52)	5963.06	8074.14	7574.08	29
H(53)	5089.38	9058.09	6999.55	30
H(43)	8337.93	13559.24	9543.06	34
H(26)	10394.53	13560.95	8799.63	29
H(36)	5586.86	10921.62	8865.38	27
H(78)	3967.38	3665.44	9403.14	30
H(7)	8999.45	6769.43	6024.95	32
H(83)	6434.17	5790.25	8308.81	26
H(6)	8287.63	5915.99	4729.56	35
H(85)	6590.32	8085.85	9621.24	29
H(44)	7605.19	14400.69	8684.81	39

Table 8 Atomic Occupancy for exp_1880_auto.

Atom	*Occupancy*	Atom	*Occupancy*	Atom	*Occupancy*
F(29)	0.934(11)	F(32)	0.979(11)	F(31)	0.728(6)
F(18)	0.636(10)	F(28)	0.834(10)	F(23)	0.728(6)
F(25)	0.728(6)	F(12)	0.525(12)	F(27)	0.525(12)
F(51)	0.272(6)	F(50)	0.272(6)	F(52)	0.272(6)
F(58)	0.475(12)	F(59)	0.475(12)	F(56)	0.272(12)
F(57)	0.475(12)	F(11)	0.525(12)	F(54)	0.364(10)
F(17)	0.636(10)	F(55)	0.364(10)	F(16)	0.789(14)
F(53)	0.211(14)				

Table 9 Solvent masks information for exp_1880_auto.

Number	X	Y	Z	Volume	Electron count	Content
1	0.000	0.500	0.000	446.4	113.4	?
2	0.210	0.365	0.778	8.0	0.0	?
3	0.790	0.635	0.222	8.0	0.0	?

Experimental

Single crystals of $C_{87}H_{51}BF_{24}IrNOP_2$ were submitted for X-ray determination. A suitable crystal was selected and mounted on a MiTeGen tip using Parabol oil and placed on a XtaLAB AFC12 (RCD3): Kappa single diffractometer. The crystal was kept at 100.0(5) K during data collection. Using Olex2[14], the structure was solved with the XT[15] structure solution program using Intrinsic Phasing and refined with the SHELXL[16] refinement package using Least Squares minimisation. There was significant solvent disorder in the model, and it was not possible to locate the solvent positions. A SQUEEZE algorithm was applied, resulting in the masking of 113 electrons found within solvent voids. This value suggests there is one DCM and one MeOH residing in the asymmetric unit. Furthermore, it was not possible to locate the hydride due to high Ir absorption. However, it is clear via the Ir geometry that a hydride is present (square pyramidal geometry in the model; octahedral geometry if a hydride is present). Furthermore, this assignment bodes well with the ^{1}H NMR data, which clearly illustrates an Ir-H environment.

Crystal structure determination of [exp_1880_auto]

Crystal Data for $C_{87}H_{51}BF_{24.02}IrNOP_2$ (M =1847.61 g/mol): triclinic, space group P-1 (no. 2), a = 14.18320(10) Å, b = 17.0432(2) Å, c = 17.74540(10) Å, α = 102.7360(10)°, β = 107.1920(10)°, γ = 90.7360(10)°, V = 3983.03(6) Å^3, Z = 2, T = 100.0(5) K, μ(Mo Kα) = 1.820 mm^{-1}, $Dcalc$ = 1.541 g/cm^3, 162727 reflections measured (3.262° ≤ 2Θ ≤ 53°), 16505 unique (R_{int} = 0.1001, R_{sigma} = 0.0409) which were used in all calculations. The final R_1 was 0.0343 (I > 2σ(I)) and wR_2 was 0.0871 (all data).

Refinement model description

Number of restraints - 0, number of constraints - unknown.

Details:

1. Fixed Uiso
 At 1.2 times of:
  All C(H) groups, All C(H,H) groups
2. Others
 Sof(F51)=Sof(F50)=Sof(F52)=1-FVAR(1)
 Sof(F31)=Sof(F23)=Sof(F25)=FVAR(1)
 Sof(F58)=Sof(F59)=Sof(F57)=1-FVAR(2)
 Sof(F12)=Sof(F27)=Sof(F11)=FVAR(2)
 Sof(F54)=Sof(F55)=1-FVAR(3)
 Sof(F18)=Sof(F17)=FVAR(3)
 Sof(F53)=1-FVAR(4)
 Sof(F16)=FVAR(4)
3.a Secondary CH2 refined with riding coordinates:
 C11(H11A,H11B)
3.b Aromatic/amide H refined with riding coordinates:
 C57(H57), C25(H25), C23(H23), C50(H50), C2(H2), C31(H31), C19(H19), C56(H56), C29(H29), C47(H47), C20(H20), C92(H92), C3(H3), C8(H8), C54(H54), C60(H60), C94(H94), C42(H42), C01Q(H01Q), C37(H37), C76(H76), C87(H87), C71(H71), C5(H5), C21(H21), C34(H34), C51(H51), C59(H59), C58(H58), C89(H89), C80(H80), C75(H75), C22(H22), C28(H28), C27(H27), C73(H73), C45(H45), C35(H35), C52(H52), C53(H53), C43(H43), C26(H26), C36(H36), C78(H78), C7(H7), C83(H83), C6(H6), C85(H85), C44(H44)

This report has been created with Olex2, compiled on 2020.11.12 svn.r5f609507 for OlexSys.

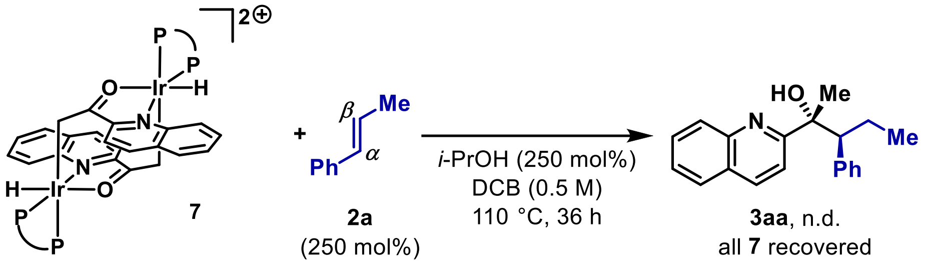

Scheme S29. Reaction of **7** under standard conditions.

GP6: A flame-dried Schlenk tube was charged with crystal **7** (185 mg, 0.10 mmol, 100 mol%, 0.98 D). The Schlenk tube was evacuated and backfilled with N_2 for three times. Then DCB (0.2 mL, 0.5 M) was added, followed by the addition of (*E*)-1-propenylbenzene (**2a**, 32.4 µL, 29.5 mg, 0.25 mmol, 250 mol%) and *i*-PrOH (19.1 µL, 15.0 mg, 0.25 mmol, 250 mol%). The tube was sealed and heated at 110 °C for 24 h. After cooling to room temperature, Filtration of the reaction mixture afforded a yellow solid (crystal **7**, 180 mg, 97% Yield).

7 NMR Spectra

^{1}H NMR (500 MHz, CD_3Cl, 298 K) and ^{13}C NMR (125 MHz, CD_3Cl, 298 K) of **2g**.

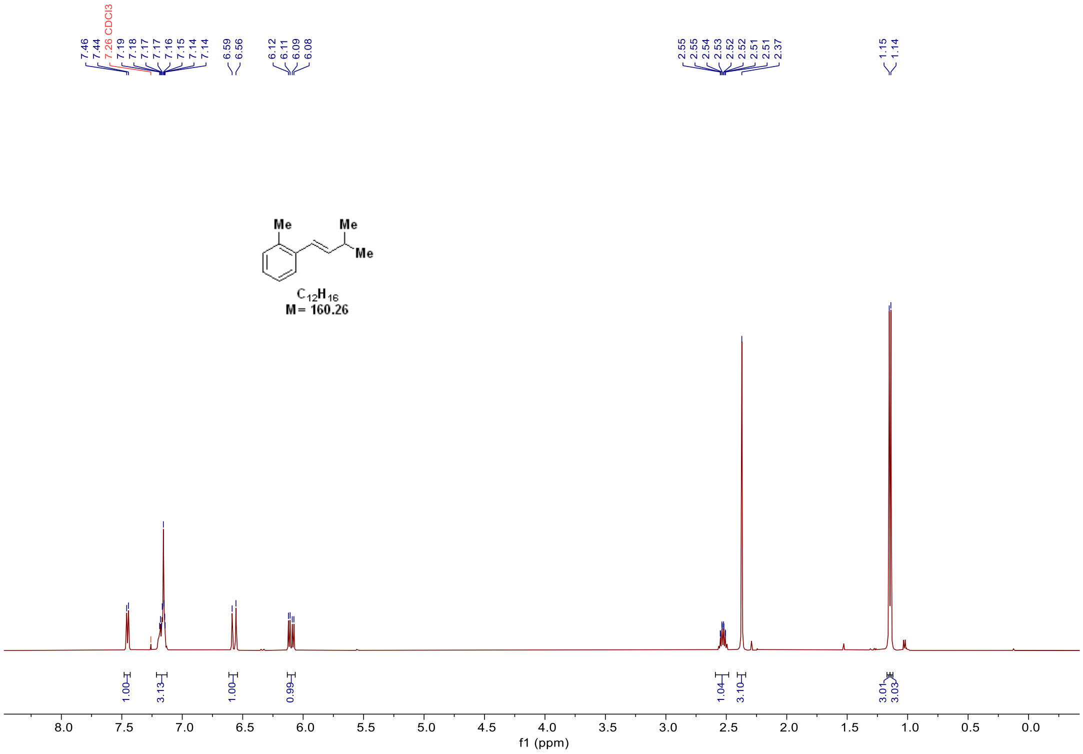

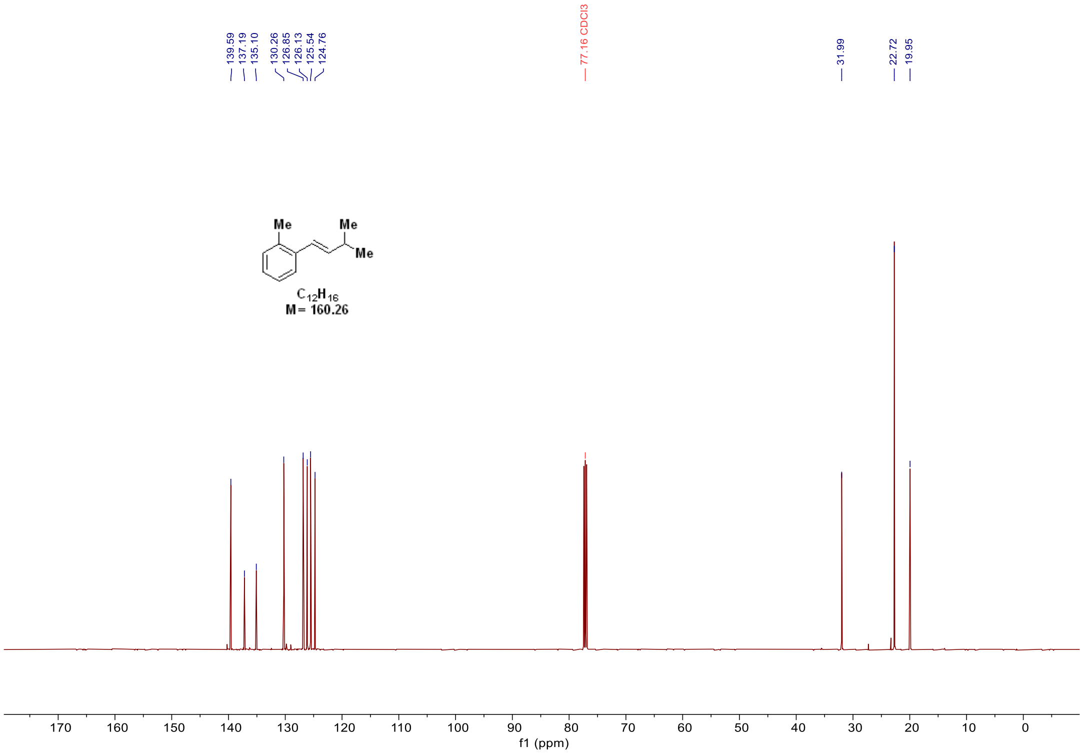

^{1}H NMR (500 MHz, CD_3Cl, 298 K), ^{13}C NMR (125 MHz, CD_3Cl, 298 K) and ^{19}F NMR (471 MHz, CD_3Cl, 298 K) of $[Ir(cod)_2]BARF$

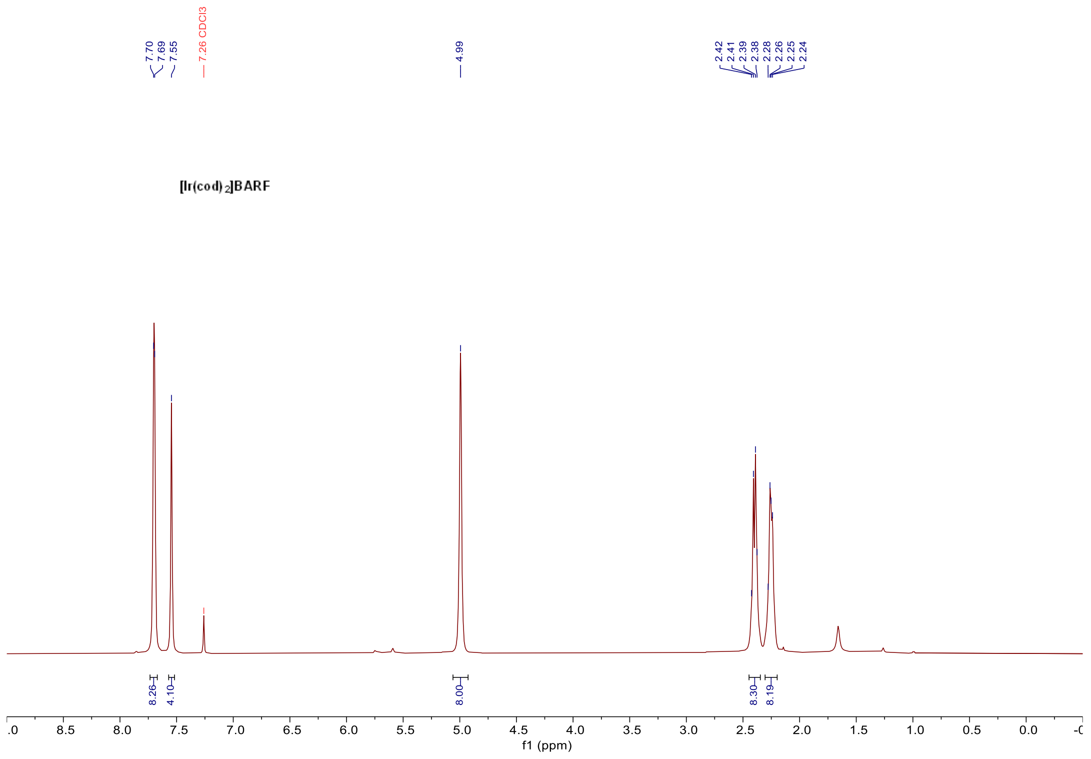

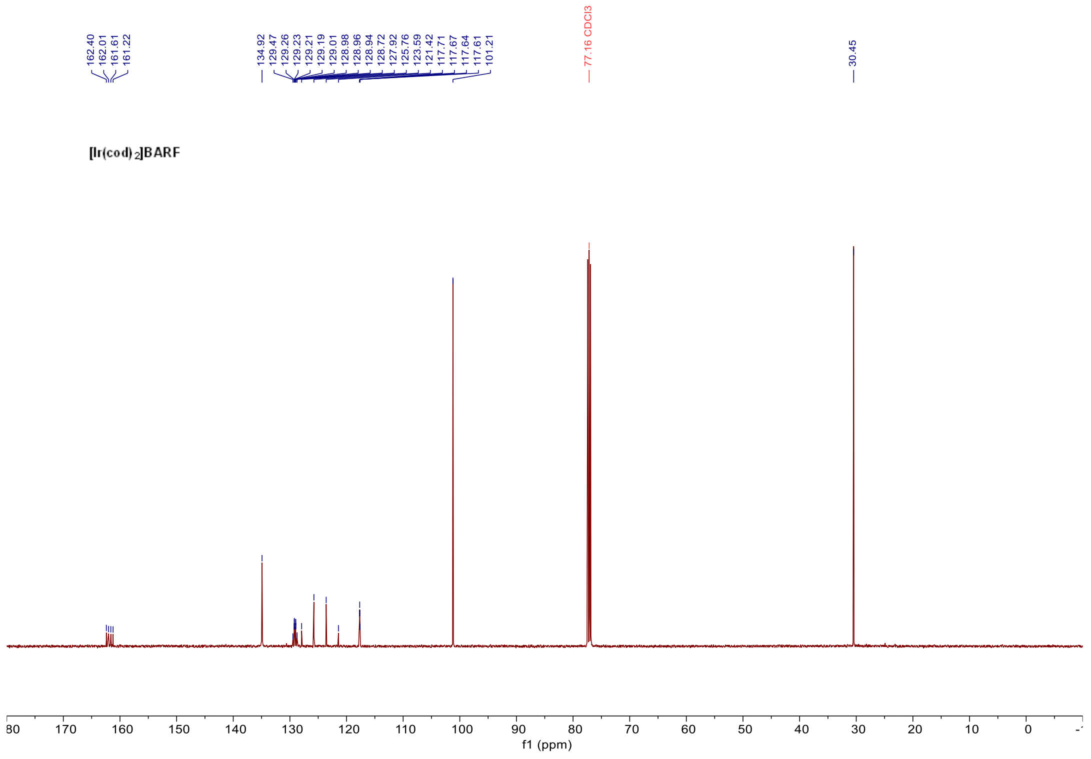

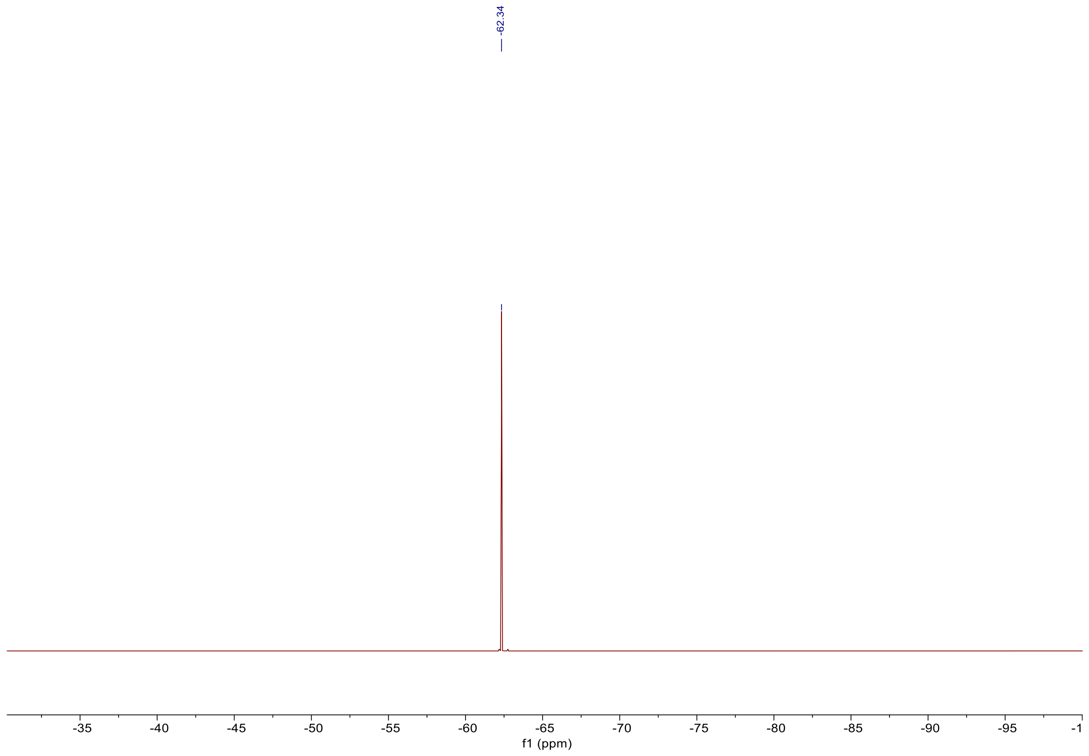
-62.34
-35
-40
-45
-50
-55
-60
-65
-70
-75
-80
-85
-90
-95
f1 (ppm)

^{1}H NMR (500 MHz, CD_3Cl, 298 K) and ^{13}C NMR (125 MHz, CD_3Cl, 298 K) of **3aa**.

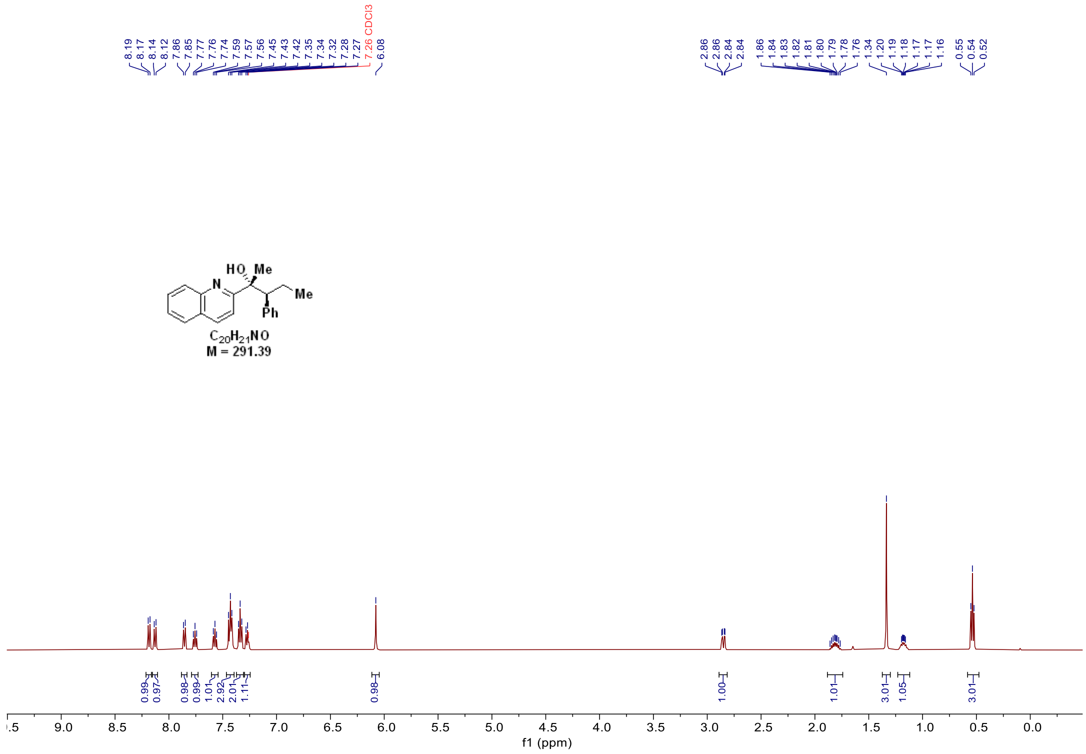

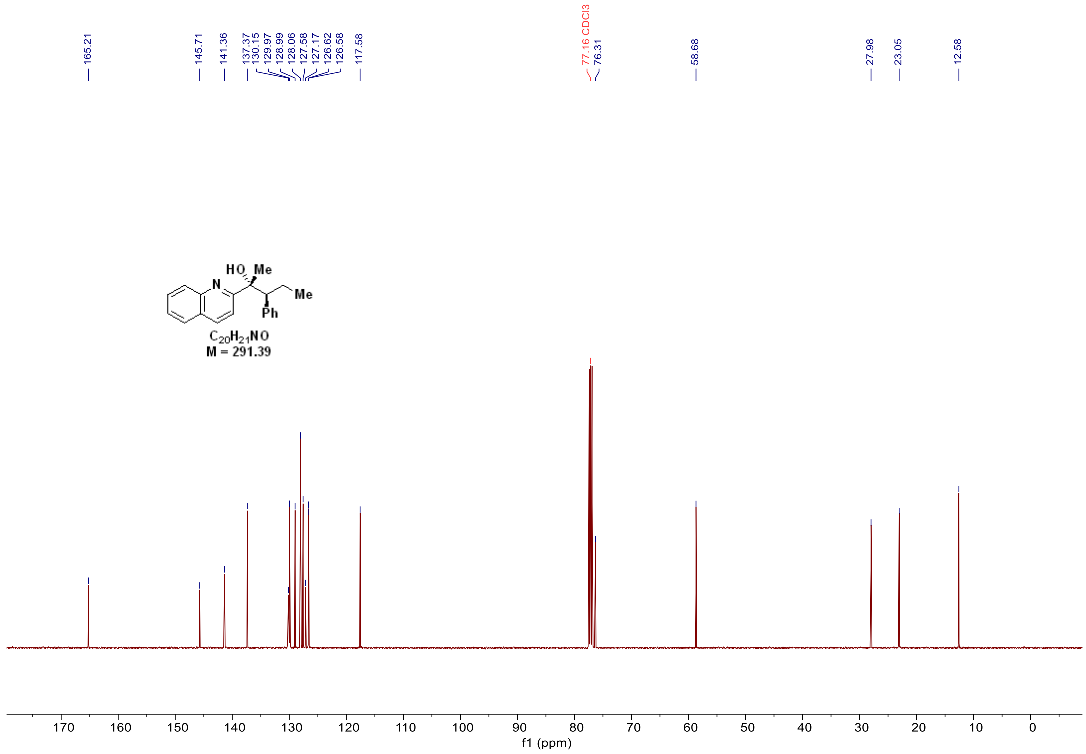

^{1}H NMR (500 MHz, CD_3Cl, 298 K) and ^{13}C NMR (125 MHz, CD_3Cl, 298 K) of **3ab**.

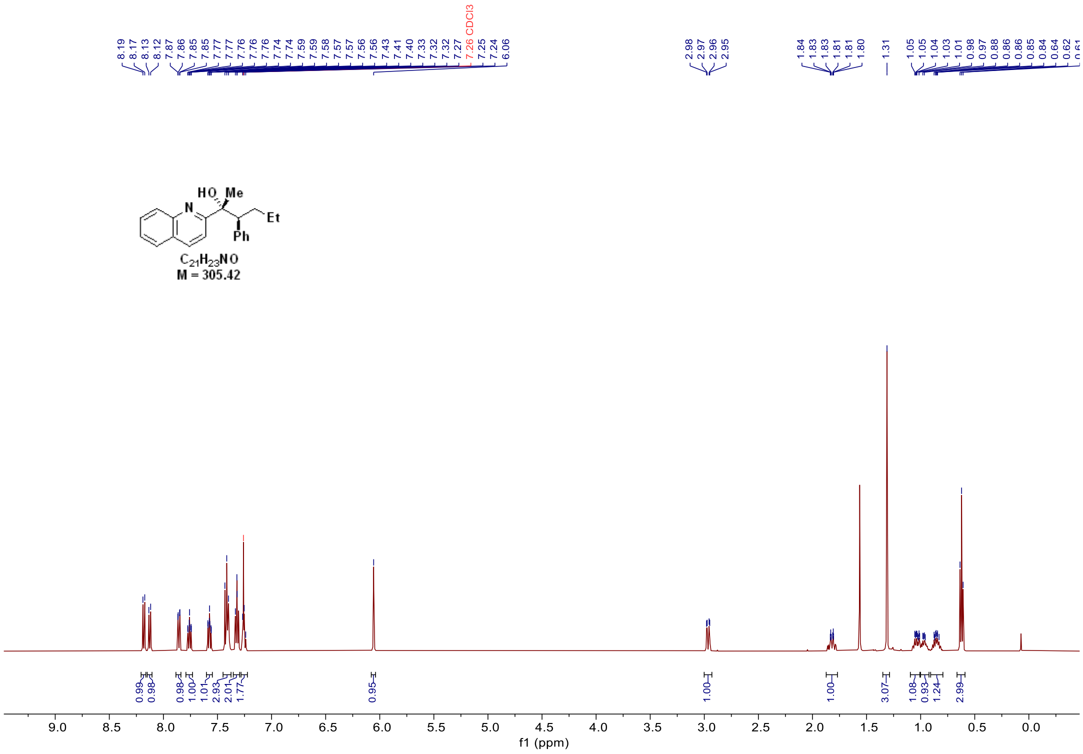

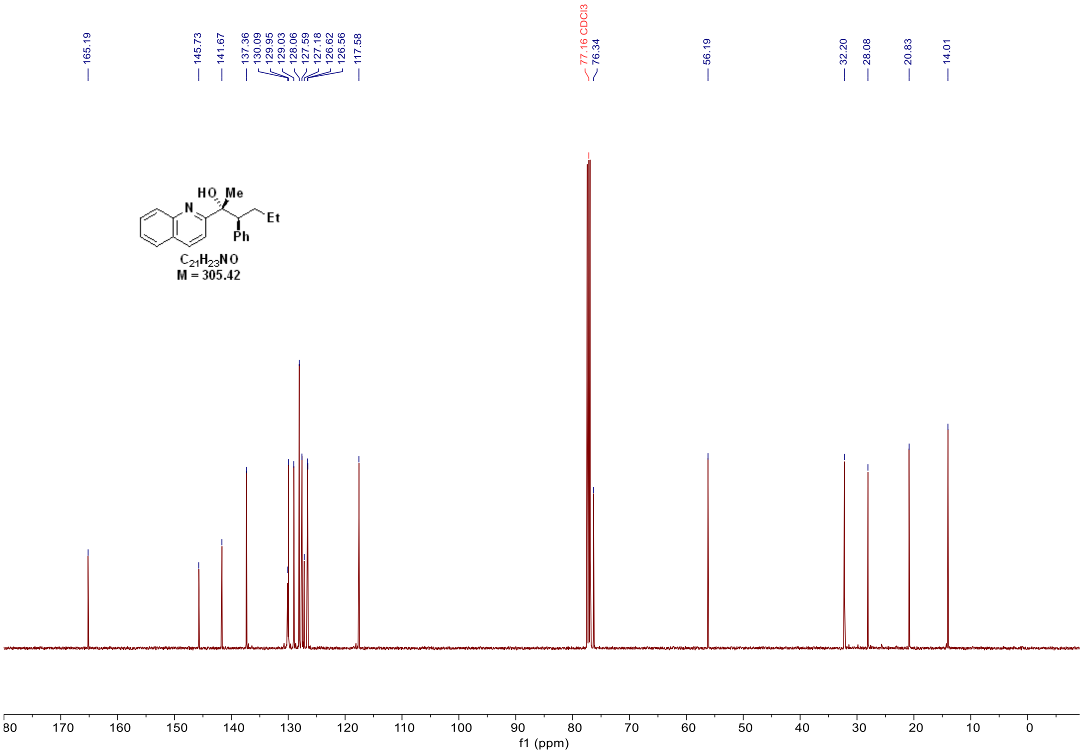

^1H NMR (500 MHz, CD_3Cl, 298 K) and ^{13}C NMR (125 MHz, CD_3Cl, 298 K) of **3ac**.

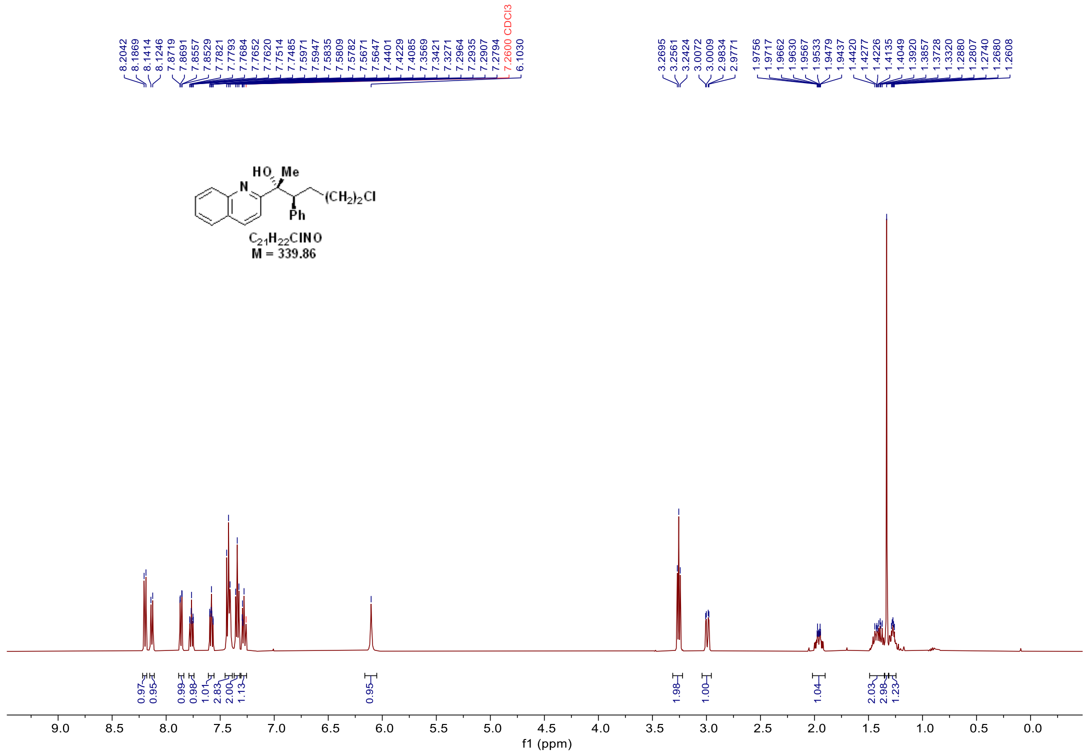

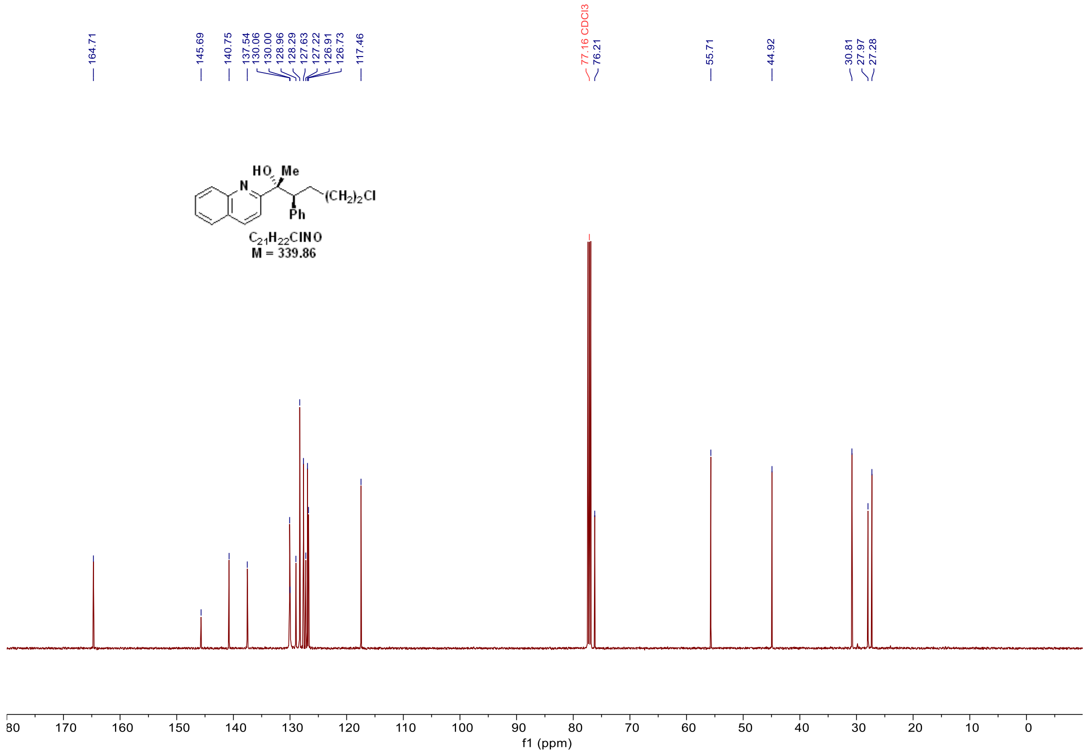

^{1}H NMR (500 MHz, CD_3Cl, 298 K) and ^{13}C NMR (125 MHz, CD_3Cl, 298 K) of **3ad**.

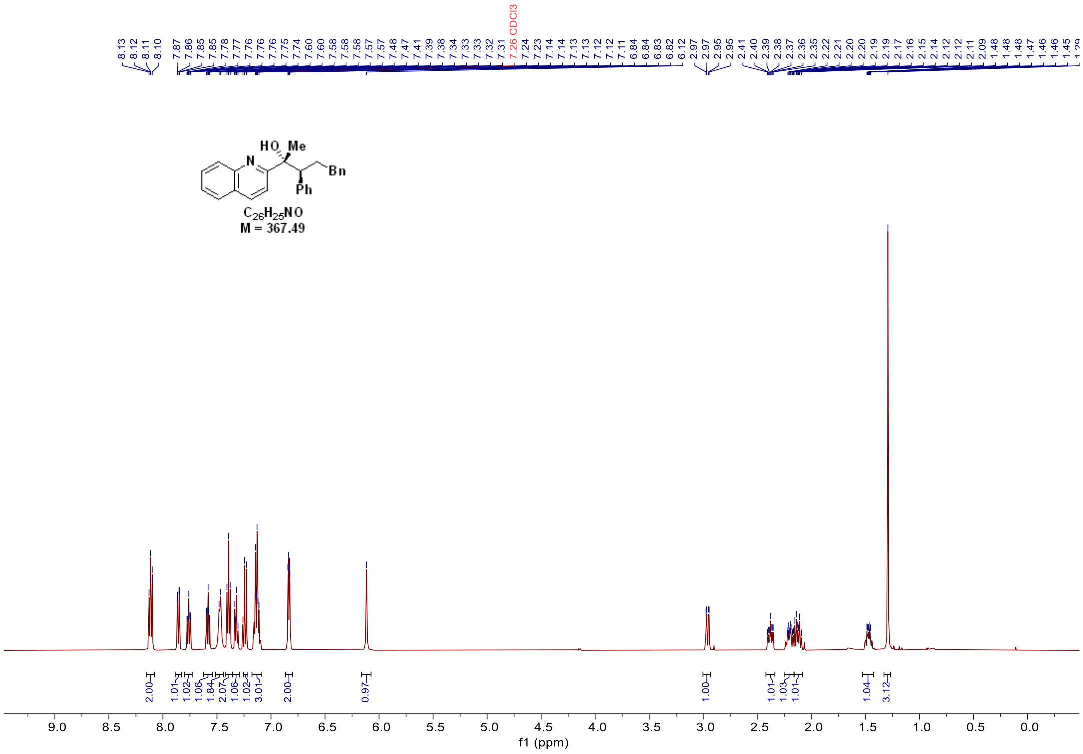

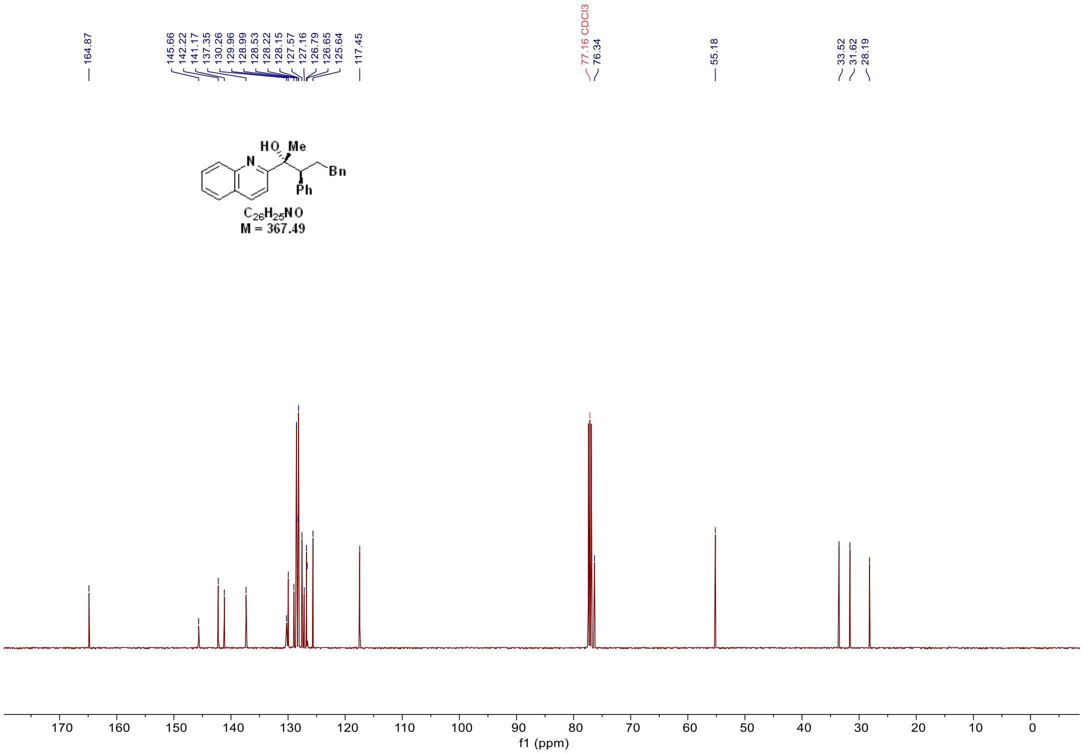

^{1}H NMR (500 MHz, CD_3Cl, 298 K) and ^{13}C NMR (125 MHz, CD_3Cl, 298 K) of **3ae**.

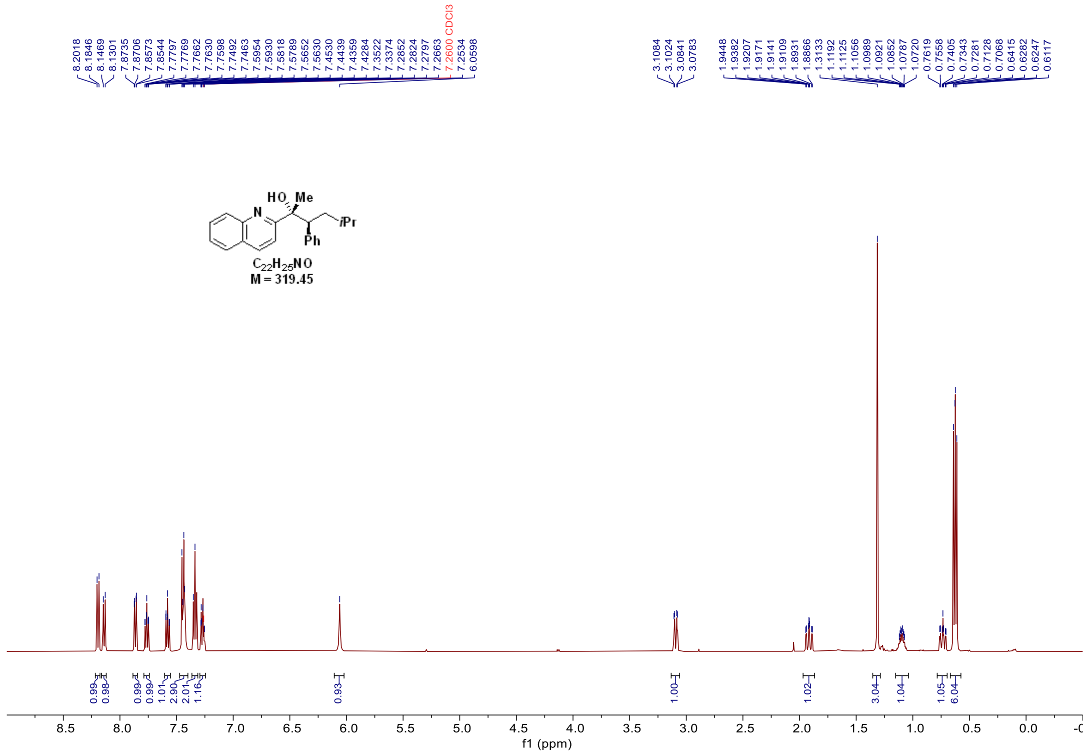

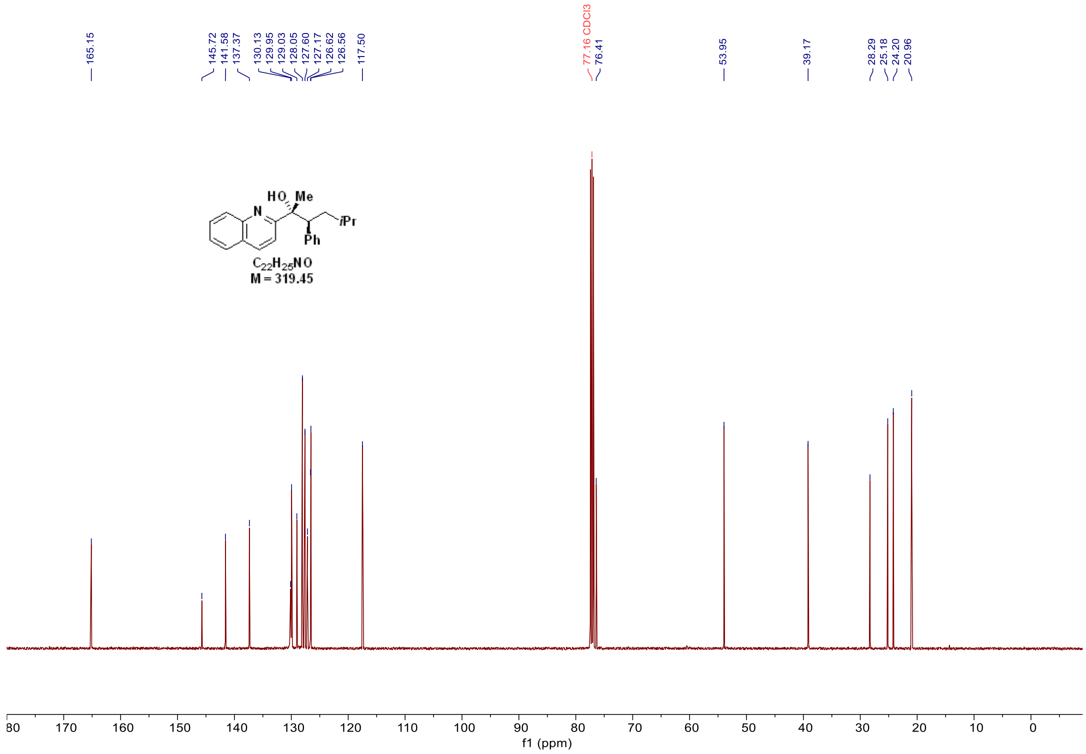

^{1}H NMR (500 MHz, CD_3Cl, 298 K) and ^{13}C NMR (125 MHz, CD_3Cl, 298 K) of **3af**.

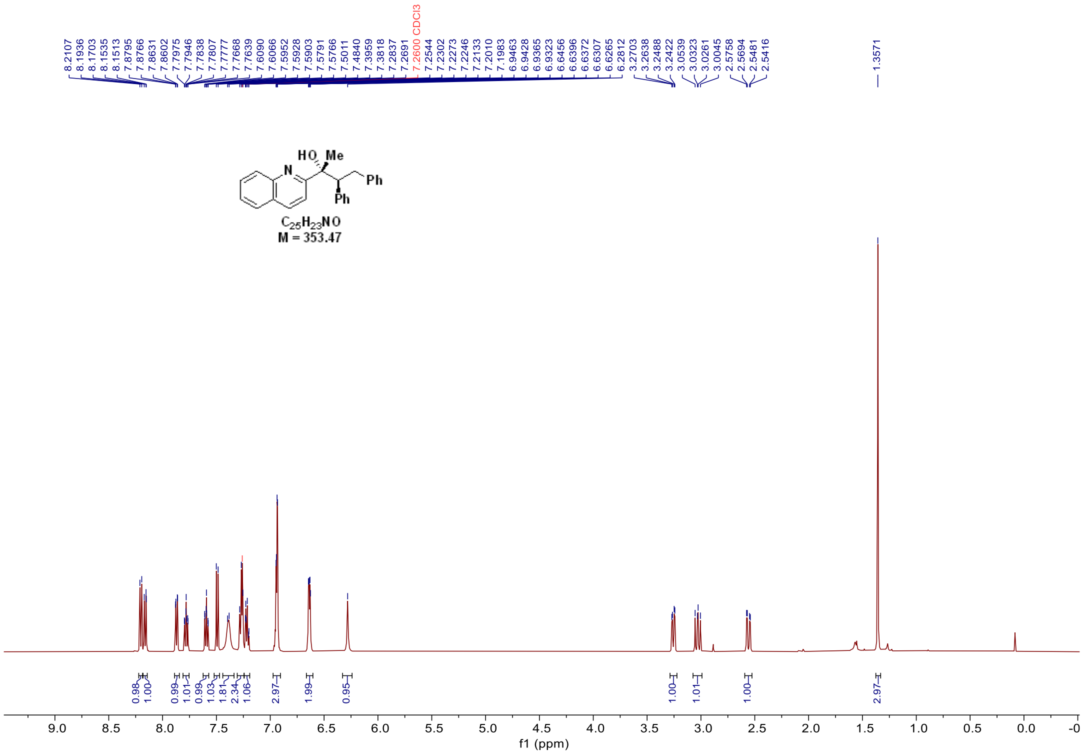

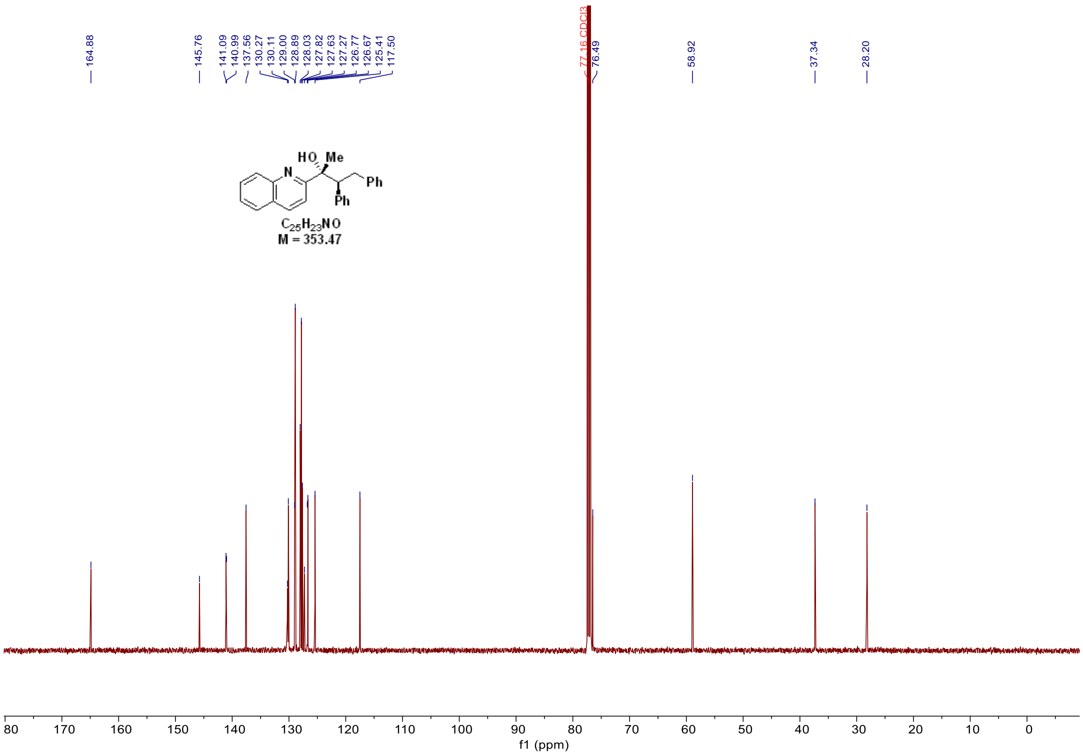

^{1}H NMR (500 MHz, CD_3Cl, 298 K) and ^{13}C NMR (125 MHz, CD_3Cl, 298 K) of **3ag**.

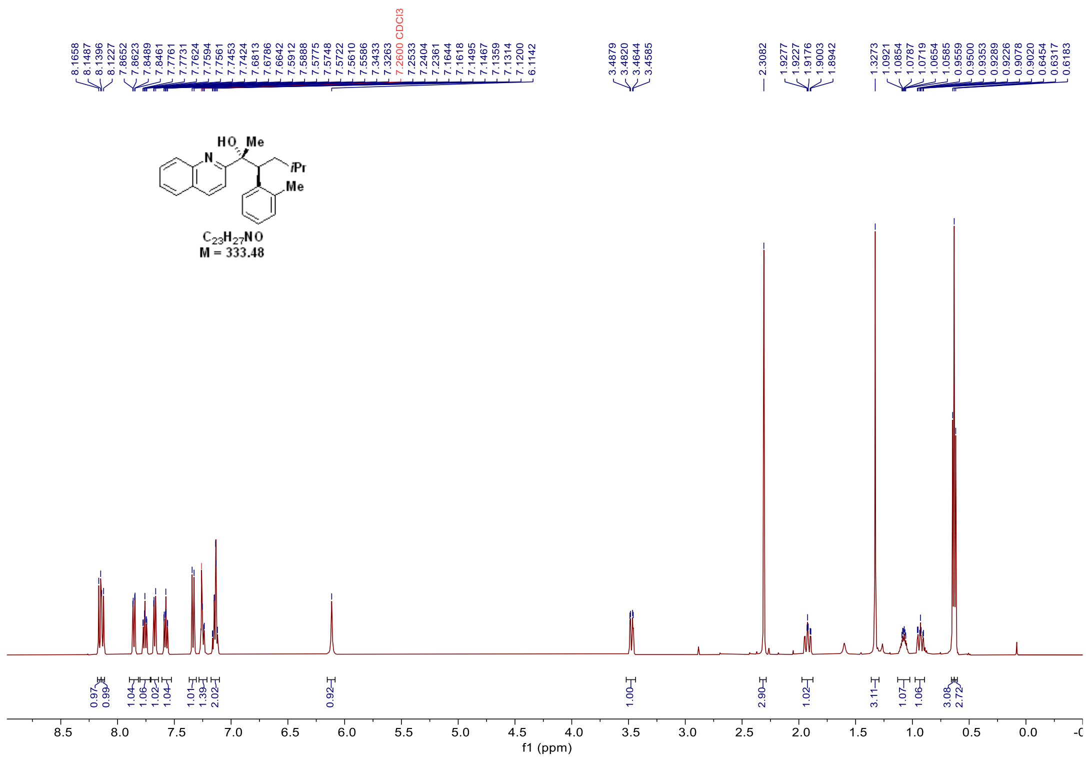

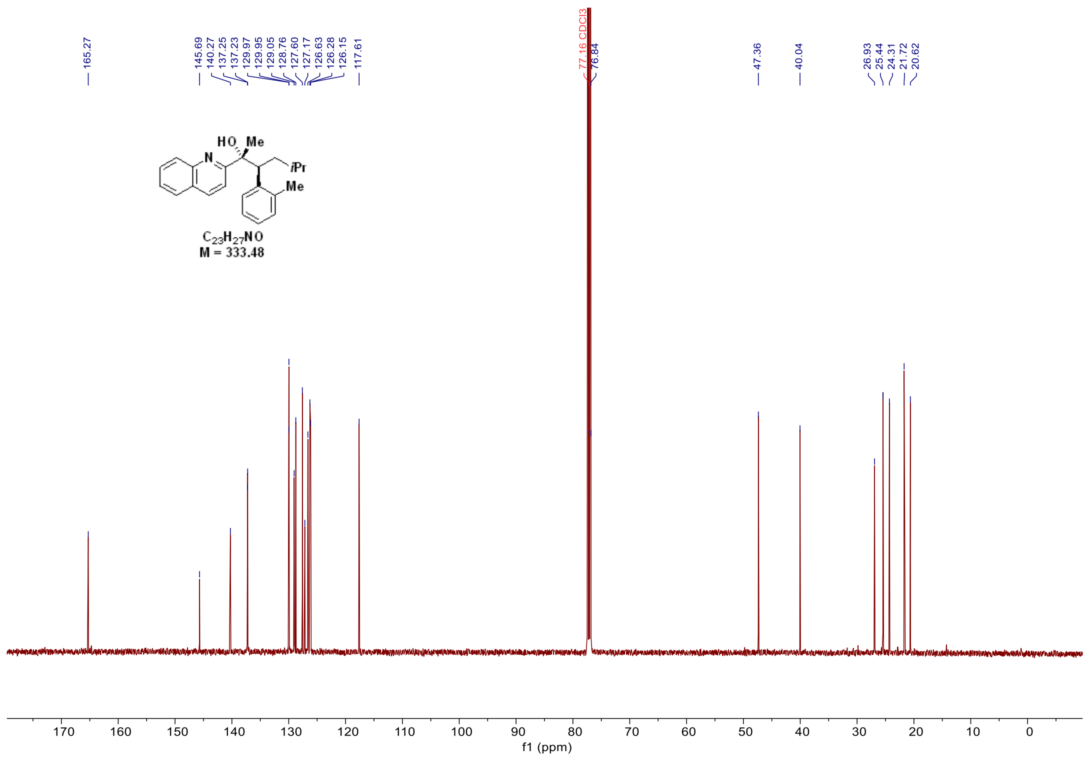

^{1}H NMR (500 MHz, CD_3Cl, 298 K) and ^{13}C NMR (125 MHz, CD_3Cl, 298 K) of **3ah**.

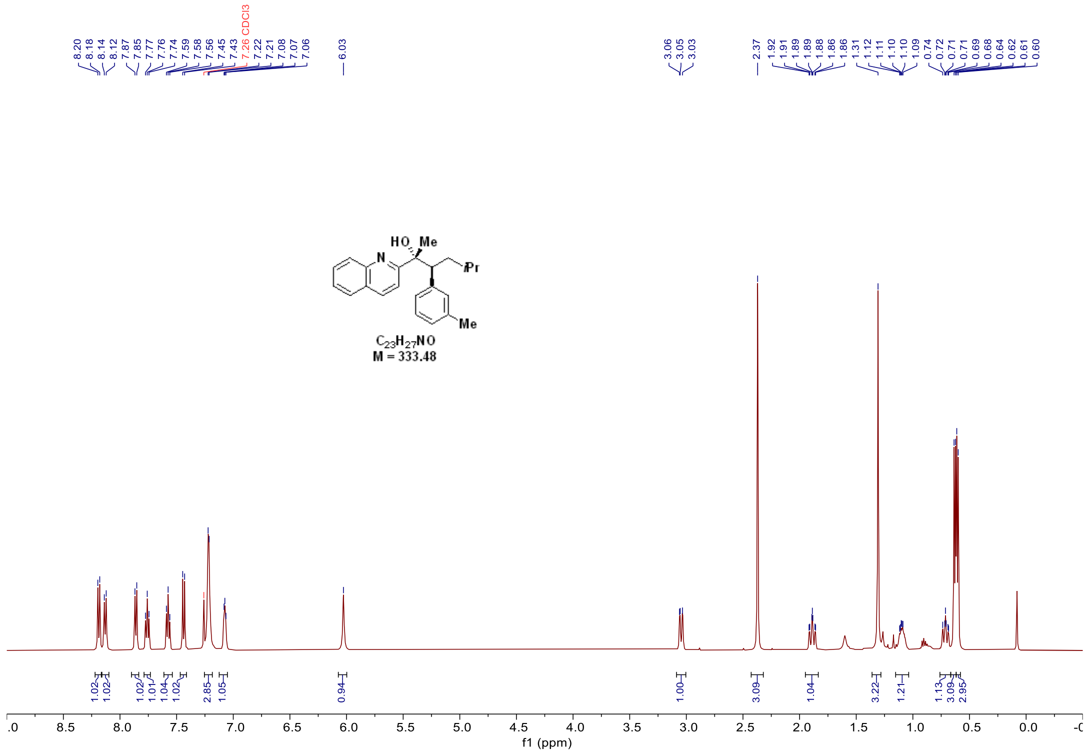

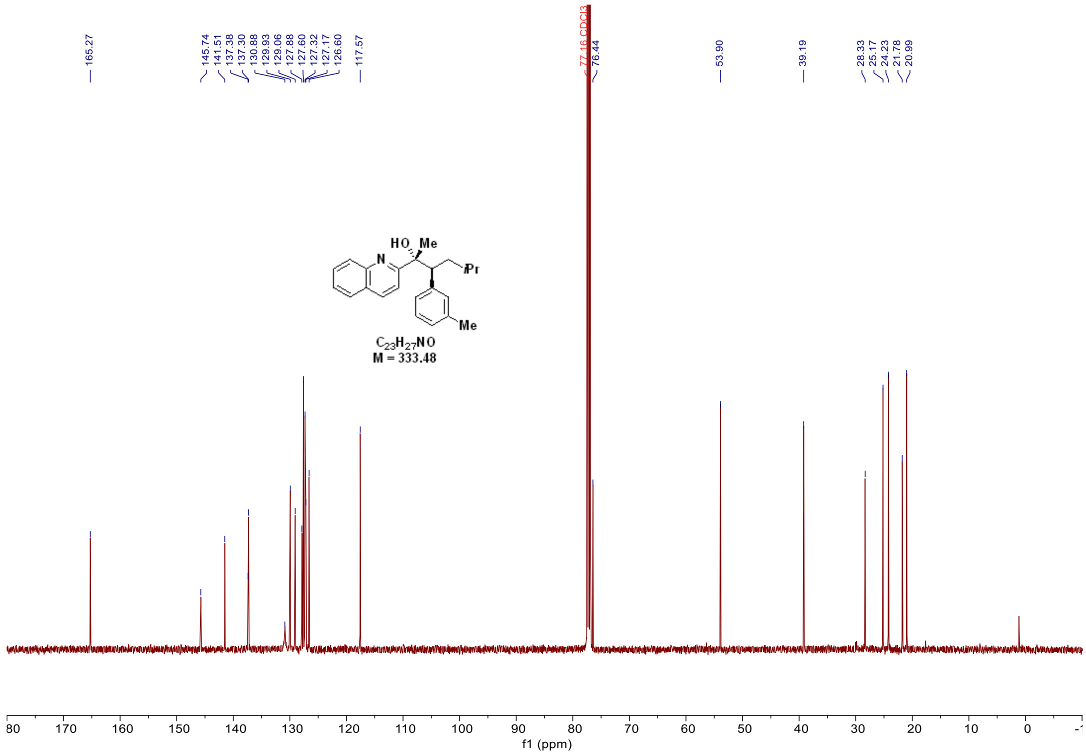

^{1}H NMR (500 MHz, CD_3Cl, 298 K) and ^{13}C NMR (125 MHz, CD_3Cl, 298 K) of **3ai**.

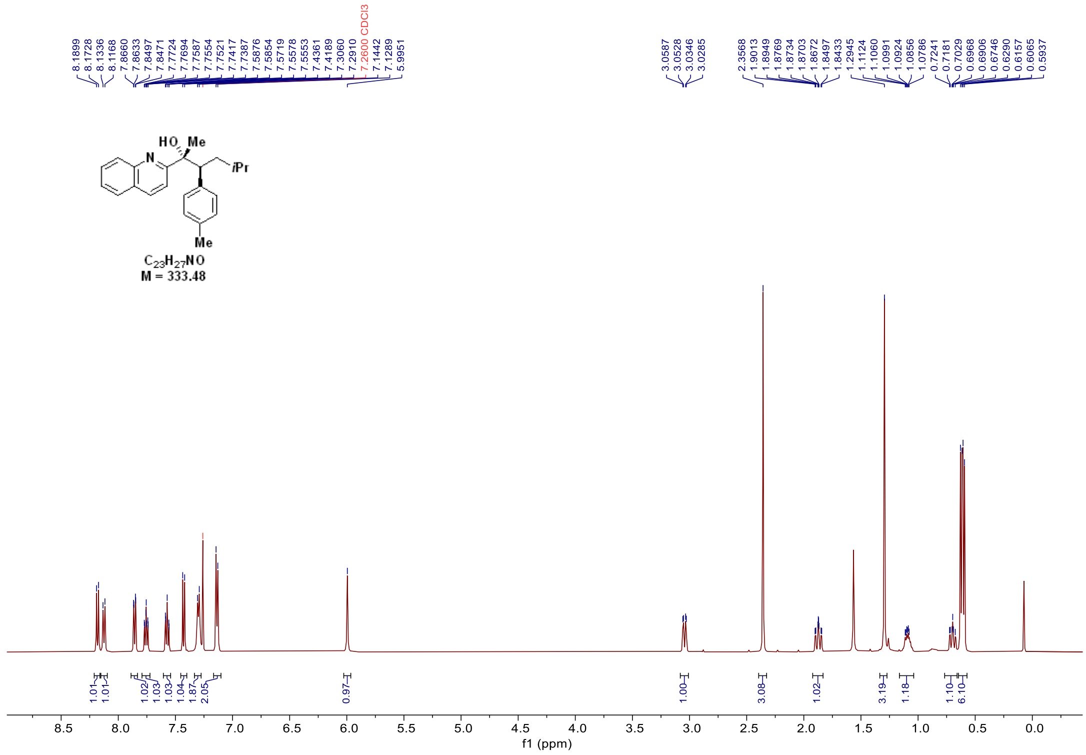

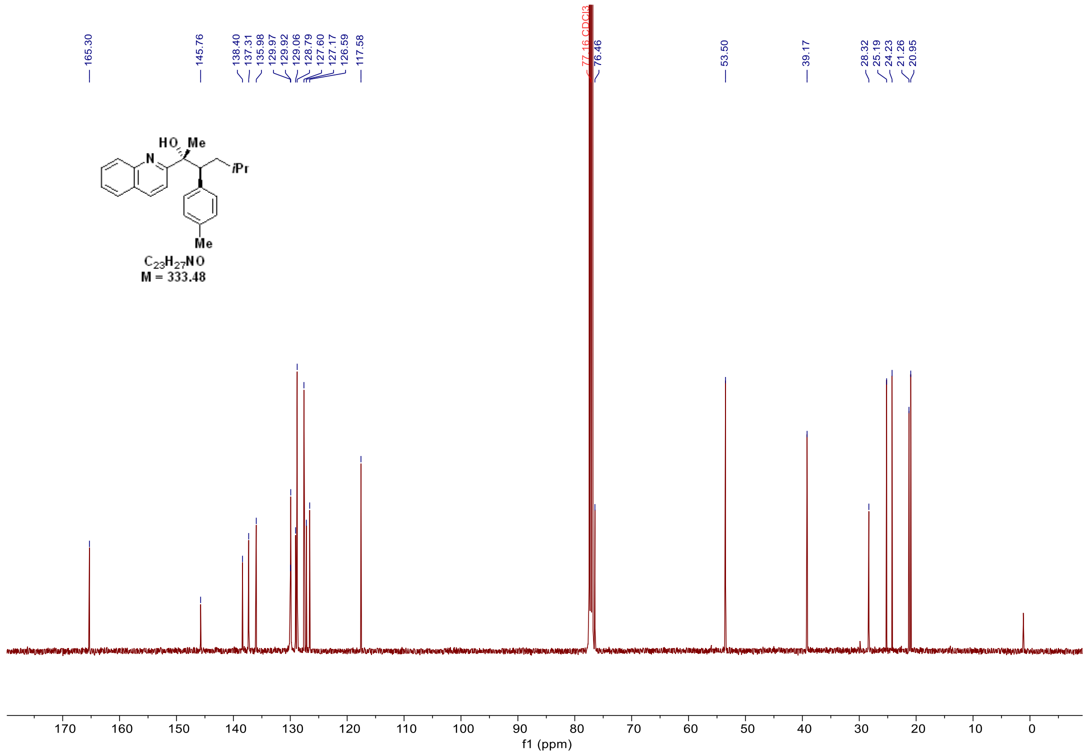

^{1}H NMR (500 MHz, CD_3Cl, 298 K), ^{13}C NMR (125 MHz, CD_3Cl, 298 K) and ^{19}F NMR (471 MHz, CD_3Cl, 298 K) of **3aj**.

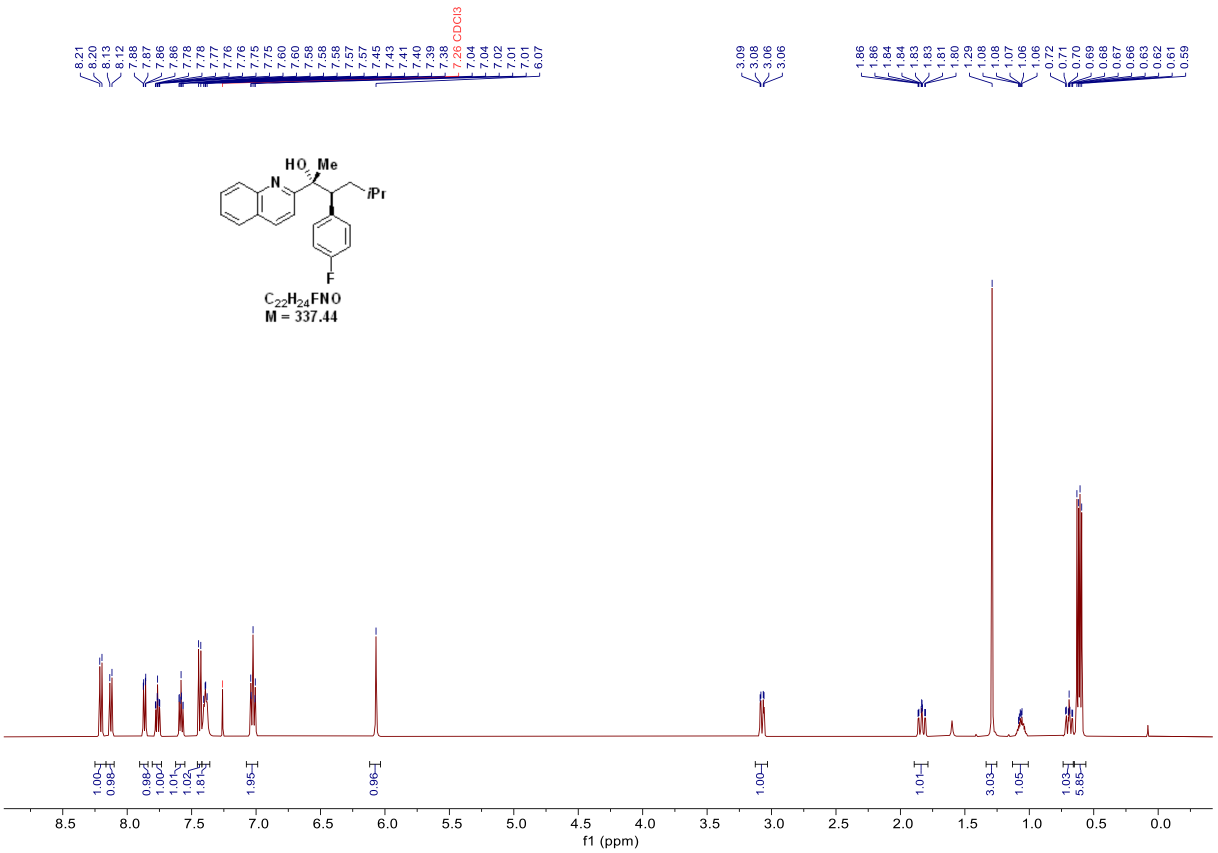

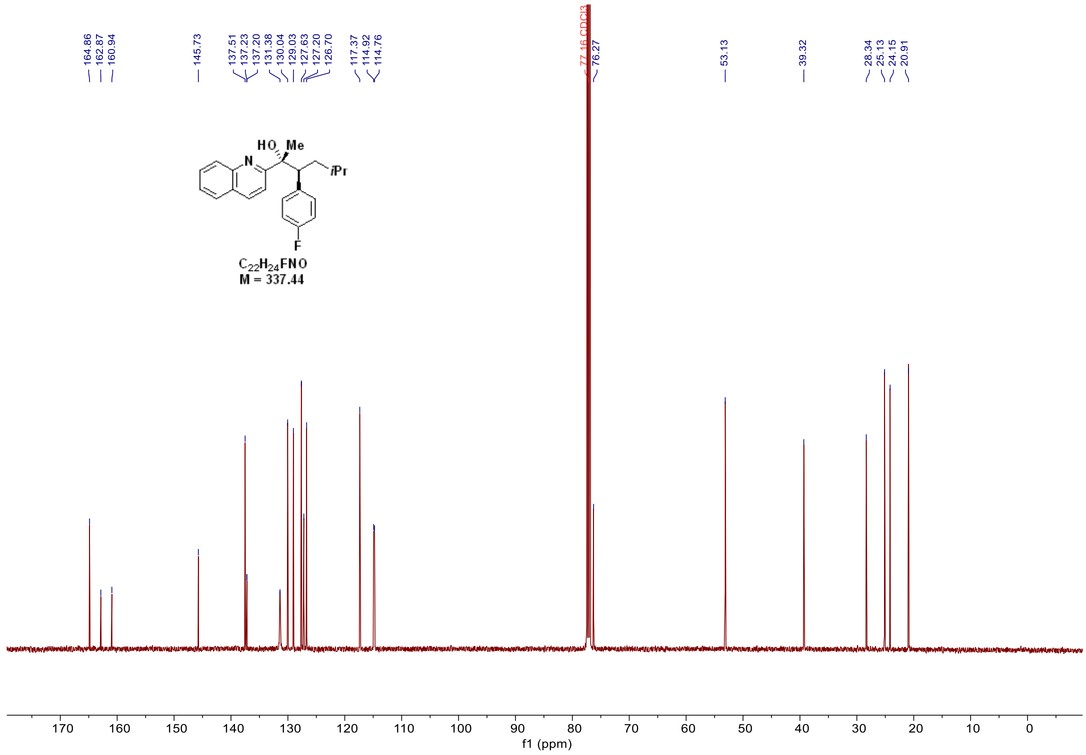

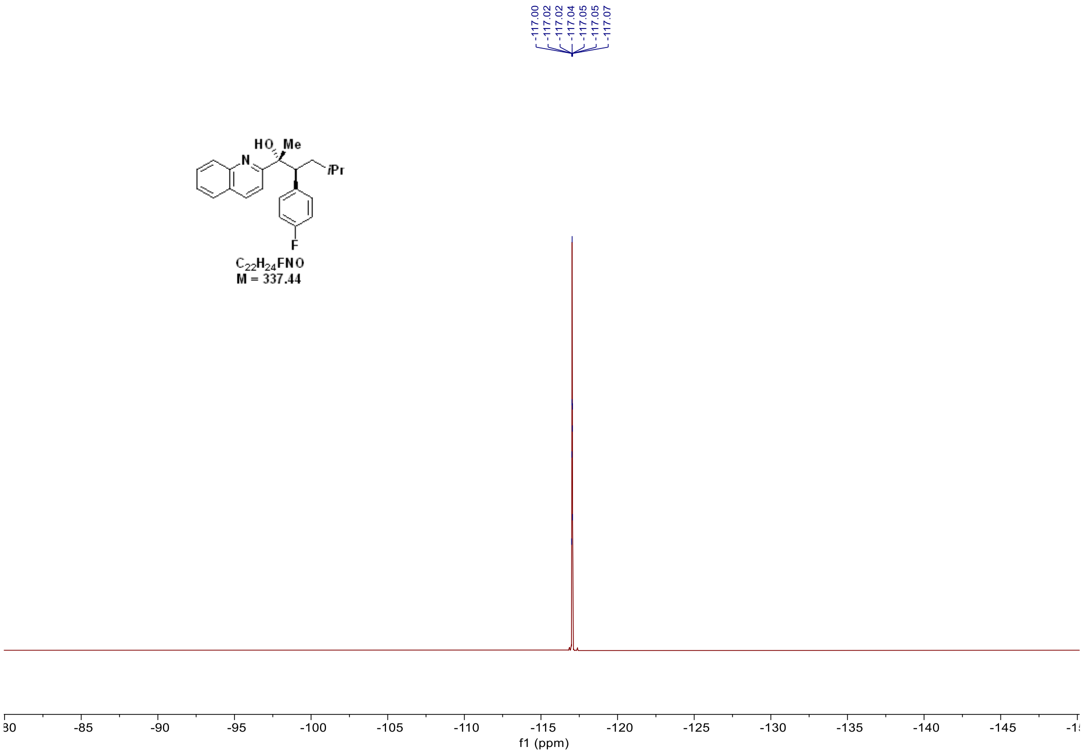

$C_{22}H_{24}FNO$
M = 337.44

^{1}H NMR (500 MHz, CD_3Cl, 298 K) and ^{13}C NMR (125 MHz, CD_3Cl, 298 K) of **3ak**.

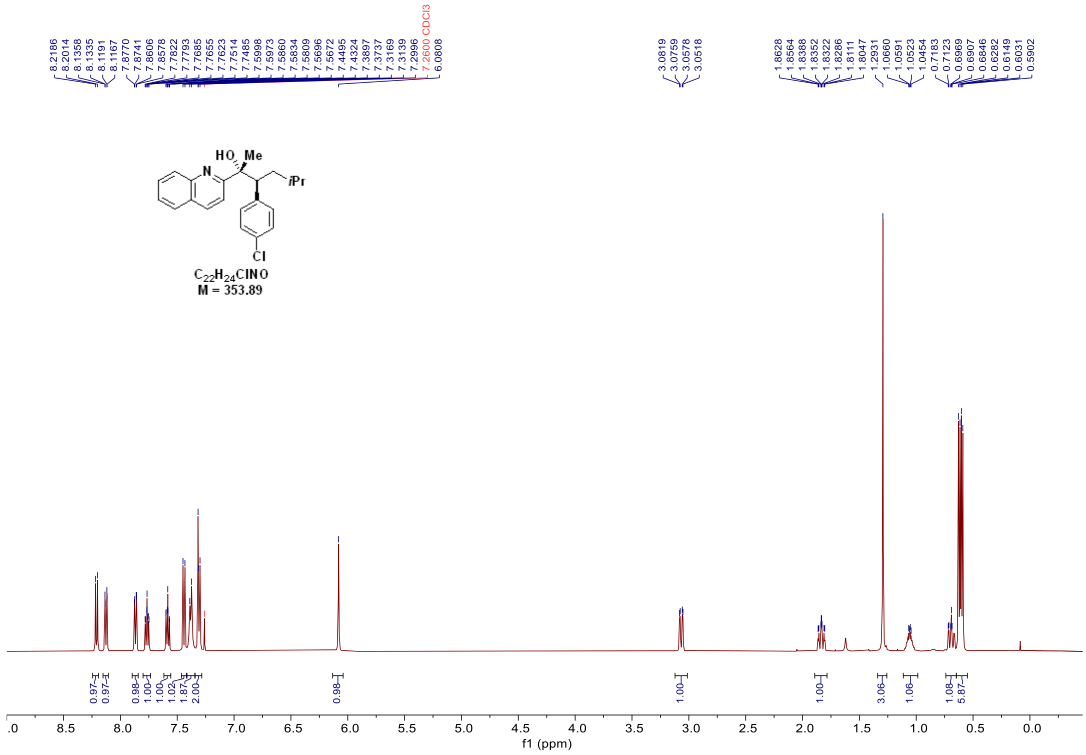

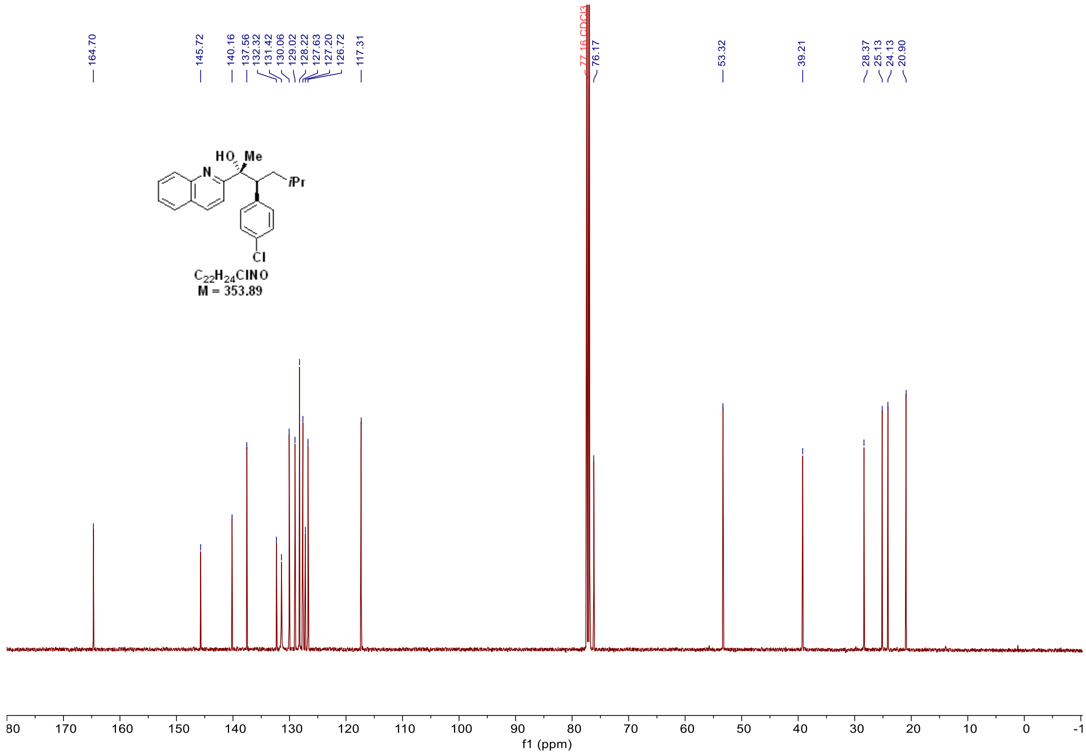

[1] H NMR (500 MHz, CD_3Cl, 298 K) and [13] C NMR (125 MHz, CD_3Cl, 298 K) of **3al**.

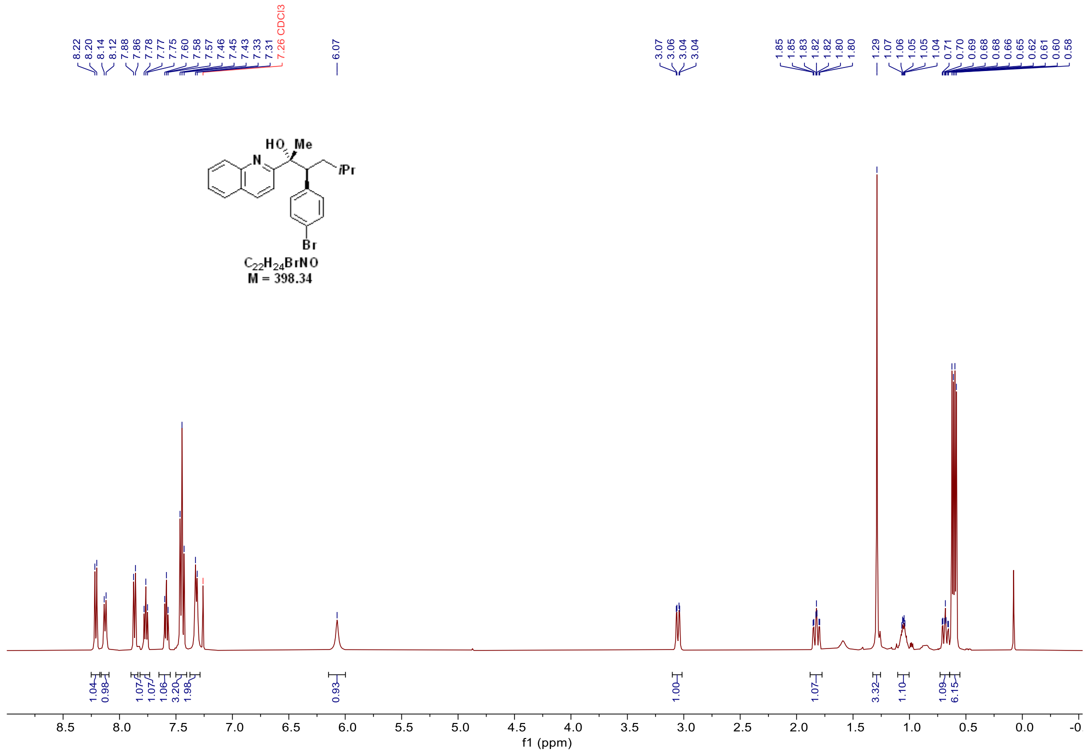

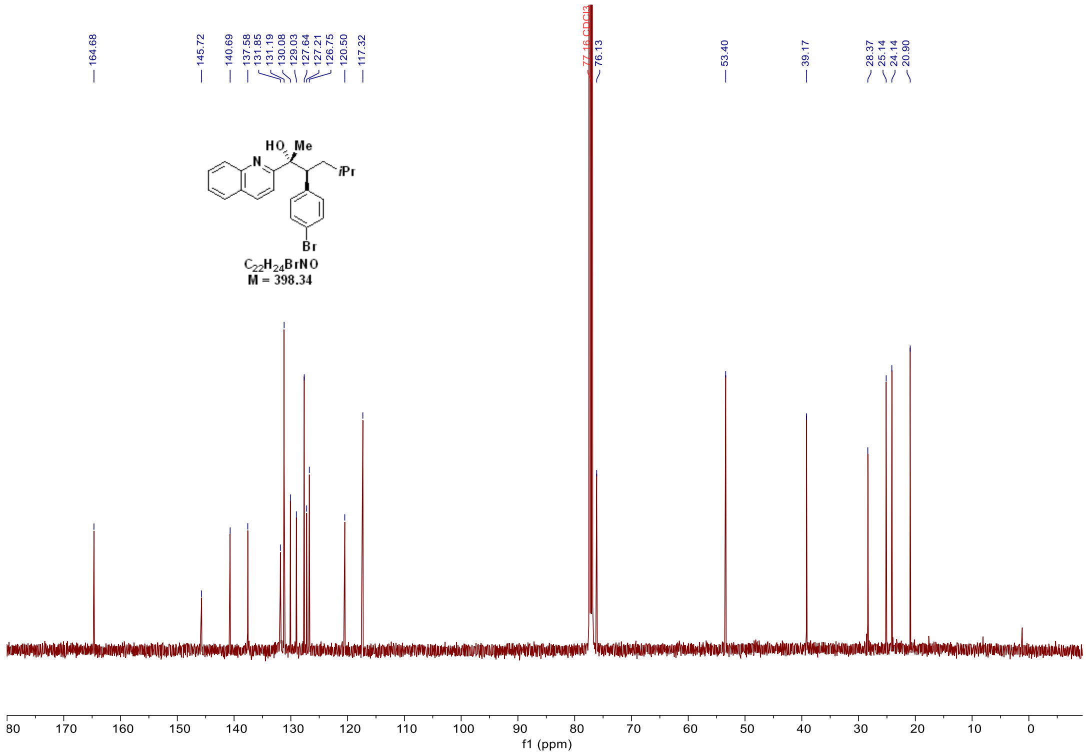

^{1}H NMR (500 MHz, CD_3Cl, 298 K), ^{13}C NMR (125 MHz, CD_3Cl, 298 K) and ^{19}F NMR (471 MHz, CD_3Cl, 298 K) of **3am**.

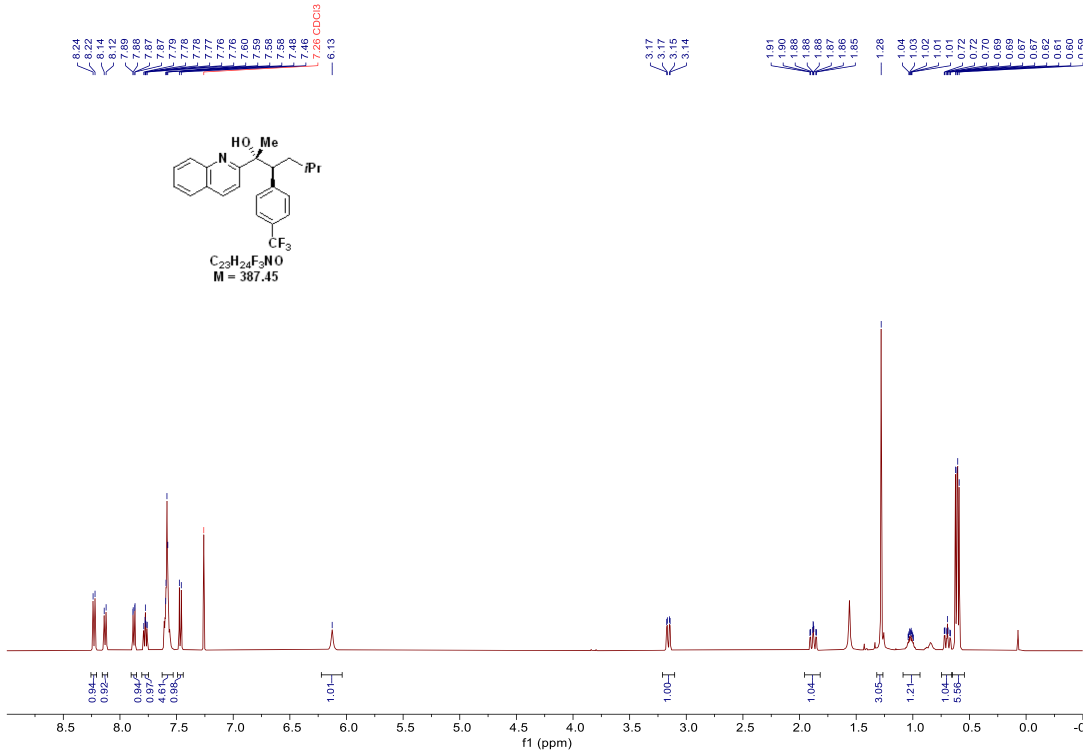

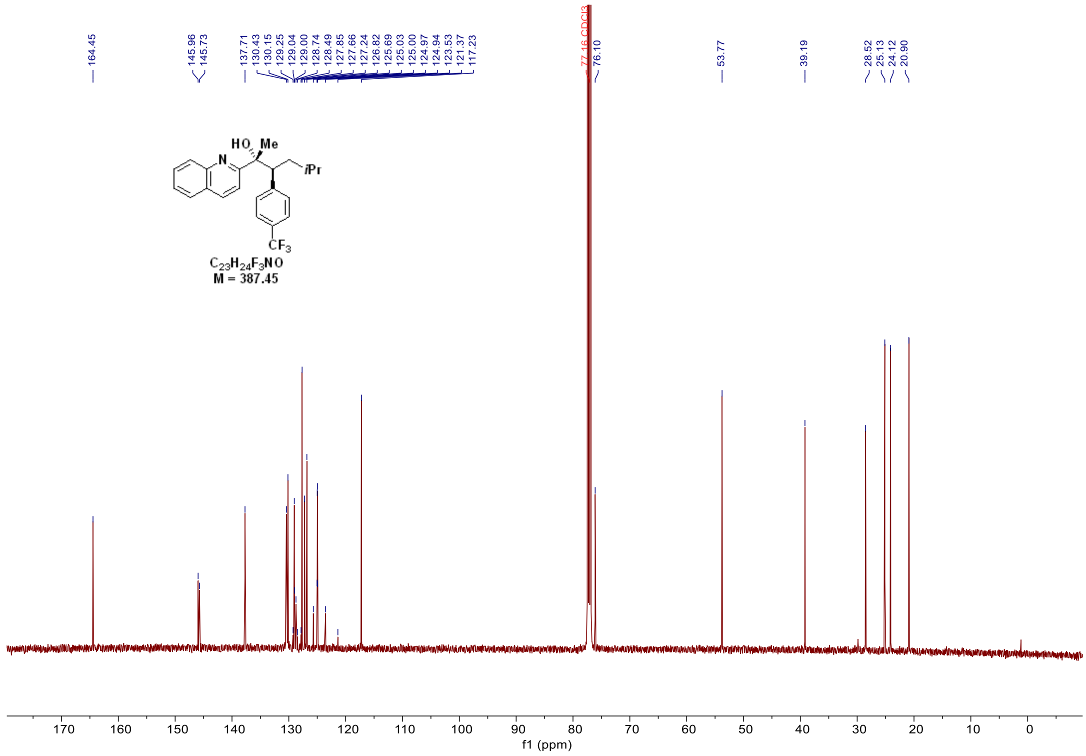

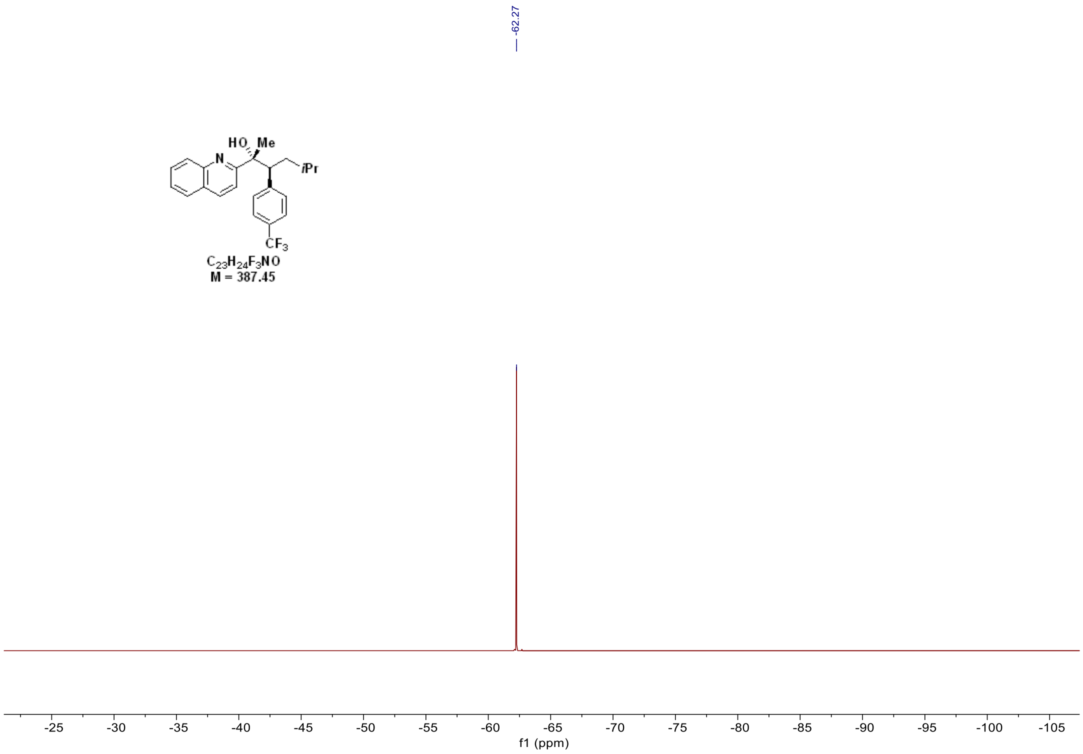
-62.27
HO Me
N
iPr
CF3
C23H24F3NO
M = 387.45
-25
-30
-35
-40
-45
-50
-55
-60
-65
-70
-75
-80
-85
-90
-95
-100
-105
f1 (ppm)

^{1}H NMR (400 MHz, CD_3Cl, 298 K) and ^{13}C NMR (100 MHz, CD_3Cl, 298 K) of **3an**.

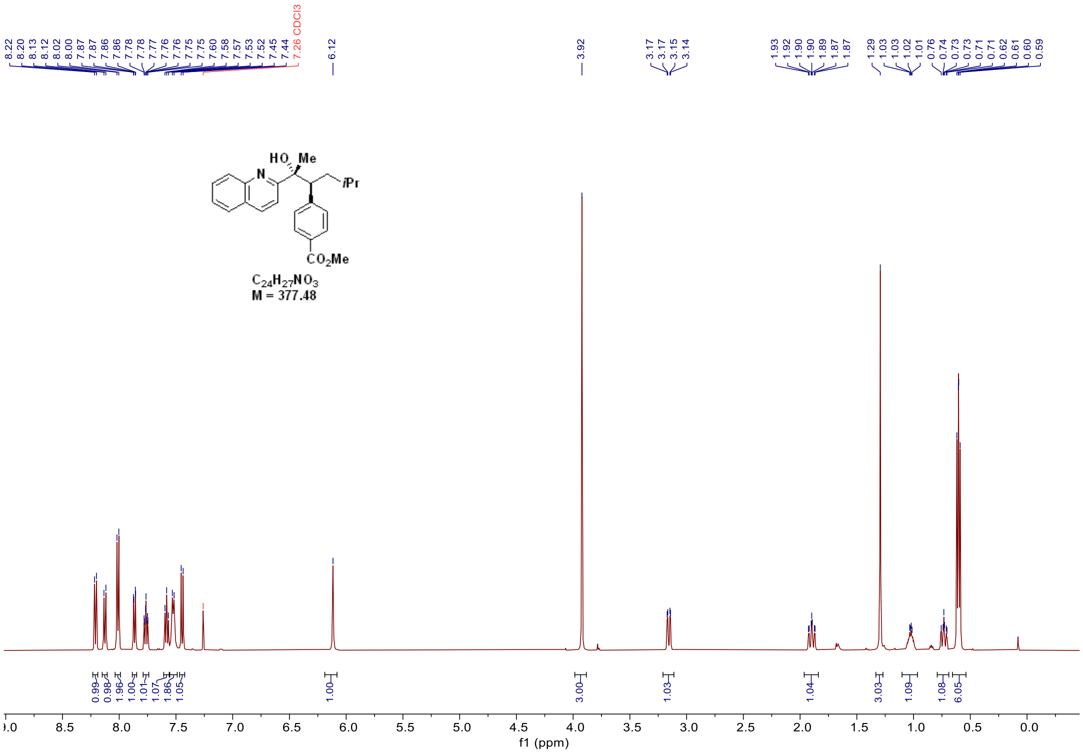

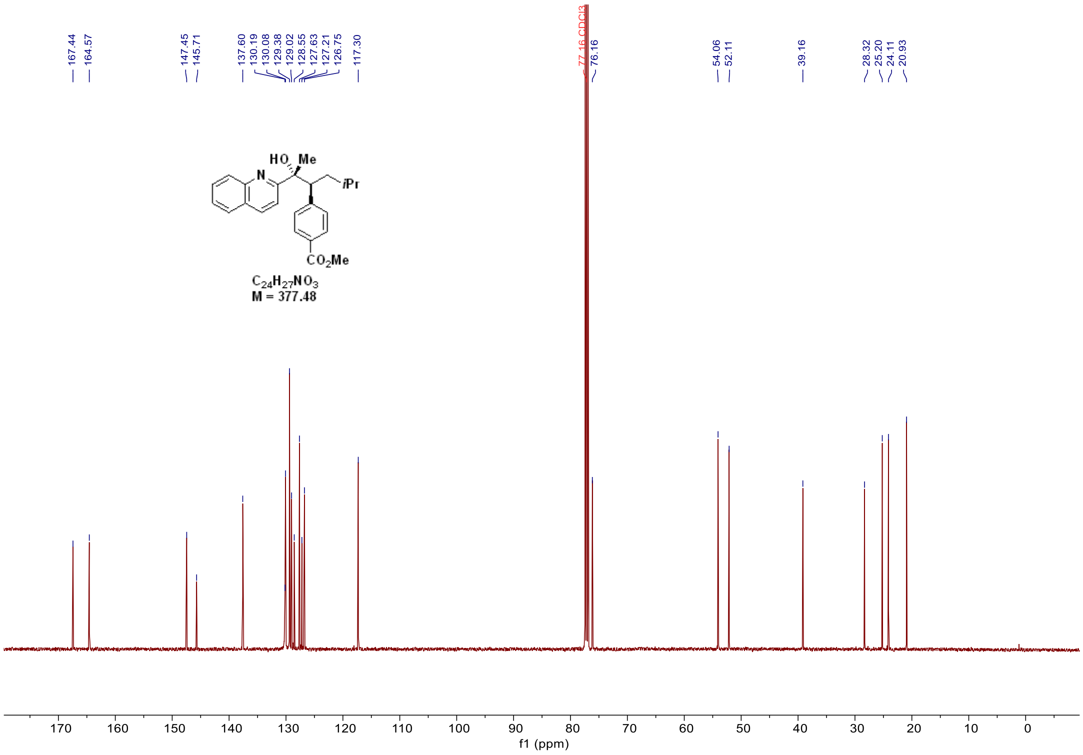

^{1}H NMR (400 MHz, CD_3Cl, 298 K) and ^{13}C NMR (100 MHz, CD_3Cl, 298 K) of **3ao**.

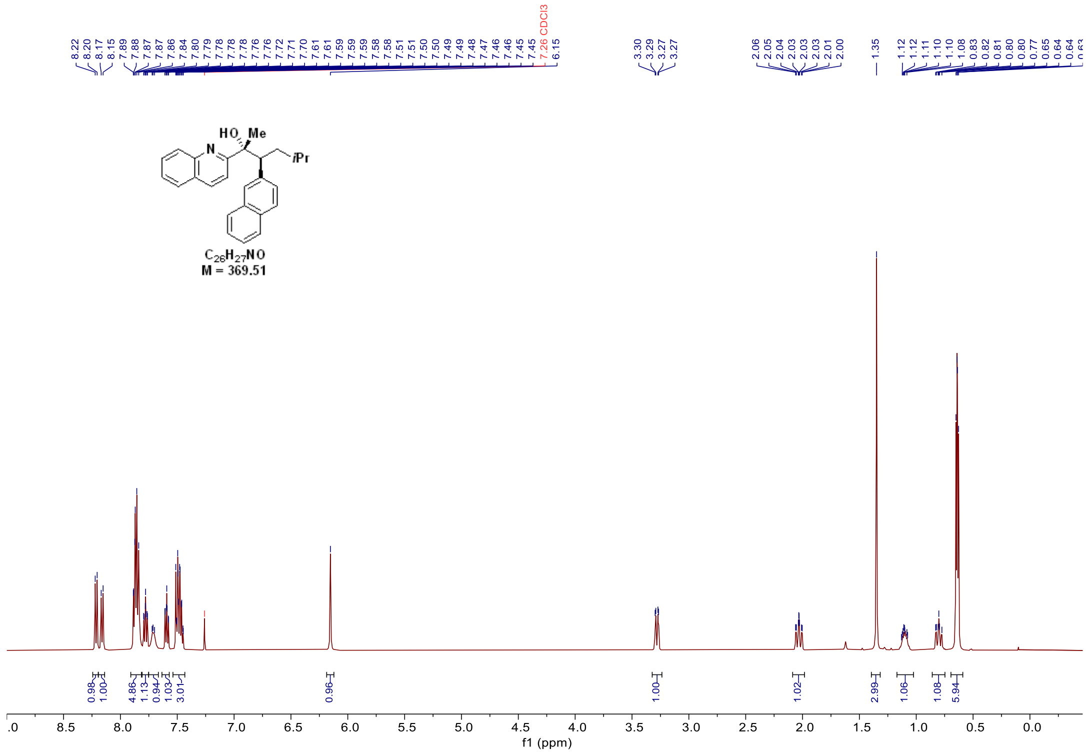

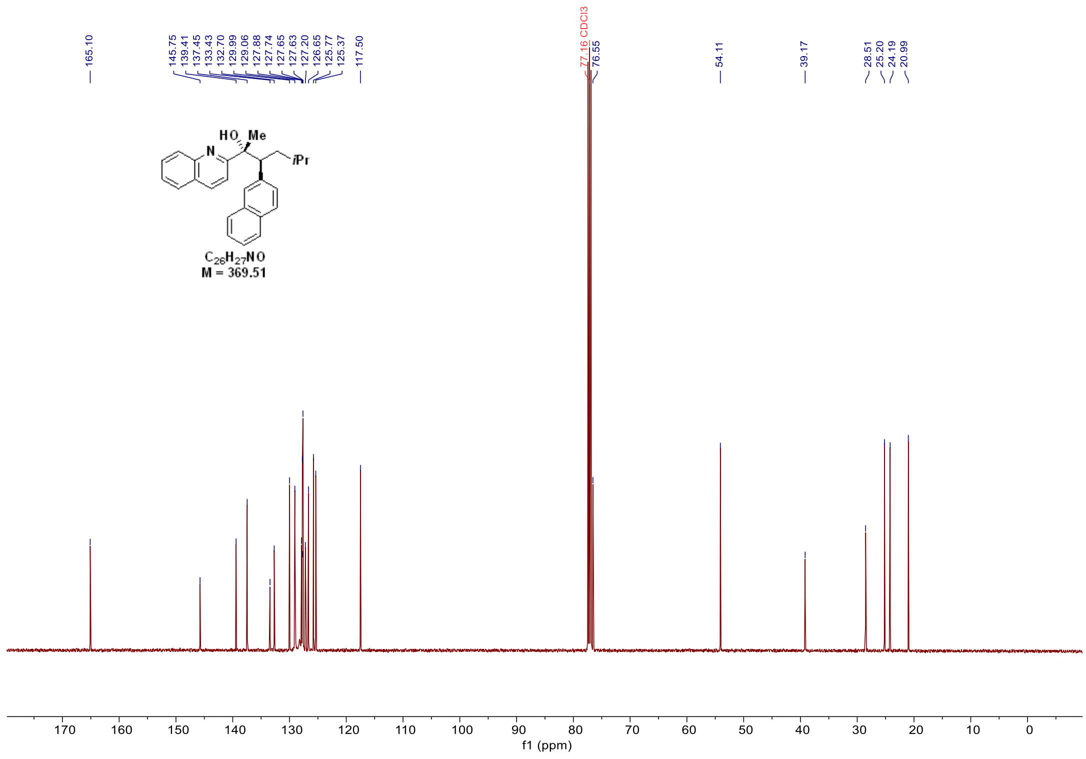

^{1}H NMR (400 MHz, CD_3Cl, 298 K) and ^{13}C NMR (100 MHz, CD_3Cl, 298 K) of ***ent*-3aa'**.

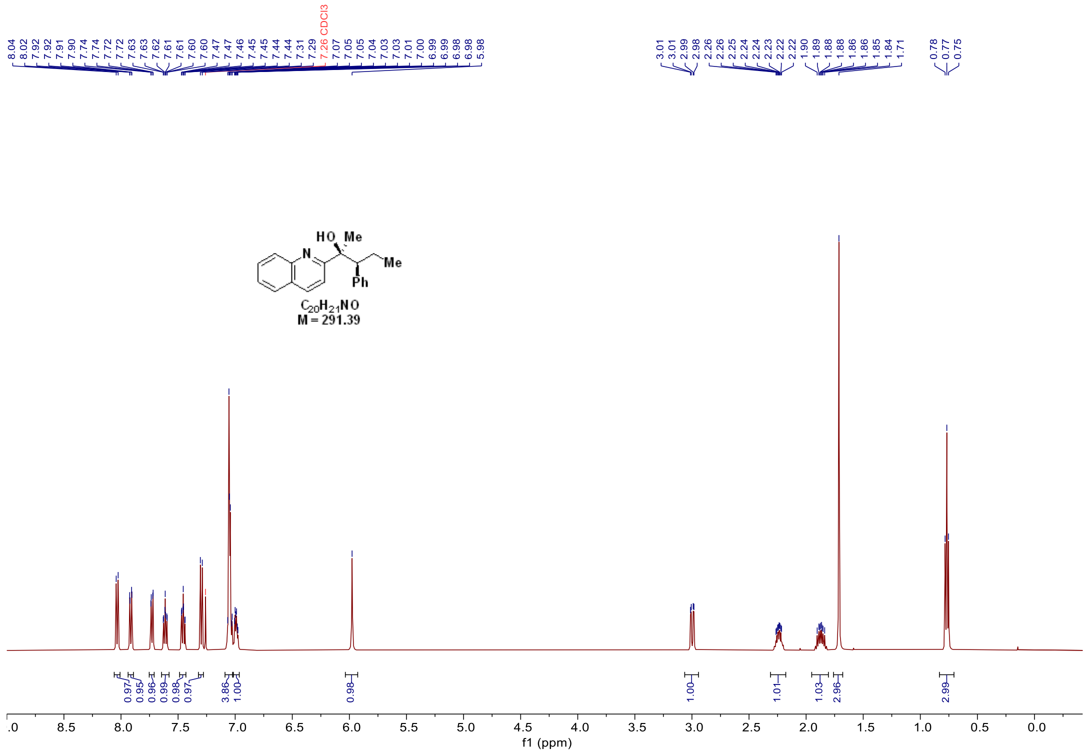

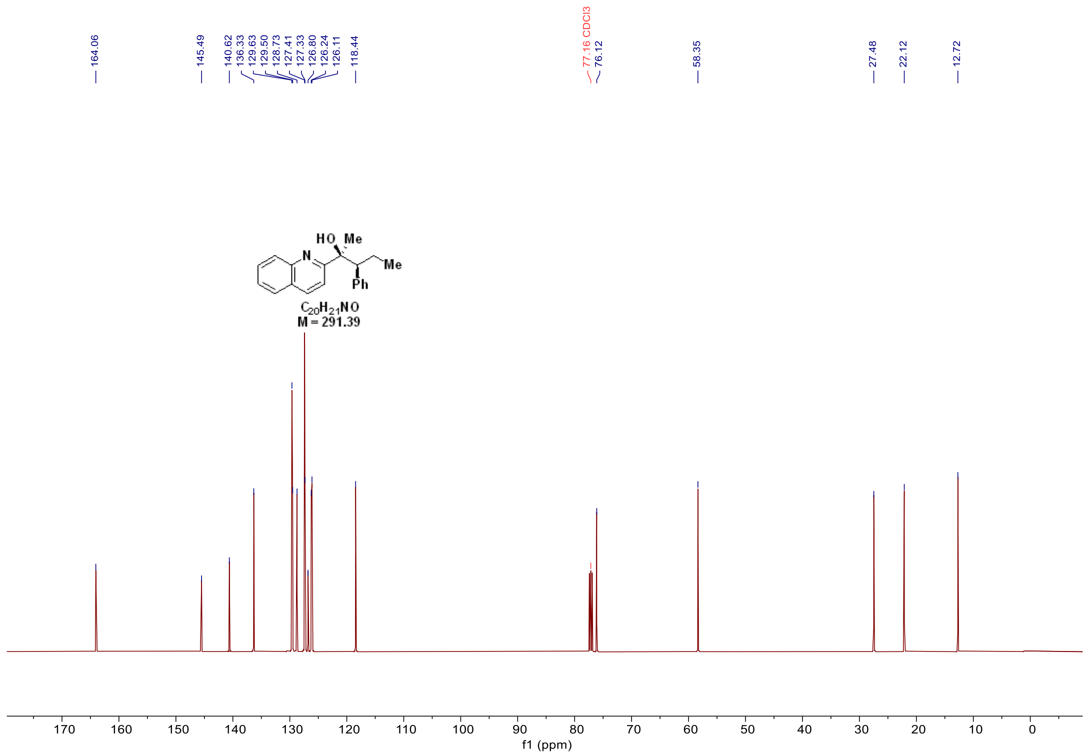

^{1}H NMR (500 MHz, CD_3Cl, 298 K) and ^{13}C NMR (125 MHz, CD_3Cl, 298 K) of **3ap**.

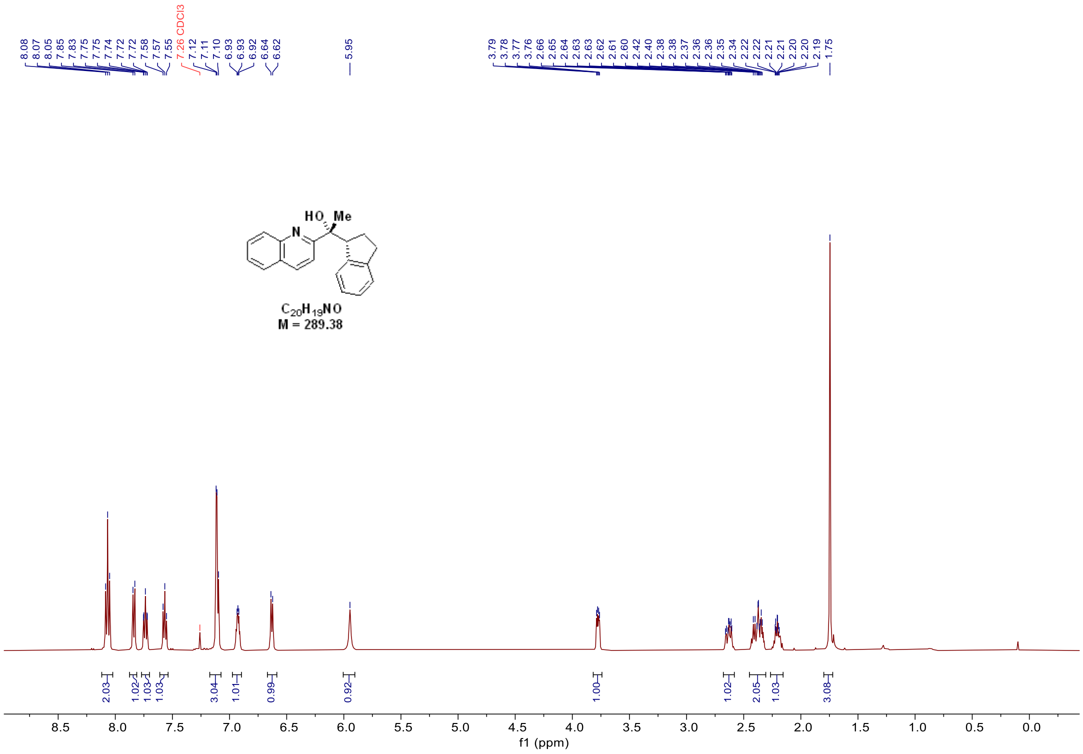

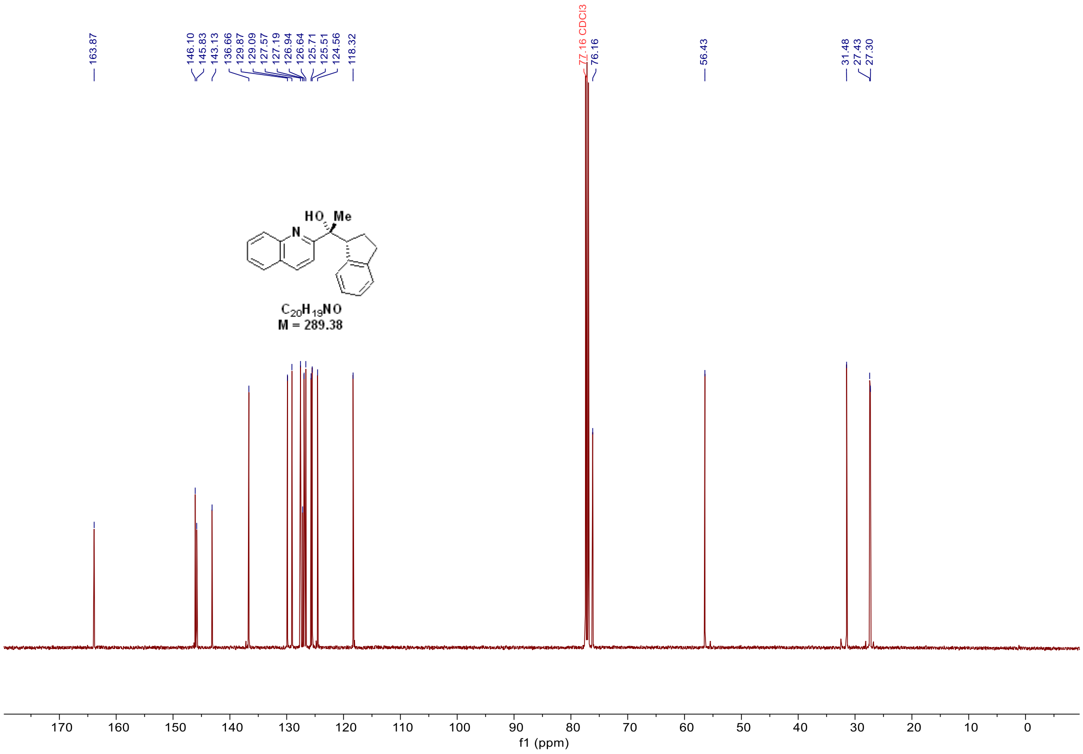

^{1}H NMR (500 MHz, CD_3Cl, 298 K) and ^{13}C NMR (125 MHz, CD_3Cl, 298 K) of **3aq**.

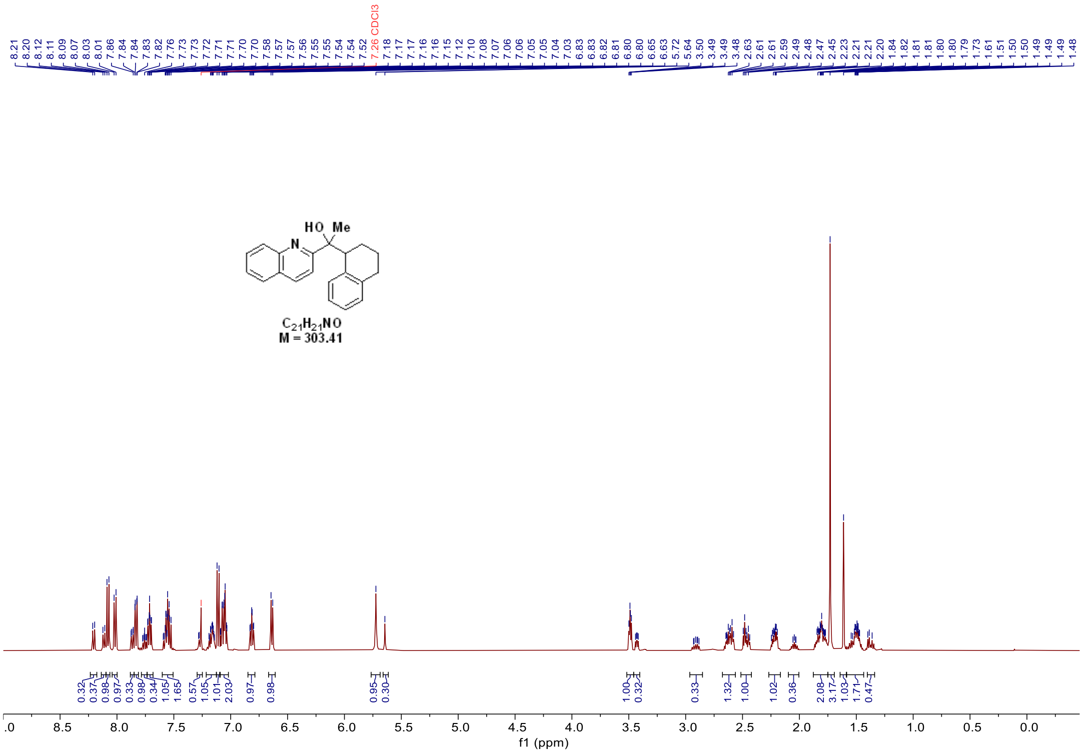

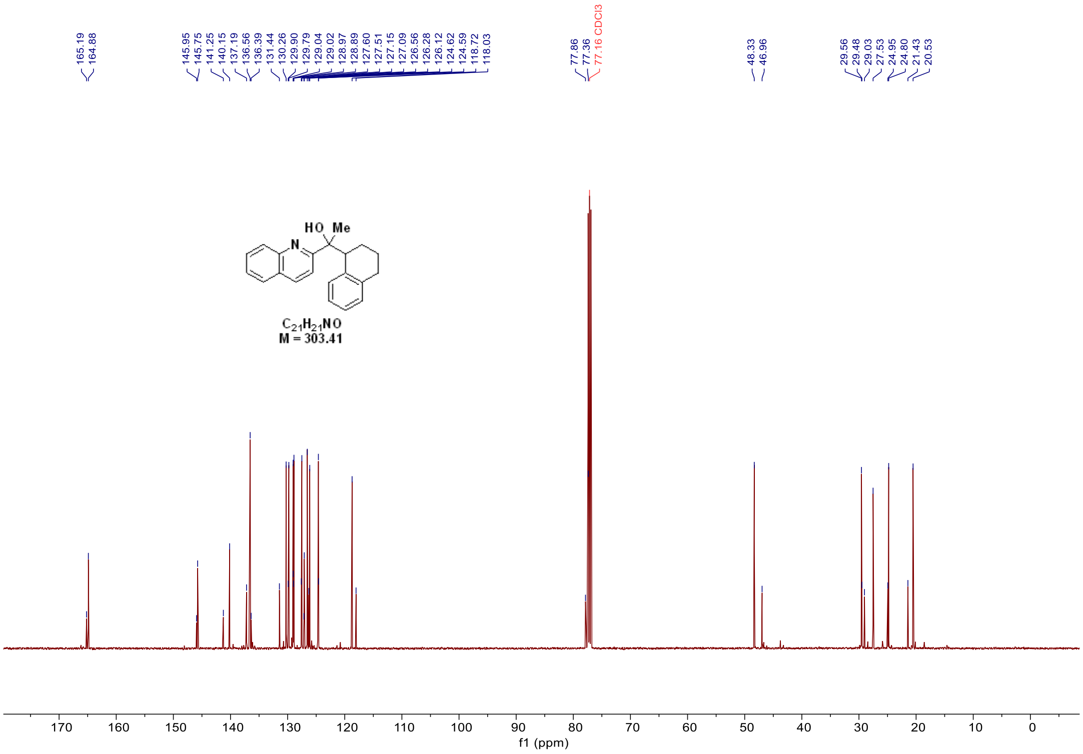

^{1}H NMR (500 MHz, CD_3Cl, 298 K), ^{13}C NMR (125 MHz, CD_3Cl, 298 K) and HMBC of **3ar**.

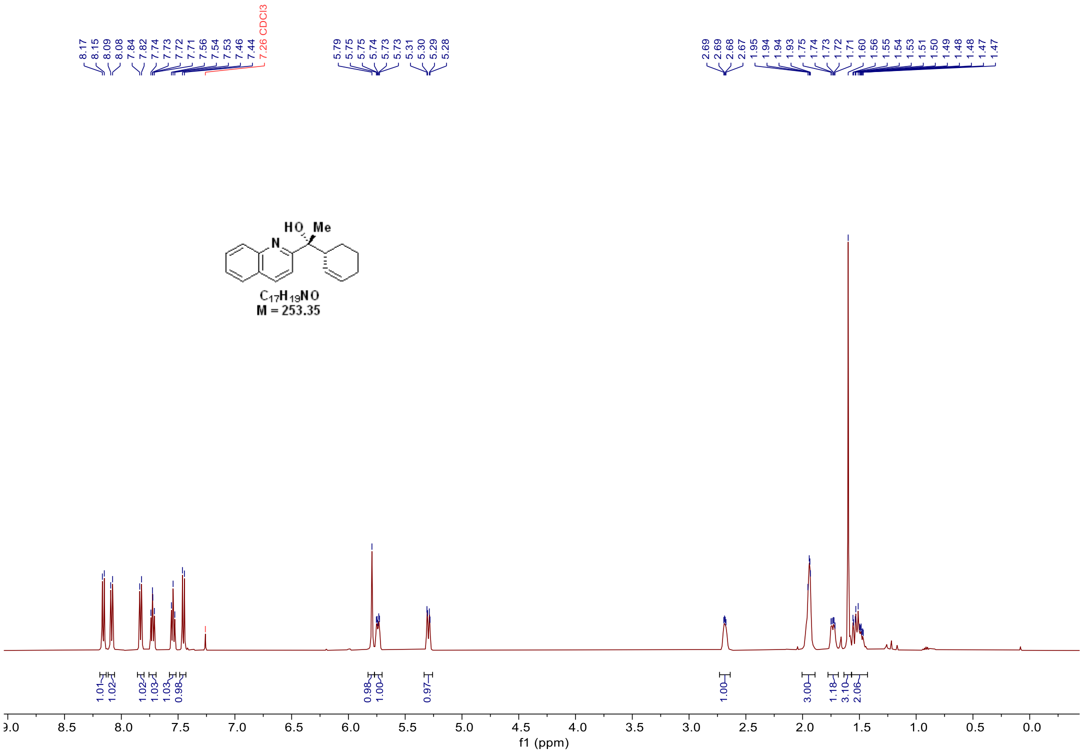

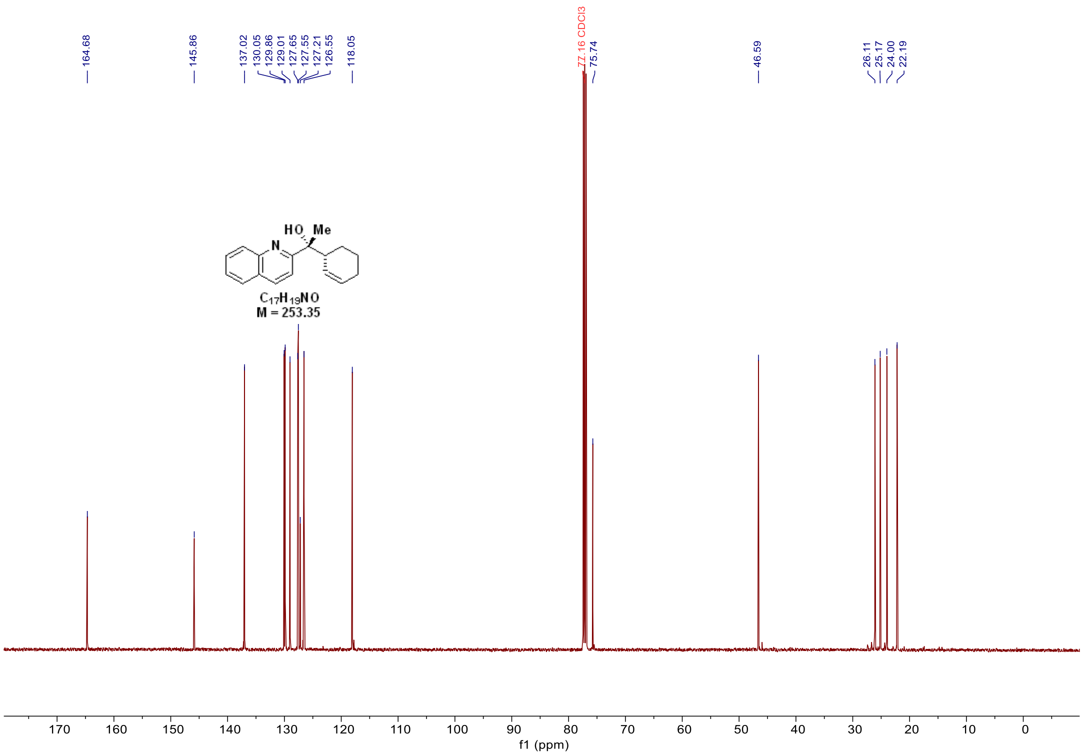

^{1}H NMR (500 MHz, CD_3Cl, 298 K) and ^{13}C NMR (125 MHz, CD_3Cl, 298 K) of **3bp**.

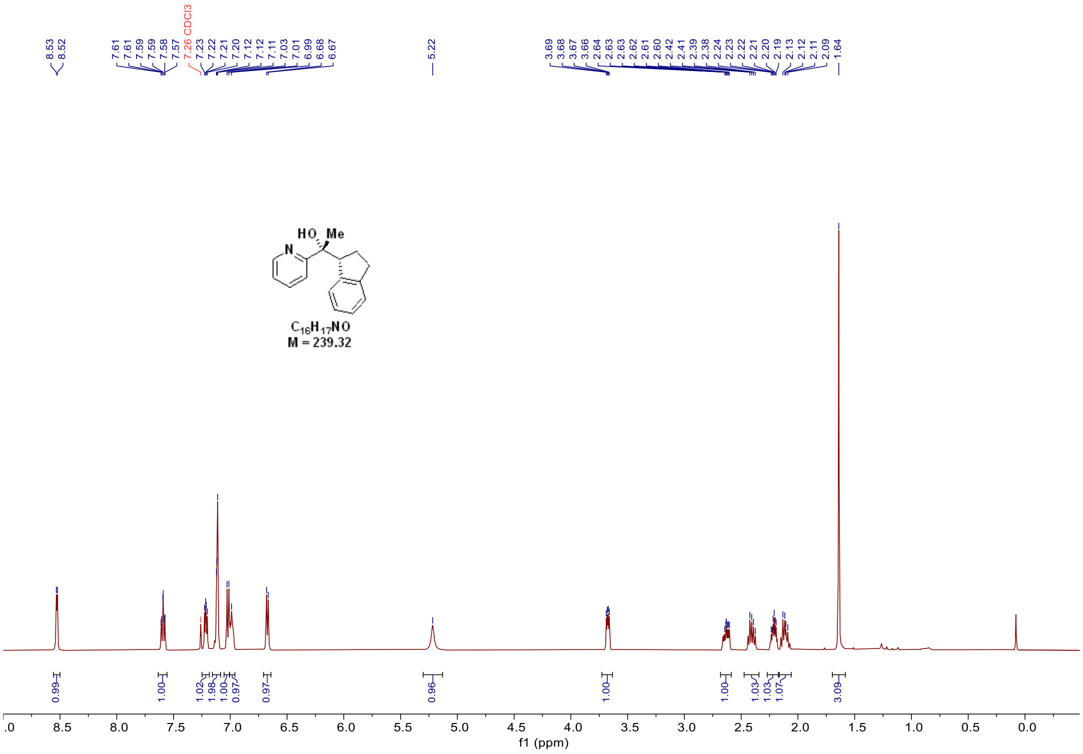

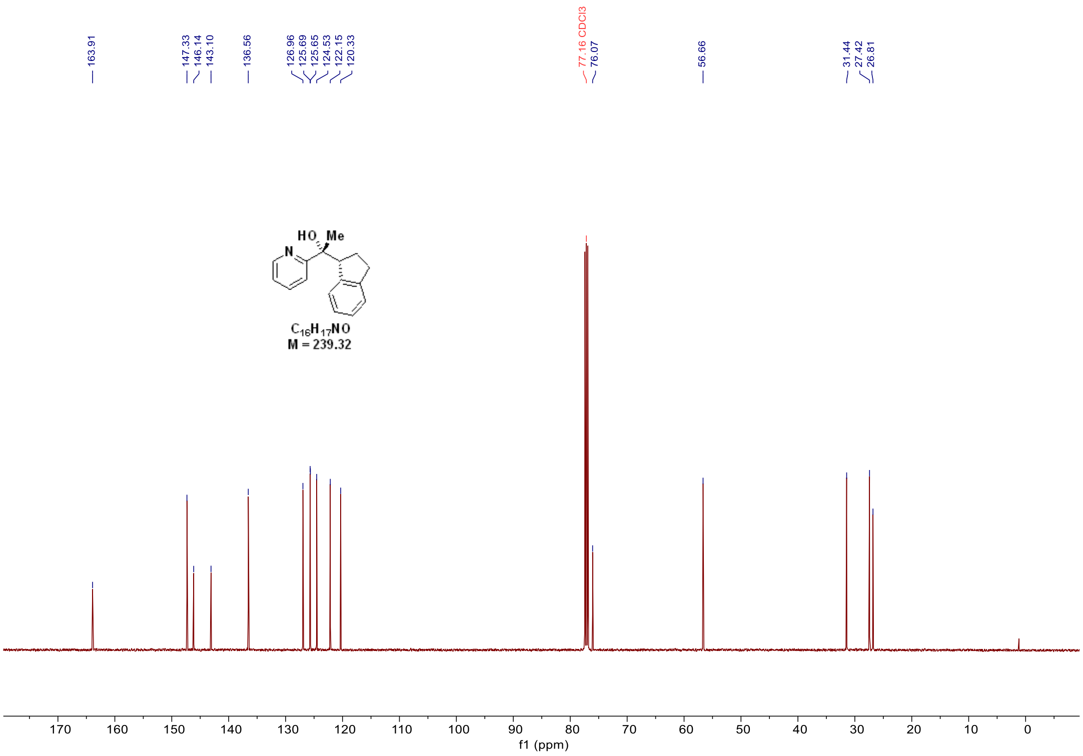

^{1}H NMR (500 MHz, CD_3Cl, 298 K) and ^{13}C NMR (125 MHz, CD_3Cl, 298 K) of **3cp**.

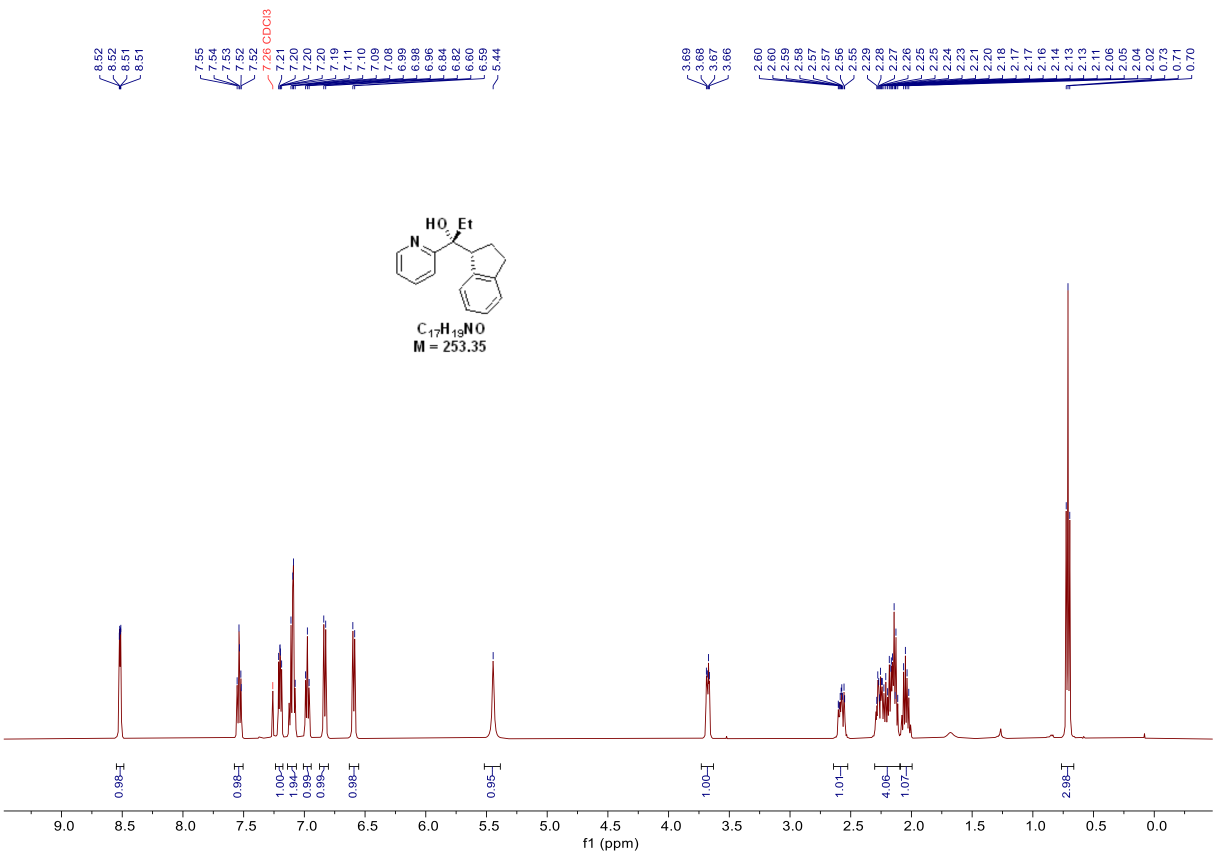

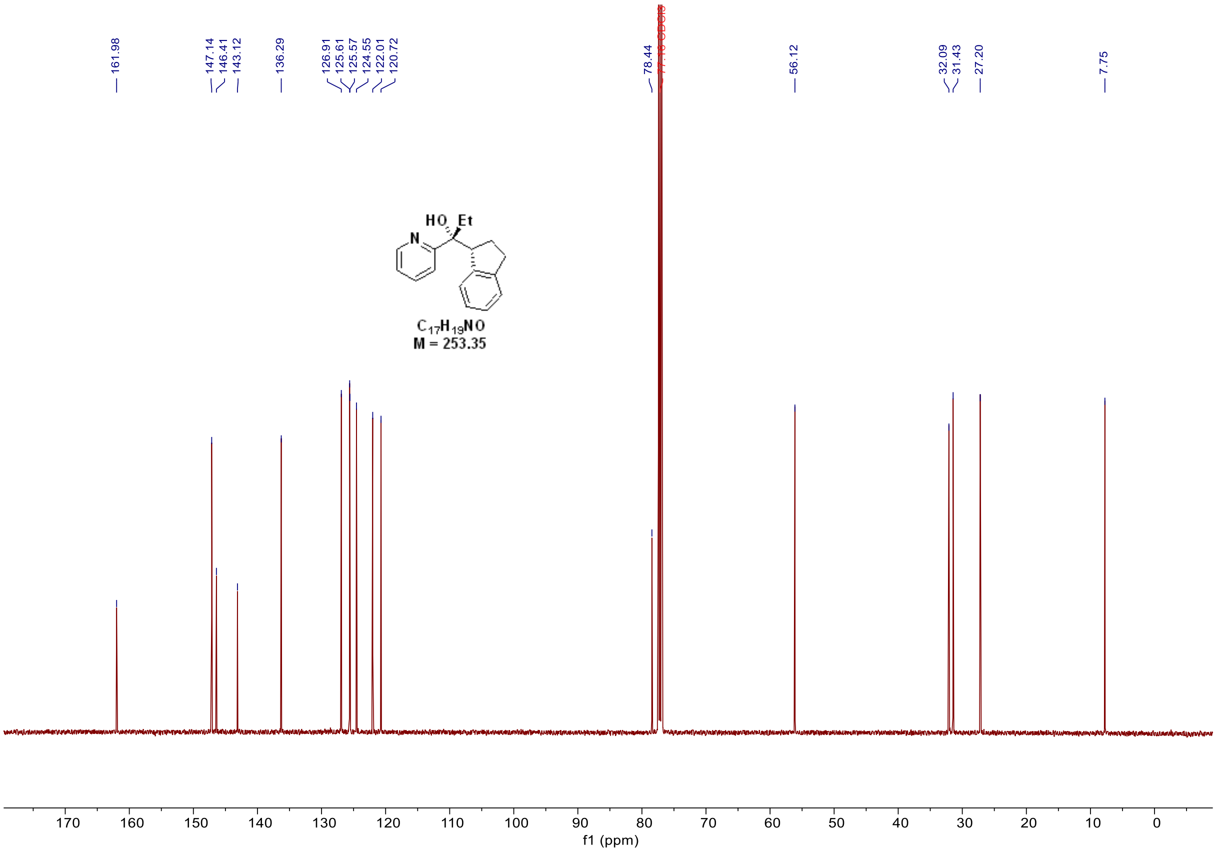

^{1}H NMR (400 MHz, CD_3Cl, 298 K) and ^{13}C NMR (100 MHz, CD_3Cl, 298 K) of **3dp**.

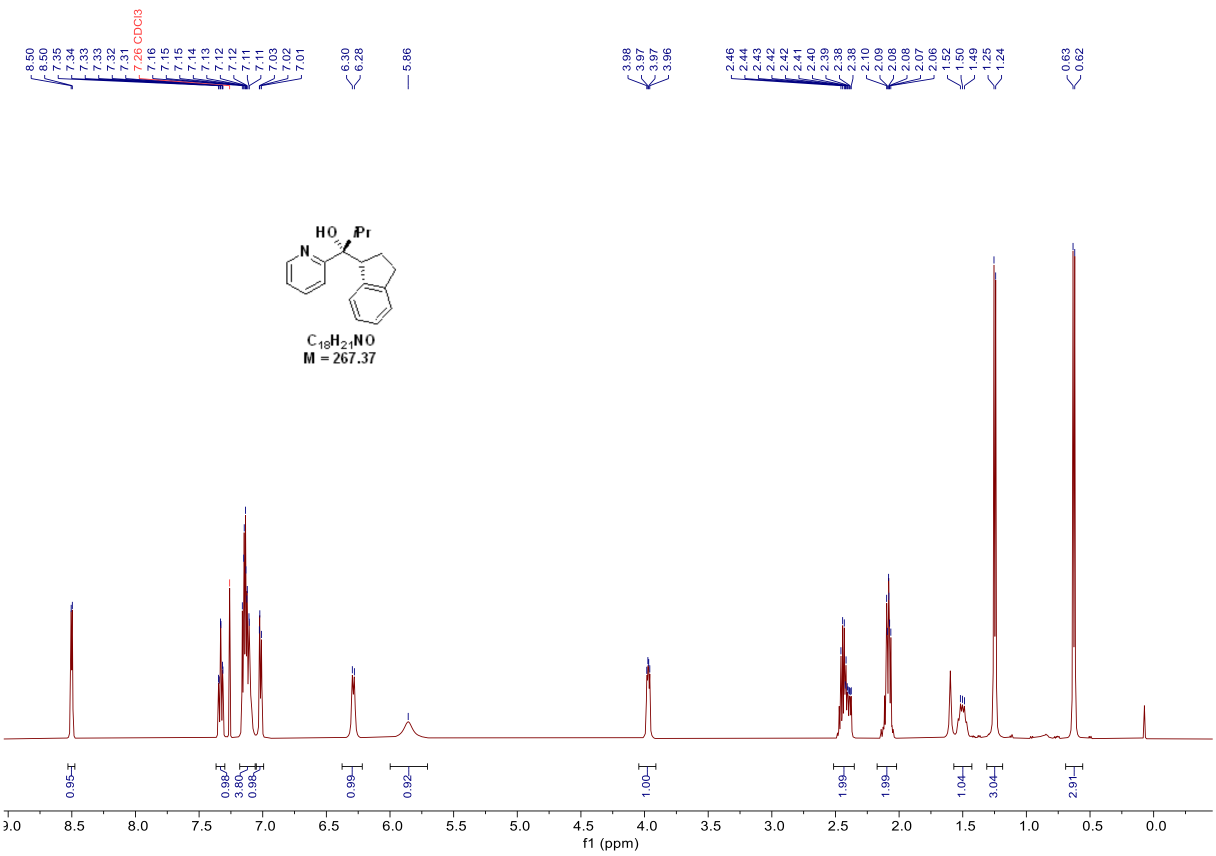

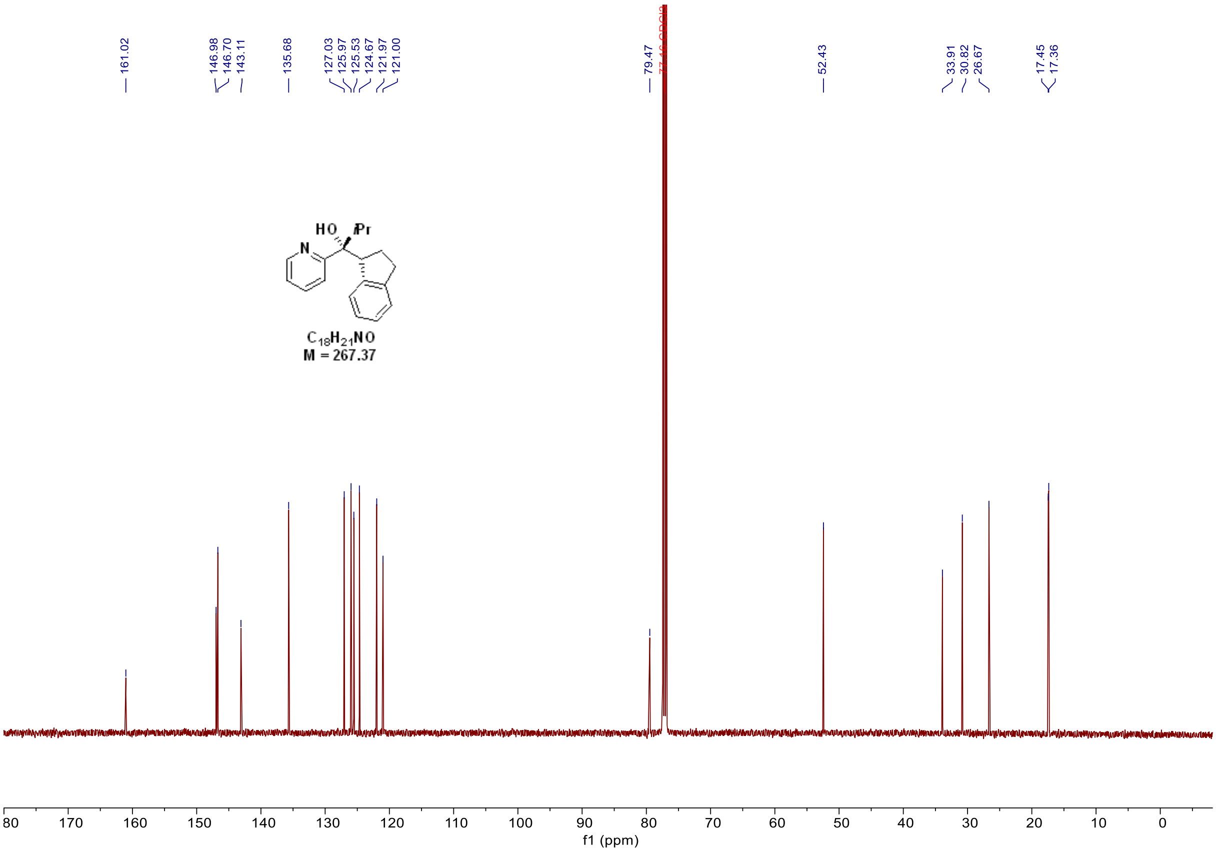

^{1}H NMR (400 MHz, CD_3Cl, 298 K) and ^{13}C NMR (100 MHz, CD_3Cl, 298 K) of **3ep**.

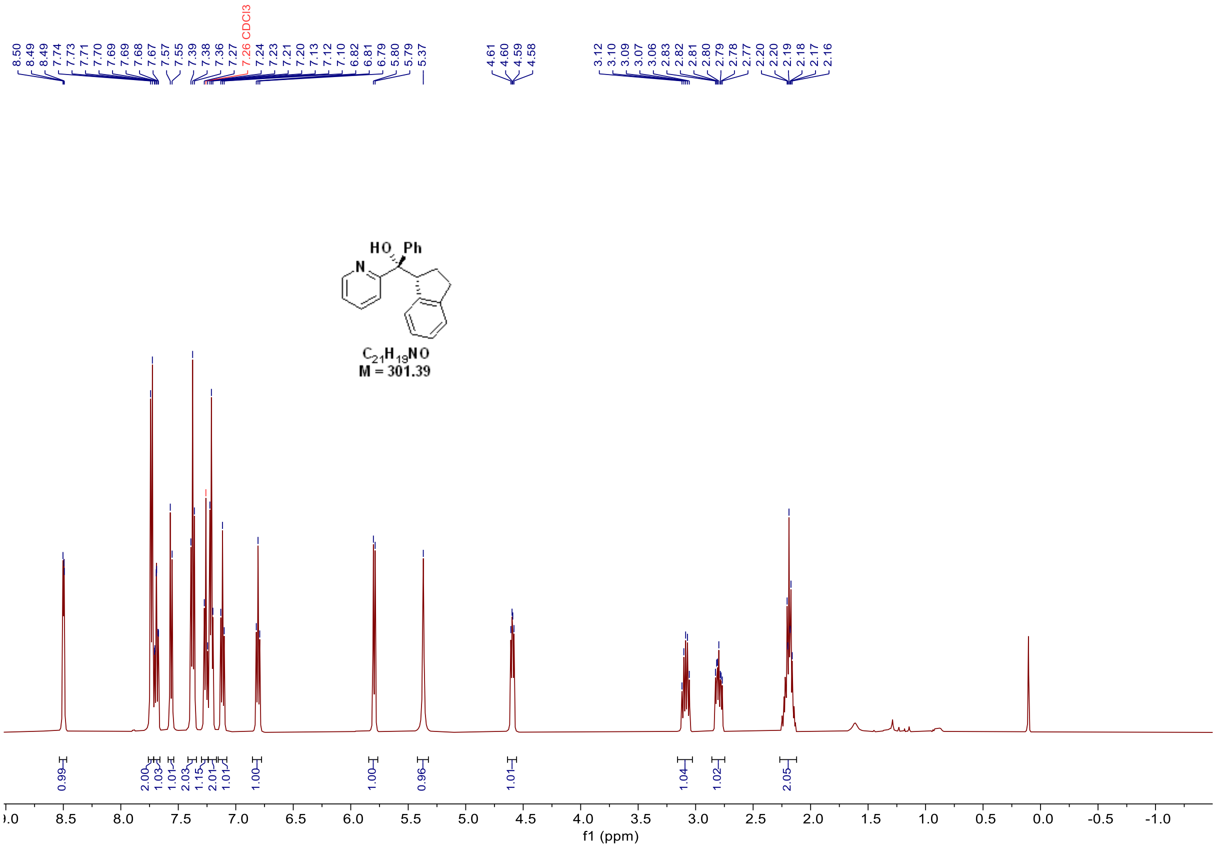

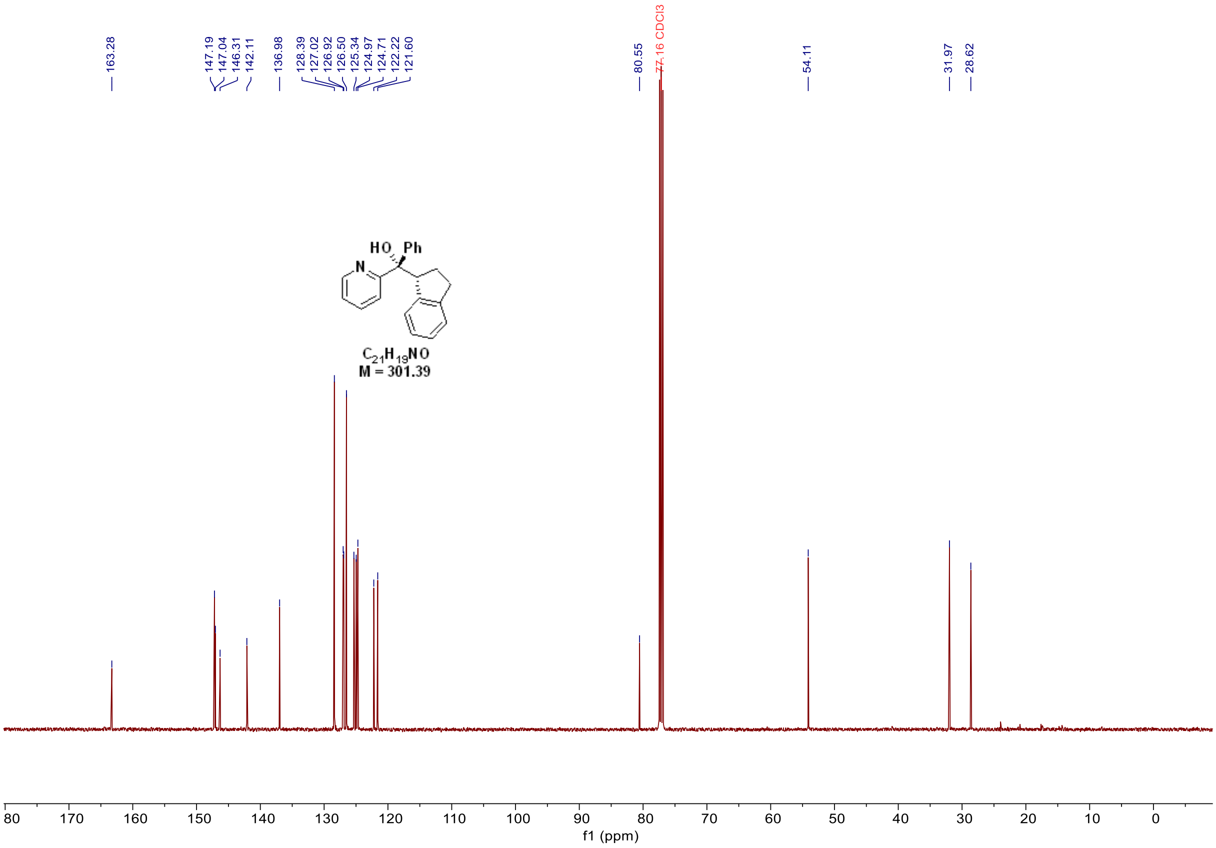

^{1}H NMR (500 MHz, CD_3Cl, 298 K) and ^{13}C NMR (125 MHz, CD_3Cl, 298 K) of **3fp**.

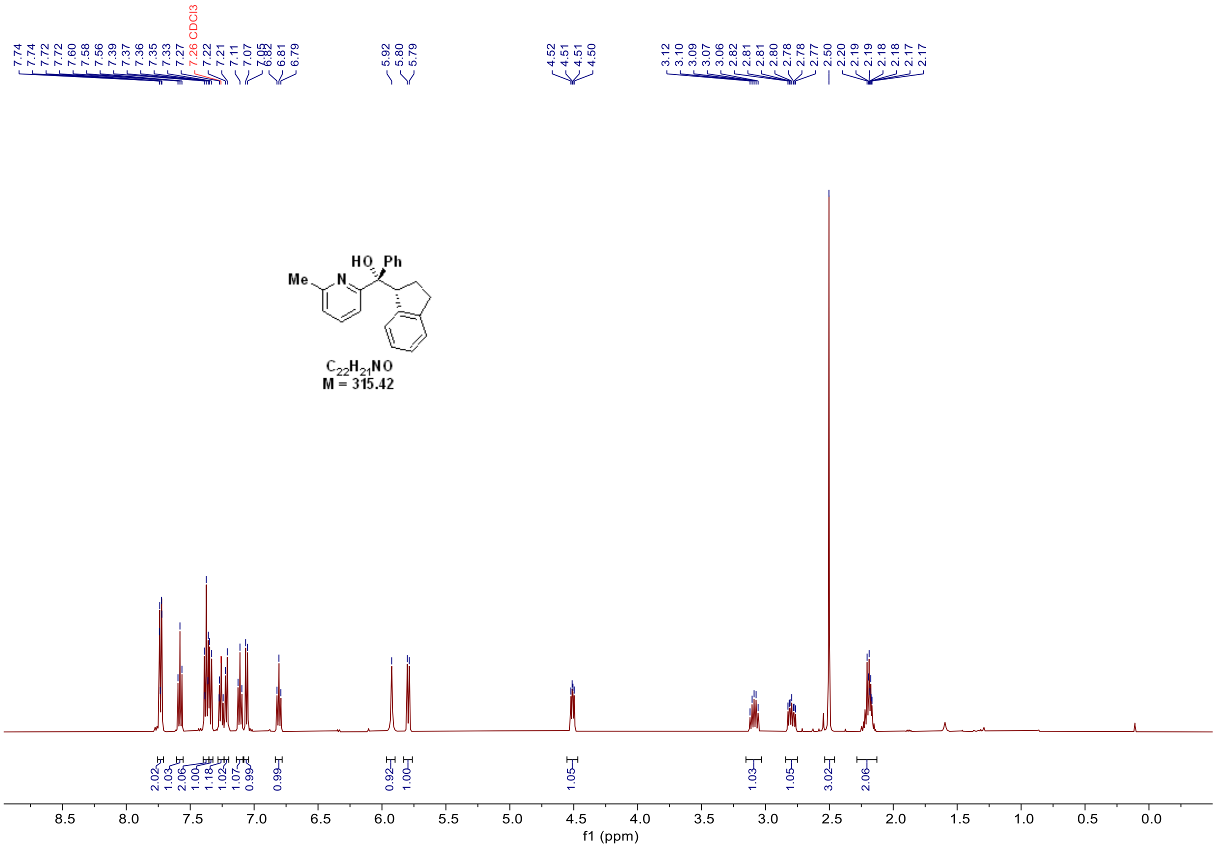

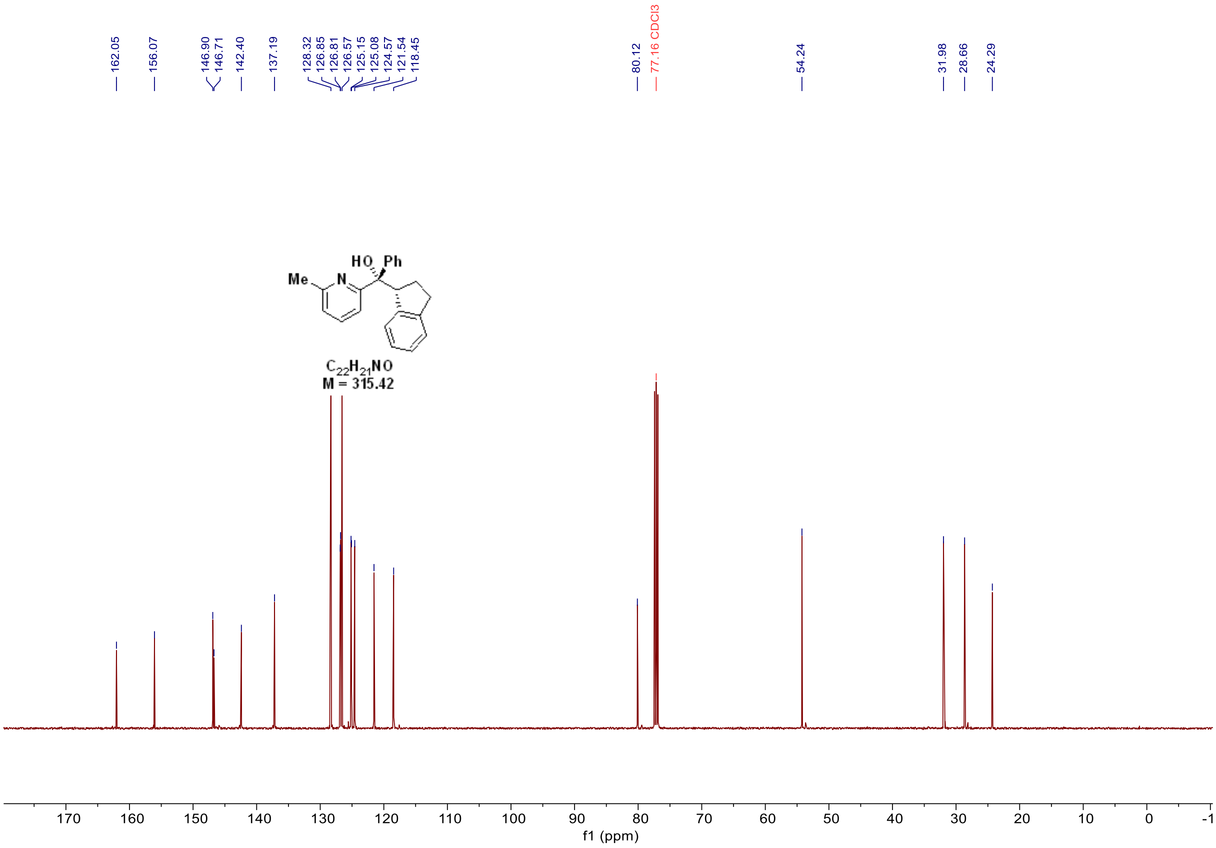

^{1}H NMR (500 MHz, CD_3Cl, 298 K) and ^{13}C NMR (125 MHz, CD_3Cl, 298 K) of **3gp**.

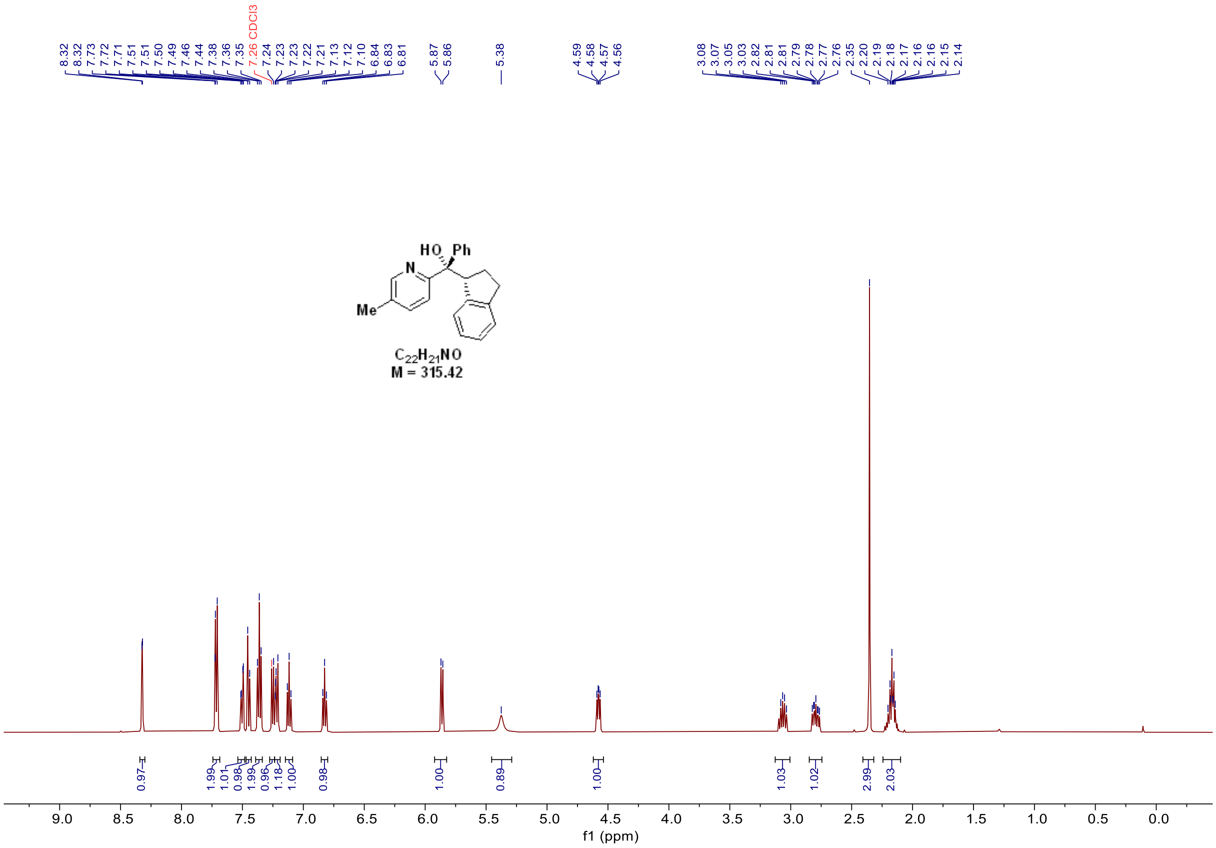

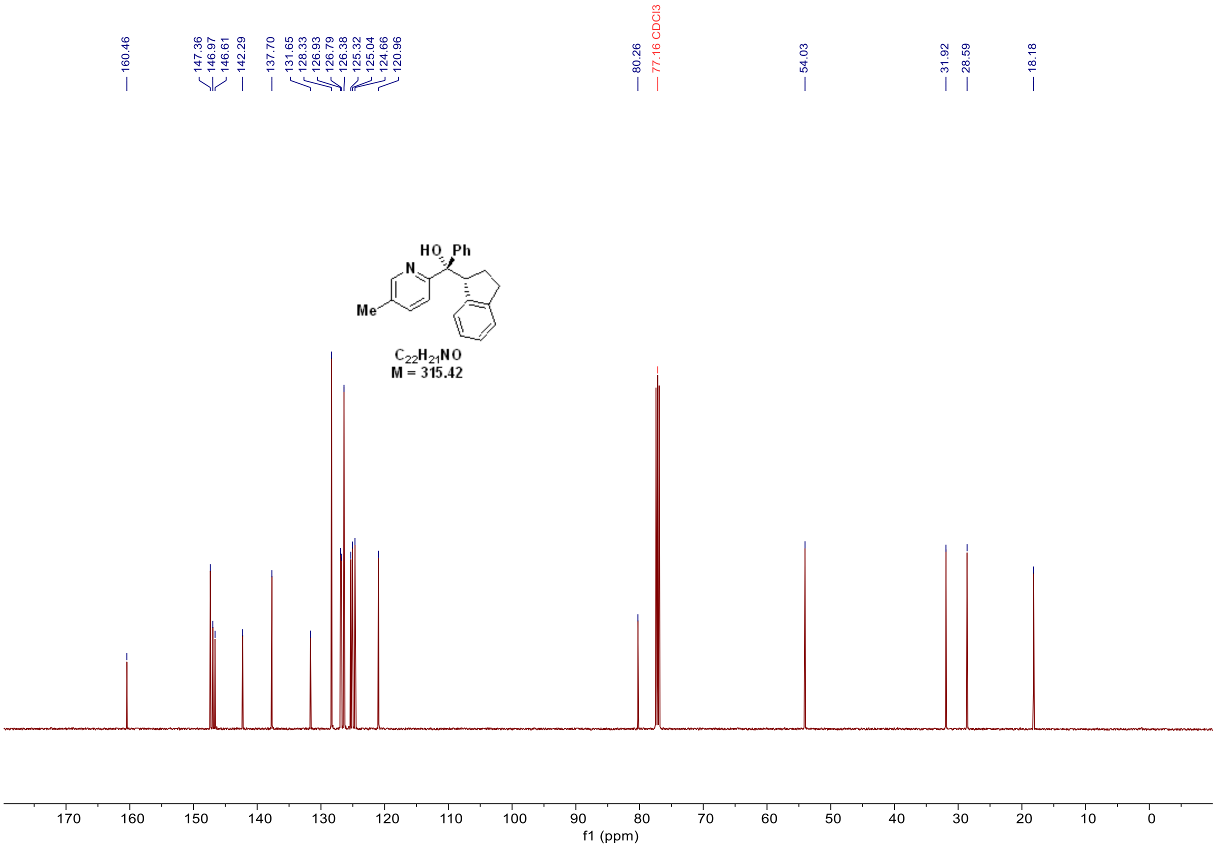

^{1}H NMR (500 MHz, CD_3Cl, 298 K) and ^{13}C NMR (125 MHz, CD_3Cl, 298 K) of **3hp**.

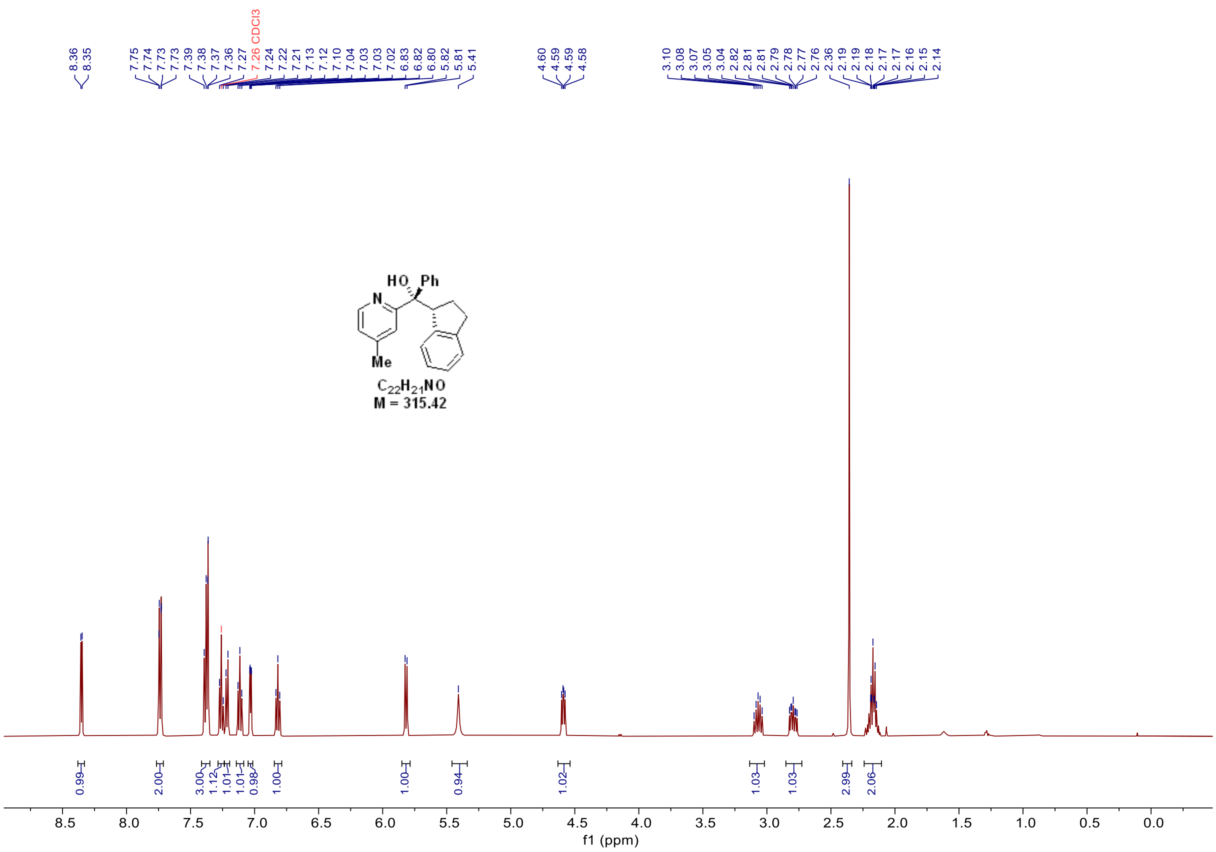

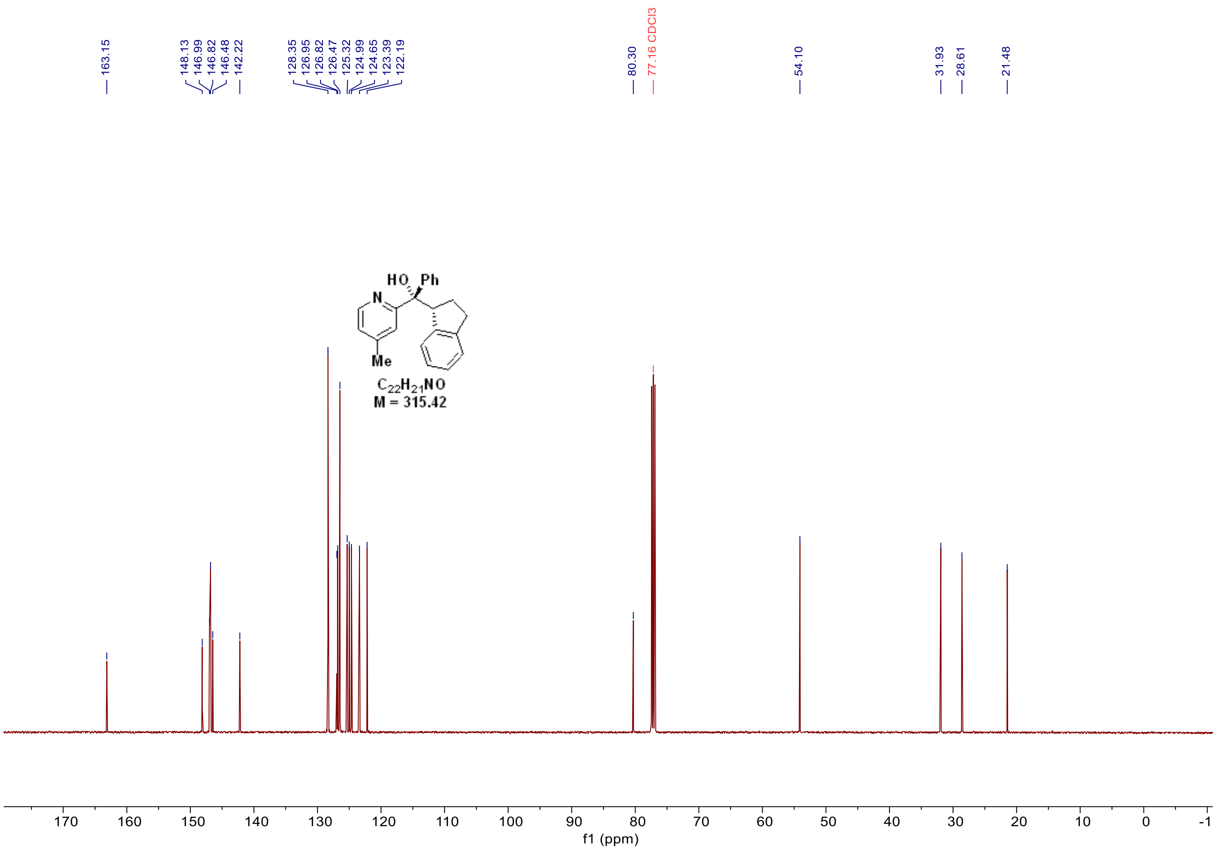

^{1}H NMR (500 MHz, CD_3Cl, 298 K) and ^{13}C NMR (125 MHz, CD_3Cl, 298 K) of **3ip**.

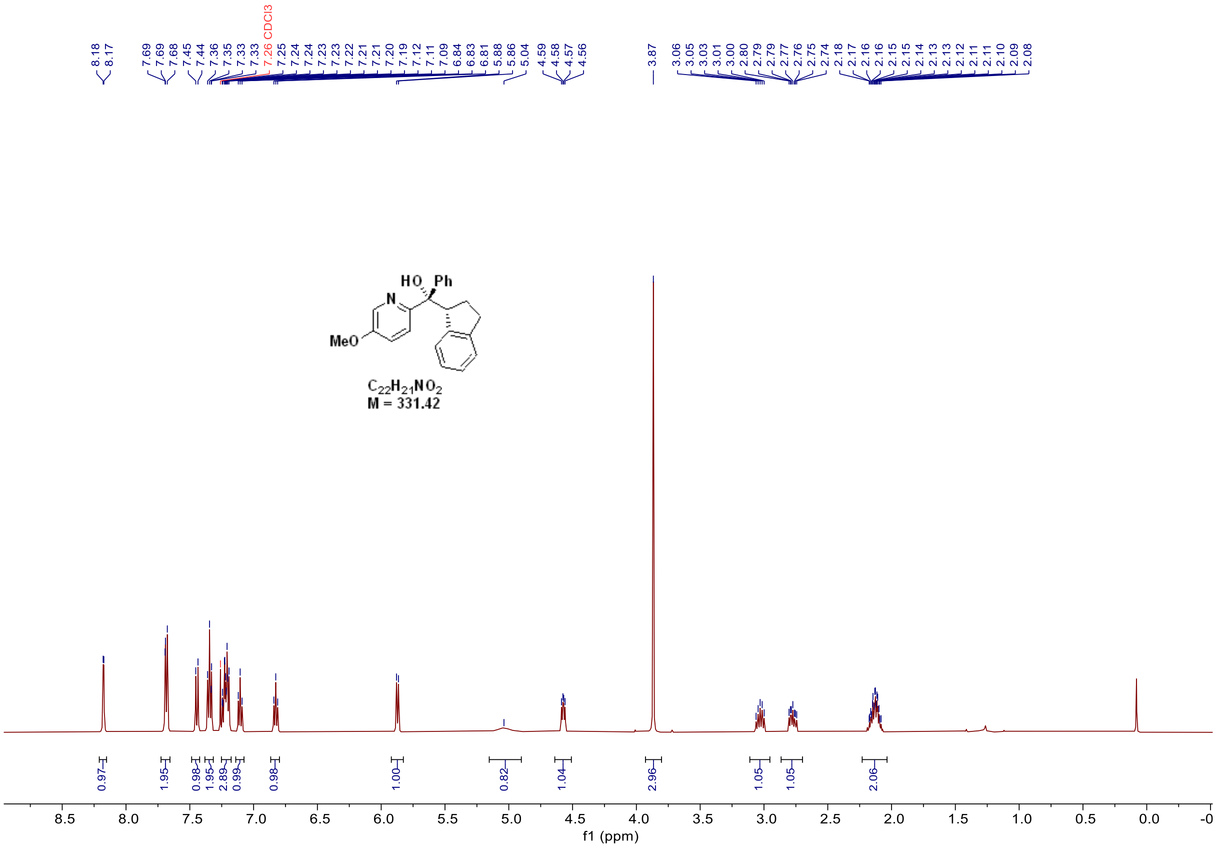

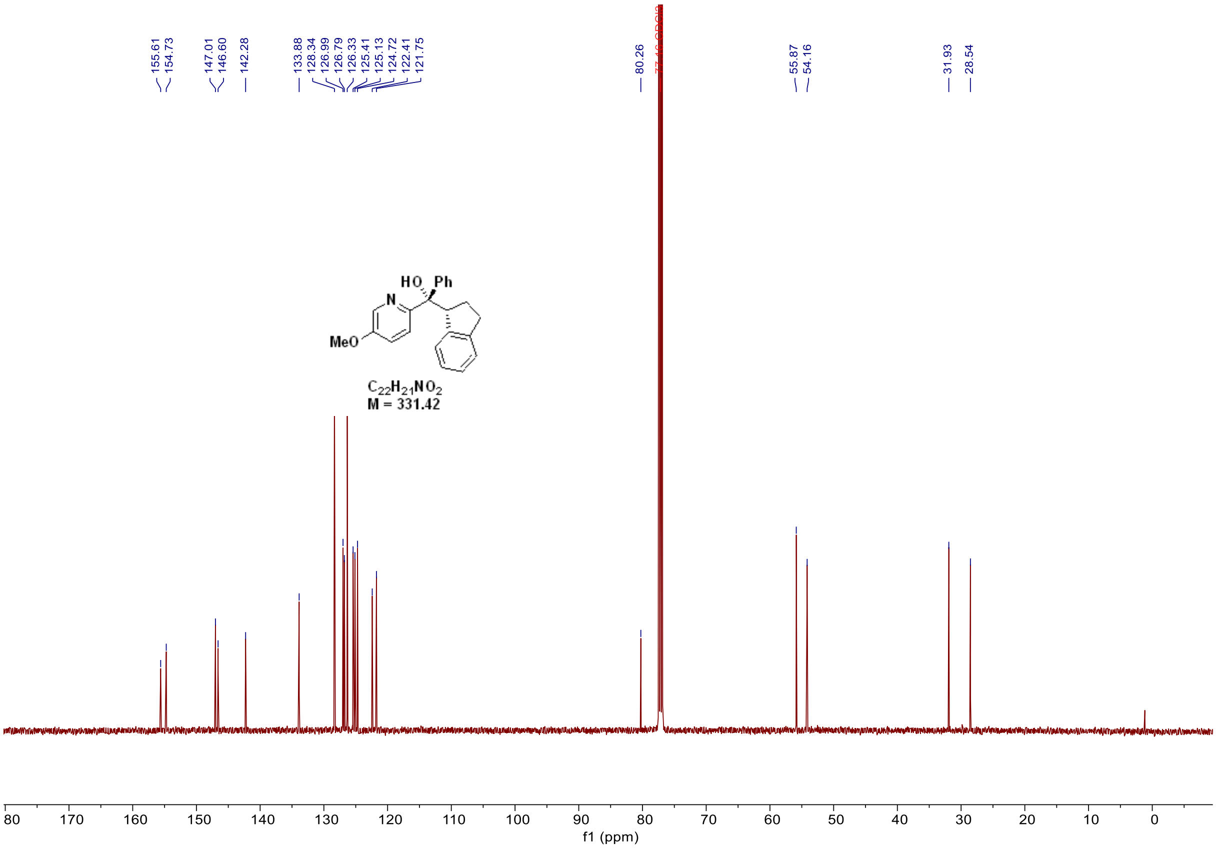

^{1}H NMR (500 MHz, CD_3Cl, 298 K), ^{13}C NMR (125 MHz, CD_3Cl, 298 K) and ^{19}F NMR (471 MHz, CD_3Cl, 298 K) of **3jp**.

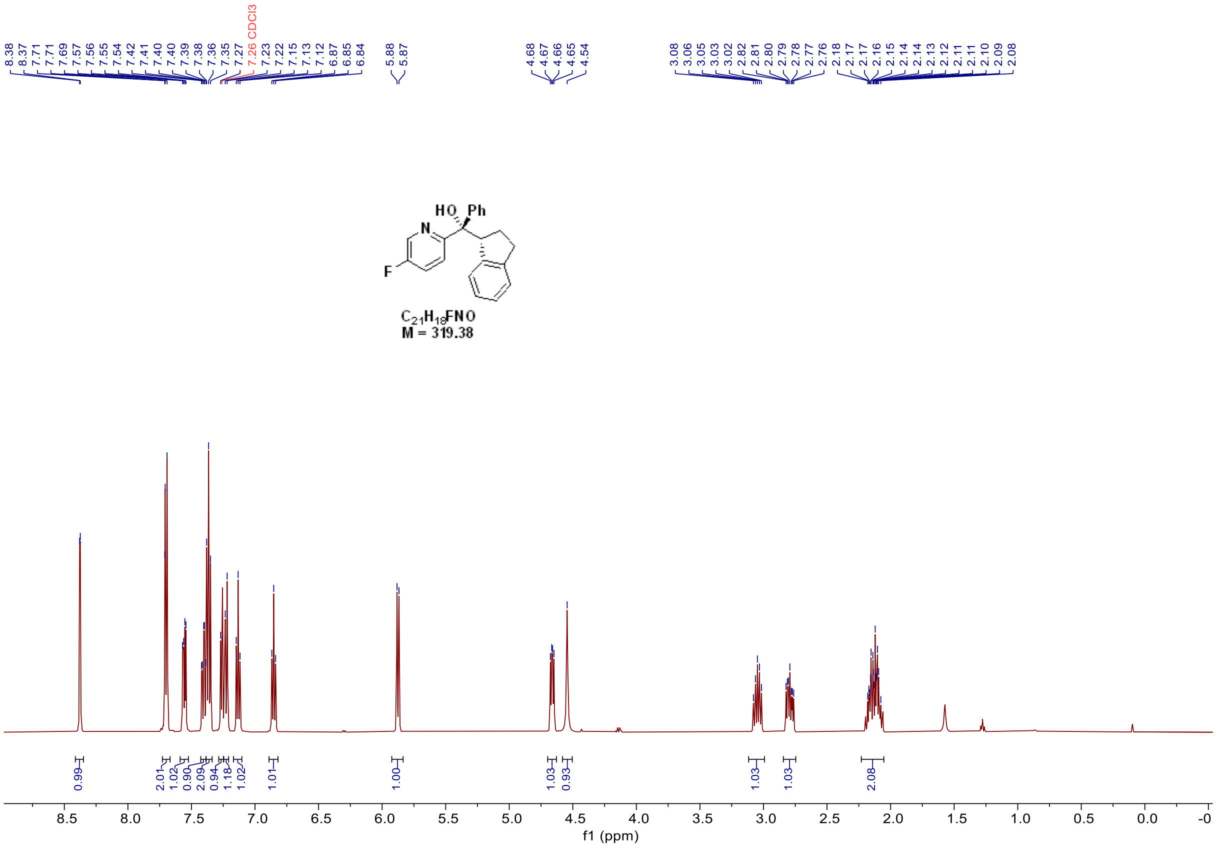

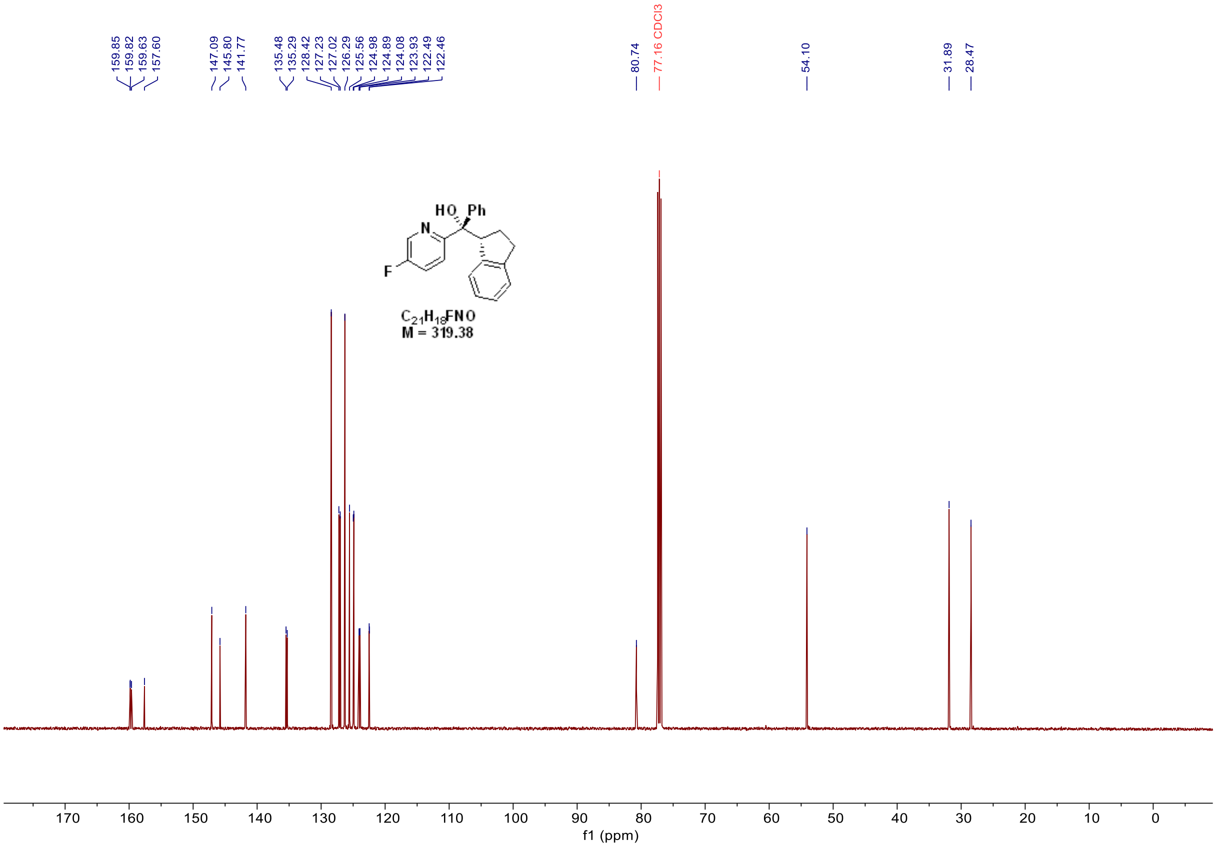

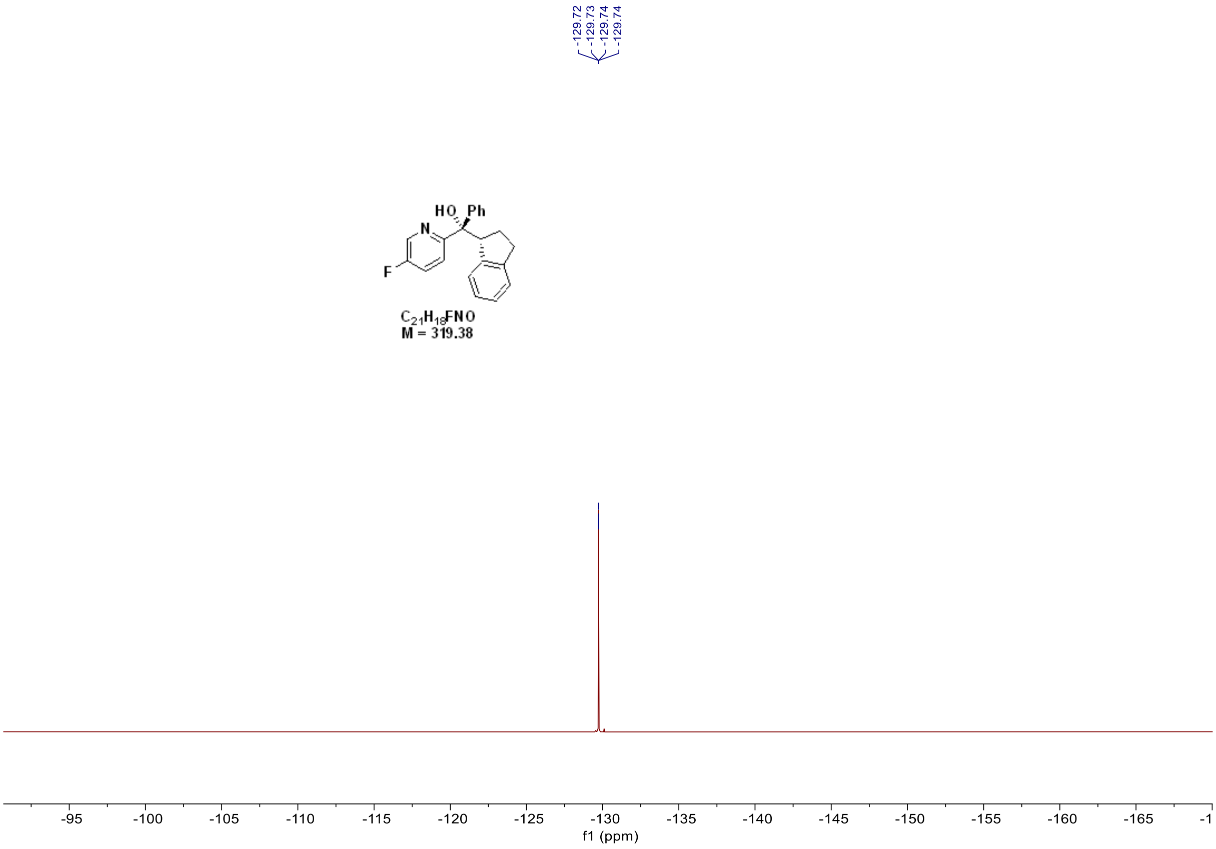
-129.72
-129.73
-129.74
-129.74
HO Ph
N
F
C21H18FNO
M = 319.38
-95
-100
-105
-110
-115
-120
-125
-130
-135
-140
-145
-150
-155
-160
-165
f1 (ppm)

^{1}H NMR (500 MHz, CD_3Cl, 298 K) and ^{13}C NMR (125 MHz, CD_3Cl, 298 K) of **3kp**.

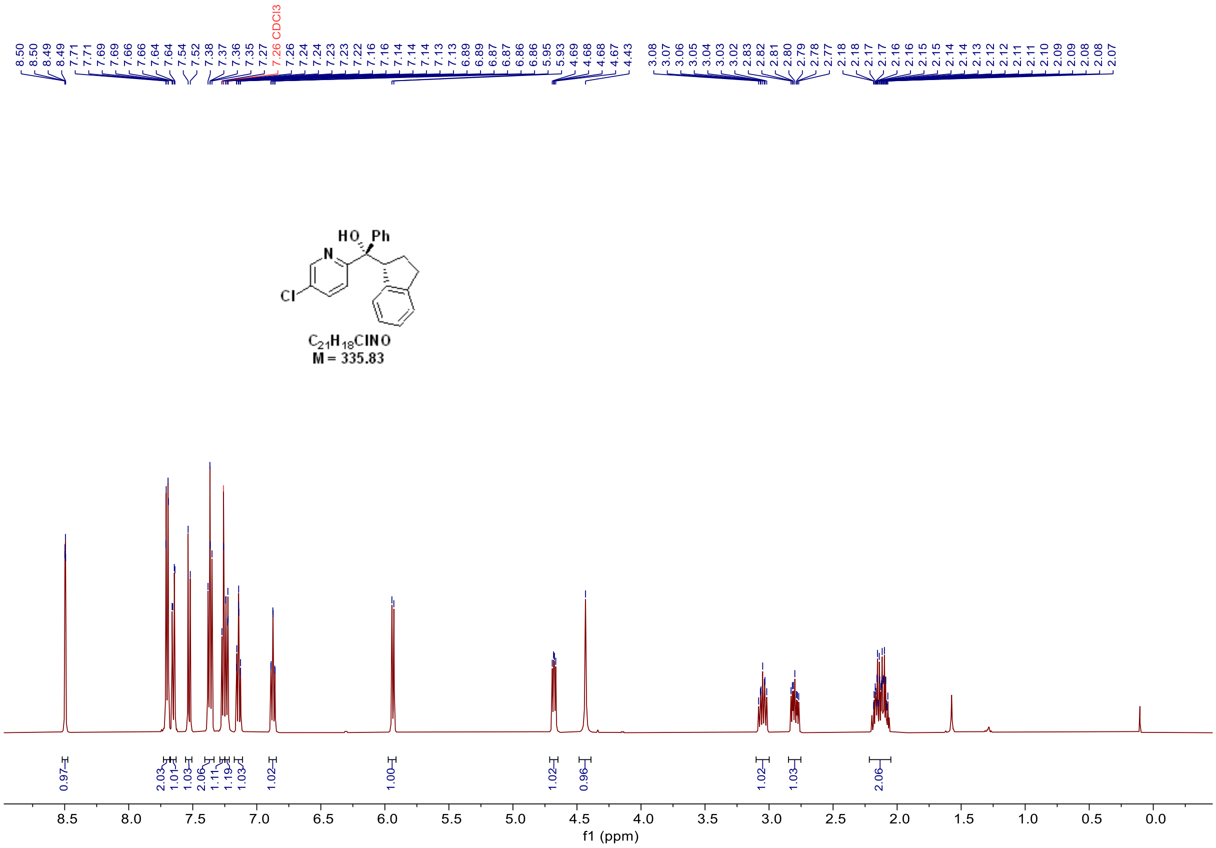

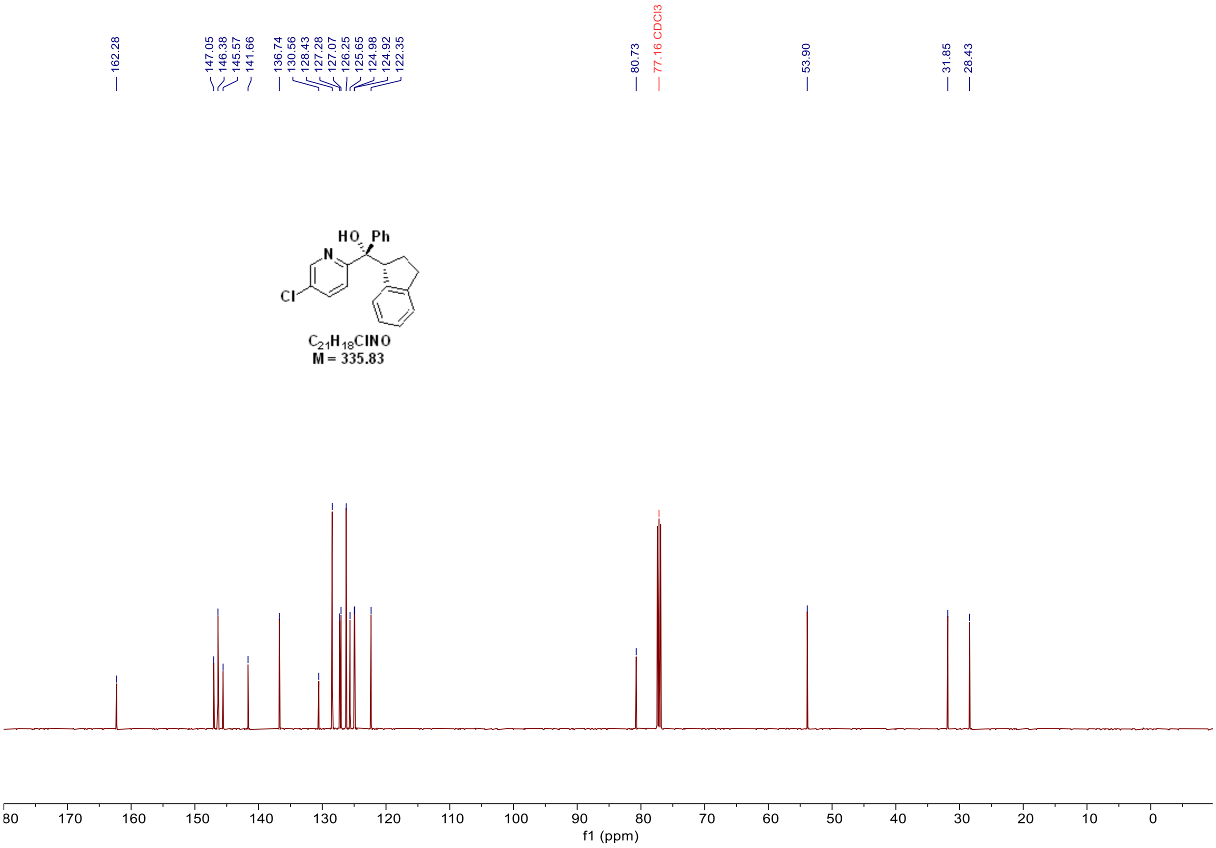

^{1}H NMR (500 MHz, CD_3Cl, 298 K) and ^{13}C NMR (125 MHz, CD_3Cl, 298 K) of **3lp**.

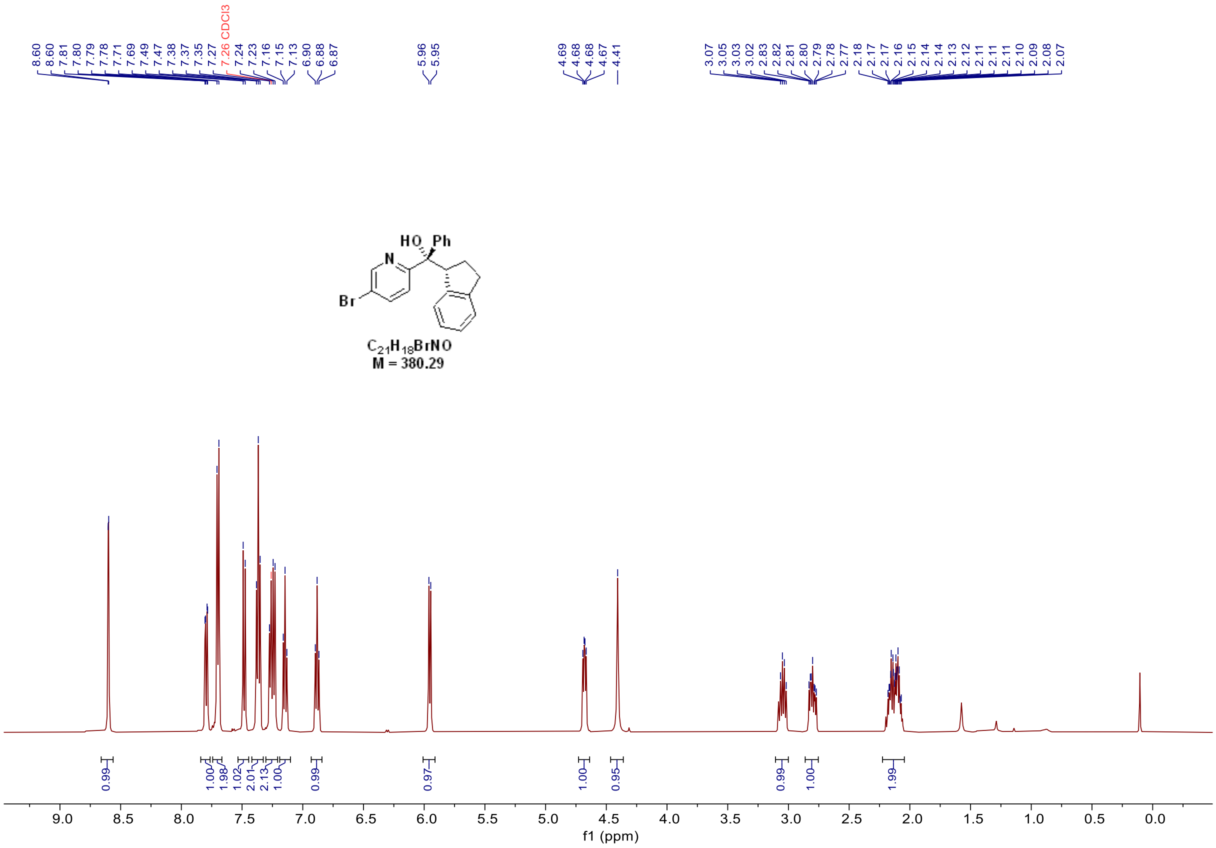

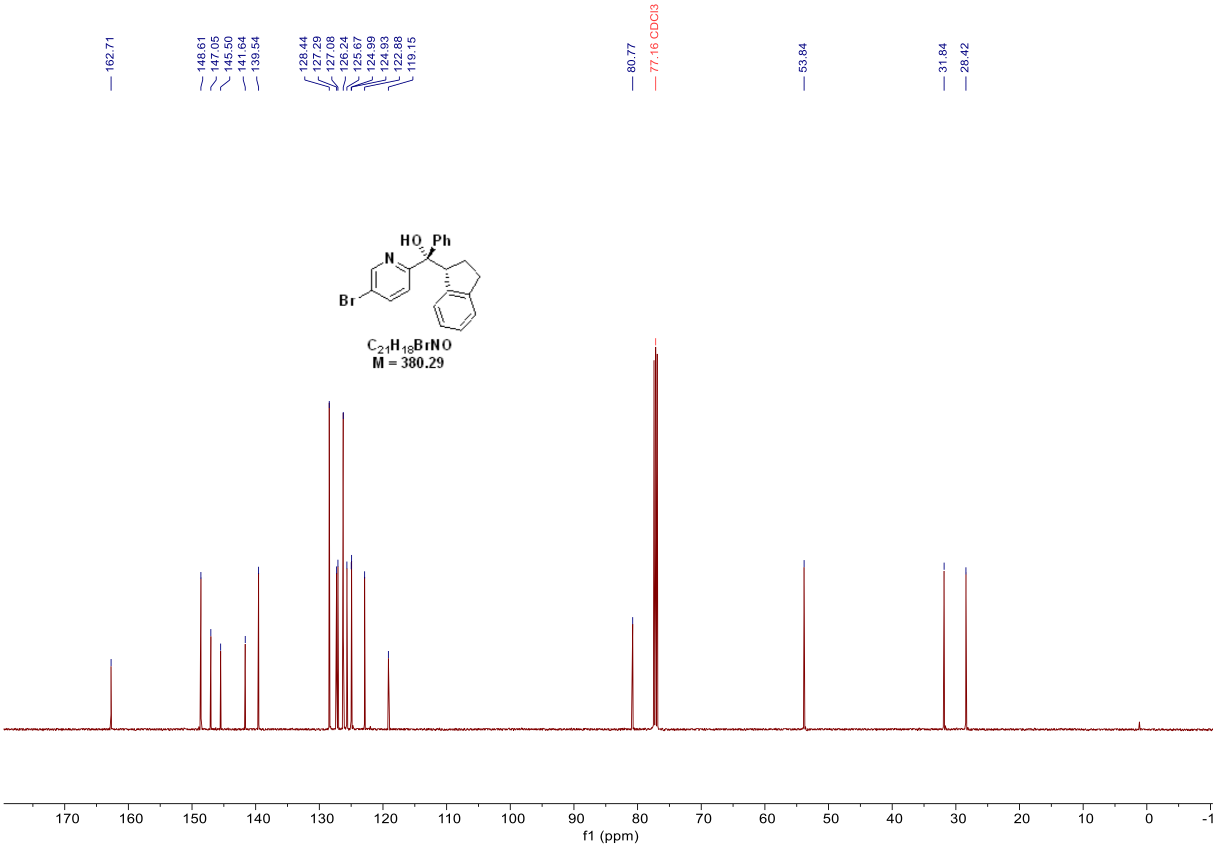

^{1}H NMR (500 MHz, CD_3Cl, 298 K), ^{13}C NMR (125 MHz, CD_3Cl, 298 K) and ^{19}F NMR (471 MHz, CD_3Cl, 298 K) of **3mp**.

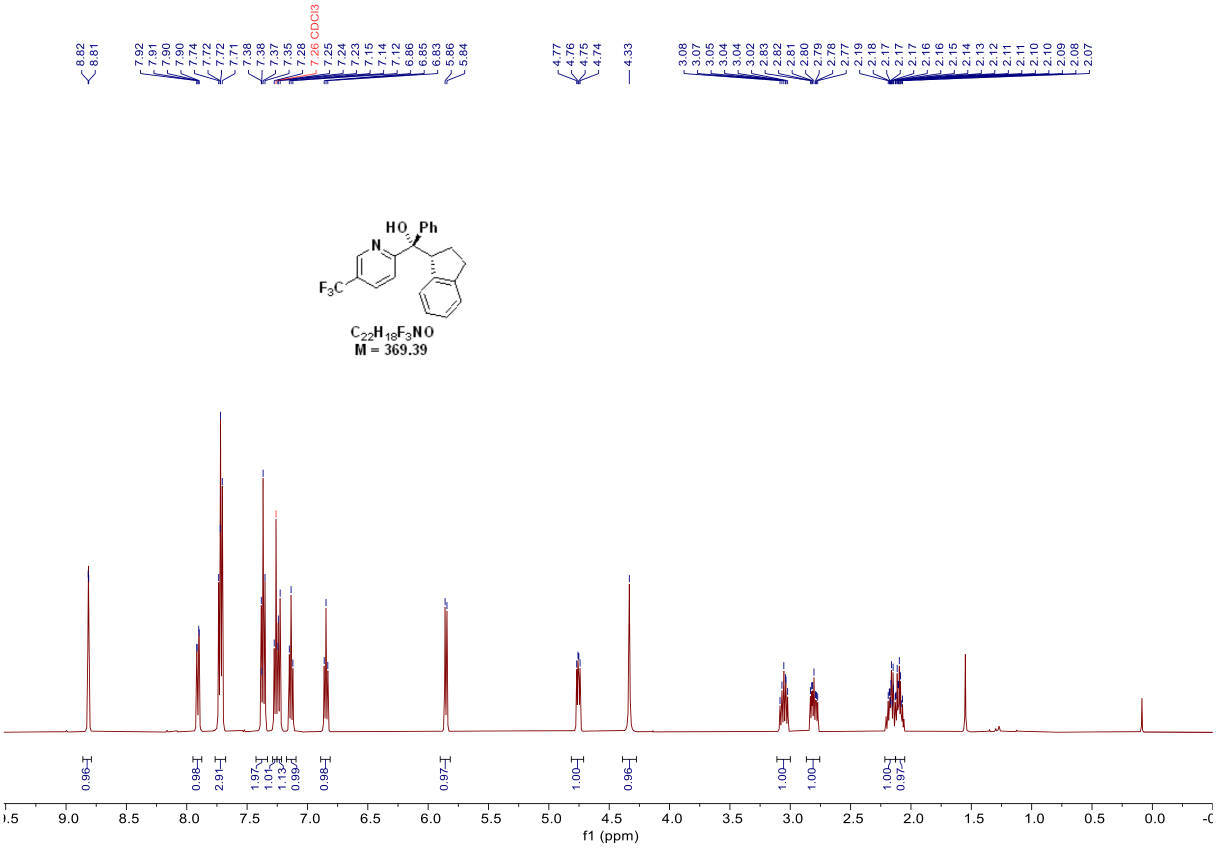

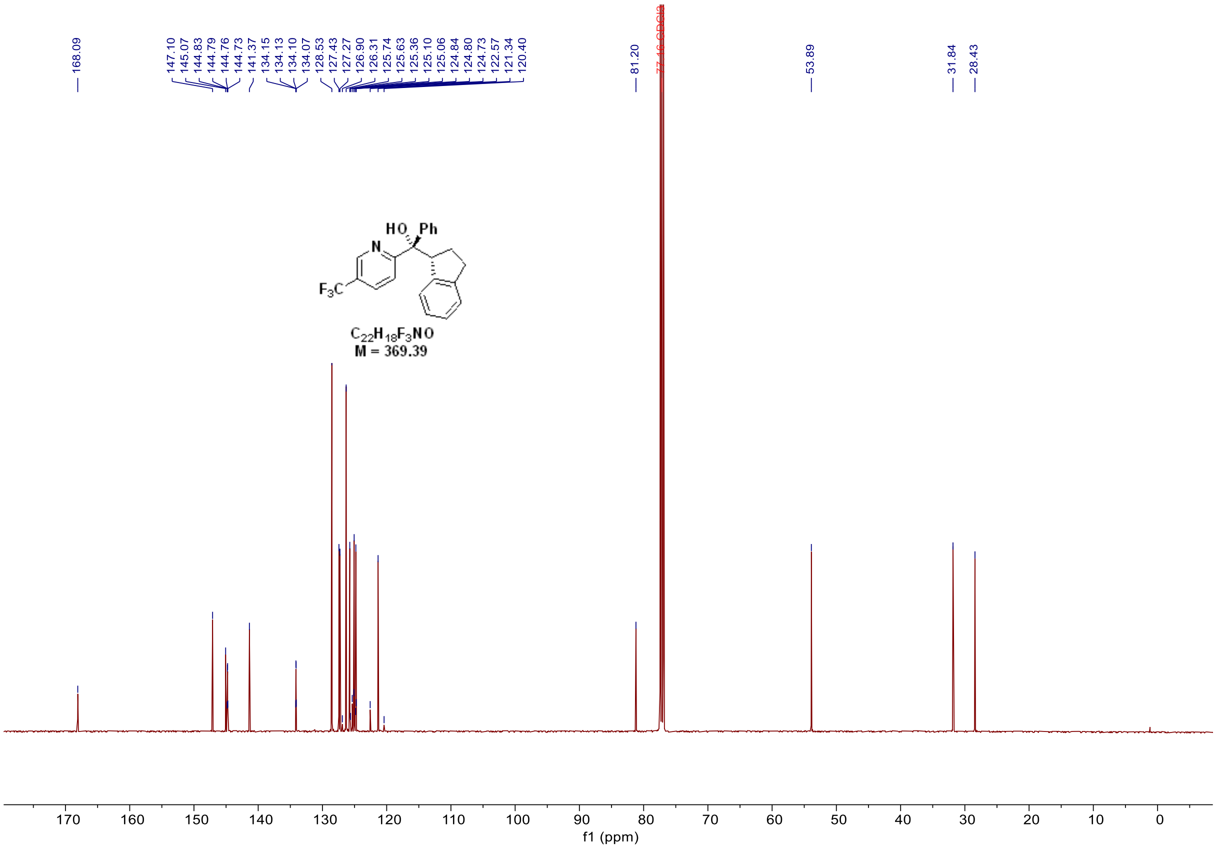

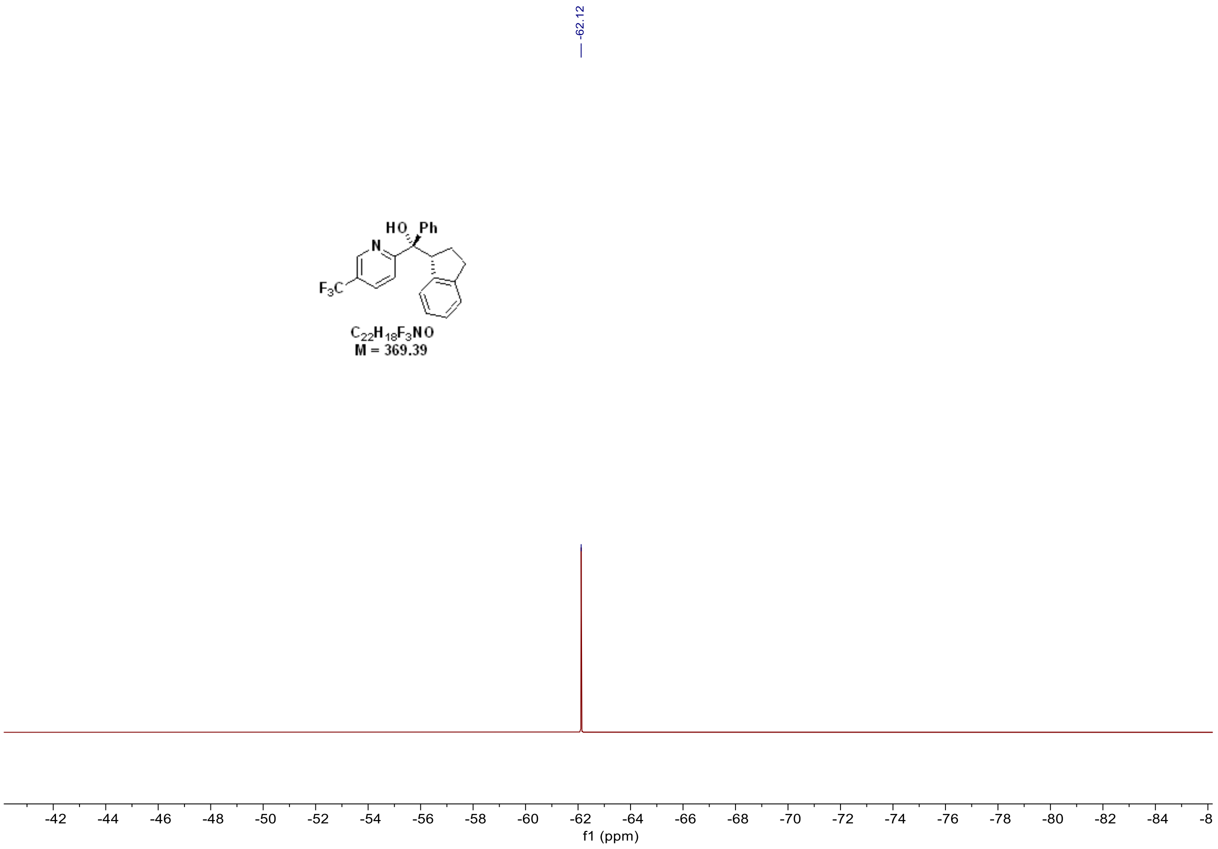
-62.12
HO
Ph
N
F3C
C22H18F3NO
M = 369.39
-42
-44
-46
-48
-50
-52
-54
-56
-58
-60
-62
-64
-66
-68
-70
-72
-74
-76
-78
-80
-82
-84
-8
f1 (ppm)

^{1}H NMR (500 MHz, CD_3Cl, 298 K) and ^{13}C NMR (125 MHz, CD_3Cl, 298 K) of **3np**.

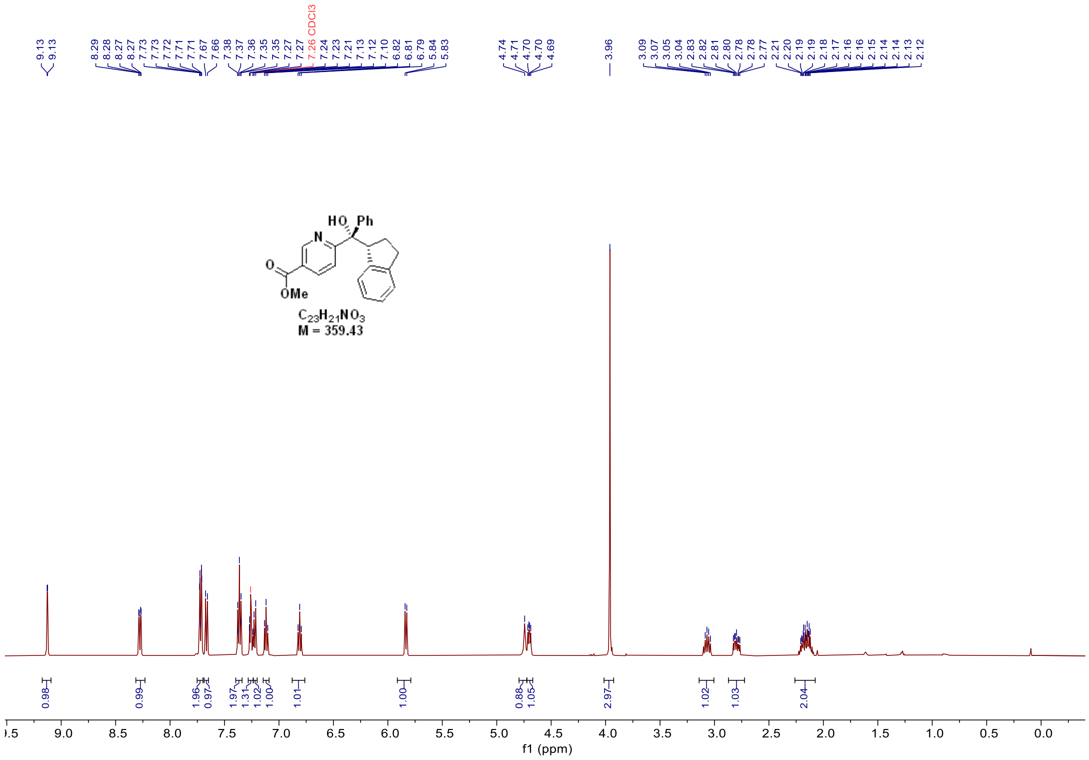

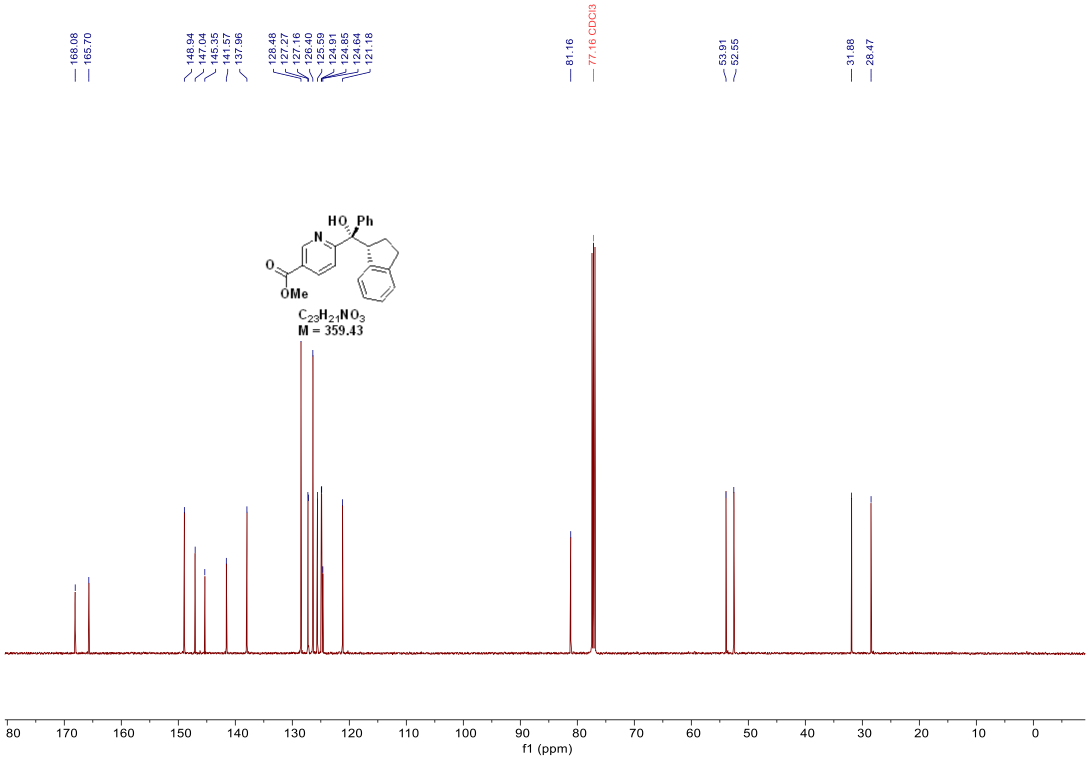

^{1}H NMR (500 MHz, CD_3Cl, 298 K) and ^{13}C NMR (125 MHz, CD_3Cl, 298 K) of **3op**.

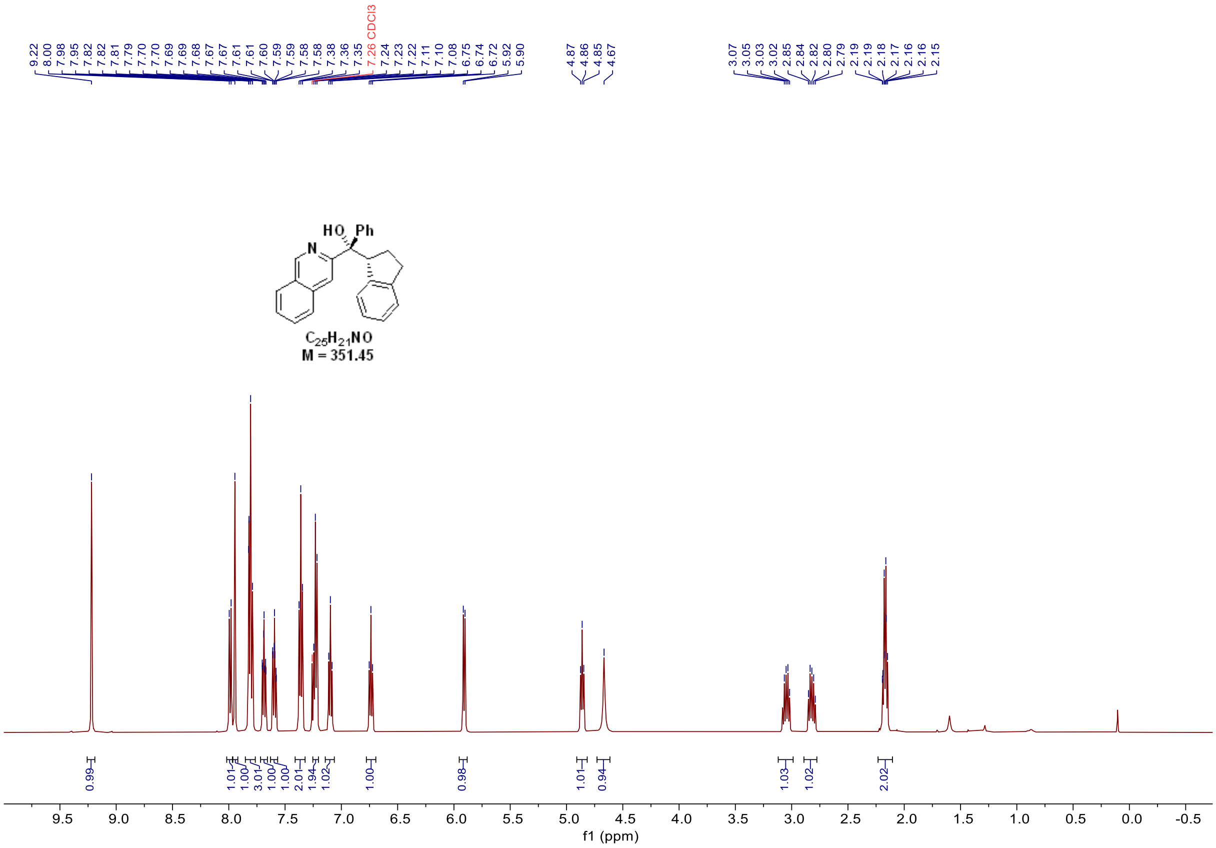

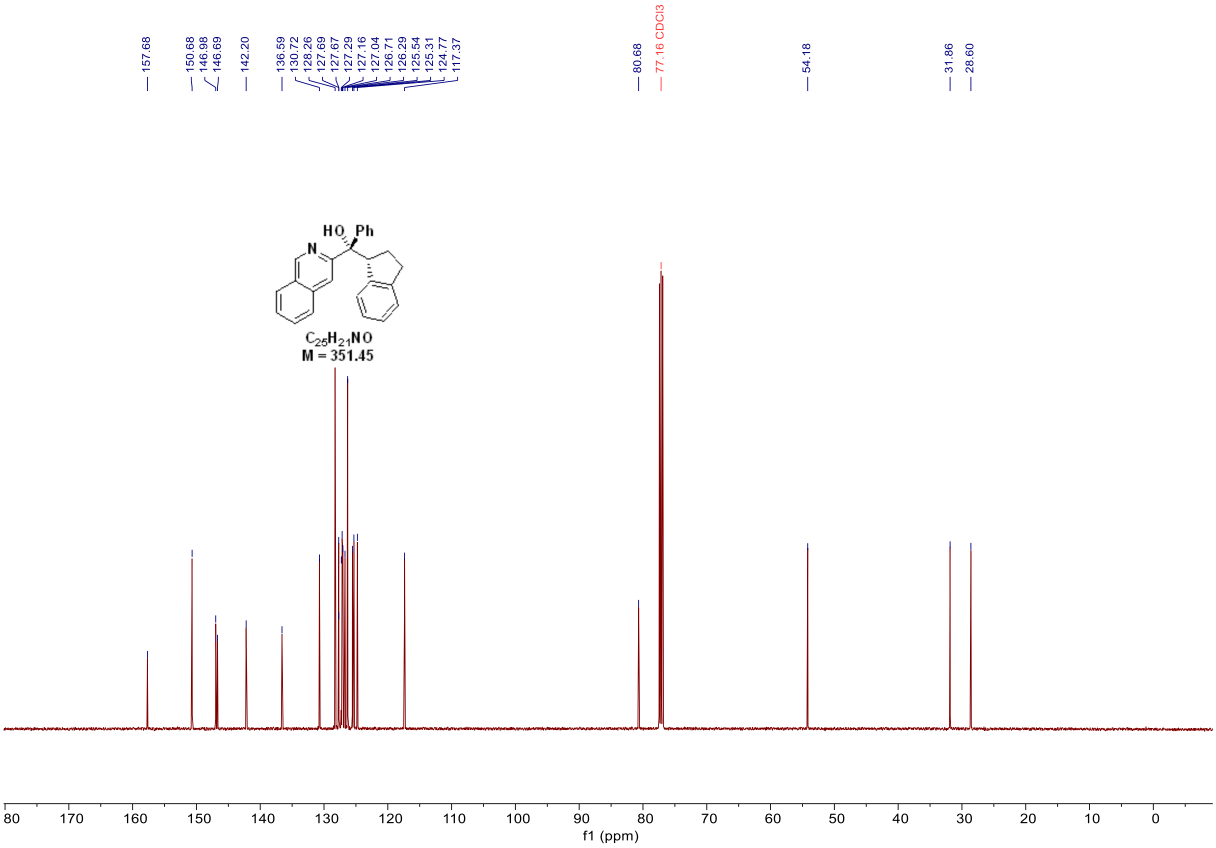

^{1}H NMR (500 MHz, CD_3Cl, 298 K) and ^{13}C NMR (125 MHz, CD_3Cl, 298 K) of **4a**.

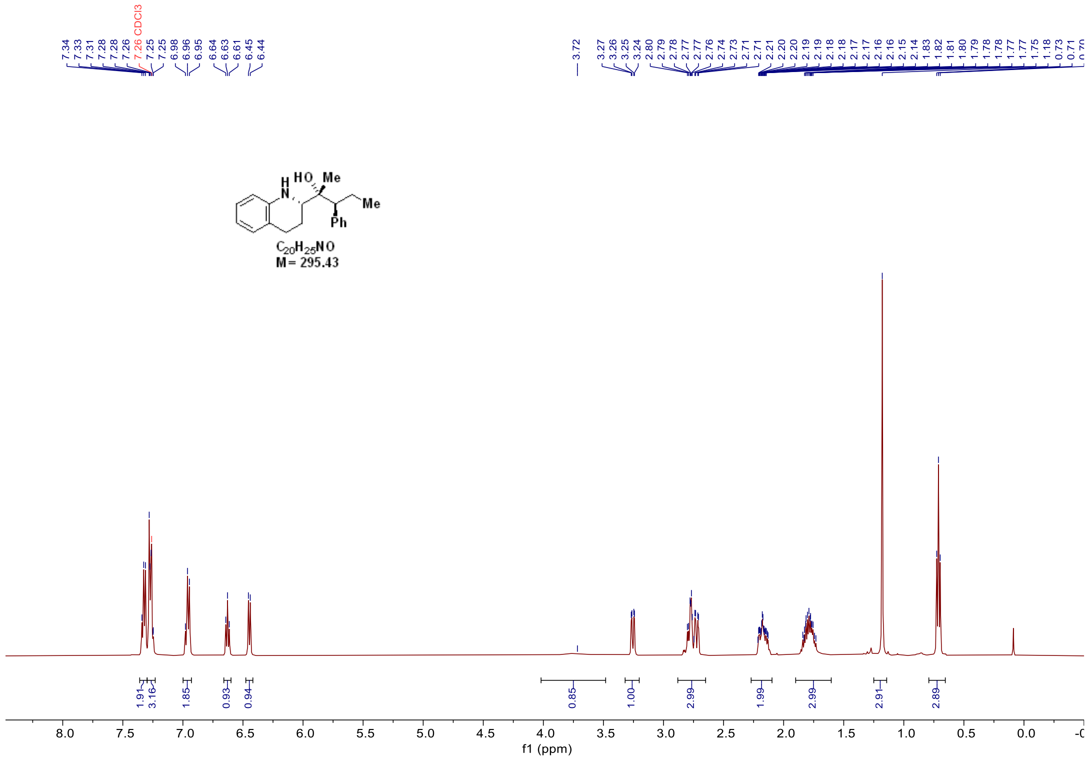

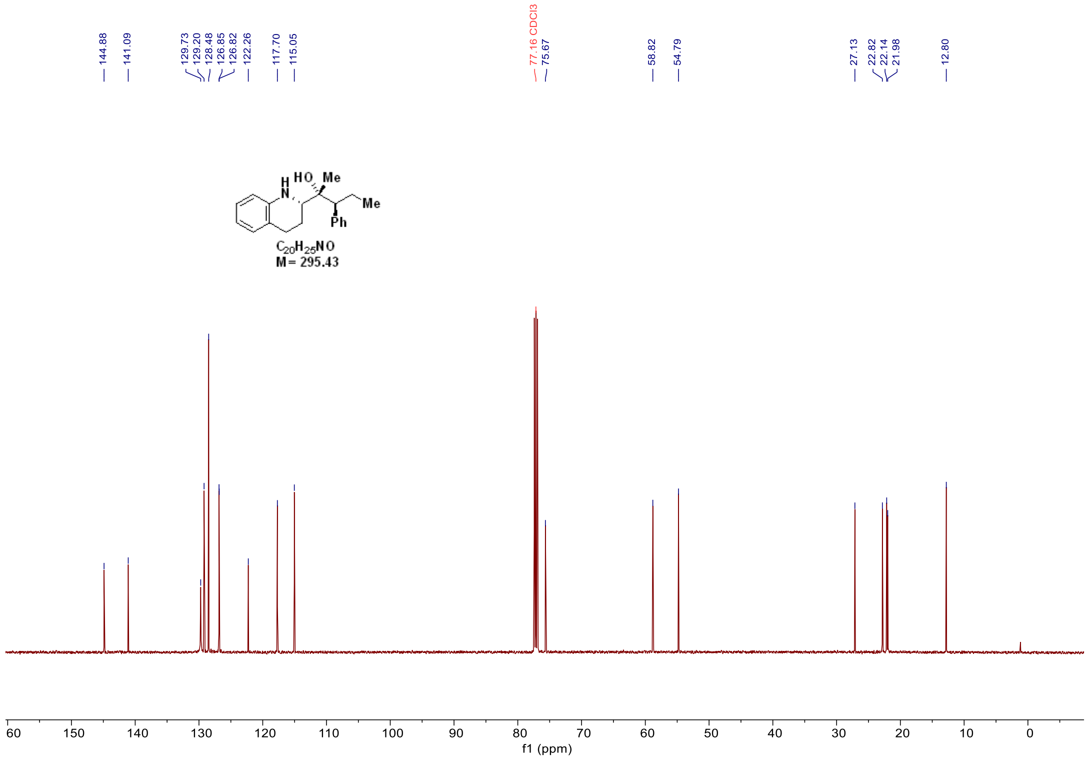

^{1}H NMR (500 MHz, CD_3Cl, 298 K) and ^{13}C NMR (125 MHz, CD_3Cl, 298 K) of **8a**.

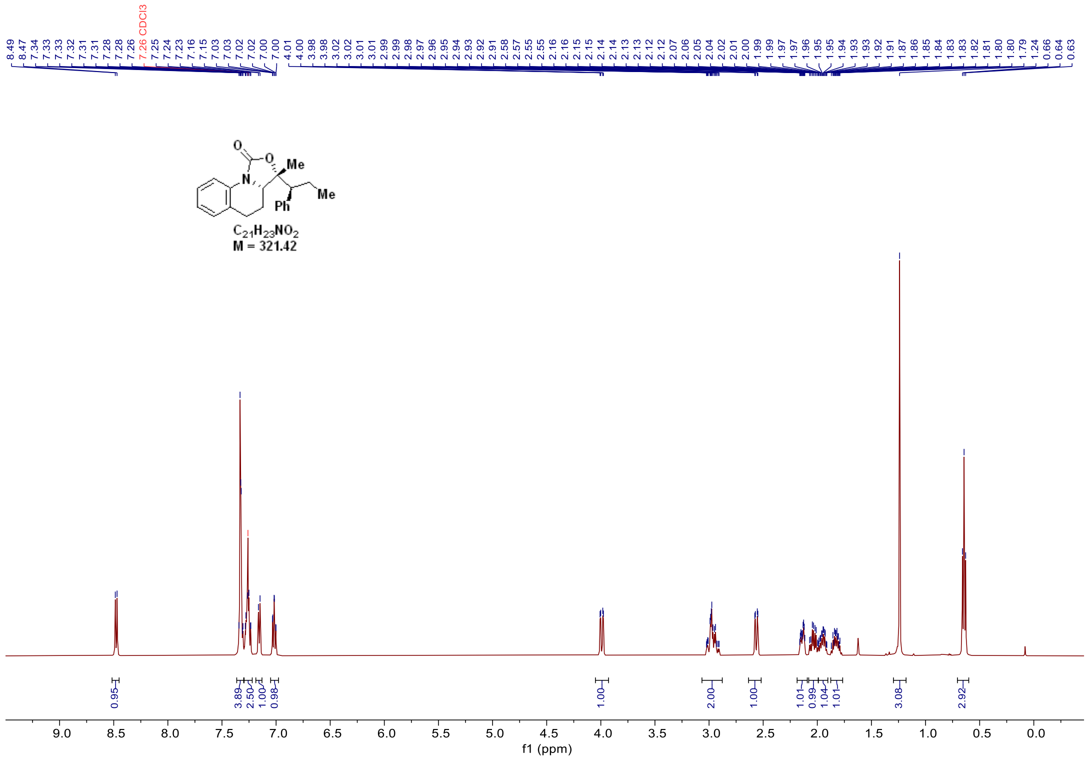

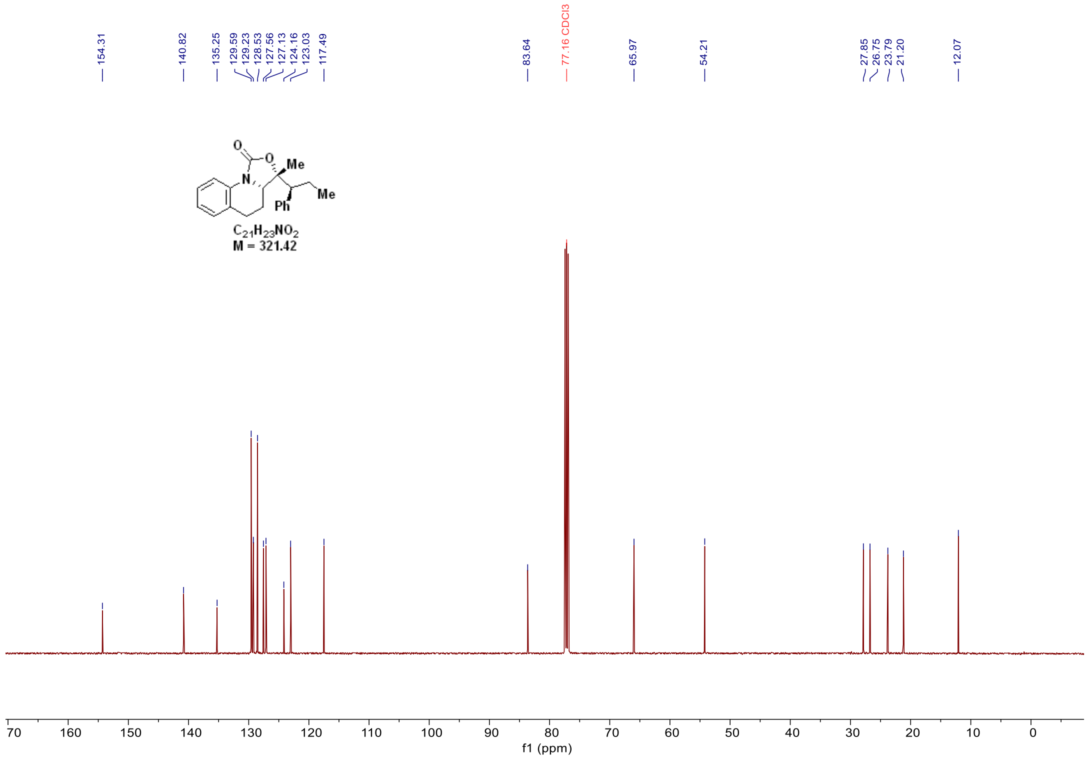

^{1}H NMR (500 MHz, CD_3Cl, 298 K) and ^{13}C NMR (125 MHz, CD_3Cl, 298 K) of **4b**.

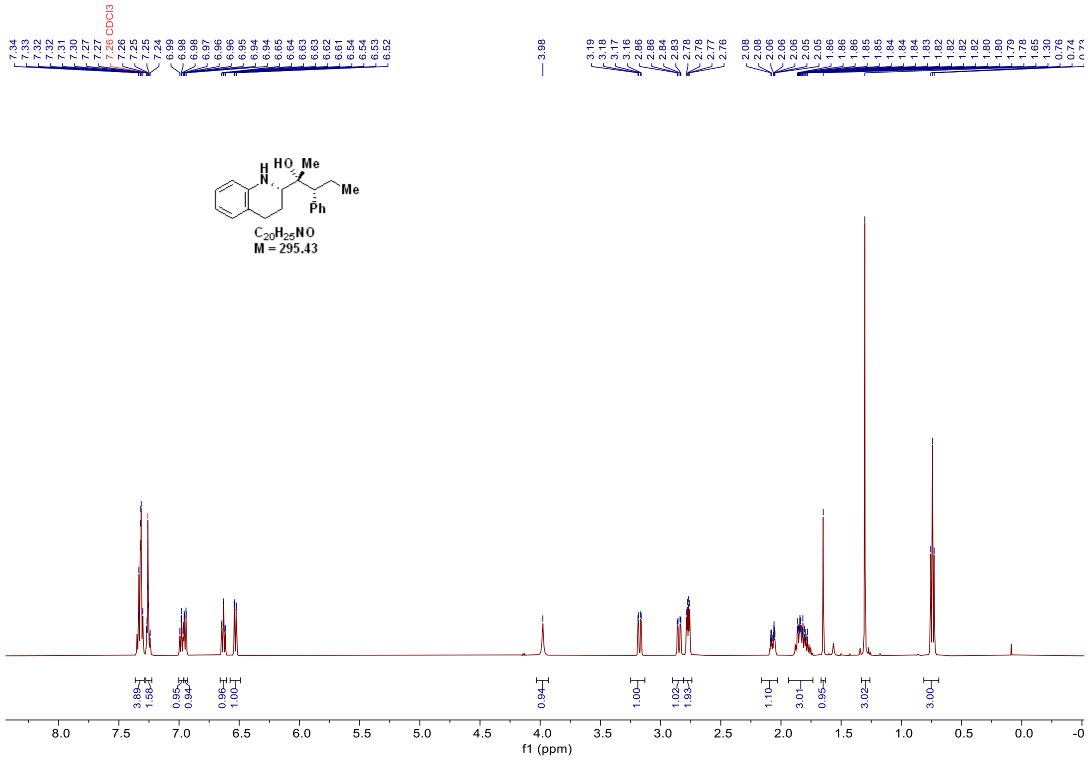

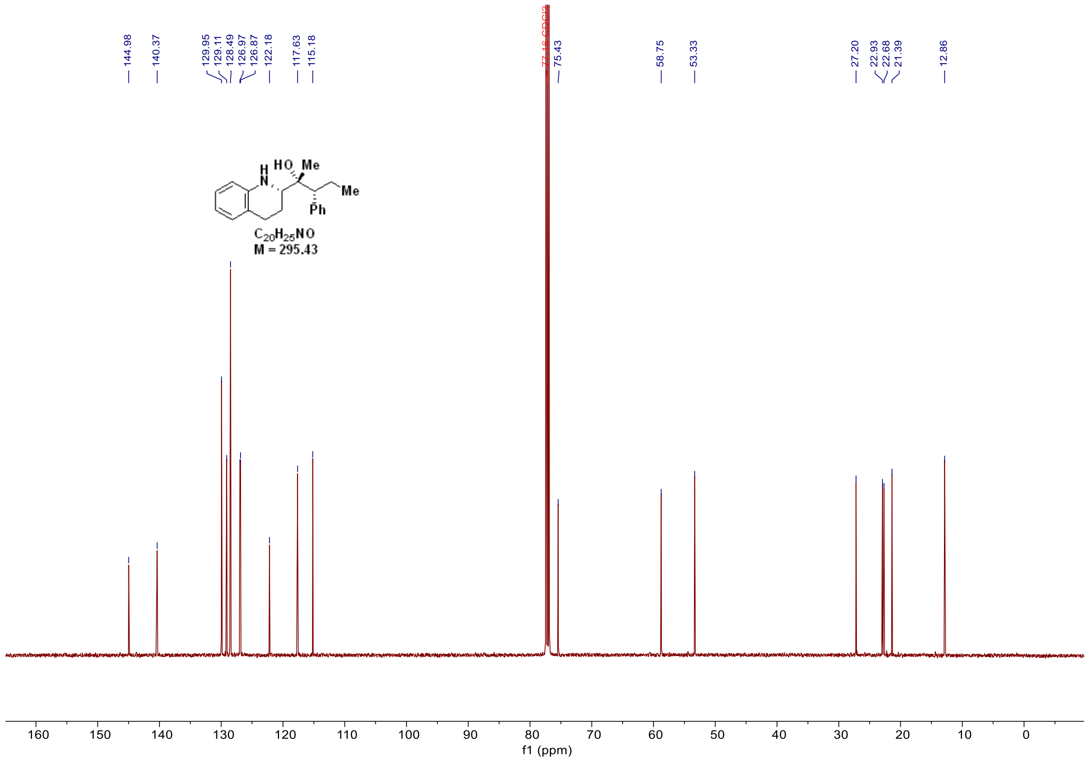

^{1}H NMR (500 MHz, CD_3Cl, 298 K) and ^{13}C NMR (125 MHz, CD_3Cl, 298 K) of **8b**.

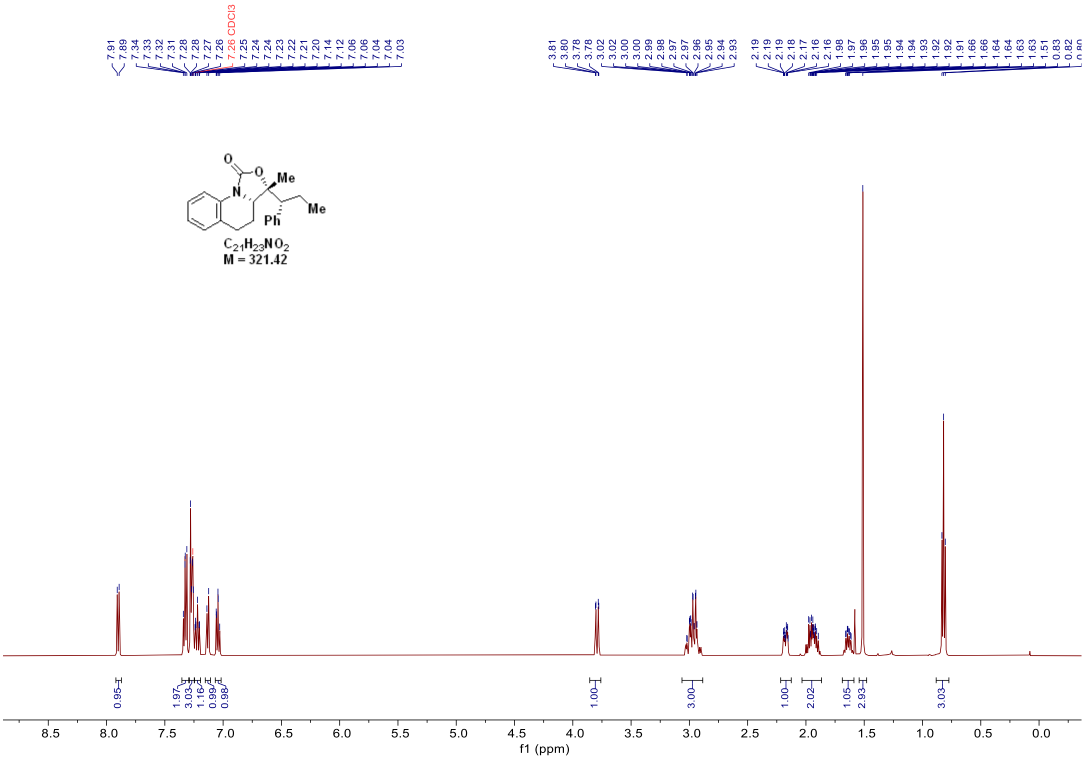

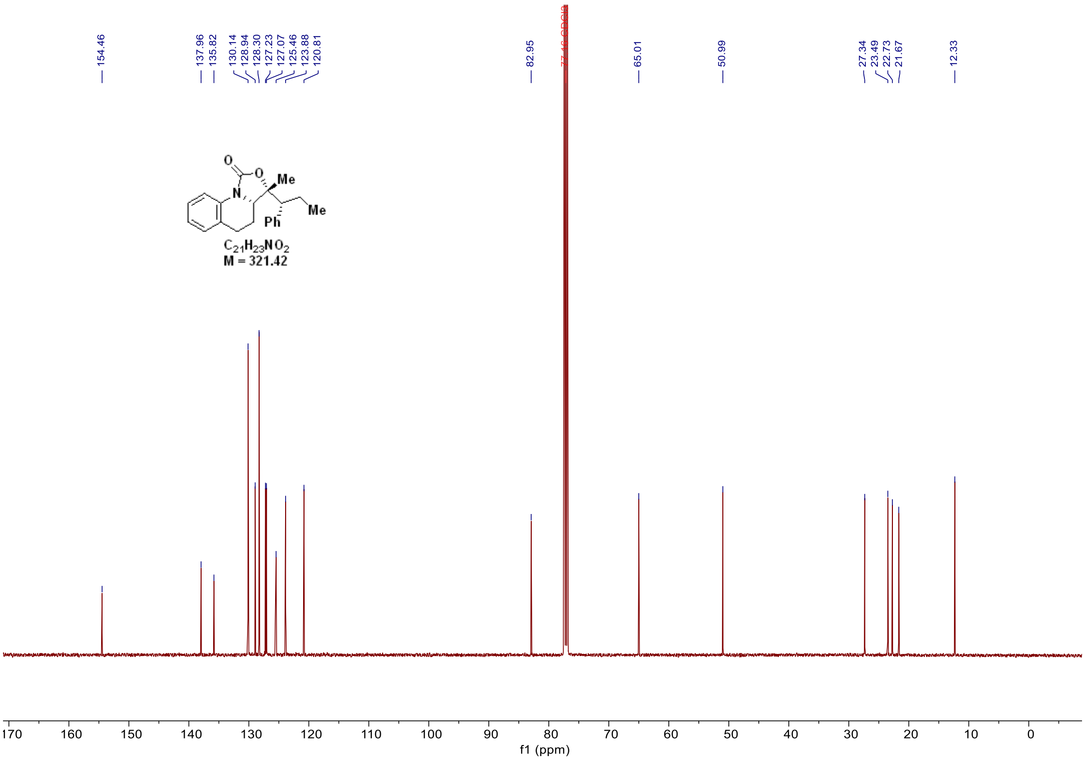

^1H NMR (500 MHz, CD_3Cl, 298 K) and ^{13}C NMR (125 MHz, CD_3Cl, 298 K) of **5**.

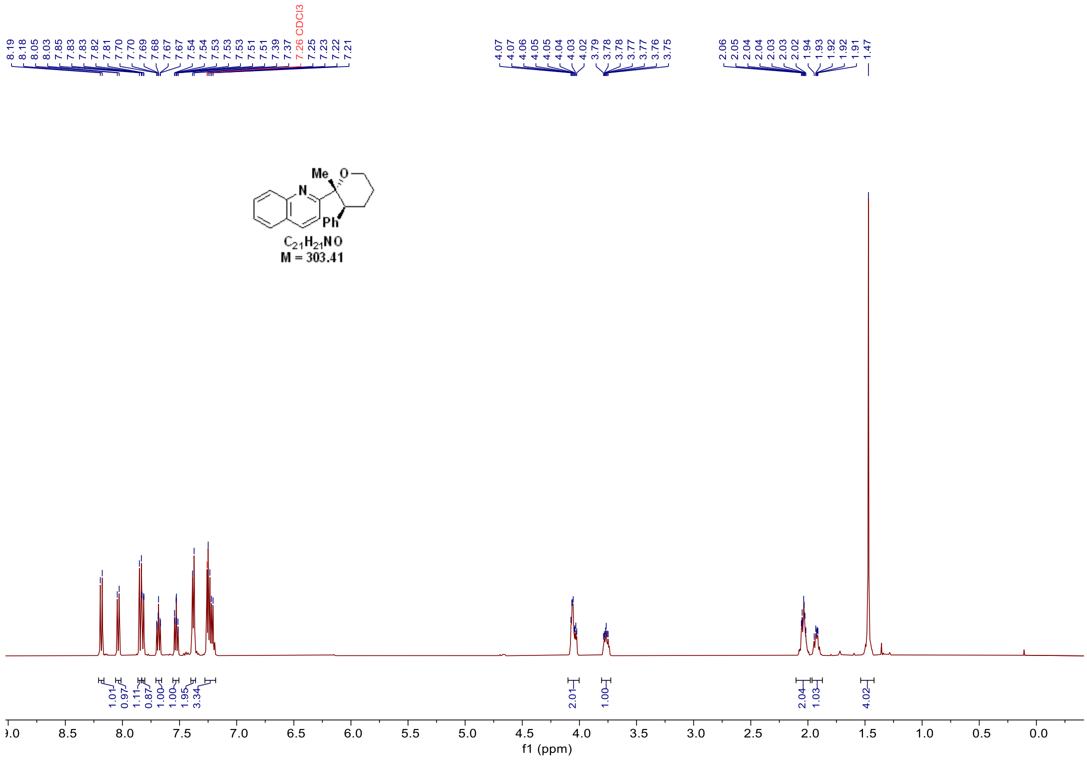

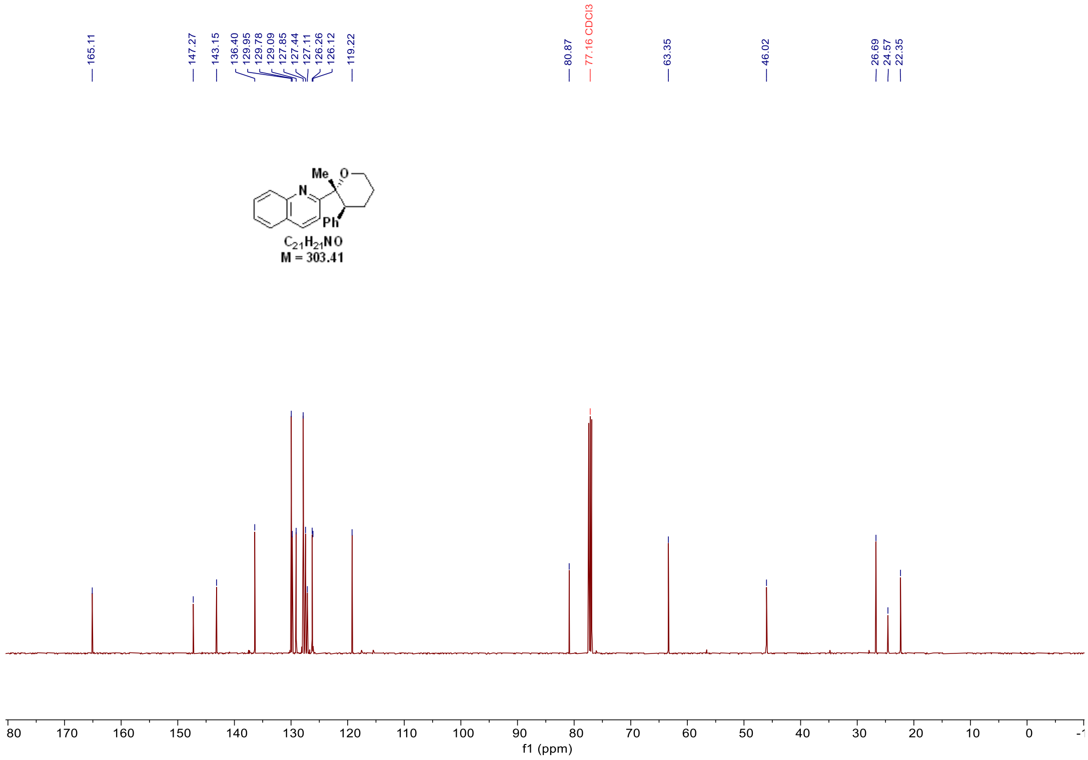

^{1}H NMR (500 MHz, CD_3Cl, 298 K) and ^{13}C NMR (125 MHz, CD_3Cl, 298 K) of **9**.

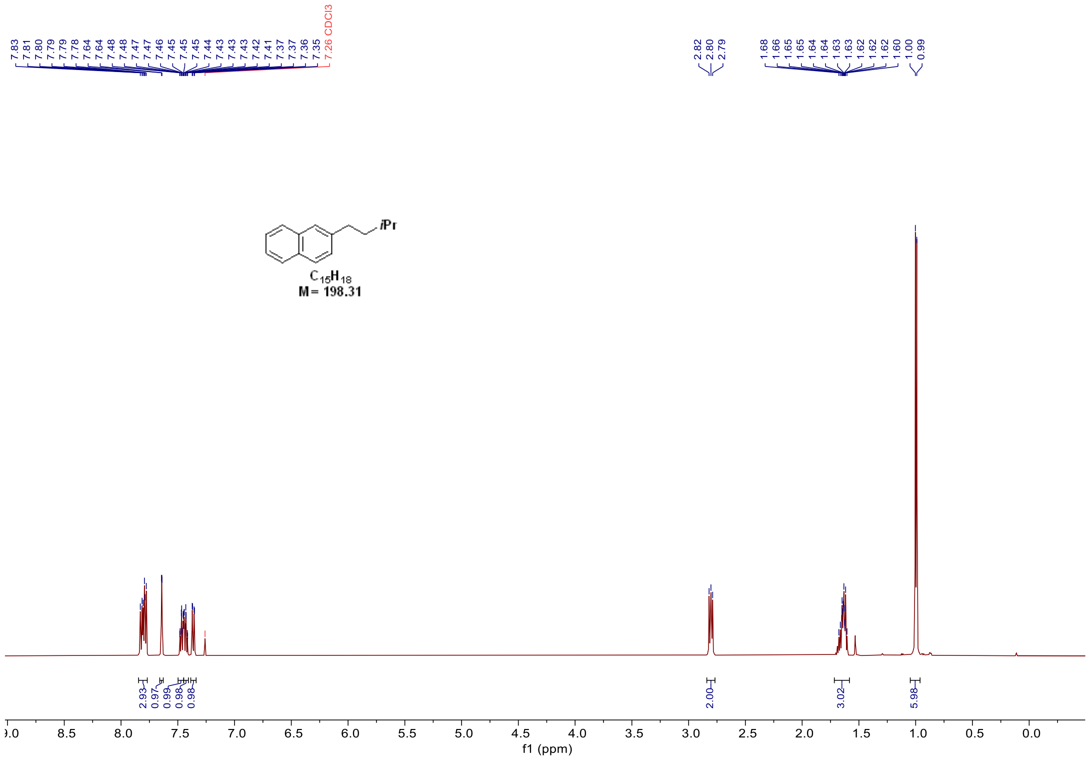

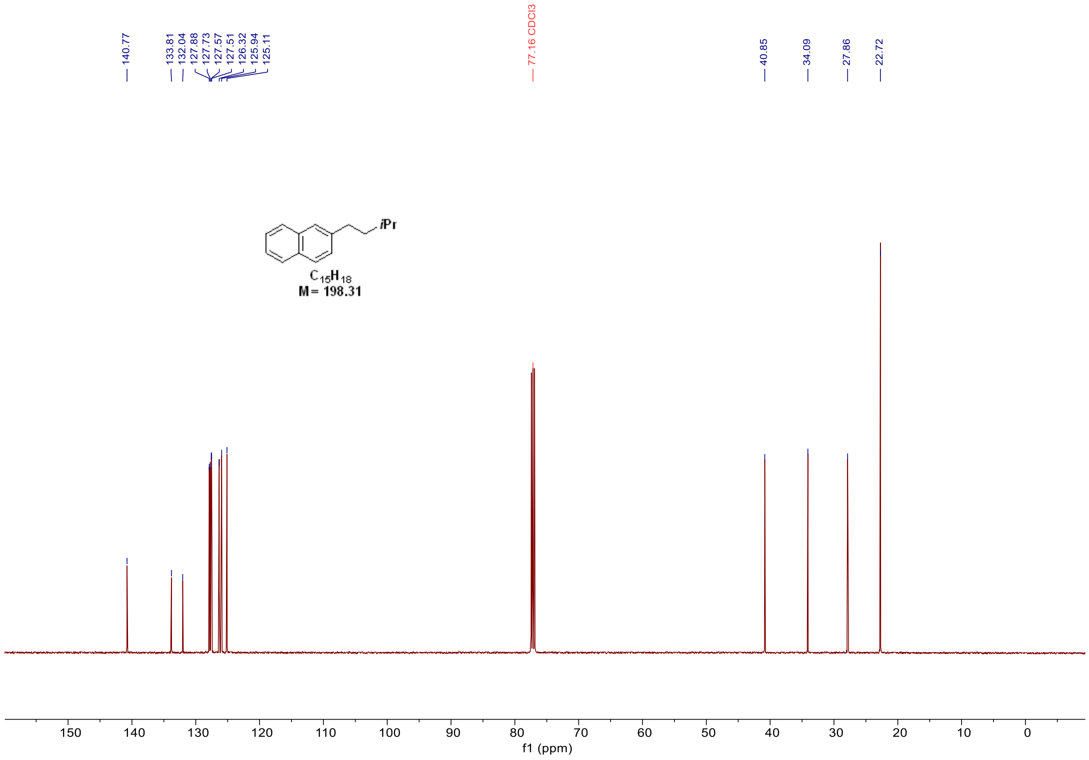

^{1}H NMR (500 MHz, CD_3Cl, 298 K) and ^{13}C NMR (125 MHz, CD_3Cl, 298 K) of **1-(quinolin-2-yl)ethan-1-one (1a-H$_2$)**.

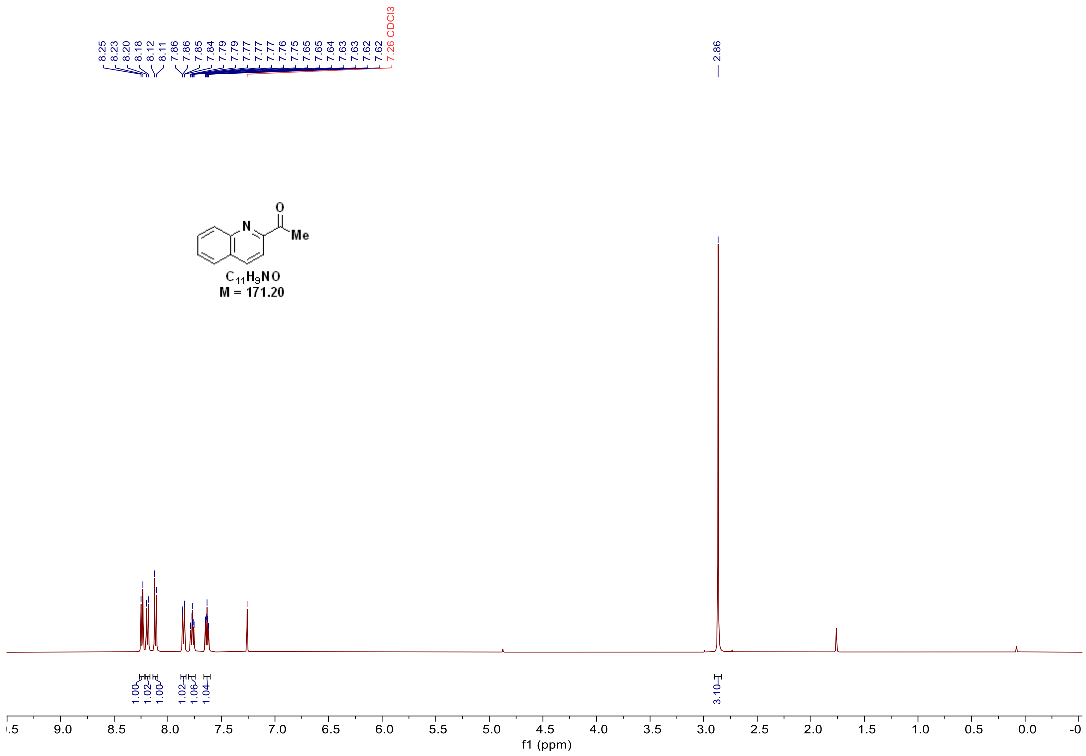

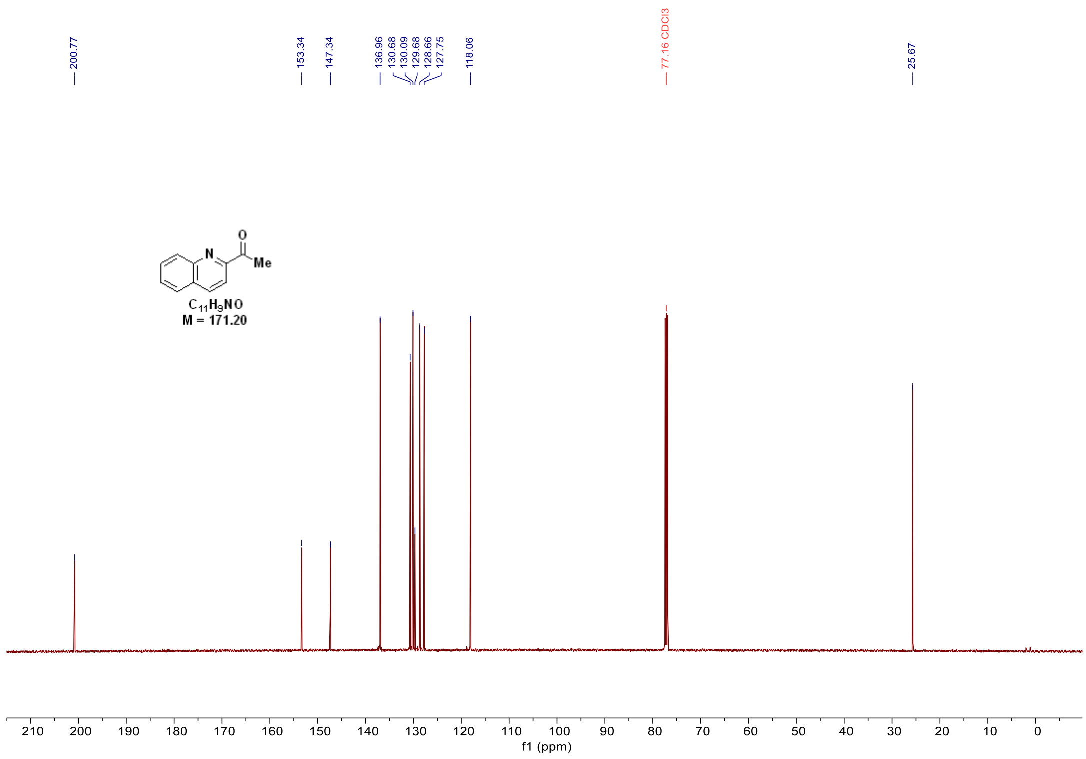

8 References

[1] Cao, Y.; Zhang, S.; Antilla, J. C. *ACS Catal*. **2020**, *10*, 10914–10919.

[2] (a) Gómez, I.; Alonso, E.; Ramón, D. J.; Yus, M. *Tetrahedron* **2000**, *56*, 4043–4052. (b) Fakhfakh, M. A.; Fournet, A.; Prina, E.; Mouscadet, J.-F.; Franck, X.; Hocquemillera, R.; Figadère, B. *Bioorg*. *Med*. *Chem*. **2003**, *11*, 5013–5023. (c) Chen, F.; He, D.; Chen, L.; Chang, X.; Wang, D. Z.; Xu, C.; Xing, X. *ACS Catal*. **2019**, *9*, 5562–5566. (d) Nian, S.; Ling, F Chen, J.; Wang, Z.; Shen, H.; Yi, X.; Yang, Y.-F.; She, Y.; Zhong, W. *Org*. *Lett*. **2019**, *21*, 5392–5396. (e) Yu, R.; Hao, F.; Zhang, X.; Fang, Z.; Jin, Z.; Liu, G.; Dai, G.; Wu, J. *J*. *Org*. *Chem*. **2023**, *88*, 8279–8285.

[3] Roy, S.; Das, S. K.; Chattopadhyay, B. *Angew*. *Chem*. *Int*. *Ed*. **2018**, *57*, 2238–2243.

[4] (a) Brown, E. V.; Shambhu, M. B. *J*. *Org*. *Chem*. **1971**, *36*, 2002–2004. (b) Wang, X.; Wang, D. Z. *Tetrahedron* **2011**, *67*, 3406–3411. (c) Yang, H.; Huo, N.; Yang, P.; Pei, H.; Lv, H.; Zhang, X. *Org*. *Lett*. **2015**, *17*, 4144–4147. (d) Wang, Z.; Zhao, X.; Wang, S.; Huang, A.; Wang, Y.; He, J.; Ling, F.; Zhong, W. *Org*. *Biomol*. *Chem*. **2021**, *19*, 9746–9751. (e) Xu, X.; You, Y.; Jin, M. Y.; Meng, F.-J.; Xu, C.; Xing, X. *Sci*. *China Chem*. **2023**, *66*, 1443–1449.

[5] Zhong, S.; Deng, G.-J.; Dai, Z.; Huang, H. *Org*. *Chem*. *Front*. **2021**, *8*, 4419–4425.

[6] Guanti, G.; Riva, R. *Tetrahedron*: *Asymmetry* **2001**, *12*, 1185–1200.

[7] (a) Kiyokawa, K.; Ishizuka, M.; Minakata, S. *Angew*. *Chem*. *Int*. *Ed*. **2023**, *62*, e202218743. (b) Cheng, D.; Xia, H.; Gu, H.; Wang, Y.; Li, J.-H.; Xu, X. *Synthesis* **2023**, *55*, 4011–4019.

[8] Yue, F.; Dong, J.; Liu, Y.; Wang, Q. *Org. Biomol. Chem.* **2021**, *19*, 8924–8928.

[9] Crisenza, G. E. M.; McCreanor, N. G.; Bower, J. F. *J*. *Am*. *Chem*. *Soc*. **2014**, *136*, 10258–10261.

[10] (a) Tang, W.; Sun, Y.; Xu, L.; Wang, T.; Fan, Q.; Lamc, K.-H.; Chanc, A. S. C. *Org*. *Biomol*. *Chem*. **2010**, *8*, 3464–3471. (b) Cao, J.; Su, Y.-X.; Zhang, X.-Y.; Zhu, S.-F. *Angew*. *Chem*. *Int*. *Ed*. **2023**, *62*, e202212976.

[11] Bertolini, F.; Crotti, S.; Di Bussolo, V.; Macchia, F.; Pineschi, M. *J. Org. Chem.* **2008**, *73*, 8998–9007.

[12] Wang, F.-F.; Luo, C.-P.; Wang, Y.; Deng, G.; Yang, L. *Org. Biomol. Chem.* **2012**, *10*, 8605–8608.

[13] Nielsen, C. D. T.; Burés, J. *Chem. Sci.* **2019**, *10*, 348–353.

[14] Dolomanov, O.V.; Bourhis, L.J.; Gildea, R. J.; Howard, J. A. K.; Puschmann, H. *J. Appl. Cryst.* **2009**, *42*, 339–341.

[15] Sheldrick, G. M. *Acta Cryst.* **2015**, *A71*, 3–8.

[16] Sheldrick, G.M. *Acta Cryst.* **2015**, *C71*, 3–8.
